# Redundant and receptor-specific activities of TRADD, RIPK1 and FADD in death receptor signaling

**DOI:** 10.1038/s41419-019-1396-5

**Published:** 2019-02-11

**Authors:** Simone Füllsack, Alevtina Rosenthal, Harald Wajant, Daniela Siegmund

**Affiliations:** Division of Molecular Internal Medicine, Department of Internal Medicine II, University Hospital Würzburg, Würzburg, Germany

## Abstract

We evaluated redundant and receptor-specific activities of TRADD, RIPK1, and FADD in RIPK3-expressing HeLa cells lacking expression of these proteins or any combination of two of these factors. We confirmed the opposing role of FADD in TNF- and TRAIL-induced necroptosis and observed an anti-necroptotic function of TRADD. RIPK1 and TRADD act in a redundant manner in TNF- but not TRAIL-induced apoptosis. Complementary, FADD proved to be sufficient for TRAIL- but not for TNF-induced apoptosis. TRADD and RIPK1, however, redundantly mediated proinflammatory signaling in response to TNF and TRAIL. FADD deficiency sensitized more efficiently for TNFR1-mediated necroptosis than caspase-8 deficiency pointing to a caspase-8 independent inhibitory activity of FADD on TNF-induced necroptosis. Based on these characteristics, we propose a model in which the death receptor-specific activities of TRADD, RIPK1, and FADD are traced back to their hierarchically different position in TNFR1- and TRAIL death receptor signaling.

## Introduction

The death domain (DD) has been originally recognized due to its relevance for apoptosis induction by CD95 (Fas/APO-1) and tumor necrosis factor (TNF) receptor 1 (TNFR1)^1,2^, but is also present in the CD95-related death receptors TNF-related death-inducing ligand (TRAIL) receptor 1 (TRAILR1, also called death receptor 4 (DR4)) and TRAILR2/DR5 (ref. ^3^). The DD-containing adapter proteins TNFR1-associated death domain protein (TRADD) and Fas associated death domain protein (FADD) and the DD-containing serine/threonine kinase receptor interacting protein (RIPK1) have been isolated and cloned by virtue of their binding to TNFR1 and CD95^4–7^. While TRADD and RIPK1 are readily recruited into the liganded TNFR1 signaling complex, these molecules are not or only poorly detectable in the receptor signaling complexes of CD95, TRAILR1, and TRAILR2 (refs. ^8–10^). Complementary, FADD tightly binds to CD95 and the TRAIL death receptors in a ligand-dependent fashion, while it is not part of the plasma membrane-associated TNFR1 signaling complex^9^. Nevertheless, TRADD, FADD, and RIPK1 have all been implicated in signaling by each of the mentioned DD-containing receptors.

The huge majority of studies revealed an essential role of FADD in caspase activation and apoptosis induction by TNFR1, CD95, and the TRAIL death receptors^11–17^. A few reports, however, failed to see an effect of reduced/defective FADD expression on TNF-^8^ or TRAILR1-induced apoptosis^18^. FADD is furthermore of differential relevance for nuclear factor of kappaB (NFκB) signaling and necroptosis induction by death receptors. With respect to activation of NFκB transcription factors by CD95 and the TRAIL death receptors, FADD has been found to be an essential factor while it is dispensable for this response in the case of TNFR1^19–23^. Similarly, FADD fulfills a crucial role in TRAIL death receptor- and CD95-induced necroptosis but is not required for necroptotic TNFR1 signaling^24^. Moreover, FADD has even an inhibitory effect on TNF-induced necroptosis^24,25^.

A crucial role of RIPK1 for necroptosis induction by all aforementioned death receptors is well documented^26,27^. However, there are conflicting data concerning the relevance of RIPK1 in TNFR1-induced NFκB signaling. While in some studies RIPK1 was found to be largely dispensable for NFκB activation by TNFR1 (refs. ^28–30^), other reports observed an almost obligate role of RIPK1 in this type of TNFR1 response^22,23,31–35^. This discrepancy might reflect redundant activities of RIPK1 and TRADD but this issue has been poorly addressed so far. Consistently, however, various studies demonstrated that RIPK1 is required for NFκB signaling by CD95 and the TRAIL death receptors^22,23,36–38^.

Early on, TRADD has been considered as a crucial factor for caspase-8 activation and NFκB signaling in the context of TNFR1 signaling. TRADD interacts strongly with FADD and the TNF receptor-2 associated factor 2 (TRAF2) molecule which promotes the activation of the NFκB pathway-stimulatory inhibitor of kappaB (IκB) kinase 2 (IKK2)^39^. Moreover, ectopic expression of FADD and TRAF2 deletion mutants interfering with these interactions efficiently prevents apoptosis induction and NFκB activation by TNFR1 (ref. ^39^). Surprisingly, analysis of cells with knockout or knockdown of TRADD revealed varying effects on these TNFR1 activities reaching from no or mild inhibition^8,15^ to complete abrogation^40–43^. Again, redundancy between RIPK1 and TRADD has been discussed as a possible explanation for these unexpected findings. From studies with TRADD siRNA there is initial evidence for a necroptosis-inhibitory activity of TRADD in TNFR1 signaling^40^. Although TRADD is not part of the receptor signaling complexes of CD95 and the TRAIL death receptors, knockdown studies gave evidence for a contribution of TRADD to CD95- and TRAIL death receptor-induced NFκB signaling^44,45^. In accordance with the known anti-necroptotic effects of NFκB activation, it has been furthermore found that TRADD knockout fibroblasts are sensitized for TRAIL-induced apoptosis^44^.

Stimulation of death receptors results in the appearance of cytosolic complexes which contain one or more of the three cytosolic DD proteins TRADD, FADD, and RIPK1 but also caspase-8 and RIPK3. These cytosolic complexes have been implicated in the cell death-inducing activities of the various death receptors but may also contribute to NFκB signaling^8,21,23,46,47^. The activity of these complexes is manifold regulated by phosphorylation and various types of ubiquitination. It is, however, poorly understood how the liganded receptor signaling complexes trigger the formation of the cytosolic complexes and to which extent there are functional redundancies between the receptor signaling complexes and the secondarily formed cytosolic complexes.

In sum, experimental data regarding redundant/cooperative functions of TRADD, FADD, and RIPK1 are limited and the basis of their DR receptor-specific activities are hardly understood. We therefore generated in this study a collection of RIPK3-expressing and therefore necroptosis-competent HeLa transfectants deficient in expression of TRADD, RIPK1, and FADD or double-deficient in the expression of any combination of two of these molecules and evaluated this cell panel side by side with respect to cell death induction and NFκB signaling.

## Material and methods

### Cells and cytokines

The RIPK3-expressing HeLa transfectant HeLa-RIPK3 was a kind gift of Martin Leverkus (University Hospital Aachen) and has been described elsewhere^48^. HeLa-RIPK3 cells and the various variants derived thereof were cultured at 37 °C at 5% CO_2_ in RPMI 1640 medium supplemented with 10% fetal calf serum (FCS; Gibco). Human TNF was a kind gift of Prof. Daniela Männel (University of Regensburg) and human Killer-TRAIL (TRAIL) was from Enzo Life Sciences. ZVAD (carbobenzoxy-valyl-alanyl-aspartyl-[*O*-methyl]-fluoromethylketone), necrostatin-1, MLN4924, and cycloheximide were from Sigma. Necrostatin-1s was from Merck Millipore. If not stated otherwise, chemicals were from Sigma.

### Generation of HeLa-RIPK3 knockout variants

To obtain HeLa-RIPK3-TRADD_KO_, HeLa-RIPK3-RIPK1_KO_, HeLa-RIPK3-FADD_KO_, and HeLa-RIPK3-casp8_KO_ cells, HeLa-RIPK3 cells were transfected using polyethylenimine (PEI; Polysciences Inc., Warrington, USA) with mixtures of GeneArt CRISPR Nuclease (CD4 Reporter) vector plasmids (ThermoFisher Scientific) encoding three guide RNAs targeting the gene of interest and Cas9. The guide sequences (5′ to 3′) used were for TRADD: GGCCTGACCGATCCCAATGGCGG, GGCCGCGCTCGCCCAGCACTCGG, and GAAATCTGAAGTGCGGCTCGGGG; for RIPK1: GCTCCTGGGCGTCATCATAGAGG, GCTCTGCTGGGAAGCGAATCCGG, and GAAAAACTGTGCCCGTAAACTGG; for FADD: GCGCGTGGGCAAGCGCAAGCTGG, GCGGCGCGTCGACGACTTCGAGG, and GGGCCATGTCCCCGATGTCATGG; and for caspase-8: GCCTGGACTACATTCCGCAAAGG, GCTCTTCCGAATTAATAGACTGG, and GCCTGAGAGAGCGATGTCCTCGG. All guide RNAs are based on sequences chosen from Table 1 of the supplementary website of Mali et al.^49^ (http://arep.med.harvard.edu/human_crispr/). In brief, PEI transfection was performed as follows: A plasmid-PEI mixture was prepared by adding 36 µl of a 1 mg/ml water solution of PEI dropwise under vortexing in 2 ml of serum-free RPMI 1640 medium containing 12 µg plasmid DNA. After incubating the plasmid-PEI mixture for 15 min at room temperature, the mixture was added to a 15-cm tissue culture dish with close to confluent HeLa-RIPK3 cells which had received immediately before 15 ml fresh serum free medium. In the case of the generation of the HeLa-RIPK3-TRADD_KO_ and HeLa-RIPK3-RIPK1_KO_ cells, several 10 cm tissue culture dishes were seeded the next day with 50–500 cells to allow the growth of isolated transferable clones. After 2–3 weeks around 20 clones were expanded and analyzed by western blotting for TRADD and RIPK1 expression. Three clones lacking TRADD or RIPK1 expression were then pooled and used for further studies. Several clones showing no evidence for changed target expression were pooled and used as control cells (HeLa-RIPK3_con_). In case of the generation of the HeLa-RIPK3-FADD_KO_ and HeLa-RIPK3-casp8_KO_ cells, transfected cells were challenged the next day with 2.5 µg/ml CHX plus 100 ng/ml TRAIL (for HeLa-RIPK3-FADD_KO_ cells) or with 2.5 µg/ml CHX plus 100 ng/ml TRAIL plus necrostatin-1 (90 µM) (for HeLa-RIPK3-casp8_KO_ cells). While all cells of a control transfection with empty guide DNA plasmid died within few days, numerous colonies developed from the transfections with FADD and caspase-8 guide DNA plasmids. After app. 2 weeks these cells were harvested, checked for absence of FADD and caspase-8 expression and used for further studies. For the generation of the HeLa-RIPK3-FADD/TRADD_DKO_ and HeLa-RIPK3-RIPK1/TRADD_DKO_ cells, HeLa-RIPK3-TRADD_KO_ were transfected as described above with the GeneArt CRISPR Nuclease (CD4 Reporter) plasmid mixtures encoding the three FADD guide RNAs or the three RIPK1 guide RNAs. In the case of the transfection of the HeLa-RIPK3-TRADD_KO_ variant with the FADD-specific CRISPR/Cas9 plasmid mixture, cells were treated the next day with 2.5 µg/ml CHX plus 100 ng/ml TRAIL. In the case of transfection with the RIPK1-specific CRISPR/Cas9 plasmid mixture, the HeLa-RIPK3-TRADD_KO_ cells were treated the next day with 2.5 µg/ml CHX, 100 ng/ml TRAIL, and 20 µM ZVAD. In both cases surviving cells were pooled and checked for the additional absence of FADD and RIPK1, respectively. Similarly, HeLa-RIPK3-FADD/RIPK1_KO_ cells were obtained by transfection of HeLa-RIPK3-RIPK1_KO_ cells with the FADD-specific guide RNA mixture and selection with 2.5 µg/ml CHX plus 100 ng/ml TRAIL.

**Table 1:** Viability differences of TNF treatment groups of interest

Bonferroni’s multiple comparison test	Means (%)	Mean Diff. (%)	*P* value
**Apoptosis sensitivity in the absence of CHX** ^a^			
TRADD-KO vs TRADD-KO TNF + N	100 vs 90	10	**
**Apoptosis sensitivity in the presence of CHX** ^a^			
EV vs EV TNF + C + N	100 vs 34	66	***
CON vs CON TNF + C + N	100 vs 64	36	***
TRADD-KO vs TRADD-KO TNF + C + N	100 vs 28	72	***
RIPK1-KO vs RIPK1-KO TNF + C + N	100 vs 30	70	***
FADD-TRADD-DKO vs FADD-TRADD-DKO TNF + C + N	100 vs 68	32	***
**Necroptosis sensitivity in the absence of CHX** ^a^			
FADD-KO vs FADD-KO TNF + Z	100 vs 7	93	***
TRADD-KO vs TRADD-KO TNF + Z	100 vs 84	16	***
FADD-TRADD-DKO vs FADD-TRADD-DKO TNF + Z	100 vs 84	16	***
**Necroptosis sensitivity in the presence of CHX** ^a^			
CON vs CON TNF + C + Z	100 vs 19	81	***
FADD-KO vs FADD-KO TNF + C + Z	100 vs 23	77	***
TRADD-KO vs TRADD-KO TNF + C + Z	100 vs 22	78	***
FADD-TRADD-DKO vs FADD-TRADD-DKO TNF + C + Z	100 vs 11	89	***
Casp.8-KO vs Casp.8-KO TNF + C + Z	100 vs 63	37	***
**Effect of CHX on apoptosis sensitivity** ^a^			
EV TNF + N vs EV TNF + C + N	84 vs 34	50	***
CON TNF + N vs CON TNF + C + N	97 vs 64	33	***
TRADD-KO TNF + N vs TRADD-KO TNF + C + N	90 vs 28	62	***
RIPK1-KO TNF + N vs RIPK1-KO TNF + C + N	91 vs 30	62	***
FADD-TRADD-DKO TNF + N vs FADD-TRADD-DKO TNF + C + N	90 vs 68	22	***
**Effect of CHX on necroptosis sensitivity** ^a^			
CON TNF + Z vs CON TNF + C + Z	98 vs 19	79	***
TRADD-KO TNF + Z vs TRADD-KO TNF + C + Z	84 vs 22	62	***
FADD-TRADD-DKO TNF + Z vs FADD-TRADD-DKO TNF + C + Z	84 vs 11	74	***
Casp.8-KO TNF + Z vs Casp.8-KO TNF + C + Z	91 vs 63	28	***
**Effect of FADD and TRADD on apoptosis sensitivity** ^b,d^			
CON TNF + C + N vs FADD-KO TNF + C + N	64 vs 96	−32	***
CON TNF + C + N vs TRADD-KO TNF + C + N	64 vs 28	35	***
CON TNF + C + N vs FADD-TRADD-DKO TNF + C + N	64 vs 68	−4.7	ns
**Effect of FADD and RIPK1 on apoptosis sensitivity** ^b,d^			
CON TNF + C + N vs FADD-KO TNF + C + N	64 vs 96	−32	***
CON TNF + C + N vs RIPK1-KO TNF + C + N	64 vs 30	34	***
CON TNF + C + N vs FADD-RIPK1-DKO TNF + C + N	64 vs 90	−26	***
**Effect of TRADD and RIPK1 on apoptosis sensitivity** ^b,d^			
CON TNF + C + N vs TRADD-KO TNF + C + N	64 vs 28	35	***
CON TNF + C + N vs RIPK1-KO TNF + C + N	64 vs 30	34	***
CON TNF + C + N vs TRADD-RIPK1-DKO TNF + C + N	64 vs 90	−26	***
**Effect of FADD and TRADD on necroptosis sensitivity** ^c,d^			
CON TNF + Z vs FADD-KO TNF + Z	98 vs 7	91	***
CON TNF + Z vs TRADD-KO TNF + Z	98 vs 84	14	***
CON TNF + Z vs FADD-TRADD-DKO TNF + Z	98 vs 84	14	**
**Effect of FADD and RIPK1 on necroptosis sensitivity** ^d^			
CON TNF + Z vs FADD-KO TNF + Z	98 vs 7	91	***
CON TNF + Z vs RIPK1-KO TNF + Z	98 vs 101	−2,4	ns
CON TNF + Z vs FADD-RIPK1-DKO TNF + Z	98 vs 100	−2,0	ns
CON TNF + C + Z vs FADD-KO TNF + C + Z	19 vs 23	−3,3	ns
CON TNF + C + Z vs RIPK1-KO TNF + C + Z	19 vs 97	−78	***
CON TNF + C + Z vs FADD-RIPK1-DKO TNF + C + Z	19 vs 92	−73	***
**Effect of TRADD and RIPK1 on necroptosis sensitivity** ^d^			
CON TNF + Z vs TRADD-KO TNF + Z	98 vs 84	14	***
CON TNF + Z vs RIPK1-KO TNF + Z	98 vs 101	−2.4	ns
CON TNF + Z vs TRADD-RIPK1-DKO TNF + Z	98 vs 97	0.94	ns
CON TNF + C + Z vs TRADD-KO TNF + C + Z	19 vs 22	−3.0	ns
CON TNF + C + Z vs RIPK1-KO TNF + C + Z	19 vs 97	−78	***
CON TNF + C + Z vs TRADD-RIPK1-DKO TNF + C + Z	19 vs 97	−78	***

Viability data of with 100 ng/ml TNF-treated cells for all HeLa variants investigated and all co-treatment conditions were compiled and analyzed by ANOVA (one-way, Bonferroni comparison of all pairs of columns) using the GraphPad Prism5 software. For a complete table containing all 2628 possible comparisons see Supplementary Table II

HeLa-RIPK3 = CON; HeLa-RIPK3-FADD_KO_ = FADD-KO; HeLa-RIPK3-TRADD_KO_ = TRADD-KO; HeLa-RIPK3-RIPK1_KO_ = RIPK1-KO; HeLa-RIPK3-Casp8_KO_ = Casp.8-KO; HeLa-RIPK3-FADD/TRADD_DKO_ = FADD-TRADD-DKO; HeLa-RIPK3-FADD/RIPK1_DKO_ = FADD-RIPK1-DKO; HeLa-RIPK3-TRADD/RIPK1_DKO_ = TRADD-RIPK1-DKO; HeLa-EV = EV; Z = ZVAD; N = necrostatin-1; C = CHX; vs = versus; ns = non-specific. ***p < 0.001; **p < 0.01; *p < 0.05

^a^Only comparisons showing significant differences are listed in this section of the table

^b^HeLa-RIPK3-TRADD_KO_ cells are the only cell variant showing a significant apoptotic effect (10%) in the absence of CHX. In this section are therefore only comparisons of apoptosis induction in CHX-treated cells

^c^In the presence of CHX, FADD and TRADD had no significant effect on necroptosis

^d^Please note, in these sections some rows were repeatedly shown to facilitate comparison of the effects of mono- and double deficiency in each section

### Viability assay

Cells were seeded in 96-well tissue culture plates in 100 µl medium/FCS (20 × 10^3^ cells per well). The following day, medium was replaced by medium containing the indicated mixtures of CHX, ZVAD, and nec-1 and finally after 30 min TNF and TRAIL were added. Cells were stimulated in technical triplicates. For later normalization, one triplicate was challenged with a cytotoxic mixture of 2.5 µg/ml CHX, 400 ng/ml Fc-CD95L, 400 ng/ml TRAIL, 400 ng/ml TNF, 100 ng/ml TWEAK, and 0.02% sodium azide in medium/FCS yielding in complete cell killing. The next day, supernatants and detached cells were removed and remaining viable cells were quantified by crystal violet staining. Viability values were finally obtained by normalization using the values of untreated cells (=100 %) and cells treated with the cytotoxic mixture (0%). All viability data obtained in course of this study for treatments with 100 ng/ml TNF or 100 ng/ml TRAIL were compiled in a single table for each of the two ligands and analyzed by ANOVA (one-way, Bonferroni comparison of all pairs of columns) using the GraphPad Prism5 software. All viability data are listed in Supplementary Table I. The significance results of the Bonferroni comparison for TNF-treated cells are listed in Supplementary Table II, and for TRAIL-treated cells in Supplementary Table III.

### IL8 ELISA

Cells were seeded in 96-well tissue culture plates in 100 µl medium with 10% FCS at a density of 20 × 10^3^ cells per well. Next day, medium was replaced by medium/FCS supplemented with the inhibitors combinations of interest. After 30 min TNF and TRAIL were added. Cells were stimulated in technical triplicates and the next day the cell were analyzed for their IL8 content using the OptEIA IL8 ELISA Kit according to the recommendations of the supplier (BD Biosciences). Results of three or more independent experiments were analyzed by ANOVA (one-way, Bonferroni comparison of selected pairs of columns).

### Immunoprecipitation

Ligand-induced signaling complexes of TNFR1 and the TRAIL receptors were essentially immunoprecipitated as described elsewhere for TNFR1 (ref. ^50^). In brief, per stimulation group cells were seeded to two 15 cm tissue culture dishes and grown near confluency. Cells were then stimulated with 500 ng/ml of Fc-TNF(32 W/86T), an Fc fusion protein of the TNFR1-specific TNF mutant TNF(32W/86T)^50^, or 2 µg/ml of Fc-Flag-TRAIL (Supplementary Data Fig. S1). As a control, one group remained untreated. After the indicated times all plates were washed three times with icecold phosphate-buffered saline (PBS) to remove unbound Fc fusion proteins. Cells were scratched with a rubber policeman into 10 ml icecold PBS, pelleted by centrifugation, and were resuspended in 1.5 ml lysis buffer (30 mM Tris HCl pH 7.5, 120 mM NaCl, 1% Triton X-100, 10% glycerol) supplemented with the cOmplete^TM^ protease inhibitor protease cocktail (1 tablet per 25 ml lysis buffer; Sigma-Aldrich). Samples were incubated 20 min on ice and insoluble debris were removed by centrifugation twice at 14000*g* for 20 min. The lysates of the untreated control cells were supplemented with 5 ng Fc-TNF(32W/86T) or 10 ng Fc-Flag-TRAIL. Lysates were supplemented with 40 µl protein G agarose and incubated overnight at 4 °C under gentle agitation. Protein G beads were recovered and washed four times in lysis buffer by centrifugation for 30 s at 500*g*. Protein G agarose was incubated for 15 min at 75 °C in Lämmli sample buffer and protein G agarose was removed by centrifugation. Cleared supernatants were subjected to western blotting (see below).

### Western blot analysis

Cells were challenged with the reagents of interest, scratched into the supernatant, and recovered by centrifugation. After washing twice in icecold PBS, cell pellets were dissolved in Lämmli buffer. Total cell lysates (or samples derived from immunoprecipitation experiments) were cleared by centrifugation and subjected to SDS-PAGE. After transfer of proteins to 0.2 µm nitrocellulose blotting membrane (GE Healthcare Life Sciences), the membranes were blocked with 5% non-fat dried milk powder in PBS with 0.05% (v/v) Tween-20 (PBST) or in 20 mM Tris HCl, pH 7.6 with 0.05% (v/v) Tween-20 (TBST). After three washes for 5 min with PBST or TBST, membranes were incubated overnight at 4 °C with the primary antibody of interest (0.5–2 µg/ml) in PBST or TBST supplemented with sodium azide (0.02%). Membranes were washed again three times 5 min and were then incubated at room temperature for 1–3 h with HRP-labeled secondary antibodies from Cell Signaling (anti-rabbbit (#7074)) or Dako (anti-goat (#P0449), anti-mouse (#P0260) and anti-rabbit (#P0448)) in PBST or TBST. In the case of the anti-rabbit secondary antibody from Cell Signaling and the anti-goat antibody from Dako PBST/TBST have been supplemented with 2.5% (w/v) non-fat dried milk powder. After washing (3 × 10 min PBST or TBST) antigen–antibody complexes on the membranes were visualized using the ECL^™^ Prime Western Blotting System (Sigma).

Following primary antibodies have been used: anti-caspase-8 (Santa Cruz, E-20, sc-6133), anti-caspase-8 (Enzo, 5F7), anti-caspase-3 (Cell Signaling, 8G10), anti-caspase-9 (Cell Signaling, # 9502), anti-PARP (BD Biosciences), anti-CYLD (Cell Signaling, D1A10), anti-DR4/TRAILR1 (Cell Signaling, D9S1R), anti-DR5/TRAILR2 (Cell Signaling, D4E9), anti-phospho-RIPK1 (Ser166) (Cell signaling, D1L3S), which is specific for serine 166 phosphorylated necroptosis-competent RIPK1, anti-RIPK1 (Cell Signaling, D94C12), anti-RIPK1 (BD Biosciences, #610459), anti-tubulin (ThermoFisher Scientific (DM1A), anti-TNFR1 (Cell Signaling, C25C1), anti-TRADD (Cell Signaling, 7G8), anti-A20 (Cell Signaling, D13H3), anti-IKKß (Cell Signaling, D30C6), anti-FADD (Cell Signaling, #2782), anti-Sharpin (Abcam ab125188), anti-TRAF2 (Santa Cruz, C-20, sc-876), anti-FLIP (Biomol, NF6, AG-20B-0056), anti-phospho-IκBα (Ser32) (Cell Signaling, 14D4), which recognizes serine 32 phosphorylated IκBα prone for ubiquitination and proteasomal degradation, and anti-IκBα (Cell Signaling, L35A5).

## Results

### Relevance of DD adapter proteins for TNF- and TRAIL-induced cell death

**Fig. 1:**
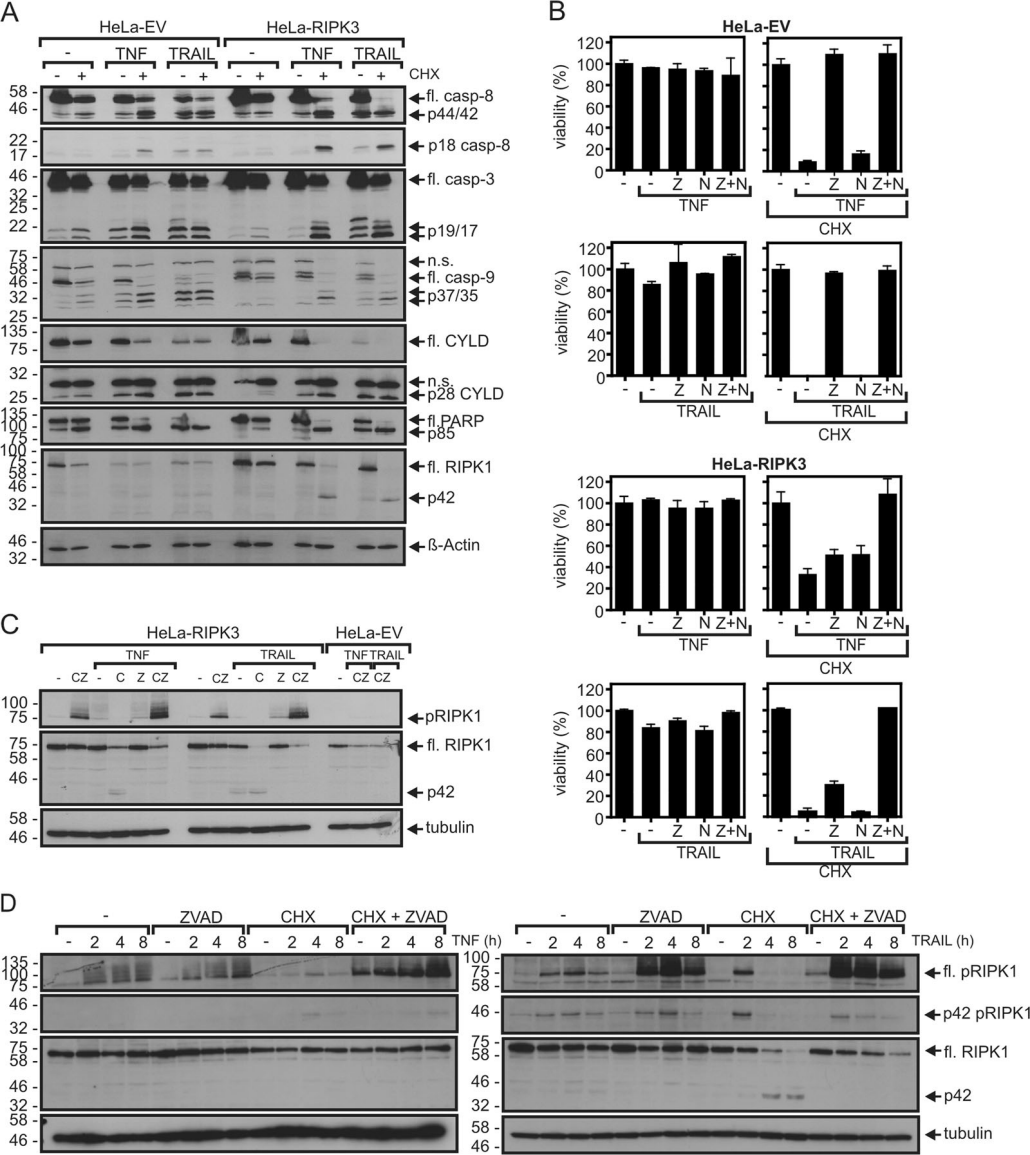
TNF and TRAIL induce apoptosis and necroptosis in HeLa-RIPK3 transfectants. **a** HeLa-EV and HeLa-RIPK3 cells were stimulated overnight with 100 ng/ml TNF or 100 ng/ml TRAIL in the presence and absence of CHX (2.5 µg/ml) and total cell lysates were analyzed by western blot for processing of the indicated caspases and caspase substrates. fl full-length. **b** Cells were challenged overnight in technical triplicates with the indicated mixtures of TNF (100 ng/ml), TRAIL (100 ng/ml), CHX (2.5 µg/ml), ZVAD (Z, 20 µM), and nec1 (N, 90 µM). Cellular viability was evaluated by crystal violet staining. A representative panel of experiments is shown. For statistical analysis of independent experiments please see Tables 1 and 2 and Supplementary Tables I–III. **c** HeLa-EV and Hela-RIPK3 cells were treated with the indicated mixtures of 100 ng/ml TNF, 100 ng/ml TRAIL, 2.5 µg/ml CHX (C), and 20 µM ZVAD (Z) for 8 h and RIPK1 phosphorylation was analyzed by western blot. **d** Hela-RIPK3 CRISPR/Cas9 control cells (HeLa-RIPK3_con_ cells, see also Fig. 2a) were treated with the indicated mixtures of TNF (100 ng/ml), TRAIL (100 ng/ml), CHX (2.5 µg/ml), and ZVAD (20 µM) for 0–8 h. Total cell lysates were analyzed for RIPK1 phosphorylation by western blotting

In our studies, we used HeLa transfectants stably expressing RIPK3 (ref. ^48^). The empty vector control HeLa transfectant (HeLa-EV) and the HeLa-RIPK3 transfectant showed only a significant cell death response with TNF in the presence of the protein synthesis inhibitor cycloheximide (CHX) (Fig. 1a, b; Table 1, Supplementary Table II). TRAIL alone induced significant caspase activation and moderate cell death but again robust cell death induction occurred in the presence of CHX (Fig. 1a, b; Table 2, Supplementary Table III). Generally, it appeared that TNF and TRAIL-induced processing of caspase-8 and caspase-8 substrates such as CYLD and RIPK1 (refs. ^51–53^) is enhanced in the CHX-sensitized HeLa-RIPK3 cells (Fig. 1a). While cell death induction by TNF and TRAIL was fully blocked by the caspase inhibitor ZVAD in CHX-sensitized HeLa-EV cells, HeLa-RIPK3 transfectants were only rescued by combined application of ZVAD and the necroptosis inhibitor necrostatin-1 (nec1) which act by inhibition of RIPK1 kinase activity (Fig. 1b; Tables 1 and 2, Supplementary Tables I and III). Moreover, phosphorylation of RIPK1, a hallmark of DR-induced necroptosis, was much more evident in TNF- and TRAIL-treated HeLa-RIPK3 cells than in HeLa-EV cells (Fig. 1c). Noteworthy, in HeLa-RIPK3 cells TNF-induced RIPK1 phosphorylation was already robustly detectable in the absence of ZVAD, while this compound significantly enhanced TRAIL-induced RIK1P phosphorylation (Fig. 1d). In accordance with the necroptosis-inhibitory activity of caspase-8, CHX, which facilitates caspase-8 activation and apoptosis induction, antagonized TNF- and TRAIL-induced RIPK1 phosphorylation (Fig. 1d). These data confirmed the expected apoptosis and necroptosis competence of HeLa-RIPK3 cells.

**Table 2:** Viability differences of TRAIL treatment groups of interest

Bonferroni’s multiple comparison test	Means (%)	Mean diff. (%)	*P* value
**Apoptosis sensitivity in the absence of CHX** ^a^			
CON vs CON TRAIL + N	100 vs 65	35	***
TRADD-KO vs TRADD-KO TRAIL + N	100 vs 79	21	***
RIPK1-KO vs RIPK1-KO TRAIL + N	100 vs 77	23	***
FADD-TRADD-DKO vs FADD-TRADD-DKO TRAIL + N	100 vs 77	23	***
TRADD-RIPK1-DKO vs TRADD-RIPK1-DKO TRAIL + N	100 vs 68	32	***
**Apoptosis sensitivity in the presence of CHX** ^a^			
EV vs EV TRAIL + C + N	100 vs 18	82	***
CON vs CON TRAIL + C + N	100 vs 6	94	***
TRADD-KO vs TRADD-KO TRAIL + C + N	100 vs 15	85	***
RIPK1-KO vs RIPK1-KO TRAIL + C + N	100 vs 11	89	***
FADD-TRADD-DKO vs FADD-TRADD-DKO TRAIL + C + N	100 vs 75	25	***
TRADD-RIPK1-DKO vs TRADD-RIPK1-DKO TRAIL + C + N	100 vs 12	88	***
**Necroptosis sensitivity in the absence of CHX** ^a^			
CON vs CON TRAIL + Z	100 vs 79	21	***
TRADD-KO vs TRADD-KO TRAIL + Z	100 vs 83	17	**
FADD-TRADD-DKO vs FADD-TRADD-DKO TRAIL + Z	100 vs 76	24	***
Casp.8-KO vs Casp.8-KO TRAIL + Z	100 vs 77	23	***
**Necroptosis sensitivity in the presence of CHX** ^a^			
CON vs CON TRAIL + C + Z	100 vs 28	72	***
TRADD-KO vs TRADD-KO TRAIL + C + Z	100 vs 11	89	***
Casp.8-KO vs Casp.8-KO TRAIL + C + Z	100 vs 62	38	***
**Effect of CHX on apoptosis sensitivity** ^a^			
EV TRAIL + N vs EV TRAIL + C + N	81 vs 18	63	***
CON TRAIL + N vs CON TRAIL + C + N	65 vs 6	59	***
TRADD-KO TRAIL + N vs TRADD-KO TRAIL + C + N	79 vs 15	64	***
RIPK1-KO TRAIL + N vs RIPK1-KO TRAIL + C + N	77 vs 11	66	***
TRADD-RIPK1-DKO TRAIL + N vs TRADD-RIPK1-DKO TRAIL + C + N	68 vs 12	56	***
**Effect of CHX on necroptosis sensitivity** ^a^			
CON TRAIL + Z vs CON TRAIL + C + Z	79 vs 28	51	***
TRADD-KO TRAIL + Z vs TRADD-KO TRAIL + C + Z	83 vs 11	72	***
**Effect of FADD and TRADD on apoptosis sensitivity** ^b^			
CON TRAIL + N vs FADD-KO TRAIL + N	65 vs 95	−30	***
CON TRAIL + N vs TRADD-KO TRAIL + N	65 vs 79	−14	ns
CON TRAIL + N vs FADD-TRADD-DKO TRAIL + N	65 vs 77	−12	ns
CON TRAIL + C + N vs FADD-KO TRAIL + C + N	6 vs 89	−83	***
CON TRAIL + C + N vs TRADD-KO TRAIL + C + N	6 vs 15	−8.8	ns
CON TRAIL + C + N vs FADD-TRADD-DKO TRAIL + C + N	6 vs 75	−69	***
**Effect of FADD and RIPK1 on apoptosis sensitivity** ^b^			
CON TRAIL + N vs FADD-KO TRAIL + N	65 vs 95	−30	***
CON TRAIL + N vs RIPK1-KO TRAIL + N	65 vs 77	−12	ns
CON TRAIL + N vs FADD-RIPK1-DKO TRAIL + N	65 vs 92	−27	***
CON TRAIL + C + N vs FADD-KO TRAIL + C + N	6 vs 89	−83	***
CON TRAIL + C + N vs RIPK1-KO TRAIL + C + N	6 vs 11	−5.0	ns
CON TRAIL + C + N vs FADD-RIPK1-DKO TRAIL + C + N	6 vs 87	−81	***
Effect of TRADD and RIPK1 on apoptosis sensitivity			
No significant effect in all six comparisons			
**Effect of FADD and TRADD on necroptosis sensitivity** ^b^			
CON TRAIL + Z vs FADD-KO TRAIL + Z	79 vs 92	−13	**
CON TRAIL + Z vs TRADD-KO TRAIL + Z	79 vs 83	−4.6	ns
CON TRAIL + Z vs FADD-TRADD-DKO TRAIL + Z	79 vs 76	2.4	ns
CON TRAIL + C + Z vs FADD-KO TRAIL + C + Z	28 vs 89	−61	***
CON TRAIL + C + Z vs TRADD-KO TRAIL + C + Z	28 vs 11	17	ns
CON TRAIL + C + Z vs FADD-TRADD-DKO TRAIL + C + Z	28 vs 95	−67	***
**Effect of FADD and RIPK1 on necroptosis sensitivity** ^b^			
CON TRAIL + Z vs FADD-KO TRAIL + Z	79 vs 92	−13	**
CON TRAIL + Z vs RIPK1-KO TRAIL + Z	79 vs 96	−18	***
CON TRAIL + Z vs FADD-RIPK1-DKO TRAIL + Z	79 vs 98	−19	***
CON TRAIL + C + Z vs FADD-KO TRAIL + C + Z	28 vs 89	−61	***
CON TRAIL + C + Z vs RIPK1-KO TRAIL + C + Z	28 vs 95	−67	***
CON TRAIL + C + Z vs FADD-RIPK1-DKO TRAIL + C + Z	28 vs 88	−60	***
**Effect of TRADD and RIPK1 on necroptosis sensitivity** ^b^			
CON TRAIL + Z vs TRADD-KO TRAIL + Z	79 vs 83	−4.6	ns
CON TRAIL + Z vs RIPK1-KO TRAIL + Z	79 vs 96	−18	***
CON TRAIL + Z vs TRADD-RIPK1-DKO TRAIL + Z	79 vs 92	−13	ns
CON TRAIL + C + Z vs TRADD-KO TRAIL + C + Z	28 vs 11	17	ns
CON TRAIL + C + Z vs RIPK1-KO TRAIL + C + Z	28 vs 95	−67	***
CON TRAIL + C + Z vs TRADD-RIPK1-DKO TRAIL + C + Z	28 vs 98	−70	***

Viability data of with 100 ng/ml TRAIL-treated cells for all HeLa variants investigated and all co-treatment conditions were compiled and analyzed by ANOVA (one-way, Bonferroni comparison of all pairs of columns) using the GraphPad Prism5 software. For a complete table containing all 2628 possible comparisons see Supplementary Table III

HeLa-RIPK3 = CON; HeLa-RIPK3-FADD_KO_ = FADD-KO; HeLa-RIPK3-TRADD_KO_ = TRADD-KO; HeLa-RIPK3-RIPK1_KO_ = RIPK1-KO; HeLa-RIPK3-Casp8_KO_ = Casp.8-KO; HeLa-RIPK3-FADD/TRADD_DKO_ = FADD-TRADD-DKO; HeLa-RIPK3-FADD/RIPK1_DKO_ = FADD-RIPK1-DKO; HeLa-RIPK3-TRADD/RIPK1_DKO_ = TRADD-RIPK1-DKO; HeLa-EV = EV; Z = ZVAD; N = necrostatin-1; C = CHX; vs = versus; ns = non specific ***p < 0.001; **p < 0.01; *p < 0.05

^a^Only comparisons showing significant differences are listed in this section of the table

^b^Please note, in these sections some rows were repeatedly shown to facilitate comparison of the effects of mono- and double deficiency in each section.

**Fig. 2:**
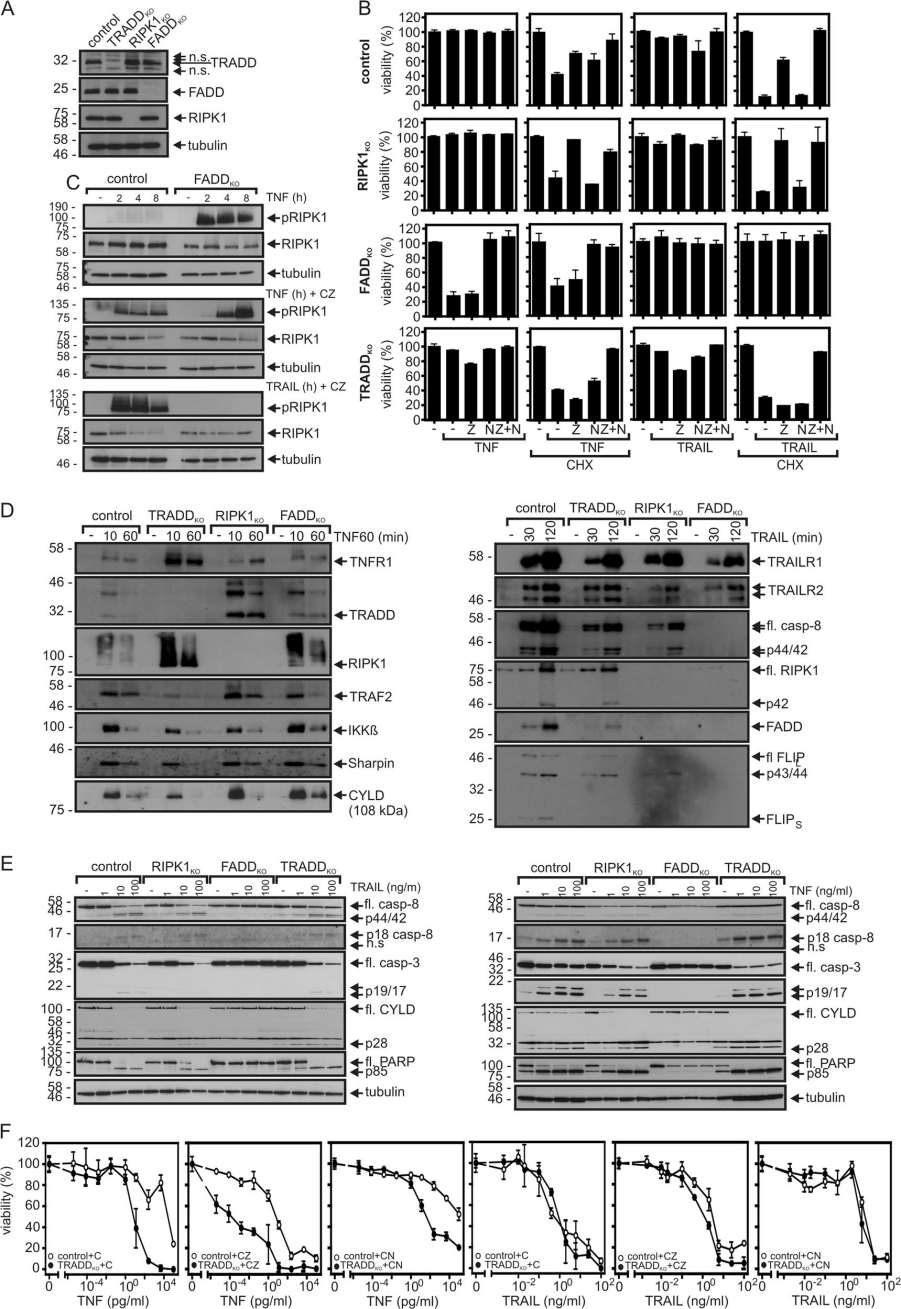
Relevance of TRADD, RIPK1, and FADD for caspase activation and cell death induction by TNF and TRAIL. **a** Western blot evaluation of TRADD, RIPK1, and FADD expression of HeLa-RIPK3_con_, HeLa-RIPK3-TRADD_KO_, HeLa-RIPK3-RIPK1_KO_, and HeLa-RIPK3-FADD_KO_ cells. fl full-length. **b** The various HeLa-RIPK3 variants were stimulated in technical triplicates as indicated with TNF (100 ng/ml), TRAIL (100 ng/ml), CHX (2.5 µg/ml), ZVAD (Z, 20 µM), and nec1 (N, 90 µM). The next day, cellular viability was evaluated by crystal violet staining. A representative panel of experiments is shown. For statistical analysis of independent experiments please see Tables 1 and 2 and Supplementary Tables I–III. **c** Western blot analysis of phosphorylated RIPK1 in HeLa-RIPK3 and HeLa-RIPK3-FADD_KO_ cells treated for 2, 4, or 8 h with 100 ng/ml of TNF or TRAIL. Where indicated cells were challenged in the presence of CHX (2.5 µg/ml) and ZVAD (20 µM). **d** TNFR1- and TRAIL death receptor-associated signaling complexes were immunoprecipitated from the various HeLa-RIPK3 variants with a TNFR1-specific Fc fusion protein of TNF or Fc-TRAIL and protein G beads. IPs were analyzed by western blotting for the presence of the indicated proteins. For western blot analysis of lysates see Supplementary Data (Fig. S5A). **e** HeLa-RIPK3 variants were stimulated overnight in the presence of 2.5 µg/ml CHX with 1, 10, or 100 ng/ml of TNF or TRAIL. Total cell lysates were analyzed by western blot. **f** HeLa-RIPK3_con_ and HeLa-RIPK3-TRADD_KO_ cells were challenged in technical triplicates with increasing concentrations of TNF or TRAIL in the presence of the indicated mixtures of CHX (C, 2.5 µg/ml), nec1 (N, 90 µM, apoptotic conditions), and ZVAD (Z, 20 µM, necroptotic conditions). Cellular viability was determined the next day by crystal violet staining

Next, we generated by means of the CRISPR/Cas9 technology variants of the HeLa-RIPK3 cells deficient in the expression of TRADD, RIPK1, and FADD (Fig. 2a). CRISPR/Cas9 processed HeLa-RIPK3 clones with no evidence for changed target expression were pooled and used as HeLa-RIPK3 control cells (HeLa-RIPK3_con_). The RIPK1-deficient HeLa-RIPK3 cells were completely rescued from TNF/TRAIL-induced cell death by ZVAD while nec-1 as well as its more RIPK1-specific variant nec-1s showed no effect despite being fully protective in combination with ZVAD in HeLa-RIPK3 and HeLa-RIPK3-TRADD_KO_ cells (Fig. 2b, Supplementary Data Fig. S2A, Tables 1 and 2, Supplementary Tables II and III). This confirmed that RIPK1 is essential for TNF- and TRAIL-induced necroptosis in the HeLa-RIPK3 model. While FADD deficiency rescued HeLa-RIPK3 cells from TRAIL-induced necroptosis, it sensitized for TNF-induced phosphorylation of RIPK1 and necroptosis even in the absence of ZVAD (Fig. 2b, c; Tables 1 and 2, Supplementary Tables II and III). TNF- and TRAIL-induced RIPK1 phosphorylation were furthermore similarly inhibited by nec-1 and nec-1s (Supplementary Data Fig. S2B). The differential relevance of FADD for TNF- and TRAIL-induced necroptosis correlated furthermore with the fact that ligand-induced RIPK1 recruitment into the receptor signaling complex was abrogated in FADD-deficient cells in the case of TRAIL but not in the case of TNF (Fig. 2d). In contrast, both TNF- and TRAIL-induced activation of caspases and apoptosis were completely blocked in the FADD-deficient HeLa-RIPK3 cells (Fig. 2b–e). TRADD deficiency sensitized for both TNF-induced necroptosis and TNF-induced apoptosis in the presence of CHX (Fig. 2f, Table 1). In contrast, TRADD deficiency had neither an effect on TRAIL-induced caspase-8 activation and apoptosis (Fig. 2f, Supplementary Data Fig. S3, Table 1) nor an effect on TRAIL-induced necroptosis (Fig. 2f, Table 1). TRADD and RIPK1 deficiency showed also no major effect on TNF- and TRAIL-induced caspase activation (Fig. 2e).

### The TRADD-RIPK1 dyad is obligate for death receptor-induced NFκB signaling but is only required for TNFR1-induced apoptosis

**Fig. 3:**
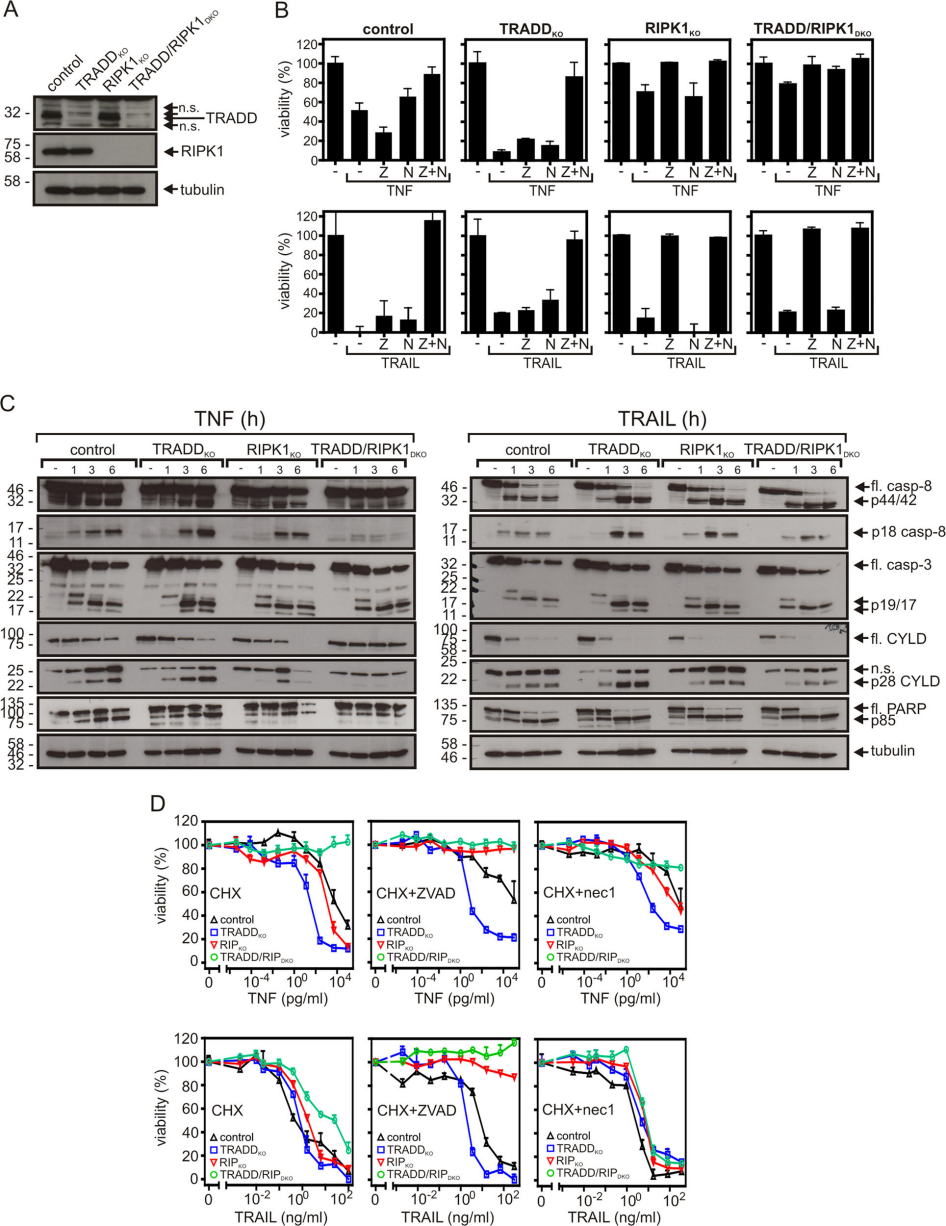
TRADD and RIPK1 act redundantly in apoptotic TNFR1 signaling. **a** HeLa-RIPK3_con_, HeLa-RIPK3-TRADD_KO_, HeLa-RIPK3-RIPK1_KO_, and HeLa-RIPK3-TRADD/RIPK1_DKO_ cells were evaluated for expression of TRADD, RIPK1, and FADD by western blot. **b** CHX-sensitized (2.5 µg/ml) cells were challenged overnight in technical triplicates as indicated with TNF (100 ng/ml), TRAIL (100 ng/ml), ZVAD (Z, 20 µM), and nec1 (N, 90 µM) and cellular viability was determined by crystal violet staining. A representative panel of experiments is shown. For statistical analysis of independent experiments please see Tables 1 and 2 and Supplementary Tables I–III. **c** HeLa-RIPK3 variants were stimulated for the indicated times with 100 ng/ml TNF or 100 ng/ml TRAIL in the presence of 2.5 µg/ml CHX. Total cell lysates were analyzed by western blotting. **d** The indicated HeLa-RIPK3 variants were stimulated in triplicates with increasing concentrations of TNF or TRAIL in the presence of the indicated combinations of CHX (2.5 µg/ml), ZVAD (Z, 20 µM), and nec1 (N, 90 µM). Next day, cell viability was evaluated by crystal violet

Since the HeLa-RIPK3-TRADD_KO_ cells were not protected and even somewhat sensitized for TNF-induced apoptosis, we looked for a possible redundant apoptotic activity of TRADD and RIPK1. For this, we secondarily deleted TRADD expression in the HeLa-RIPK3-RIPK1_KO_ variant (Fig. 3a). The resulting TRADD-RIPK1 double-deficient Hela-RIPK3 variant was highly resistant against TNF-induced caspase activation and apoptosis (Fig. 3b, c; Table 1, Supplementary Table II). Apoptotic TRAIL signaling, however, remained largely unaffected (Fig. 3b, c; Supplementary Table III). In accordance with the fact that TNF- and TRAIL-induced cell killing was completely abrogated by ZVAD in the RIPK1-KO and TRADD/RIPK1 DKO variants (Fig. 3b, Tables 1 and 2, Supplementary Tables II and III), there was no phosphorylation of MLKL in these cells (Supplementary Data Fig. S4). Thus, TRADD and RIPK1 redundantly mediate caspase activation and apoptosis in HeLa-RIPK3 cells in TNFR1 but not TRAIL death receptor signaling. FADD, furthermore, has a pivotal role in TNF- and TRAIL-induced apoptosis (Fig. 2b–e) but only in the case of TRAIL it is also sufficient to induce apoptosis (Fig. 3b, c).

**Fig. 4:**
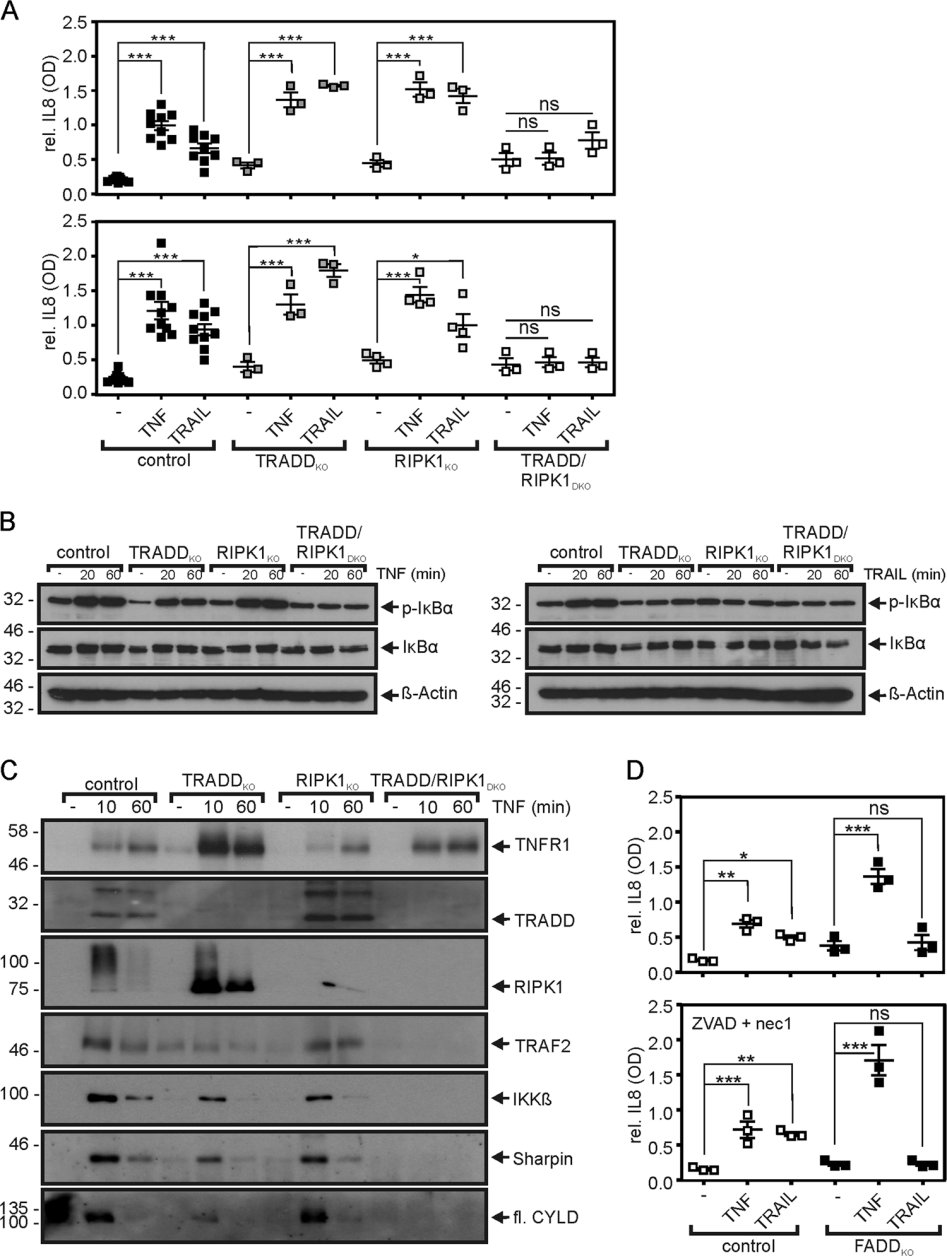
TRADD and RIPK1 act redundantly in proinflammatory death receptor signaling. **a** Cells were challenged overnight in triplicates with TNF (100 ng/ml) or TRAIL (100 ng/ml) in the absence (upper panel) and presence (lower panel) of a mixture of 20 µM ZVAD and 90 µM necrostatin-1. Next day, supernatants were analyzed for the presence of IL8 by ELISA. Treatment with ZVAD/necrostatin-1 served to prevent effects of cell death on IL8 production (e.g. due to apoptosis-associated activation of caspases). Shown are the results from independent experiments. **b** Cells were stimulated in the presence of 2.5 µg/ml CHX and 10 µM MLN4924 with 100 ng/ml TNF or 100 ng/ml TRAIL and were analyzed by western blot for expression and phosphorylation of IκBα. MLN4924 has been added to prevent proteasomal degradation of IκBα to avoid underestimation of IκBα phosphorylation. MLN4924 is an inhibitor of the NEDD8-activating enzyme which is required for the functionality of the E3 ligase complex responsible for K48 ubiquitination of IκBα. **c** TNFR1-associated signaling complexes were immunoprecipitated with a TNFR1-specific Fc-TNF mutant fusion protein and protein G beads. IPs were analyzed by western blot for the presence of the indicated proteins. fl full-length. For western blot analysis of lysates see supplementary Data Fig. S5B. **d** HeLa-RIPK3 and HeLa-RIPK3-FADD_KO_ cells were stimulated with 100 ng/ml TNF or 100 ng/ml TRAIL overnight. Cells in the lower panel were treated in the presence of 20 µM ZVAD and 90 µM necrostatin-1. Cell supernatants were analyzed for IL8 production. Shown are the results of three independent experiments. ns non-specific; ***p < 0.001; **p < 0.01; *p < 0.05

Surprisingly, the response pattern was quite different when TNF- and TRAIL-induced proinflammatory signaling was considered. Again, there was a redundant role of TRADD and RIPK1 in TNFR1 signaling. Thus, TNFR1 recruitment of the IKK complex, phosphorylation of IκBα, and upregulation of the NFκB-regulated cytokine IL8 were only modestly affected in the HeLa-RIPK3-RIPK1_KO_ and HeLa-RIPK3-TRADD_KO_ cells but were blunted in the HeLa-RIPK3-TRADD/RIPK1_DKO_ cells (Fig. 4a–c). In this case, however, RIPK1 and TRADD fulfilled, at least partially, a redundant role in TRAIL death receptor signaling, too. There was significant IL8 production in HeLa-RIPK3-RIPK1_KO_ and HeLa-RIPK3-TRADD_KO_ cells challenged with TRAIL while this effect was blocked in the double-deficient HeLa-RIPK3 variant (Fig. 4a). There was also significant phosphorylation of IκBα in TRAIL-treated HeLa-RIPK3 cells while there was no or only poor IκBα phosphorylation in the single- and double-knockout variants. In earlier work, we reported that activation of the classical NFκB pathway by CD95 is full dependent from FADD^22^. In accordance with this finding, TRAIL-induced IL8 production was abrogated in HeLa-RIPK3-FADD_KO_ cells (Fig. 4d).

### TRADD inhibits FADD-independent TNFR1-induced apoptosis

**Fig. 5:**
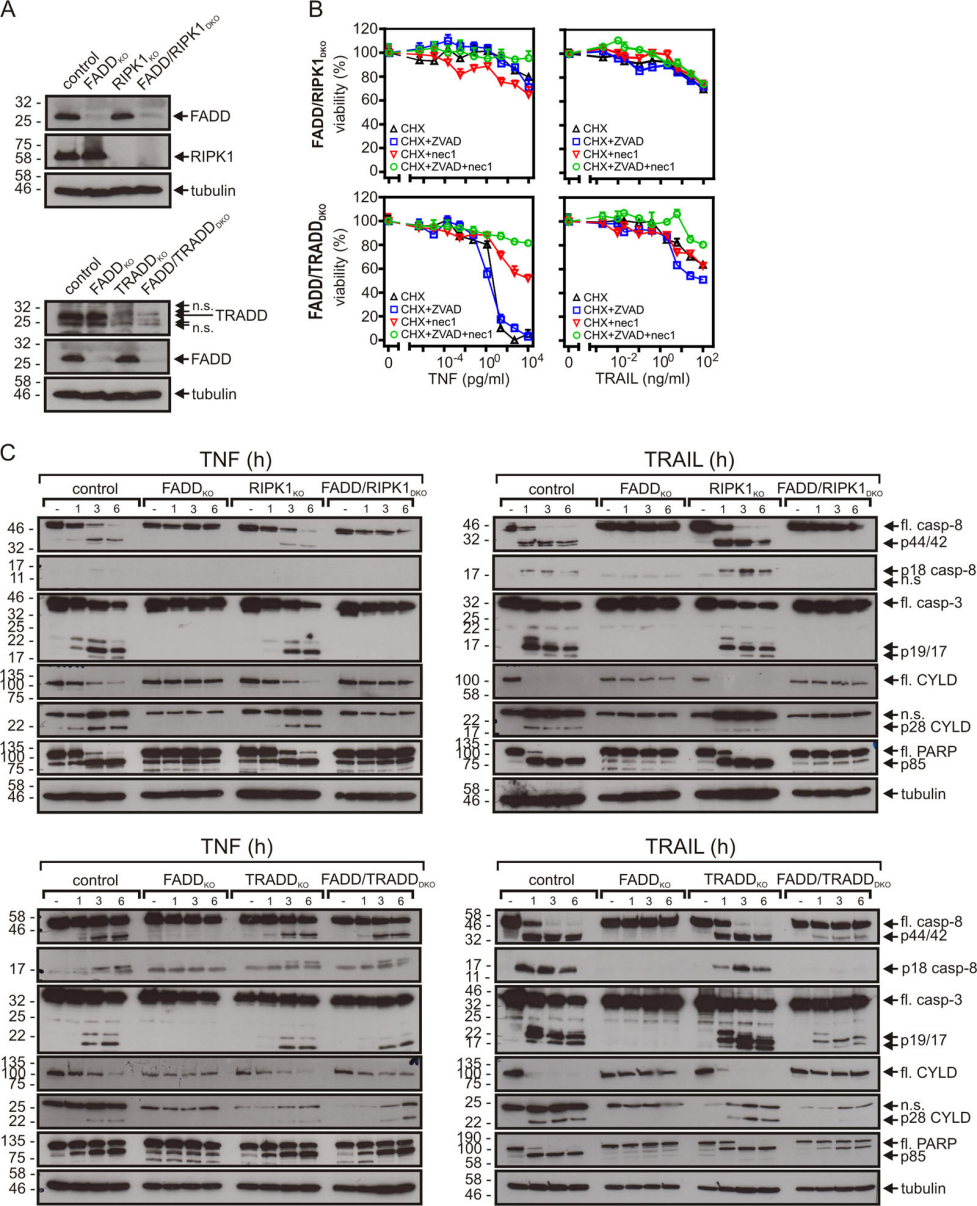
Cytotoxic TNF and TRAIL signaling in FADD-RIPK1 and FADD-TRADD double-deficient HeLa-RIPK3 cells. **a** Western blot evaluation of TRADD, RIPK1, and FADD expression of HeLa-RIPK3_con_, HeLa-RIPK3-FADD/RIPK1_DKO_ and HeLa-RIPK3-FADD/TRADD_DKO_ cells. **b** Cells were sensitized with 2.5 µg/ml CHX and were stimulated overnight in technical triplicates with the indicated combinations of TNF, TRAIL, ZVAD (20 µM), and nec1 (90 µM). Cellular viability was finally determined by crystal violet staining. A representative panel of experiments is shown. For statistical analysis of independent experiments please see Tables 1 and 2 and Supplementary Tables I–III. **c** Cells were sensitized with 2.5 µg/ml CHX and stimulated with 100 ng/ml TNF or 100 ng/ml TRAIL for 0–6 h. Total cell lysates were analyzed by western blot for processing of the indicated caspases and caspase substrates. fl full-length

In light of the strong redundancy of TRADD and RIPK1 in TNFR1 and TRAIL death receptor signaling, we also generated FADD-TRADD and FADD-RIPK1 double-deficient variants of the HeLa-RIPK3 cells to evaluate them for evidence of redundant activities of FADD and TRADD or RIPK1 in death receptor signaling (Fig. 5a). In good accordance with the crucial role of RIPK1 in TNF-induced necroptosis and of FADD in TNF-induced apoptosis, the FADD-RIPK1 double-deficient cells turned out to be highly resistant against the cytotoxic effects of TNF (Fig. 5b, c; Supplementary Table II). Not unexpected, the HeLa-RIPK3-FADD/RIPK1_DKO_ cells were also highly resistant against TRAIL (Fig. 5b, c; Supplementary Table III). CHX-sensitized HeLa-RIPK3-FADD/TRADD_DKO_ cells were still significantly killed by TNF in the presence of ZVAD. Thus, RIPK1 is not only required but also sufficient for necroptosis induction by TNF (Fig. 5b, Table 1). Surprisingly, however, although the individual knock out of FADD protects from TNF-induced apoptosis (Fig. 2b–e), the additional knock out of TRADD resensitized the FADD-deficient cell variant for TNF-induced caspase activation and apoptosis (Fig. 5b, c; Table 1).

**Fig. 6:**
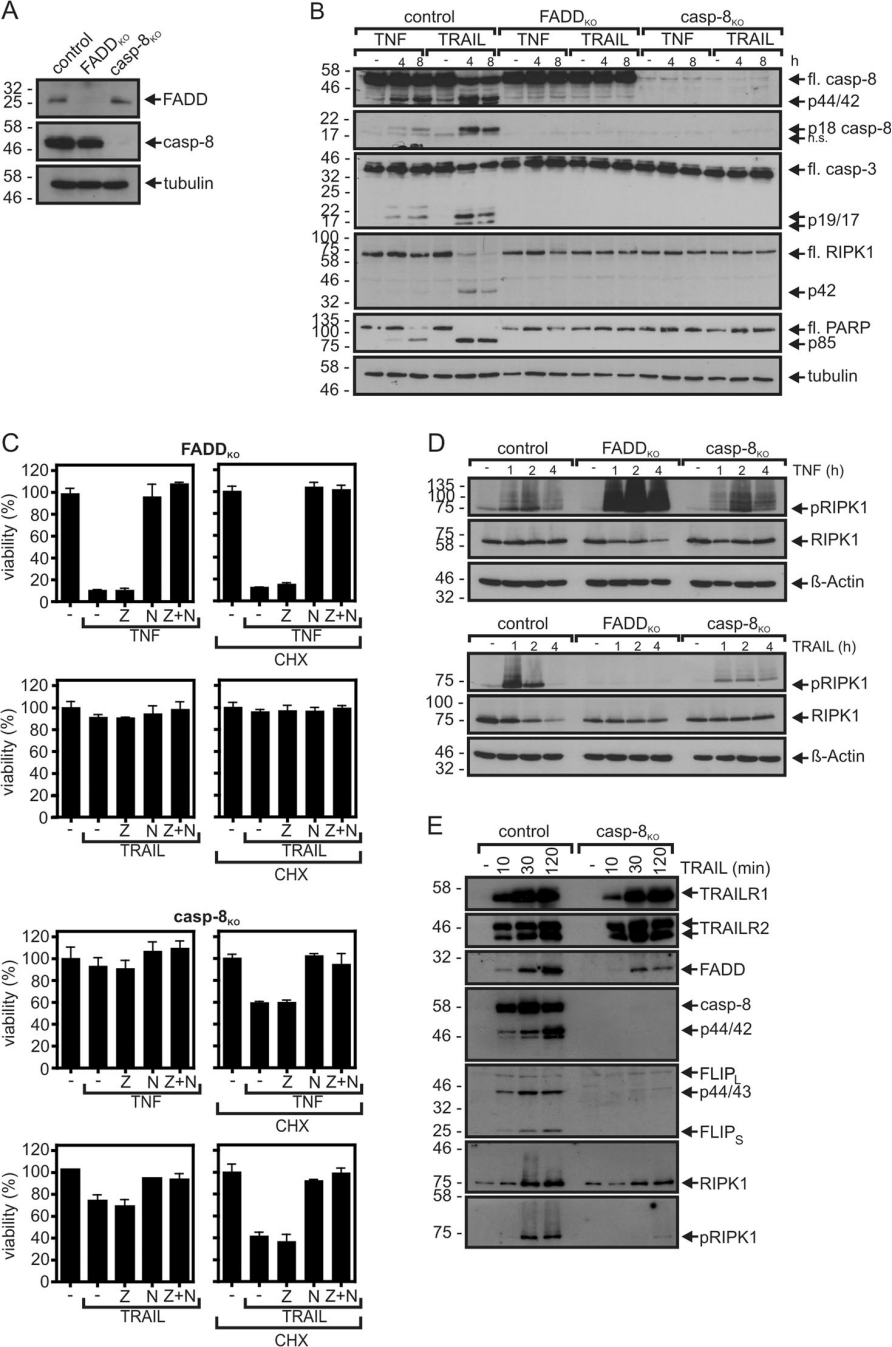
Evidence for a caspase-8-independent anti-necroptotic activity of FADD in TNFR1 signaling. **a** HeLa-RIPK3_con_, HeLa-RIPK3-FADD_KO_, and HeLa-RIPK3-casp8_KO_ cells were analyzed by western blot for expression of caspase-8 and FADD. **b** Cells were stimulated for the indicated times with 100 ng/ml TNF or 100 ng/ml TRAIL in the presence of 2.5 µg/ml CHX and total cell lysates were analyzed by western blot for processing of the indicated caspases and caspase substrates. fl full-length. **c** HeLa-RIPK3 variants were stimulated in technical triplicates as indicated with TNF (100 ng/ml), TRAIL (100 ng/ml), CHX (2.5 µg/ml), ZVAD (Z, 20 µM), and nec1 (N, 90 µM). One day later cellular viability was quantified by crystal violet staining. A representative panel of experiments is shown. For statistical analysis of independent experiments please see Tables 1 and 2 and Supplementary Tables I–III. **d** Cells were treated for the indicated times with TNF (100 ng/ml) or TRAIL (100 ng/ml). Total cell lysates were analyzed by western blot for expression and phosphorylation of RIPK1. **e** The TRAIL death receptor-associated signaling complex was immunoprecipitated from HeLa-RIPK3_con_ and HeLa-RIPK3-casp8_KO_ cells with Fc-TRAIL and protein G beads. IPs were analyzed by western blot for the presence of the indicated proteins. For western blot analysis of lysates see Supplementary Data Fig. S5C

Caspase-8 activation and apoptosis induction by TNF in TRADD proficient HeLa-RIPK3 cells are fully dependent on FADD expression (Fig. 2b–e). Since it is furthermore very well established that caspase-8 inhibits death receptor-induced necroptosis, the enhanced necroptotic TNF activity observed in FADD-deficient HeLa-RIPK3 cells (Fig. 2b, c) might simply reflect the lack of caspase-8 activation. To proof this idea, we also deleted caspase-8 from HeLa-RIPK3 cells (Fig. 6a). As expected neither TNF nor TRAIL-induced caspase activation in HeLa-RIPK3-casp8_KO_ cells (Fig. 6b). Despite the lack of caspase activation there was still significant cell death induction by TNF which was inhibitable by necrostatin-1 but not by ZVAD arguing for pure necroptosis induction (Fig. 6c, Table 1, Supplementary Table II). However, while TNF robustly induced necroptosis in HeLa-RIPK3-FADD_KO_ cells in the absence of CHX, TNF-induced necroptosis in HeLa-RIPK3-casp8_KO_ was largely dependent on sensitization with CHX (Fig. 6c, Table 1). Moreover, in contrast to FADD deficiency, deletion of caspase-8 expression poorly affected TNF-induced RIPK1 phosphorylation (Fig. 6d). Other than HeLa-RIPK3-FADD_KO_ cells, HeLa-RIPK3-casp8_KO_ cells still underwent TRAIL-induced necroptosis (Fig. 6c, Table 2). Thus, in context of TNFR1-induced necroptosis FADD seems to fulfill a dual anti-necroptotic function, first by enabling caspase-8 dependent inhibition of the ripoptosome as it has been frequently shown in the literature and second by interfering with the activity of a CHX-sensitive anti-necroptotic factor with caspase-8-independent activity. A good candidate for the latter is FLIP_L_. On the one hand, the expression of FLIP_L_ is highly CHX sensitive due to its turnover by proteasomal degradation (e.g. refs. ^54,55^). On the other hand, it has a central role in the control of death receptor signaling and has already been identified as a necroptosis inhibitory factor^46^.

We also investigated TRAIL-induced death receptor signaling complex formation in HeLa-RIPK3-casp8_KO_ cells. In accordance with their maintained necroptosis competence, TRAIL-treated HeLa-RIPK3-casp8_KO_ cells showed significant recruitment of RIPK1 into the TRAIL-DR signaling complex (Fig. 6e). RIPK1 recruitment and RIPK1 modification, however, was significantly less efficient than in HeLa-RIPK3_con_ cells. Moreover, recruitment of FLIP_L/S_ and FADD, which is essential for caspase-8, FLIP_L/S_ and RIPK1 recruitment (Fig. 2d), were also attenuated. Thus, secondary FADD-mediated recruitment of caspase-8 seems to stabilize the TRAIL death receptor-associated signaling complex.

## Discussion

We comprehensively analyzed induction of apoptosis, necroptosis, and NFκB signaling by TNFR1 and the TRAIL death receptors in a panel of HeLa-RIPK3 variants with individual knockout and double-knockout of TRADD, FADD, and RIPK1. This way, we were able to derive the following characteristics of death receptor signaling from one cellular model with the same set of reagents and methods:

I. RIPK1 is essential for necroptosis induction by the TRAIL death receptors and TNFR1. However, only in the case of TNFR1, RIPK1 is already sufficient in the absence of FADD and TRADD to induce necroptosis robustly (Figs. 2b and 5b, Tables 1 and 2, Supplementary Tables II and III).
II. TRADD inhibits TNFR1- but not TRAIL-induced apoptosis and necroptosis (Fig. 2f).
III. RIPK1 and TRADD are redundantly required for TRAIL death receptor- and TNFR1-induced proinflammatory signaling (Fig. 4a, b).
IV. RIPK1 and TRADD are redundantly needed for TNFR1-induced apoptosis but are dispensable for TRAIL death receptor-induced apoptosis (Fig. 3b, c; Tables 1 and 2, Supplementary Tables II and III).
V. FADD is not only required but also, in the absence of TRADD and RIPK1, sufficient for robust TRAIL-triggered caspase activation and cell death induction (Figs. 2b–e and  3b, c; Tables 1 and 2, Supplementary Tables II and III). In contrast, TNF killing is largely abrogated in the absence of the TRADD/RIPK1 dyad (Fig. 3b, c).
VI. FADD is required for TRAIL- but not TNF-induced proinflammatory NFκB signaling (Fig. 4d).
VII. RIPK1 allows TNF- but not TRAIL-induced caspase-8 activation in the absence of FADD and TRADD (Fig. 5b, c).
VIII. FADD is required for TRAIL-induced necroptosis but antagonizes TNF-induced necroptosis in a caspase-8 dependent and a caspase-8 independent manner (Fig. 6, Tables 1 and 2, Supplementary Tables II and III).

**Fig. 7:**
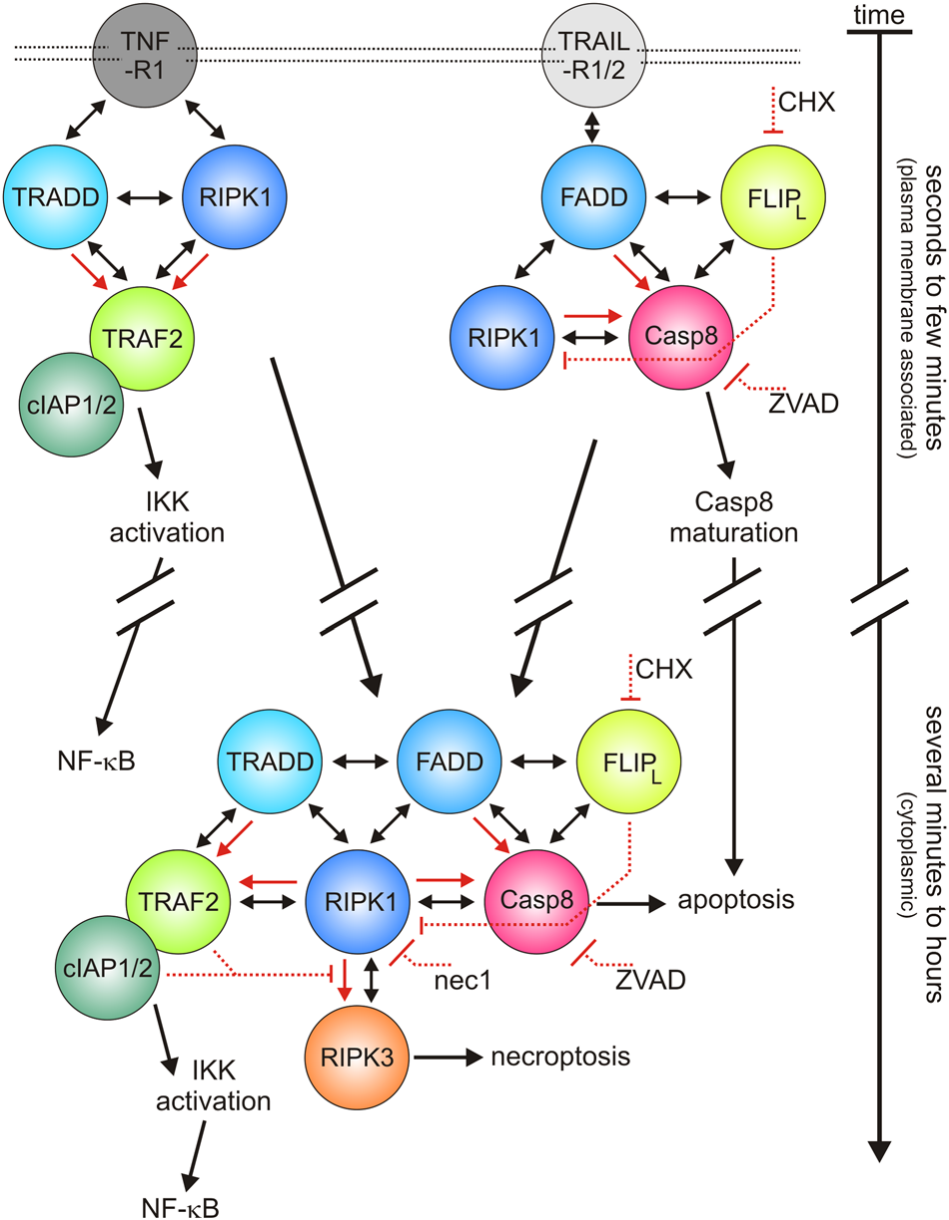
Model of TNFR1 and TRAIL death receptor signaling. For simplicity, the various literature known modifications (phosphorylation, ubiquitination, processing) and oligomerization events which enable TRADD, FADD, RIPK1 and their binding partners to control the activity of proinflammatory and cytotoxic signaling pathways are not indicated. Please note the dynamics of the cytoplasmic complex is unknown. Thus, it is unclear whether two or more relatively stable complexes are formed that interact secondarily in a transient fashion or whether all proteins can assemble into one type of complex. Double headed arrows refer to protein–protein interactions. Red headed arrows indicate activating/stimulating events. Red dotted blocked lines refer to inhibitory events/effects

All these findings (summarized in Table 3) can be explained by a model in which (i) the RIPK1-TRADD pair and FADD along with caspase-8 act in a reciprocal sequence in TNFR1- and TRAIL death receptor-induced signaling and in which (ii) caspase-8 activation is linked to FADD and RIPK1, activation of NFκB to RIPK1, and TRADD and RIPK3 activation to RIPK1 (Fig. 7). RIPK1 and TRADD act in this model upstream of FADD in TNFR1 signaling but downstream of FADD and caspase-8 in TRAIL signaling. FADD is therefore dispensable for RIPK3 activation by TNF but required in the case of TRAIL (characteristic I and VIII). FADD also recruits the anti-necroptotic molecules caspase-8 and FLIP_L_ which explains why deletion of FADD sensitizes for TNF-induced necroptosis (characteristic VIII). Since the NFκB-stimulating TRADD-RIPK1 dyad acts downstream of FADD in TRAIL death receptor signaling in our model, this furthermore explains the broadly documented need of FADD in NFκB activation by TRAIL^19–21^ (characteristic VI). This is also in good accordance with the reported TRAIL-induced formation of a cytoplasmic complex containing RIPK1, TRADD, FADD, caspase-8, and the proinflammatory TRAF2 protein^23^. Reciprocally, the positioning of FADD downstream of the TRADD-RIPK1 dyad in TNFR1 signaling in the model fits with the TNFR1-specific requirement of TRADD and/or RIPK1 for FADD-mediated caspase-8 activation and apoptosis (characteristic V). Vice versa, the direct binding of FADD to the TRAIL death receptors not only explains the well-known requirement of FADD for caspase-8 activation^11,14,16^ but also why in this context FADD is sufficient for caspase-8 activation and apoptosis in the absence of TRADD and RIPK1 (Fig. 3b, c). The known ability of RIPK1 to directly interact with caspase-8 and its TNFR1-proximal position may also explain why TNF, in contrast to TRAIL, can engage caspase-8 and apoptosis in a FADD-independent manner (characteristic VII). Since the necroptotic bottleneck RIPK1 is in our model receptor proximally located in TNF signaling but receptor distally (downstream of FADD) in TRAIL signaling, the model also gives a solid explanation for the RIPK1 sufficiency in necroptotic TNF signaling (characteristic I).

**Table 3:** Summary of TNF- and TRAIL-responses in HeLa variants lacking expression of TRADD, RIPK1, FADD, and caspase-8

Variant	TNF					TRAIL				
	Apoptosis		Necroptosis		IL8/NFκB	Apoptosis		Necroptosis		IL8/NFκB
	+N	+CN	+Z	+CZ		+N	+CN	+Z	+CZ	
EV	−	++	−	−	n.i.	−	+++	−	−	n.i.
CON	−	++	−	+++	Intact	++	+++	++	+++	Intact
TRADD-KO	+	+++	++	+++	Intact	++	+++	++	+++	Intact
FADD-KO	−	−	+++	+++	Intact	−	−	−	−	Absent
RIPK1-KO	−	++	−	−	Intact	++	+++	−	−	Intact
Casp.8-KO	−	−	−	++	n.i.	−	−	++	++	n.i.
FADD-TRADD-DKO	−	++	++	+++	n.i.	++	++	++	−	n.i.
FADD-RIPK1-DKO	−	−	−	−	n.i.	−	−	−	−	n.i.
TRADD-RIPK1-DKO	−	−	−	−	Absent	++	+++	−	−	Absent

HeLa-RIPK3 = CON; HeLa-RIPK3-FADD_KO_ = FADD-KO; HeLa-RIPK3-TRADD_KO_ = TRADD-KO; HeLa-RIPK3-RIPK1_KO_ = RIPK1-KO; HeLa-RIPK3-Casp8_KO_ = Casp.8-KO; HeLa-RIPK3-FADD/TRADD_DKO_ = FADD-TRADD-DKO; HeLa-RIPK3-FADD/RIPK1_DKO_ = FADD-RIPK1-DKO; HeLa-RIPK3-TRADD/RIPK1_DKO_ = TRADD-RIPK1-DKO; HeLa-EV = EV; N = necrostatin-1, Z = ZVAD; C = CHX; n.i. = not investigated. Cell death coding: “−” no significant effect; “+” cell death = 1–10 %; “++” cell death = 11–70%; “+++” cell death = 71–100%.

The fact that TRADD deficiency affects apoptosis and necroptosis in case of TNF but not in the case of TRAIL (characteristic II) could also be understood as a consequence of reciprocal sequential activity of TRADD/RIPK1 and FADD in TNF and TRAIL signaling. Since TRADD acts in TNF signaling parallel to RIPK1 and upstream of FADD, it can here antagonize RIPK1-mediated necroptosis and FADD-dependent caspase-8 activation. In contrast, due to its position downstream of FADD in TRAIL signaling, TRADD is in this case not protective. TRADD may fulfill its survival function in a twofold way.

First, it strongly interacts with TRAF2 and is therefore able to recruit the E3 ligases cIAP1 and cIAP2 which by RIPK1 ubiquitination prevent necroptotic RIPK1–RIPK3 interaction and apoptotic RIPK1-dependent caspase-8 activation^56^.

Second, RIPK1 K63-ubiquitinated by TRADD-associated TRAF2 and cIAP molecules furthermore serves as substrates for further linear ubiquitination by the E3 ligase complex LUBAC, resulting in recruitment and activation of linear ubiquitin-binding kinase complexes (Tab2-TAK1, IKK) required for the activation of the classical NFκB pathway in response to TNF and TRAIL^56^. In accordance with the receptor proximal position of TRADD and RIPK1 in TNF signaling, TNF-induced activation of the NFκB pathway occurs rapidly in seconds to very few minutes and much faster as caspase-8 activation via the downstream located FADD molecule. In accordance with the reciprocal hierarchy of TRADD/RIPK1 and FADD in TNFR1 and TRAIL death receptor signaling, however, there is the complementary situation in response to TRAIL: rapid caspase-8 activation and delayed NFκB activation. The rapid NFκB response upon TNFR1 activation enables anti-apoptotic NFκB targets to interfere with the slow activation of caspase-8 and apoptosis. TRADD deficiency can therefore result in enhanced TNF-induced apoptosis. In the case of TRAIL, however, the receptor proximal activation of caspase-8 is too fast to be controlled by the slow TRAIL-induced NFκB response and so TRADD deficiency has no major impact.

From our comparative analysis of HeLa-RIPK3-TRADD_KO_, HeLa-RIPK3-RIPK1_KO_, and HeLa-RIPK3-TRADD/RIPK1_DKO_ cells redundant activities of TRADD and RIPK1 in TNF- and TRAIL-induced NFκB activation and in TNF-induced apoptosis are clearly evident. This can straightforwardly explain the contradictory reports in the literature concerning the relevance of TRADD and RIPK1 for TNF/TRAIL signaling obtained with single knockout or knockdown cells (see Introduction). It appears plausible that dependent on the relative expression levels of TRADD and RIPK1 in different cellular systems, these two molecules show varying relevance for the aforementioned signaling effects. Since the RIPK1 ubiquitinating TRAF2-cIAP1/2 complexes can interact with both TRADD and RIPK1, it appears possible that the position, type, and extent of RIPK1 ubiquitination, and thus its activity, is fine-tuned by the way how TRAF2-cIAP1/2 complexes interact with RIPK1, thus directly or via TRADD. Indeed, modified RIPK1 species are still present in TNF-treated TRADD-deficient HeLa-RIPK3 cells (Figs. 2d and 4c). Future studies must show whether this reflects a general reduction in ubiquitination or a preferential reduction of certain types of ubiquitination.

In sum, our data show that caspase-8 activation and apoptosis, RIPK1/RIPK3 activation and necroptosis, and classical NFκB signaling act in a DR subtype-specific hierarchical sequence, which explains the partly DR subgroup-specific functions of TRADD, FADD, and RIPK1. Future studies must now reveal how TRADD, FADD, and RIPK1 targeting regulator proteins, e.g. FLIP and TRAF2, and the DR type-specific sequence of action of TRADD, FADD, or RIPK1 (Table 3) play together to control the balance between apoptotic, necroptotic, and proinflammatory DR signaling.

## Supplementary information

supplemental table I

supplemental table II

supplemental table III
