# Supplementary material for: Redundant and receptor-specific activities of TRADD, RIPK1 and FADD in death receptor signaling
Source: Cell Death Dis. 2019 Feb 11;10(2):122. doi: 10.1038/s41419-019-1396-5 (PMC6370826; doi:10.1038/s41419-019-1396-5)
Supplement: Supplementary file 1 — supplemental table I [file 41419_2019_1396_MOESM1_ESM.pdf]

Supplemental table I. Compilation of all viability data

HeLa-EV	N =	Viability with 100 ng/ml ligand (%)	Average (%)	EC_{50} (ng/ml)
TNF	7	74 ; 75 ; 76 ; 83 ; 96 ; 97 ; 104	86	
TNF + Z	3	88 ; 95 ; 101	95	
TNF + N	3	63 ; 93 ; 97	84	
TNF + ZN	3	87 ; 89 ; 102	93	
TNF + C	7	7 ; 9; 23 ; 25 ; 30 ; 32 ; 33	23	1,3 ; 3,1
TNF + CZ	10	85 ; 85 ; 89 ; 92 ; 98 ; 103 ; 107 ; 109 ; 109; 110	99	
TNF + CN	5	10 ; 16 ; 30 ; 56 ; 58	34	
TNF + CZN	7	72 ; 82 ; 93 ; 106 ; 108 ; 110 ;110	97	
TRAIL	8	82 ; 85 ; 90 ; 91 ; 93 ; 100 ; 115 ; 119	97	
TRAIL + Z	6	94 ; 95; 96 ; 96 ; 101 ; 106	98	
TRAIL + N	3	63 ; 68 ; 112	81	
TRAIL + ZN	4	87 ; 90 ; 97 ; 98	93	
TRAIL + C	7	0 ; 1 ; 17 ; 28 ; 30 ; 31 ; 38	21	0,9 ; 1,5 ; 2 ; 2
TRAIL + CZ	7	80 ; 91 ; 96 ; 97 ; 101 ; 104 ; 106	96	
TRAIL + CN	4	1 ; 7 ; 25 ; 37	18	1 ; 1,2 ; 12
TRAIL + CZN	4	83 ; 91 ; 99 ; 100	93	
**HeLa-RIPK3**				
TNF	45	62 ; 68 ; 68 ; 69 ; 72 ; 75 ; 78 ; 78 ; 82 ; 82 ; 83 ; 86 ; 89 ; 90 ; 90 ; 92 ; 92 ; 95 ; 96 ; 96 ; 98 ; 98 ; 99 ; 99 ; 99 ; 100 ; 100 ; 101 ; 102 ; 103 ; 103 ; 104 ; 104 ; 105 ; 105 ; 105 ; 106 ; 106 ; 107 ; 108 ; 108 ; 108 ; 108 ; 109 ; 110	94	
TNF + Z	39	80 ;80 ; 81 ; 82 ; 89 ; 90 ; 91 ; 91 ; 93 ; 95 ; 95 ; 96 ; 96 ; 97 ; 97 ; 98 ; 98 ; 98 ; 99 ; 101 ; 101 ; 102 ; 102 ; 102 ; 102 ; 102 ; 103 ; 103 ; 103 ; 103 ; 104 ; 104 ; 104 ; 106 ; 108 ; 109 ; 110 ; 110 ; 110	98	
TNF + N	29	82 ; 86 ; 87 ; 91 ; 92 ; 92 ; 93 ; 95 ; 95 ; 95 ; 96 ; 97 ; 98 ; 98 ; 98 ; 99 ; 100 ; 100 ; 100 ; 101 ; 101 ; 101 ; 103 ; 103 ; 104 ; 104 ; 104 ; 105 ; 106	97	
TNF + ZN	35	80 ; 83 ; 84 ; 84 ; 85 ; 88 ; 90 ; 90 ; 91 ; 92 ; 92 ; 94 ; 94 ; 95 ; 96 ; 96 ; 96 ; 98 ; 98 ; 98 ; 98 ; 99 ; 100 ; 101 ; 101 ; 102 ; 102 ; 102 ; 103 ; 104 ; 105 ; 107 ; 107 ; 109 ; 110	96	
TNF + C	9	0 ; 6 ; 16 ; 21 ; 28 ; 32 ; 35 ; 37 ; 40	24	0,2 ; 0,2 ; 0,2 ; 0,6 ; 0,6 ; 2,2 ; 5,2 ; 5,2 ; 7,9
TNF + CZ	4	9 ; 14 ; 25 ; 29	19	0,005 ; 0,03 ; 0,2 ; 5,2
TNF + CN	21	50 ; 51 ; 51 ; 52 ; 52 ; 56 ; 58 ; 59 ; 60 ; 61 ; 61 ; 63 ; 65 ; 65 ; 67 ; 68 ; 72 ; 77 ; 82 ; 83 ; 84	64	
TNF + CZN	23	82 ; 82 ; 83 ; 85 ; 86 ; 86 ; 88 ; 89 ; 92 ; 93 ; 93 ; 93 ; 94 ; 101 ; 101 ; 103 ; 103 ; 104 ; 105 ; 105 ; 106 ; 107 ; 108	95	
TRAIL	33	70 ; 71 ; 71 ; 73 ; 73 ; 74 ; 78 ; 79 ; 81 ; 81 ; 82 ; 82 ; 83 ; 86 ; 87 ; 88 ; 88 ; 88 ; 89 ; 90 ; 90 ; 92 ; 94 ; 94 ; 95 ; 96 ; 97 ; 97 ; 98 ; 98 ; 100 ; 103 ; 107	87	
TRAIL + Z	27	52 ; 55 ; 57 ; 59 ; 60 ; 60 ; 62 ; 66 ; 68 ; 74 ; 75 ; 78 ; 79 ; 81 ; 82 ; 82 ; 85 ; 88 ; 89 ; 91 ; 93 ; 95 ; 97 ; 98 ; 100 ; 101 ; 101	79	
TRAIL + N	17	51 ; 53 ; 54 ; 56 ; 58 ; 58 ; 59 ; 59 ; 64 ; 65 ; 66 ; 67 ; 72 ; 72 ; 82 ; 86 ; 87	65	
TRAIL + ZN	25	80 ; 83 ; 85 ; 86 ; 86 ; 88 ; 90 ; 90 ; 92 ; 93 ; 93 ; 94 ; 94 ; 95 ; 97 ; 98 ; 99 ; 99 ; 99 ; 99 ; 102 ; 102 ; 104 ; 108 ; 109	95	
TRAIL + C	19	0 ; 0 ; 2 ; 3 ; 4 ; 4 ; 5 ; 5 ; 6 ; 7 ; 8 ; 8 ; 8 ; 9 ; 9 ; 9 ; 11 ; 12 ; 22	7	0,5 ; 0,8 ; 1 ; 1,4 ; 1,4 ; 1,4 ; 1,5 ; 1,6 ; 1,6 ; 1,8 ; 1,8 ; 2,1 ; 2,1 ; 3,2 ; 3,5 ; 4 ; 4,6 ; 5,7 ; 8,1
TRAIL + CZ	8	11 ; 18 ; 22 ; 31 ; 32 ; 32 ;32 ; 46	28	2,9 ; 3 ; 3,1 ; 3,9 ; 4,2 ; 4,2 ; 4,7 ; 4,9
TRAIL + CN	5	0 ; 3 ; 3 ; 11 ; 14	6	1,1 ; 2,4 ; 3,9 ; 4,7 ; 8,1
TRAIL + CZN	17	90 ; 91 ; 91 ; 93 ; 94 ; 94 ; 96 ; 97 ; 98 ; 99 ; 99 ; 100 ; 102 ; 103 ; 106 ; 106 ; 109	98	
**$FADD_{KO}$**				
TNF	20	0 ; 0 ; 0 ;1 ; 1 ; 1 ; 1 ; 2 ; 2 ; 2 ; 3 ; 3 ; 6 ; 11 ; 15 ; 19 ;25 ; 31 ; 40 ; 40	10	0,02 ; 0,1 ;0,2 ; 0,2 ; 0,4 ; 0,4 ; 0,4 ; 0,4 ; 0,4 ; 0,5 ; 0,5 ; 0,6 ; 0,6 ; 0,8 ; 0,8

				; 0,8 ; 1 ; 1,5 ; 2,8 ; 3,4
TNF + Z	4	0 ; 5 ; 11 ; 13	7	0,05 ; 0,2 ; 0,2 ; 1
TNF + N	21	82 ; 85 ; 86 ; 89 ; 89 ; 90 ; 94 ; 94 ; 95 ; 98 ; 99 ; 99 ; 99 ; 99 ; 100 ; 100 ; 100 ; 103 ; 105 ; 106 ; 109	96	
TNF + ZN	25	85 ; 86 ; 88 ; 90 ; 90 ; 91 ; 95 ; 96 ; 97 ; 98 ; 99 ; 99 ; 102 ; 103 ; 103 ; 103 ; 103 ; 105 ; 105 ; 105 ; 107 ; 108 ; 108 ; 110 ; 110	99	
TNF + C	5	1 ; 8 ; 36 ; 40 ; 41	25	0,002 ; 0,004 ; 0,008 ; 0,008 ; 0,1
TNF + CZ	4	6 ; 21 ; 22 ; 41	23	0,02 ; 0,03 ; 0,06 ; 0,08
TNF + CN	16	81 ; 85 ; 85 ; 89 ; 93 ; 94 ; 95 ; 95 ; 97 ; 100 ; 103 ; 103 ; 104 ; 104 ; 104 ; 106	96	
TNF + CZN	20	80 ; 80 ; 86 ; 91 ; 91 ; 92 ; 93 ; 94 ; 98 ; 101 ; 101 ; 102 ; 103 ; 104 ; 104 ; 105 ; 106 ; 106 ; 107 ; 108	98	
TRAIL	31	81 ; 82 ; 83 ; 87 ; 88 ; 88 ; 88 ; 88 ; 91 ; 92 ; 92 ; 94 ; 94 ; 94 ; 98 ; 98 ; 100 ; 100 ; 101 ; 102 ; 102 ; 103 ; 103 ; 103 ; 104 ; 105 ; 107 ; 107 ; 107 ; 109 ; 110	97	
TRAIL + Z	28	62 ; 65 ; 65 ; 67 ; 68 ; 83 ; 86 ; 89 ; 90 ; 90 ; 91 ; 93 ; 93 ; 95 ; 96 ; 97 ; 97 ; 99 ; 100 ; 101 ; 101 ; 101 ; 102 ; 104 ; 104 ; 106 ; 108 ; 109	92	
TRAIL + N	26	78 ; 81 ; 82 ; 86 ; 87 ; 89 ; 91 ; 90 ; 90 ; 90 ; 94 ; 94 ; 94 ; 95 ; 95 ; 96 ; 97 ; 98 ; 99 ; 99 ; 103 ; 106 ; 107 ; 108 ; 109 ; 110	95	
TRAIL + ZN	22	84 ; 84 ; 86 ; 87 ; 89 ; 91 ; 93 ; 94 ; 96 ; 97 ; 97 ; 97 ; 98 ; 98 ; 99 ; 102 ; 106 ; 106 ; 107 ; 109 ; 109 ; 110	97	
TRAIL + C	31	62 ; 64 ; 65 ; 66 ; 78 ; 78 ; 80 ; 82 ; 82 ; 83 , 86 ; 86 ; 87 ; 88 ; 88 ; 89 ; 89 ; 90 ; 91 ; 91 , 93 ; 94 ; 95 ; 95 ; 96 ; 96 ; 99 ; 100 ; 107 ; 107 ; 109	88	
TRAIL + CZ	22	63 ; 69 ; 71 ; 72 ; 80 ; 82 ; 83 ; 85 ; 86 ; 87 ; 87 ; 96 ; 97 ; 97 ; 97 ; 98 ; 98 ; 100 ; 101 ; 102 ; 108 ; 109	89	
TRAIL + CN	22	68 ; 70 ; 71 ; 72 ; 79 ; 80 ; 81 ; 82 ; 84 ; 85 ; 92 ; 92 ; 97 ; 97 ; 97 ; 98 ; 98 ; 99 ; 100 ; 103 ; 106 ; 108	89	
TRAIL + CZN	17	80 ; 81 ; 81 ; 82 ; 82 ; 84 ; 88 ; 89 ; 94 ; 95 ; 97 ; 99 ; 99; 100 ; 101 ; 107 ; 109	92	
**$TRADD_{KO}$**				
TNF	29	71 ; 82 ; 83 ; 85 ; 87 ; 88 ; 89 ; 89 ; 90 ; 90 ; 90 ; 92 ; 92 ; 92 ; 94 ; 94 ; 94 ; 94; 96 ; 97 ; 98 ; 98 ; 99 ; 99 ; 102 ; 102 ; 105 ; 106 ; 106	93	
TNF + Z	28	60 ; 62 ; 63 ; 67 ; 67 ; 67 ; 68 ; 75 ; 80 ; 81 ; 82 ; 84 ; 85 ; 85 ; 88 ; 88 ; 89 ; 91 ; 92 ; 92 ; 93 ; 94 ; 95 ; 98 ; 98 ; 100 ; 101 ; 104	84	
TNF + N	26	76 ; 76 ; 78 ; 79 ; 80 ; 81 ; 81 ; 84 ; 86 ; 87 ; 88 ; 88 ; 89 ; 90 ; 91 ; 91 ; 93 ; 93 ; 94 ; 95 ; 96 ; 100 ; 103 ; 104 ; 104 ; 107	90	
TNF + ZN	22	81 ; 84 ; 84 ; 87 ; 90 ; 92 ; 93 ; 94 ; 96 ; 96 ; 96 ; 97 ; 97 ; 98 ; 98 ; 99 ; 101 ; 104 ; 105 ; 108 ; 109 ; 110	96	
TNF + C	6	0 ; 0 ; 0 ; 2 ; 10 ; 36	8	0,009 ; 0,03 ; 0,03 ; 0,04 ; 0,06 ; 0,2
TNF + CZ	4	19 ; 21 ; 21 ; 28	22	0,002 ; 0,008 ; 0,03 ; 0,07
TNF + CN	4	20 ; 23 ; 27 ; 43	28	0,04 ; 0,1 ; 0,2 ; 0,2
TNF + CZN	17	80 ; 81 ; 86 ; 88 ; 89 ; 89 ; 92 ; 93 ; 97 ; 97 ; 98 ; 99 ; 100 ; 105 ; 106 ; 107 ; 107	95	
TRAIL	16	80 ; 82 ; 85 ; 87 ; 88 ; 90 ; 91 ; 93 ; 95 ; 96 ; 97 ; 99 ; 100 ; 100 ; 101 ; 103	93	
TRAIL + Z	10	63 ; 64 ; 70 ; 76 ; 87 ; 92 ; 93 ; 94 ; 96 ; 99	83	
TRAIL + N	9	69 ; 70 ; 74 ; 75 ; 77 ; 84 ; 87 ; 88 ; 89	79	
TRAIL + ZN	12	90 ; 93 ; 93 ; 93 ; 97 ; 100 ; 100 ; 101 ; 107 ; 109 ; 110 ; 110	100	
TRAIL + C	9	0 ; 0 ; 1 ; 1 ; 9 ; 9 ; 15 ; 17 ; 23	8	0,04 ; 0,3 ; 0,6 ; 0,9 ; 1,9 ; 2,9 ; 3,3 ; 3,6 ; 4
TRAIL + CZ	6	0 ; 0 ; 6 ; 16 ; 17 ; 28	11	1 ; 1,1 ; 1,6 ; 2,3 ; 2,4 ; 2,7
TRAIL + CN	5	8 ; 8 ; 15 ; 16 ; 28	15	2 ; 2,4 ; 2,7 ; 5,2 ; 8,9
TRAIL + CZN	9	87 ; 91 ; 95 ; 95 ; 96 ; 97 ; 98 ; 100 ; 109	96	
**$RIPK1_{KO}$**				
TNF	24	80 ; 87 ; 87 ; 88 ; 92 ; 93 ; 95 ; 96 ; 97 ; 97 ; 98 ; 98 ; 100 ; 100 ; 100 ; 102 ; 103 ; 104 ; 105 ; 106 ; 106 ; 107 ; 110 ; 110	98	
TNF + Z	19	92 ; 94 ; 94 ; 95 ; 96 ; 97 ; 97 ; 98 ; 98 ; 100 ; 101 ; 101 ; 105 ; 106 ; 107 ; 108 ; 108 ; 108 ; 109	101	

TNF + N	21	72 ; 73 ; 75 ; 83 ; 84 ; 87 ; 88 ; 89 ; 92 ; 92 ; 93 ; 94 ; 94 ; 94 ; 99 ; 99 ; 100 ; 101 ; 102 ; 105 ; 106	92	
TNF + ZN	17	80 ; 88 ; 91 ; 93 ; 95 ; 96 ; 96 ; 98 ; 98 ; 98 ; 99 ; 101 ; 104 ; 106 ; 106 ; 107 ; 109	98	
TNF + C	7	6 ; 13 ; 14 ; 21 ; 28 ; 28 ; 43	22	0,1 ; 0,2 ; 0,2 ; 0,6 ; 0,6 ; 1,2 ; 5,2
TNF + CZ	15	89 ; 91 ; 92 ; 93 ; 94 ; 94 ; 95 ; 96 ; 97 ; 99 ; 99 ; 101 ; 101 ; 106 ; 110	97	
TNF + CN	4	15 ; 26 ; 35 ; 45	30	0,5 ; 0,6 ; 7 ; 31
TNF + CZN	15	87 : 87 ; 93 ; 96 ; 97 ; 97 ; 98 ; 98 ; 101 ; 102 : 102 ; 104 ; 104 ; 105 ; 107	99	
TRAIL	21	71 ; 72 ; 73 ; 79 ; 80 ; 81 ; 83 ; 83 ; 84 ; 85 ; 88 ; 88 ; 88 ; 89 ; 91 ; 92 ; 93 ; 94 ; 97 ; 100 ; 107	87	
TRAIL + Z	15	81 ; 83 ; 89 ; 92 ; 94 ; 94 ; 96 ; 98 ; 98 ; 100 ; 100 ; 100 ; 102 ; 105 ; 106	96	
TRAIL + N	13	65 ; 69 ; 69 ; 71 ; 72 ; 73 ; 78 ; 81 ; 82 ; 83 ; 84 ; 87 ; 88	77	
TRAIL + ZN	16	83 ; 86 ; 86 ; 87 ; 89 ; 90 ; 92 ; 93 ; 94 ; 95 ; 95 ; 99 ; 101 ; 101 ; 103 ; 107	94	
TRAIL + C	7	4 ; 4 ; 7 ; 8 ; 9 ; 16 ; 18	9	2,1 ; 2,8 ; 3 ; 3 ; 3,3 ; 3,4 ; 4,5
TRAIL + CZ	12	80 ; 83 ; 88 ; 90 ; 92 ; 93 ; 95 ; 97 ; 99 ; 106 ; 108 ; 109	95	
TRAIL + CN	5	1 ; 3 ; 11 ; 18 ; 23	11	0,2 ; 0,3 ; 1,1 ; 1,1 ; 2,7
TRAIL + CZN	11	85 ; 87; 91 ; 92 ; 96 ; 97 ; 97 ; 98 ; 99 ; 101 ; 101	95	

**FADD-TRADD$_{DKO}$**				
TNF	16	71 ; 79 ; 81 ; 81 ; 82 ; 84 ; 90 ; 91 ; 92 ; 92 ; 93 ; 93 ; 93 ; 96 ; 100 ; 100	89	
TNF + Z	12	73 ; 76 ; 76 ; 79 ; 80 ; 81 ; 84 ; 89 ; 89 ; 94 ; 95 ; 95	84	
TNF + N	17	70 ; 73 ; 77 ; 81 ; 84 ; 87 ; 87 ; 89 ; 91 ; 93 ; 93 ; 94 ; 99 ; 100 ; 101 ; 102 ; 106	90	
TNF + ZN	16	81 ; 90 ; 91 ; 92 ; 92 ; 94 ; 95 ; 97 ; 99 ; 100 ; 100 ; 104 ; 105 ; 106 ; 106 ; 110	98	
TNF + C	4	0 ; 0 ; 6 ; 18	6	0,0004 ; 0,03 ; 0,04 ; 1,1
TNF + CZ	4	0 ; 1 ; 2 ; 40	11	0,003 ; 0,006 ; 0,02 ; 0,1
TNF + CN	8	50 ; 52 ; 61 ; 68 ; 72 ; 72 ; 83 ; 89	68	
TNF + CZN	11	80 ; 83 ; 86 ; 90 ; 93 ; 97 ; 97 ; 99 ; 101 ; 102 ; 108	94	
TRAIL	10	67 ; 70 ; 70 ; 74 ; 78 ; 83 ; 90 ; 91 ; 91 ; 93	81	
TRAIL + Z	11	59 ; 60 ; 68 ; 71 ; 73 ; 77 ; 82 ; 82 ; 85 ; 88 ; 96	76	
TRAIL + N	10	58 ; 58 ; 63 ; 68 ; 75 ; 77 ; 81 ; 90 ; 98 ; 101	77	
TRAIL + ZN	15	83 ; 88 ; 90 ; 91 ; 91 ; 92 ; 92 ; 92 ; 95 ; 95 ; 95 ; 103 ; 103 ; 105 ; 108	95	
TRAIL + C	9	52 ; 55 ; 63 ; 63 ; 74 ; 78 ; 78 ; 88 ; 93	72	
TRAIL + CZ	5	83 ; 86 ; 95 ; 100 ; 110	95	
TRAIL + CN	7	54 ; 64 ; 67 ; 72 ; 78 ; 92 ; 99	75	
TRAIL + CZN	10	81 ; 81 ; 84 ; 89 ; 96 ; 97 ; 98 ; 100 ; 102 ; 105	93	
**FADD + RIPK1$_{DKO}$**				
TNF	19	85 ; 86 ; 91 ; 91 ; 91 ; 97 ; 97 ; 97 ; 98 ; 100 ; 103 ; 106 ; 106 ; 106 ; 106 ; 107 ; 108 ; 110 ; 110	100	
TNF + Z	17	91 ; 93 ; 94 ; 95 ; 97 ; 97 ; 98 ; 99 ; 100 ; 101 ; 102 ; 102 ; 104 ; 106 ; 107 ; 109 ; 110	100	
TNF + N	15	89 ; 90 ; 90 ; 92 ; 92 ; 93 ; 94 ; 94 ; 94 ; 95 ; 96 ; 96 ; 100 ; 100 ; 104	95	
TNF + ZN	15	82 ; 89 ; 91 ; 92 ; 95 ; 96 ; 97 ; 98 ; 98 ; 99 ; 99 ; 99 ; 100 ; 108 ; 109	97	
TNF + C	11	80 ; 81 ; 87 ; 90 ; 95 ; 95 ; 96 ; 98 ; 101 ; 103 ; 106	94	
TNF + CZ	15	70 ; 73 ; 82 ; 83 ; 86 ; 89 ; 91 ; 94 ; 97 ; 98 ; 99 ; 101 ; 104 ; 105 ; 105	92	
TNF + CN	12	71 ; 73 ; 75 ; 84 ; 94 ; 95 ; 95 ; 96 ; 96 ; 97 ; 100 ; 103	90	
TNF + CZN	11	83 ; 90 ; 96 ; 96 ; 96 ; 97 ; 100 ; 102 ; 104 ; 104 ; 109	98	
TRAIL	12	91 ; 94 ; 96 ; 96 ; 98 ; 98 ; 100 ; 101 ; 103 ; 103 ; 104 ; 106	99	
TRAIL + Z	16	78 ; 79 ; 87 ; 95 ; 96 ; 97 ; 99 ; 99 ; 99 ; 101 ; 103 ; 103 ; 105 ; 105 ; 105 ; 110	98	
TRAIL + N	16	80 ; 81 ; 85 ; 87 ; 89 ; 91 ; 91 ; 92 ; 96 ; 96 ; 96 ; 97 ; 98 ; 99 ; 99 ; 101	92	
TRAIL + ZN	19	86 ; 88 ; 88 ; 89 ; 90 ; 90 ; 91 ; 92 ; 94 ; 97 ; 97 ; 98 ; 100 ; 100 ; 101 ; 102 ; 103 ; 105 ; 106	96	
TRAIL + C	14	69 ; 78 ; 80 ; 81 ; 85 ; 87 ; 87 ; 87 ; 88 ; 92 ; 92 ; 96 ; 97 ; 101	87	

TRAIL + CZ	11	73 ; 79 ; 81 ; 84 ; 85 ; 91 ; 91 ; 92 ; 92 ; 93 ; 104	88	
TRAIL + CN	12	75 ; 75 ; 77 ; 78 ; 78 ; 81 ; 85 ; 88 ; 92 ; 104 ; 107 ; 109	87	
TRAIL + CZN	10	85 ; 88 ; 92 ; 95 ; 97 ; 98 ; 98 ; 98 ; 98 ; 98	95	
**TRADD + RIPK1$_{DKO}$**				
TNF	31	82 ; 89 ; 89 ; 89 ; 89 ; 93 ; 94 ; 94 ; 95 ; 95 ; 95 ; 96 ; 96 ; 97 ; 97 ; 98 ; 98 ; 98 ; 98 ; 100 ; 100 ; 100 ; 101 ; 102 ; 102 ; 103 ; 103 ; 103 ; 104 ; 105 ; 107	97	
TNF + Z	18	86 ; 88 ; 92 ; 93 ; 93 ; 93 ; 94 ; 94 ; 95 ; 96 ; 98 ; 100 ; 101 ; 102 ; 102 ; 108 ; 109 ; 109	97	
TNF + N	22	83 ; 83 ; 85 ; 88 ; 88 ; 88 ; 89 ; 91 ; 91 ; 92 ; 93 ; 94 ; 96 ; 96 ; 97 ; 99 ; 99 ; 99 ; 101 ; 102 ; 103 ; 107	94	
TNF + ZN	22	80 ; 80 ; 82 ; 83 ; 86 ; 87 ; 88 ; 89 ; 90 ; 90 ; 91 ; 91 ; 92 ; 94 ; 96 ; 97 ; 98 ; 102 ; 102 ; 103 ; 103 ; 104	92	
TNF + C	23	69 ; 76 ; 77 ; 78 ; 79 ; 81 ;81 ; 82 ; 85 ; 85 ; 85 ; 86 ; 90 ; 90 ; 91 ; 91 ; 93 ; 94 ;95 ; 95 ; 97 ; 103 ; 106	87	
TNF + CZ	16	83 ; 85 ; 88 ; 92 ; 93 ; 93 ; 94 ; 95 ; 97 ; 98 ; 101 ; 103 ; 104 ; 108 ; 109 ; 110	97	
TNF + CN	19	74 ; 81 ; 83 ; 83 ; 83 ; 85 ; 87 ; 88 ; 89 ; 91 ; 92 ; 93 ; 93 ; 93 ; 95 ; 96 ; 97 ; 98 ; 101	90	
TNF + CZN	15	81 ; 86 ; 87 ; 92 ; 93 ; 95 ; 98 ; 100 ; 103 ; 104 ; 104 ; 105 ; 105 ; 106 ; 108	98	
TRAIL	12	53 ; 56 ; 60 ; 73 ; 75 ; 78 ; 81 ; 83 ; 85 ; 93 ; 93 ; 93	77	
TRAIL + Z	12	78 ; 81 ; 82 ; 89 ; 89 ; 91 ; 94 ; 95 ; 99 ; 100 ; 102 ; 103	92	
TRAIL + N	9	51 ; 52 ; 62 ; 65 ; 70 ; 72 ; 78 ; 79 ; 83	68	
TRAIL + ZN	10	86 ; 89 ; 94 ; 95 ; 101 ; 101 ; 101 ; 102 ; 106 ; 107	98	
TRAIL + C	4	0 ; 0 ; 23 ; 26	12	0,1 ; 0,6 ; 1,1 ; 25
TRAIL + CZ	8	85 ; 91 ; 94 ; 96 ; 101 ; 103 ; 106 ; 109	98	
TRAIL + CN	4	3 ; 6 ; 14 ; 23	12	0,2 ; 0,3 ; 0,3 ; 2,9
TRAIL + CZN	11	84 ; 85 ; 94 ; 95 ; 95 ; 98 ; 99 ; 103 ; 103 ; 105 ; 107	97	
**Casp-8$_{KO}$**				
TNF	14	81 ; 81 ; 82 ; 83 ; 85 ; 86 ; 86 ; 88 ; 90 ; 93 ; 93 ; 94 ; 95 ; 102	89	
TNF + Z	17	78 ; 80 ; 81 ; 83 ; 83 ; 84 ; 89 ; 89 ; 90 ; 90 ; 91 ; 93 ; 93 ; 103 ; 104 ; 106 ; 106	91	
TNF + N	12	88 ; 88 ; 92 ; 93 ; 95 ; 96 ; 100 ; 103 ; 105 ; 105 ; 106 ; 107	98	
TNF + ZN	12	89 ; 92 ; 95 ; 95 ; 95 ; 97 ; 102 ; 103 ; 106 ; 106 ; 108 ; 109	100	
TNF + C	16	50 ; 52 ; 53 ; 56 ; 57 ; 59 ; 60 ; 61 ; 63 ; 64 ; 65 ; 66 ; 67 ; 69 ; 74 ; 78	62	
TNF + CZ	12	55 ; 56 ; 57 ; 57 ; 58 ; 59 ; 60 ; 65 ; 72 ; 72 ; 73 ; 77	63	
TNF + CN	15	86 ; 87 ; 93 ; 93 ; 97 ; 97 ; 98 ; 99 ; 99 ; 100 ; 101 ; 102 ; 103 ; 105 ; 109	98	
TNF + CZN	15	81 ; 82 ; 89 ; 89 ; 92 ; 93 ; 93 ; 98 ; 101 ; 101 ; 102 ; 103 ; 107 ; 108 ; 108	96	
TRAIL	15	72 ; 73 ; 74 ; 74 ; 80 ; 81 ; 86 ; 87 ; 88 ; 89 ; 93 ; 93 ; 97 ; 100 ; 102	86	
TRAIL + Z	13	51 ; 53 ; 63 ; 70 ; 77 ; 77 ; 79 ; 81 ; 82 ; 88 ; 88 ; 94 ; 98	77	
TRAIL + N	10	90 ; 94 ; 95 ; 96 ; 97 ; 100 ; 102 ; 103 ; 104 ; 105	99	
TRAIL + ZN	9	94 ; 97 ; 98 ; 99 ; 100 ; 101 ; 103 ; 103 ; 106	100	
TRAIL + C	13	52 ; 52 ; 54 ; 63 ; 65 ; 65 ; 65 ; 67 ; 69 ; 70 ; 73 ; 74 ; 76	65	
TRAIL + CZ	8	54 ; 56 ; 61 ; 63 ; 64 ; 65 ; 67 ; 68	62	
TRAIL + CN	13	77 ; 78 ; 79 ; 89 ; 90 ; 90 ; 92 ; 95 ; 96 ; 96 ; 98 ; 101 ; 109	92	
TRAIL + CZN	10	82 ; 85 ; 92 ; 93 ; 99 ; 100 ; 100 ; 104 ; 105 ; 106	97	
