# Supplementary material for: Redundant and receptor-specific activities of TRADD, RIPK1 and FADD in death receptor signaling
Source: Cell Death Dis. 2019 Feb 11;10(2):122. doi: 10.1038/s41419-019-1396-5 (PMC6370826; doi:10.1038/s41419-019-1396-5)
Supplement: Supplementary file 2 — supplemental table II [file 41419_2019_1396_MOESM2_ESM.pdf]

Supplemental table II. Complete list of comparisons of TNF-treated cell variants.

Viability data of with 100 ng/ml TNF treated cells for all HeLa-RIPK3 variants and HeLa-EV cells and all co-treatment conditions were compiled and analyzed by ANOVA (one-way, Bonferroni comparison of all pairs of columns) using the GraphPad Prism5 software.

HeLa-RIPK3 = CON; HeLa-RIPK3-$FADD_{KO}$ = FADD-KO; HeLa-RIPK3-$TRADD_{KO}$ = TRADD-KO; HeLa-RIPK3-$RIPK1_{KO}$ = RIPK1-KO; HeLa-RIPK3-$Casp8_{KO}$ = Casp.8-KO; HeLa-RIPK3-FADD/$TRADD_{DKO}$ = FADD-TRADD-DKO; HeLa-RIPK3-FADD/$RIPK1_{DKO}$ = FADD-RIPK1-DKO; HeLa-RIPK3-TRADD/$RIPK1_{DKO}$ = TRADD-RIPK1-DKO; HeLa-EV = EV; Z = ZVAD; N = necrostatin-1; C = CHX; vs = versus; ns = non specific; *** = p < 0.001; ** = p < 0.01; * = p < 0.05.

Bonferroni's Multiple Comparison Test	Mean Diff. (%)	P value
CON vs CON TNF	5,8	ns
CON vs CON TNF+Z	1,7	ns
CON vs CON TNF+N	2,6	ns
CON vs CON TNF+Z+N	3,6	ns
CON vs CON TNF+C	76	***
CON vs CON TNF+C+Z	81	***
CON vs CON TNF+C+N	36	***
CON vs CON TNF+C+Z+N	4,8	ns
FADD-KO vs FADD-KO TNF	90	***
FADD-KO vs FADD-KO TNF+Z	93	***
FADD-KO vs FADD-KO TNF+N	3,8	ns
FADD-KO vs FADD-KO TNF+Z+N	0,56	ns
FADD-KO vs FADD-KO TNF+C	75	***
FADD-KO vs FADD-KO TNF+C+Z	78	***
FADD-KO vs FADD-KO TNF+C+N	3,9	ns
FADD-KO vs FADD-KO TNF+C+Z+N	2,4	ns
TRADD-KO vs TRADD-KO TNF	6,8	ns
TRADD-KO vs TRADD-KO TNF+Z	16	***
TRADD-KO vs TRADD-KO TNF+N	10	**
TRADD-KO vs TRADD-KO TNF+Z+N	3,7	ns
TRADD-KO vs TRADD-KO TNF+C	92	***
TRADD-KO vs TRADD-KO TNF+C+Z	78	***
TRADD-KO vs TRADD-KO TNF+C+N	72	***
TRADD-KO vs TRADD-KO TNF+C+Z+N	5,1	ns
RIPK1-KO vs RIPK1-KO TNF	1,6	ns
RIPK1-KO vs RIPK1-KO TNF+Z	-0,74	ns
RIPK1-KO vs RIPK1-KO TNF+N	8,5	ns
RIPK1-KO vs RIPK1-KO TNF+Z+N	2,1	ns
RIPK1-KO vs RIPK1-KO TNF+C	78	***
RIPK1-KO vs RIPK1-KO TNF+C+Z	2,9	ns
RIPK1-KO vs RIPK1-KO TNF+C+N	70	***
RIPK1-KO vs RIPK1-KO TNF+C+Z+N	1,5	ns
FADD-TRADD-DKO vs FADD-TRADD-DKO TNF	11	*
FADD-TRADD-DKO vs FADD-TRADD-DKO TNF+Z	16	***
FADD-TRADD-DKO vs FADD-TRADD-DKO TNF+N	10	ns
FADD-TRADD-DKO vs FADD-TRADD-DKO TNF+Z+N	2,4	ns
FADD-TRADD-DKO vs FADD-TRADD-DKO TNF+C	94	***
FADD-TRADD-DKO vs FADD-TRADD-DKO TNF+C+Z	89	***
FADD-TRADD-DKO vs FADD-TRADD-DKO TNF+C+N	32	***
FADD-TRADD-DKO vs FADD-TRADD-DKO TNF+C+Z+N	5,8	ns
FADD-RIPK1-DKO vs FADD-RIPK1-DKO TNF	0,26	ns
FADD-RIPK1-DKO vs FADD-RIPK1-DKO TNF+Z	-0,29	ns
FADD-RIPK1-DKO vs FADD-RIPK1-DKO TNF+N	5,4	ns
FADD-RIPK1-DKO vs FADD-RIPK1-DKO TNF+Z+N	3,2	ns
FADD-RIPK1-DKO vs FADD-RIPK1-DKO TNF+C	6,2	ns
FADD-RIPK1-DKO vs FADD-RIPK1-DKO TNF+C+Z	8,2	ns
FADD-RIPK1-DKO vs FADD-RIPK1-DKO TNF+C+N	10	ns
FADD-RIPK1-DKO vs FADD-RIPK1-DKO TNF+C+Z+N	2,1	ns
TRADD-RIPK1-DKO vs TRADD-RIPK1-DKO TNF	2,8	ns
TRADD-RIPK1-DKO vs TRADD-RIPK1-DKO TNF+Z	2,6	ns

TRADD-RIPK1-DKO vs TRADD-RIPK1-DKO TNF+N	6,2	ns
TRADD-RIPK1-DKO vs TRADD-RIPK1-DKO TNF+Z+N	7,8	ns
TRADD-RIPK1-DKO vs TRADD-RIPK1-DKO TNF+C	13	***
TRADD-RIPK1-DKO vs TRADD-RIPK1-DKO TNF+C+Z	2,9	ns
TRADD-RIPK1-DKO vs TRADD-RIPK1-DKO TNF+C+N	10	ns
TRADD-RIPK1-DKO vs TRADD-RIPK1-DKO TNF+C+Z+N	2,2	ns
Casp.8-KO vs Casp.8-KO TNF	12	ns
Casp.8-KO vs Casp.8-KO TNF+Z	9,2	ns
Casp.8-KO vs Casp.8-KO TNF+N	1,8	ns
Casp.8-KO vs Casp.8-KO TNF+Z+N	0,25	ns
Casp.8-KO vs Casp.8-KO TNF+C	38	***
Casp.8-KO vs Casp.8-KO TNF+C+Z	37	***
Casp.8-KO vs Casp.8-KO TNF+C+N	2,1	ns
Casp.8-KO vs Casp.8-KO TNF+C+Z+N	3,5	ns
EV vs EV TNF	14	ns
EV vs EV TNF+Z	5,3	ns
EV vs EV TNF+N	16	ns
EV vs EV TNF+Z+N	7,3	ns
EV vs EV TNF+C	77	***
EV vs EV TNF+C+Z	1,3	ns
EV vs EV TNF+C+N	66	***
EV vs EV TNF+C+Z+N	2,7	ns
CON TNF vs CON TNF+Z	-4,2	ns
CON TNF vs CON TNF+N	-3,3	ns
CON TNF vs CON TNF+Z+N	-2,2	ns
CON TNF vs CON TNF+C	70	***
CON TNF vs CON TNF+C+Z	75	***
CON TNF vs CON TNF+C+N	31	***
CON TNF vs CON TNF+C+Z+N	-1,0	ns
CON TNF vs FADD-KO TNF	84	***
CON TNF vs FADD-KO TNF+Z	87	***
CON TNF vs FADD-KO TNF+N	-2,1	ns
CON TNF vs FADD-KO TNF+Z+N	-5,3	ns
CON TNF vs FADD-KO TNF+C	69	***
CON TNF vs FADD-KO TNF+C+Z	72	***
CON TNF vs FADD-KO TNF+C+N	-1,9	ns
CON TNF vs FADD-KO TNF+C+Z+N	-3,4	ns
CON TNF vs TRADD-KO TNF	0,94	ns
CON TNF vs TRADD-KO TNF+Z	10	**
CON TNF vs TRADD-KO TNF+N	4,4	ns
CON TNF vs TRADD-KO TNF+Z+N	-2,1	ns
CON TNF vs TRADD-KO TNF+C	86	***
CON TNF vs TRADD-KO TNF+C+Z	72	***
CON TNF vs TRADD-KO TNF+C+N	66	***
CON TNF vs TRADD-KO TNF+C+Z+N	-0,76	ns
CON TNF vs RIPK1-KO TNF	-4,2	ns
CON TNF vs RIPK1-KO TNF+Z	-6,6	ns
CON TNF vs RIPK1-KO TNF+N	2,7	ns
CON TNF vs RIPK1-KO TNF+Z+N	-3,8	ns
CON TNF vs RIPK1-KO TNF+C	72	***
CON TNF vs RIPK1-KO TNF+C+Z	-3,0	ns
CON TNF vs RIPK1-KO TNF+C+N	64	***
CON TNF vs RIPK1-KO TNF+C+Z+N	-4,4	ns
CON TNF vs FADD-TRADD-DKO TNF	5,6	ns
CON TNF vs FADD-TRADD-DKO TNF+Z	9,9	ns
CON TNF vs FADD-TRADD-DKO TNF+N	4,4	ns
CON TNF vs FADD-TRADD-DKO TNF+Z+N	-3,4	ns
CON TNF vs FADD-TRADD-DKO TNF+C	88	***
CON TNF vs FADD-TRADD-DKO TNF+C+Z	83	***
CON TNF vs FADD-TRADD-DKO TNF+C+N	26	***
CON TNF vs FADD-TRADD-DKO TNF+C+Z+N	-0,0040	ns
CON TNF vs FADD-RIPK1-DKO TNF	-5,6	ns
CON TNF vs FADD-RIPK1-DKO TNF+Z	-6,1	ns
CON TNF vs FADD-RIPK1-DKO TNF+N	-0,42	ns
CON TNF vs FADD-RIPK1-DKO TNF+Z+N	-2,6	ns
CON TNF vs FADD-RIPK1-DKO TNF+C	0,36	ns

CON TNF vs FADD-RIPK1-DKO TNF+C+Z	2,4	ns
CON TNF vs FADD-RIPK1-DKO TNF+C+N	4,3	ns
CON TNF vs FADD-RIPK1-DKO TNF+C+Z+N	-3,7	ns
CON TNF vs TRADD-RIPK1-DKO TNF	-3,0	ns
CON TNF vs TRADD-RIPK1-DKO TNF+Z	-3,2	ns
CON TNF vs TRADD-RIPK1-DKO TNF+N	0,36	ns
CON TNF vs TRADD-RIPK1-DKO TNF+Z+N	2,0	ns
CON TNF vs TRADD-RIPK1-DKO TNF+C	6,8	ns
CON TNF vs TRADD-RIPK1-DKO TNF+C+Z	-2,9	ns
CON TNF vs TRADD-RIPK1-DKO TNF+C+N	4,6	ns
CON TNF vs TRADD-RIPK1-DKO TNF+C+Z+N	-3,6	ns
CON TNF vs Casp.8-KO TNF	5,7	ns
CON TNF vs Casp.8-KO TNF+Z	3,4	ns
CON TNF vs Casp.8-KO TNF+N	-4,0	ns
CON TNF vs Casp.8-KO TNF+Z+N	-5,6	ns
CON TNF vs Casp.8-KO TNF+C	32	***
CON TNF vs Casp.8-KO TNF+C+Z	31	***
CON TNF vs Casp.8-KO TNF+C+N	-3,8	ns
CON TNF vs Casp.8-KO TNF+C+Z+N	-2,3	ns
CON TNF vs EV TNF	7,7	ns
CON TNF vs EV TNF+Z	-0,49	ns
CON TNF vs EV TNF+N	9,8	ns
CON TNF vs EV TNF+Z+N	1,5	ns
CON TNF vs EV TNF+C	71	***
CON TNF vs EV TNF+C+Z	-4,5	ns
CON TNF vs EV TNF+C+N	60	***
CON TNF vs EV TNF+C+Z+N	-3,1	ns
CON TNF+Z vs CON TNF+N	0,89	ns
CON TNF+Z vs CON TNF+Z+N	1,9	ns
CON TNF+Z vs CON TNF+C	74	***
CON TNF+Z vs CON TNF+C+Z	79	***
CON TNF+Z vs CON TNF+C+N	35	***
CON TNF+Z vs CON TNF+C+Z+N	3,2	ns
CON TNF+Z vs FADD-KO TNF	88	***
CON TNF+Z vs FADD-KO TNF+Z	91	***
CON TNF+Z vs FADD-KO TNF+N	2,1	ns
CON TNF+Z vs FADD-KO TNF+Z+N	-1,1	ns
CON TNF+Z vs FADD-KO TNF+C	73	***
CON TNF+Z vs FADD-KO TNF+C+Z	76	***
CON TNF+Z vs FADD-KO TNF+C+N	2,2	ns
CON TNF+Z vs FADD-KO TNF+C+Z+N	0,73	ns
CON TNF+Z vs TRADD-KO TNF	5,1	ns
CON TNF+Z vs TRADD-KO TNF+Z	14	***
CON TNF+Z vs TRADD-KO TNF+N	8,6	ns
CON TNF+Z vs TRADD-KO TNF+Z+N	2,0	ns
CON TNF+Z vs TRADD-KO TNF+C	90	***
CON TNF+Z vs TRADD-KO TNF+C+Z	76	***
CON TNF+Z vs TRADD-KO TNF+C+N	70	***
CON TNF+Z vs TRADD-KO TNF+C+Z+N	3,4	ns
CON TNF+Z vs RIPK1-KO TNF	-0,042	ns
CON TNF+Z vs RIPK1-KO TNF+Z	-2,4	ns
CON TNF+Z vs RIPK1-KO TNF+N	6,8	ns
CON TNF+Z vs RIPK1-KO TNF+Z+N	0,39	ns
CON TNF+Z vs RIPK1-KO TNF+C	76	***
CON TNF+Z vs RIPK1-KO TNF+C+Z	1,2	ns
CON TNF+Z vs RIPK1-KO TNF+C+N	68	***
CON TNF+Z vs RIPK1-KO TNF+C+Z+N	-0,20	ns
CON TNF+Z vs FADD-TRADD-DKO TNF	9,7	ns
CON TNF+Z vs FADD-TRADD-DKO TNF+Z	14	**
CON TNF+Z vs FADD-TRADD-DKO TNF+N	8,5	ns
CON TNF+Z vs FADD-TRADD-DKO TNF+Z+N	0,71	ns
CON TNF+Z vs FADD-TRADD-DKO TNF+C	92	***
CON TNF+Z vs FADD-TRADD-DKO TNF+C+Z	88	***
CON TNF+Z vs FADD-TRADD-DKO TNF+C+N	30	***
CON TNF+Z vs FADD-TRADD-DKO TNF+C+Z+N	4,2	ns
CON TNF+Z vs FADD-RIPK1-DKO TNF	-1,4	ns

CON TNF+Z vs FADD-RIPK1-DKO TNF+Z	-2,0	ns
CON TNF+Z vs FADD-RIPK1-DKO TNF+N	3,7	ns
CON TNF+Z vs FADD-RIPK1-DKO TNF+Z+N	1,5	ns
CON TNF+Z vs FADD-RIPK1-DKO TNF+C	4,5	ns
CON TNF+Z vs FADD-RIPK1-DKO TNF+C+Z	6,5	ns
CON TNF+Z vs FADD-RIPK1-DKO TNF+C+N	8,4	ns
CON TNF+Z vs FADD-RIPK1-DKO TNF+C+Z+N	0,42	ns
CON TNF+Z vs TRADD-RIPK1-DKO TNF	1,2	ns
CON TNF+Z vs TRADD-RIPK1-DKO TNF+Z	0,94	ns
CON TNF+Z vs TRADD-RIPK1-DKO TNF+N	4,5	ns
CON TNF+Z vs TRADD-RIPK1-DKO TNF+Z+N	6,2	ns
CON TNF+Z vs TRADD-RIPK1-DKO TNF+C	11	**
CON TNF+Z vs TRADD-RIPK1-DKO TNF+C+Z	1,2	ns
CON TNF+Z vs TRADD-RIPK1-DKO TNF+C+N	8,8	ns
CON TNF+Z vs TRADD-RIPK1-DKO TNF+C+Z+N	0,53	ns
CON TNF+Z vs Casp.8-KO TNF	9,8	ns
CON TNF+Z vs Casp.8-KO TNF+Z	7,6	ns
CON TNF+Z vs Casp.8-KO TNF+N	0,17	ns
CON TNF+Z vs Casp.8-KO TNF+Z+N	-1,4	ns
CON TNF+Z vs Casp.8-KO TNF+C	36	***
CON TNF+Z vs Casp.8-KO TNF+C+Z	35	***
CON TNF+Z vs Casp.8-KO TNF+C+N	0,40	ns
CON TNF+Z vs Casp.8-KO TNF+C+Z+N	1,9	ns
CON TNF+Z vs EV TNF	12	ns
CON TNF+Z vs EV TNF+Z	3,7	ns
CON TNF+Z vs EV TNF+N	14	ns
CON TNF+Z vs EV TNF+Z+N	5,7	ns
CON TNF+Z vs EV TNF+C	76	***
CON TNF+Z vs EV TNF+C+Z	-0,37	ns
CON TNF+Z vs EV TNF+C+N	64	***
CON TNF+Z vs EV TNF+C+Z+N	1,0	ns
CON TNF+N vs CON TNF+Z+N	1,0	ns
CON TNF+N vs CON TNF+C	74	***
CON TNF+N vs CON TNF+C+Z	78	***
CON TNF+N vs CON TNF+C+N	34	***
CON TNF+N vs CON TNF+C+Z+N	2,3	ns
CON TNF+N vs FADD-KO TNF	87	***
CON TNF+N vs FADD-KO TNF+Z	90	***
CON TNF+N vs FADD-KO TNF+N	1,2	ns
CON TNF+N vs FADD-KO TNF+Z+N	-2,0	ns
CON TNF+N vs FADD-KO TNF+C	72	***
CON TNF+N vs FADD-KO TNF+C+Z	75	***
CON TNF+N vs FADD-KO TNF+C+N	1,3	ns
CON TNF+N vs FADD-KO TNF+C+Z+N	-0,15	ns
CON TNF+N vs TRADD-KO TNF	4,2	ns
CON TNF+N vs TRADD-KO TNF+Z	14	***
CON TNF+N vs TRADD-KO TNF+N	7,7	ns
CON TNF+N vs TRADD-KO TNF+Z+N	1,1	ns
CON TNF+N vs TRADD-KO TNF+C	89	***
CON TNF+N vs TRADD-KO TNF+C+Z	75	***
CON TNF+N vs TRADD-KO TNF+C+N	69	***
CON TNF+N vs TRADD-KO TNF+C+Z+N	2,5	ns
CON TNF+N vs RIPK1-KO TNF	-0,93	ns
CON TNF+N vs RIPK1-KO TNF+Z	-3,3	ns
CON TNF+N vs RIPK1-KO TNF+N	5,9	ns
CON TNF+N vs RIPK1-KO TNF+Z+N	-0,49	ns
CON TNF+N vs RIPK1-KO TNF+C	76	***
CON TNF+N vs RIPK1-KO TNF+C+Z	0,31	ns
CON TNF+N vs RIPK1-KO TNF+C+N	67	***
CON TNF+N vs RIPK1-KO TNF+C+Z+N	-1,1	ns
CON TNF+N vs FADD-TRADD-DKO TNF	8,8	ns
CON TNF+N vs FADD-TRADD-DKO TNF+Z	13	*
CON TNF+N vs FADD-TRADD-DKO TNF+N	7,6	ns
CON TNF+N vs FADD-TRADD-DKO TNF+Z+N	-0,18	ns
CON TNF+N vs FADD-TRADD-DKO TNF+C	91	***
CON TNF+N vs FADD-TRADD-DKO TNF+C+Z	87	***

CON TNF+N vs FADD-TRADD-DKO TNF+C+N	29	***
CON TNF+N vs FADD-TRADD-DKO TNF+C+Z+N	3,3	ns
CON TNF+N vs FADD-RIPK1-DKO TNF	-2,3	ns
CON TNF+N vs FADD-RIPK1-DKO TNF+Z	-2,8	ns
CON TNF+N vs FADD-RIPK1-DKO TNF+N	2,8	ns
CON TNF+N vs FADD-RIPK1-DKO TNF+Z+N	0,65	ns
CON TNF+N vs FADD-RIPK1-DKO TNF+C	3,6	ns
CON TNF+N vs FADD-RIPK1-DKO TNF+C+Z	5,6	ns
CON TNF+N vs FADD-RIPK1-DKO TNF+C+N	7,5	ns
CON TNF+N vs FADD-RIPK1-DKO TNF+C+Z+N	-0,46	ns
CON TNF+N vs TRADD-RIPK1-DKO TNF	0,29	ns
CON TNF+N vs TRADD-RIPK1-DKO TNF+Z	0,059	ns
CON TNF+N vs TRADD-RIPK1-DKO TNF+N	3,6	ns
CON TNF+N vs TRADD-RIPK1-DKO TNF+Z+N	5,3	ns
CON TNF+N vs TRADD-RIPK1-DKO TNF+C	10	ns
CON TNF+N vs TRADD-RIPK1-DKO TNF+C+Z	0,32	ns
CON TNF+N vs TRADD-RIPK1-DKO TNF+C+N	7,9	ns
CON TNF+N vs TRADD-RIPK1-DKO TNF+C+Z+N	-0,35	ns
CON TNF+N vs Casp.8-KO TNF	8,9	ns
CON TNF+N vs Casp.8-KO TNF+Z	6,7	ns
CON TNF+N vs Casp.8-KO TNF+N	-0,72	ns
CON TNF+N vs Casp.8-KO TNF+Z+N	-2,3	ns
CON TNF+N vs Casp.8-KO TNF+C	35	***
CON TNF+N vs Casp.8-KO TNF+C+Z	34	***
CON TNF+N vs Casp.8-KO TNF+C+N	-0,49	ns
CON TNF+N vs Casp.8-KO TNF+C+Z+N	0,98	ns
CON TNF+N vs EV TNF	11	ns
CON TNF+N vs EV TNF+Z	2,8	ns
CON TNF+N vs EV TNF+N	13	ns
CON TNF+N vs EV TNF+Z+N	4,8	ns
CON TNF+N vs EV TNF+C	75	***
CON TNF+N vs EV TNF+C+Z	-1,3	ns
CON TNF+N vs EV TNF+C+N	63	***
CON TNF+N vs EV TNF+C+Z+N	0,16	ns
CON TNF+Z+N vs CON TNF+C	73	***
CON TNF+Z+N vs CON TNF+C+Z	77	***
CON TNF+Z+N vs CON TNF+C+N	33	***
CON TNF+Z+N vs CON TNF+C+Z+N	1,2	ns
CON TNF+Z+N vs FADD-KO TNF	86	***
CON TNF+Z+N vs FADD-KO TNF+Z	89	***
CON TNF+Z+N vs FADD-KO TNF+N	0,16	ns
CON TNF+Z+N vs FADD-KO TNF+Z+N	-3,0	ns
CON TNF+Z+N vs FADD-KO TNF+C	71	***
CON TNF+Z+N vs FADD-KO TNF+C+Z	74	***
CON TNF+Z+N vs FADD-KO TNF+C+N	0,28	ns
CON TNF+Z+N vs FADD-KO TNF+C+Z+N	-1,2	ns
CON TNF+Z+N vs TRADD-KO TNF	3,2	ns
CON TNF+Z+N vs TRADD-KO TNF+Z	13	***
CON TNF+Z+N vs TRADD-KO TNF+N	6,6	ns
CON TNF+Z+N vs TRADD-KO TNF+Z+N	0,082	ns
CON TNF+Z+N vs TRADD-KO TNF+C	88	***
CON TNF+Z+N vs TRADD-KO TNF+C+Z	74	***
CON TNF+Z+N vs TRADD-KO TNF+C+N	68	***
CON TNF+Z+N vs TRADD-KO TNF+C+Z+N	1,5	ns
CON TNF+Z+N vs RIPK1-KO TNF	-2,0	ns
CON TNF+Z+N vs RIPK1-KO TNF+Z	-4,3	ns
CON TNF+Z+N vs RIPK1-KO TNF+N	4,9	ns
CON TNF+Z+N vs RIPK1-KO TNF+Z+N	-1,5	ns
CON TNF+Z+N vs RIPK1-KO TNF+C	75	***
CON TNF+Z+N vs RIPK1-KO TNF+C+Z	-0,73	ns
CON TNF+Z+N vs RIPK1-KO TNF+C+N	66	***
CON TNF+Z+N vs RIPK1-KO TNF+C+Z+N	-2,1	ns
CON TNF+Z+N vs FADD-TRADD-DKO TNF	7,8	ns
CON TNF+Z+N vs FADD-TRADD-DKO TNF+Z	12	ns
CON TNF+Z+N vs FADD-TRADD-DKO TNF+N	6,6	ns
CON TNF+Z+N vs FADD-TRADD-DKO TNF+Z+N	-1,2	ns

CON TNF+Z+N vs FADD-TRADD-DKO TNF+C	90	***
CON TNF+Z+N vs FADD-TRADD-DKO TNF+C+Z	86	***
CON TNF+Z+N vs FADD-TRADD-DKO TNF+C+N	28	***
CON TNF+Z+N vs FADD-TRADD-DKO TNF+C+Z+N	2,2	ns
CON TNF+Z+N vs FADD-RIPK1-DKO TNF	-3,3	ns
CON TNF+Z+N vs FADD-RIPK1-DKO TNF+Z	-3,9	ns
CON TNF+Z+N vs FADD-RIPK1-DKO TNF+N	1,8	ns
CON TNF+Z+N vs FADD-RIPK1-DKO TNF+Z+N	-0,40	ns
CON TNF+Z+N vs FADD-RIPK1-DKO TNF+C	2,6	ns
CON TNF+Z+N vs FADD-RIPK1-DKO TNF+C+Z	4,6	ns
CON TNF+Z+N vs FADD-RIPK1-DKO TNF+C+N	6,5	ns
CON TNF+Z+N vs FADD-RIPK1-DKO TNF+C+Z+N	-1,5	ns
CON TNF+Z+N vs TRADD-RIPK1-DKO TNF	-0,76	ns
CON TNF+Z+N vs TRADD-RIPK1-DKO TNF+Z	-0,99	ns
CON TNF+Z+N vs TRADD-RIPK1-DKO TNF+N	2,6	ns
CON TNF+Z+N vs TRADD-RIPK1-DKO TNF+Z+N	4,2	ns
CON TNF+Z+N vs TRADD-RIPK1-DKO TNF+C	9,1	ns
CON TNF+Z+N vs TRADD-RIPK1-DKO TNF+C+Z	-0,72	ns
CON TNF+Z+N vs TRADD-RIPK1-DKO TNF+C+N	6,8	ns
CON TNF+Z+N vs TRADD-RIPK1-DKO TNF+C+Z+N	-1,4	ns
CON TNF+Z+N vs Casp.8-KO TNF	7,9	ns
CON TNF+Z+N vs Casp.8-KO TNF+Z	5,6	ns
CON TNF+Z+N vs Casp.8-KO TNF+N	-1,8	ns
CON TNF+Z+N vs Casp.8-KO TNF+Z+N	-3,3	ns
CON TNF+Z+N vs Casp.8-KO TNF+C	34	***
CON TNF+Z+N vs Casp.8-KO TNF+C+Z	33	***
CON TNF+Z+N vs Casp.8-KO TNF+C+N	-1,5	ns
CON TNF+Z+N vs Casp.8-KO TNF+C+Z+N	-0,067	ns
CON TNF+Z+N vs EV TNF	10	ns
CON TNF+Z+N vs EV TNF+Z	1,7	ns
CON TNF+Z+N vs EV TNF+N	12	ns
CON TNF+Z+N vs EV TNF+Z+N	3,7	ns
CON TNF+Z+N vs EV TNF+C	74	***
CON TNF+Z+N vs EV TNF+C+Z	-2,3	ns
CON TNF+Z+N vs EV TNF+C+N	62	***
CON TNF+Z+N vs EV TNF+C+Z+N	-0,89	ns
CON TNF+C vs CON TNF+C+Z	4,6	ns
CON TNF+C vs CON TNF+C+N	-40	***
CON TNF+C vs CON TNF+C+Z+N	-71	***
CON TNF+C vs FADD-KO TNF	14	ns
CON TNF+C vs FADD-KO TNF+Z	17	ns
CON TNF+C vs FADD-KO TNF+N	-72	***
CON TNF+C vs FADD-KO TNF+Z+N	-76	***
CON TNF+C vs FADD-KO TNF+C	-1,3	ns
CON TNF+C vs FADD-KO TNF+C+Z	1,4	ns
CON TNF+C vs FADD-KO TNF+C+N	-72	***
CON TNF+C vs FADD-KO TNF+C+Z+N	-74	***
CON TNF+C vs TRADD-KO TNF	-69	***
CON TNF+C vs TRADD-KO TNF+Z	-60	***
CON TNF+C vs TRADD-KO TNF+N	-66	***
CON TNF+C vs TRADD-KO TNF+Z+N	-72	***
CON TNF+C vs TRADD-KO TNF+C	16	ns
CON TNF+C vs TRADD-KO TNF+C+Z	1,6	ns
CON TNF+C vs TRADD-KO TNF+C+N	-4,4	ns
CON TNF+C vs TRADD-KO TNF+C+Z+N	-71	***
CON TNF+C vs RIPK1-KO TNF	-74	***
CON TNF+C vs RIPK1-KO TNF+Z	-77	***
CON TNF+C vs RIPK1-KO TNF+N	-68	***
CON TNF+C vs RIPK1-KO TNF+Z+N	-74	***
CON TNF+C vs RIPK1-KO TNF+C	2,0	ns
CON TNF+C vs RIPK1-KO TNF+C+Z	-73	***
CON TNF+C vs RIPK1-KO TNF+C+N	-6,4	ns
CON TNF+C vs RIPK1-KO TNF+C+Z+N	-75	***
CON TNF+C vs FADD-TRADD-DKO TNF	-65	***
CON TNF+C vs FADD-TRADD-DKO TNF+Z	-60	***
CON TNF+C vs FADD-TRADD-DKO TNF+N	-66	***

CON TNF+C vs FADD-TRADD-DKO TNF+Z+N	-74	***
CON TNF+C vs FADD-TRADD-DKO TNF+C	18	ns
CON TNF+C vs FADD-TRADD-DKO TNF+C+Z	13	ns
CON TNF+C vs FADD-TRADD-DKO TNF+C+N	-44	***
CON TNF+C vs FADD-TRADD-DKO TNF+C+Z+N	-70	***
CON TNF+C vs FADD-RIPK1-DKO TNF	-76	***
CON TNF+C vs FADD-RIPK1-DKO TNF+Z	-76	***
CON TNF+C vs FADD-RIPK1-DKO TNF+N	-71	***
CON TNF+C vs FADD-RIPK1-DKO TNF+Z+N	-73	***
CON TNF+C vs FADD-RIPK1-DKO TNF+C	-70	***
CON TNF+C vs FADD-RIPK1-DKO TNF+C+Z	-68	***
CON TNF+C vs FADD-RIPK1-DKO TNF+C+N	-66	***
CON TNF+C vs FADD-RIPK1-DKO TNF+C+Z+N	-74	***
CON TNF+C vs TRADD-RIPK1-DKO TNF	-73	***
CON TNF+C vs TRADD-RIPK1-DKO TNF+Z	-74	***
CON TNF+C vs TRADD-RIPK1-DKO TNF+N	-70	***
CON TNF+C vs TRADD-RIPK1-DKO TNF+Z+N	-68	***
CON TNF+C vs TRADD-RIPK1-DKO TNF+C	-63	***
CON TNF+C vs TRADD-RIPK1-DKO TNF+C+Z	-73	***
CON TNF+C vs TRADD-RIPK1-DKO TNF+C+N	-66	***
CON TNF+C vs TRADD-RIPK1-DKO TNF+C+Z+N	-74	***
CON TNF+C vs Casp.8-KO TNF	-65	***
CON TNF+C vs Casp.8-KO TNF+Z	-67	***
CON TNF+C vs Casp.8-KO TNF+N	-74	***
CON TNF+C vs Casp.8-KO TNF+Z+N	-76	***
CON TNF+C vs Casp.8-KO TNF+C	-38	***
CON TNF+C vs Casp.8-KO TNF+C+Z	-40	***
CON TNF+C vs Casp.8-KO TNF+C+N	-74	***
CON TNF+C vs Casp.8-KO TNF+C+Z+N	-73	***
CON TNF+C vs EV TNF	-63	***
CON TNF+C vs EV TNF+Z	-71	***
CON TNF+C vs EV TNF+N	-60	***
CON TNF+C vs EV TNF+Z+N	-69	***
CON TNF+C vs EV TNF+C	1,2	ns
CON TNF+C vs EV TNF+C+Z	-75	***
CON TNF+C vs EV TNF+C+N	-10	ns
CON TNF+C vs EV TNF+C+Z+N	-73	***
CON TNF+C+Z vs CON TNF+C+N	-44	***
CON TNF+C+Z vs CON TNF+C+Z+N	-76	***
CON TNF+C+Z vs FADD-KO TNF	9,1	ns
CON TNF+C+Z vs FADD-KO TNF+Z	12	ns
CON TNF+C+Z vs FADD-KO TNF+N	-77	***
CON TNF+C+Z vs FADD-KO TNF+Z+N	-80	***
CON TNF+C+Z vs FADD-KO TNF+C	-6,0	ns
CON TNF+C+Z vs FADD-KO TNF+C+Z	-3,3	ns
CON TNF+C+Z vs FADD-KO TNF+C+N	-77	***
CON TNF+C+Z vs FADD-KO TNF+C+Z+N	-78	***
CON TNF+C+Z vs TRADD-KO TNF	-74	***
CON TNF+C+Z vs TRADD-KO TNF+Z	-65	***
CON TNF+C+Z vs TRADD-KO TNF+N	-71	***
CON TNF+C+Z vs TRADD-KO TNF+Z+N	-77	***
CON TNF+C+Z vs TRADD-KO TNF+C	11	ns
CON TNF+C+Z vs TRADD-KO TNF+C+Z	-3,0	ns
CON TNF+C+Z vs TRADD-KO TNF+C+N	-9,0	ns
CON TNF+C+Z vs TRADD-KO TNF+C+Z+N	-76	***
CON TNF+C+Z vs RIPK1-KO TNF	-79	***
CON TNF+C+Z vs RIPK1-KO TNF+Z	-81	***
CON TNF+C+Z vs RIPK1-KO TNF+N	-72	***
CON TNF+C+Z vs RIPK1-KO TNF+Z+N	-79	***
CON TNF+C+Z vs RIPK1-KO TNF+C	-2,6	ns
CON TNF+C+Z vs RIPK1-KO TNF+C+Z	-78	***
CON TNF+C+Z vs RIPK1-KO TNF+C+N	-11	ns
CON TNF+C+Z vs RIPK1-KO TNF+C+Z+N	-79	***
CON TNF+C+Z vs FADD-TRADD-DKO TNF	-69	***
CON TNF+C+Z vs FADD-TRADD-DKO TNF+Z	-65	***
CON TNF+C+Z vs FADD-TRADD-DKO TNF+N	-71	***

CON TNF+C+Z vs FADD-TRADD-DKO TNF+Z+N	-78	***
CON TNF+C+Z vs FADD-TRADD-DKO TNF+C	13	ns
CON TNF+C+Z vs FADD-TRADD-DKO TNF+C+Z	8,5	ns
CON TNF+C+Z vs FADD-TRADD-DKO TNF+C+N	-49	***
CON TNF+C+Z vs FADD-TRADD-DKO TNF+C+Z+N	-75	***
CON TNF+C+Z vs FADD-RIPK1-DKO TNF	-80	***
CON TNF+C+Z vs FADD-RIPK1-DKO TNF+Z	-81	***
CON TNF+C+Z vs FADD-RIPK1-DKO TNF+N	-75	***
CON TNF+C+Z vs FADD-RIPK1-DKO TNF+Z+N	-78	***
CON TNF+C+Z vs FADD-RIPK1-DKO TNF+C	-75	***
CON TNF+C+Z vs FADD-RIPK1-DKO TNF+C+Z	-73	***
CON TNF+C+Z vs FADD-RIPK1-DKO TNF+C+N	-71	***
CON TNF+C+Z vs FADD-RIPK1-DKO TNF+C+Z+N	-79	***
CON TNF+C+Z vs TRADD-RIPK1-DKO TNF	-78	***
CON TNF+C+Z vs TRADD-RIPK1-DKO TNF+Z	-78	***
CON TNF+C+Z vs TRADD-RIPK1-DKO TNF+N	-75	***
CON TNF+C+Z vs TRADD-RIPK1-DKO TNF+Z+N	-73	***
CON TNF+C+Z vs TRADD-RIPK1-DKO TNF+C	-68	***
CON TNF+C+Z vs TRADD-RIPK1-DKO TNF+C+Z	-78	***
CON TNF+C+Z vs TRADD-RIPK1-DKO TNF+C+N	-70	***
CON TNF+C+Z vs TRADD-RIPK1-DKO TNF+C+Z+N	-79	***
CON TNF+C+Z vs Casp.8-KO TNF	-69	***
CON TNF+C+Z vs Casp.8-KO TNF+Z	-72	***
CON TNF+C+Z vs Casp.8-KO TNF+N	-79	***
CON TNF+C+Z vs Casp.8-KO TNF+Z+N	-81	***
CON TNF+C+Z vs Casp.8-KO TNF+C	-43	***
CON TNF+C+Z vs Casp.8-KO TNF+C+Z	-44	***
CON TNF+C+Z vs Casp.8-KO TNF+C+N	-79	***
CON TNF+C+Z vs Casp.8-KO TNF+C+Z+N	-77	***
CON TNF+C+Z vs EV TNF	-67	***
CON TNF+C+Z vs EV TNF+Z	-75	***
CON TNF+C+Z vs EV TNF+N	-65	***
CON TNF+C+Z vs EV TNF+Z+N	-73	***
CON TNF+C+Z vs EV TNF+C	-3,5	ns
CON TNF+C+Z vs EV TNF+C+Z	-79	***
CON TNF+C+Z vs EV TNF+C+N	-15	ns
CON TNF+C+Z vs EV TNF+C+Z+N	-78	***
CON TNF+C+N vs CON TNF+C+Z+N	-32	***
CON TNF+C+N vs FADD-KO TNF	54	***
CON TNF+C+N vs FADD-KO TNF+Z	56	***
CON TNF+C+N vs FADD-KO TNF+N	-33	***
CON TNF+C+N vs FADD-KO TNF+Z+N	-36	***
CON TNF+C+N vs FADD-KO TNF+C	38	***
CON TNF+C+N vs FADD-KO TNF+C+Z	41	***
CON TNF+C+N vs FADD-KO TNF+C+N	-32	***
CON TNF+C+N vs FADD-KO TNF+C+Z+N	-34	***
CON TNF+C+N vs TRADD-KO TNF	-30	***
CON TNF+C+N vs TRADD-KO TNF+Z	-20	***
CON TNF+C+N vs TRADD-KO TNF+N	-26	***
CON TNF+C+N vs TRADD-KO TNF+Z+N	-33	***
CON TNF+C+N vs TRADD-KO TNF+C	56	***
CON TNF+C+N vs TRADD-KO TNF+C+Z	41	***
CON TNF+C+N vs TRADD-KO TNF+C+N	35	***
CON TNF+C+N vs TRADD-KO TNF+C+Z+N	-31	***
CON TNF+C+N vs RIPK1-KO TNF	-35	***
CON TNF+C+N vs RIPK1-KO TNF+Z	-37	***
CON TNF+C+N vs RIPK1-KO TNF+N	-28	***
CON TNF+C+N vs RIPK1-KO TNF+Z+N	-34	***
CON TNF+C+N vs RIPK1-KO TNF+C	42	***
CON TNF+C+N vs RIPK1-KO TNF+C+Z	-33	***
CON TNF+C+N vs RIPK1-KO TNF+C+N	33	***
CON TNF+C+N vs RIPK1-KO TNF+C+Z+N	-35	***
CON TNF+C+N vs FADD-TRADD-DKO TNF	-25	***
CON TNF+C+N vs FADD-TRADD-DKO TNF+Z	-21	***
CON TNF+C+N vs FADD-TRADD-DKO TNF+N	-26	***
CON TNF+C+N vs FADD-TRADD-DKO TNF+Z+N	-34	***

CON TNF+C+N vs FADD-TRADD-DKO TNF+C	58	***
CON TNF+C+N vs FADD-TRADD-DKO TNF+C+Z	53	***
CON TNF+C+N vs FADD-TRADD-DKO TNF+C+N	-4,7	Ns
CON TNF+C+N vs FADD-TRADD-DKO TNF+C+Z+N	-31	***
CON TNF+C+N vs FADD-RIPK1-DKO TNF	-36	***
CON TNF+C+N vs FADD-RIPK1-DKO TNF+Z	-37	***
CON TNF+C+N vs FADD-RIPK1-DKO TNF+N	-31	***
CON TNF+C+N vs FADD-RIPK1-DKO TNF+Z+N	-33	***
CON TNF+C+N vs FADD-RIPK1-DKO TNF+C	-30	***
CON TNF+C+N vs FADD-RIPK1-DKO TNF+C+Z	-28	***
CON TNF+C+N vs FADD-RIPK1-DKO TNF+C+N	-26	***
CON TNF+C+N vs FADD-RIPK1-DKO TNF+C+Z+N	-34	***
CON TNF+C+N vs TRADD-RIPK1-DKO TNF	-33	***
CON TNF+C+N vs TRADD-RIPK1-DKO TNF+Z	-34	***
CON TNF+C+N vs TRADD-RIPK1-DKO TNF+N	-30	***
CON TNF+C+N vs TRADD-RIPK1-DKO TNF+Z+N	-29	***
CON TNF+C+N vs TRADD-RIPK1-DKO TNF+C	-24	***
CON TNF+C+N vs TRADD-RIPK1-DKO TNF+C+Z	-33	***
CON TNF+C+N vs TRADD-RIPK1-DKO TNF+C+N	-26	***
CON TNF+C+N vs TRADD-RIPK1-DKO TNF+C+Z+N	-34	***
CON TNF+C+N vs Casp.8-KO TNF	-25	***
CON TNF+C+N vs Casp.8-KO TNF+Z	-27	***
CON TNF+C+N vs Casp.8-KO TNF+N	-35	***
CON TNF+C+N vs Casp.8-KO TNF+Z+N	-36	***
CON TNF+C+N vs Casp.8-KO TNF+C	1,5	ns
CON TNF+C+N vs Casp.8-KO TNF+C+Z	0,25	ns
CON TNF+C+N vs Casp.8-KO TNF+C+N	-34	***
CON TNF+C+N vs Casp.8-KO TNF+C+Z+N	-33	***
CON TNF+C+N vs EV TNF	-23	***
CON TNF+C+N vs EV TNF+Z	-31	***
CON TNF+C+N vs EV TNF+N	-21	ns
CON TNF+C+N vs EV TNF+Z+N	-29	***
CON TNF+C+N vs EV TNF+C	41	***
CON TNF+C+N vs EV TNF+C+Z	-35	***
CON TNF+C+N vs EV TNF+C+N	30	***
CON TNF+C+N vs EV TNF+C+Z+N	-34	***
CON TNF+C+Z+N vs FADD-KO TNF	85	***
CON TNF+C+Z+N vs FADD-KO TNF+Z	88	***
CON TNF+C+Z+N vs FADD-KO TNF+N	-1,1	ns
CON TNF+C+Z+N vs FADD-KO TNF+Z+N	-4,3	ns
CON TNF+C+Z+N vs FADD-KO TNF+C	70	***
CON TNF+C+Z+N vs FADD-KO TNF+C+Z	73	***
CON TNF+C+Z+N vs FADD-KO TNF+C+N	-0,95	ns
CON TNF+C+Z+N vs FADD-KO TNF+C+Z+N	-2,4	ns
CON TNF+C+Z+N vs TRADD-KO TNF	1,9	ns
CON TNF+C+Z+N vs TRADD-KO TNF+Z	11	*
CON TNF+C+Z+N vs TRADD-KO TNF+N	5,4	ns
CON TNF+C+Z+N vs TRADD-KO TNF+Z+N	-1,1	ns
CON TNF+C+Z+N vs TRADD-KO TNF+C	87	***
CON TNF+C+Z+N vs TRADD-KO TNF+C+Z	73	***
CON TNF+C+Z+N vs TRADD-KO TNF+C+N	67	***
CON TNF+C+Z+N vs TRADD-KO TNF+C+Z+N	0,23	ns
CON TNF+C+Z+N vs RIPK1-KO TNF	-3,2	ns
CON TNF+C+Z+N vs RIPK1-KO TNF+Z	-5,6	ns
CON TNF+C+Z+N vs RIPK1-KO TNF+N	3,7	ns
CON TNF+C+Z+N vs RIPK1-KO TNF+Z+N	-2,8	ns
CON TNF+C+Z+N vs RIPK1-KO TNF+C	73	***
CON TNF+C+Z+N vs RIPK1-KO TNF+C+Z	-2,0	ns
CON TNF+C+Z+N vs RIPK1-KO TNF+C+N	65	***
CON TNF+C+Z+N vs RIPK1-KO TNF+C+Z+N	-3,4	ns
CON TNF+C+Z+N vs FADD-TRADD-DKO TNF	6,5	ns
CON TNF+C+Z+N vs FADD-TRADD-DKO TNF+Z	11	ns
CON TNF+C+Z+N vs FADD-TRADD-DKO TNF+N	5,4	ns
CON TNF+C+Z+N vs FADD-TRADD-DKO TNF+Z+N	-2,5	ns
CON TNF+C+Z+N vs FADD-TRADD-DKO TNF+C	89	***
CON TNF+C+Z+N vs FADD-TRADD-DKO TNF+C+Z	84	***

CON TNF+C+Z+N vs FADD-TRADD-DKO TNF+C+N	27	***
CON TNF+C+Z+N vs FADD-TRADD-DKO TNF+C+Z+N	0,99	ns
CON TNF+C+Z+N vs FADD-RIPK1-DKO TNF	-4,6	ns
CON TNF+C+Z+N vs FADD-RIPK1-DKO TNF+Z	-5,1	ns
CON TNF+C+Z+N vs FADD-RIPK1-DKO TNF+N	0,57	ns
CON TNF+C+Z+N vs FADD-RIPK1-DKO TNF+Z+N	-1,6	ns
CON TNF+C+Z+N vs FADD-RIPK1-DKO TNF+C	1,4	ns
CON TNF+C+Z+N vs FADD-RIPK1-DKO TNF+C+Z	3,4	ns
CON TNF+C+Z+N vs FADD-RIPK1-DKO TNF+C+N	5,3	ns
CON TNF+C+Z+N vs FADD-RIPK1-DKO TNF+C+Z+N	-2,7	ns
CON TNF+C+Z+N vs TRADD-RIPK1-DKO TNF	-2,0	ns
CON TNF+C+Z+N vs TRADD-RIPK1-DKO TNF+Z	-2,2	ns
CON TNF+C+Z+N vs TRADD-RIPK1-DKO TNF+N	1,4	ns
CON TNF+C+Z+N vs TRADD-RIPK1-DKO TNF+Z+N	3,0	ns
CON TNF+C+Z+N vs TRADD-RIPK1-DKO TNF+C	7,8	ns
CON TNF+C+Z+N vs TRADD-RIPK1-DKO TNF+C+Z	-2,0	ns
CON TNF+C+Z+N vs TRADD-RIPK1-DKO TNF+C+N	5,6	ns
CON TNF+C+Z+N vs TRADD-RIPK1-DKO TNF+C+Z+N	-2,6	ns
CON TNF+C+Z+N vs Casp.8-KO TNF	6,7	ns
CON TNF+C+Z+N vs Casp.8-KO TNF+Z	4,4	ns
CON TNF+C+Z+N vs Casp.8-KO TNF+N	-3,0	ns
CON TNF+C+Z+N vs Casp.8-KO TNF+Z+N	-4,6	ns
CON TNF+C+Z+N vs Casp.8-KO TNF+C	33	***
CON TNF+C+Z+N vs Casp.8-KO TNF+C+Z	32	***
CON TNF+C+Z+N vs Casp.8-KO TNF+C+N	-2,8	ns
CON TNF+C+Z+N vs Casp.8-KO TNF+C+Z+N	-1,3	ns
CON TNF+C+Z+N vs EV TNF	8,7	ns
CON TNF+C+Z+N vs EV TNF+Z	0,51	ns
CON TNF+C+Z+N vs EV TNF+N	11	ns
CON TNF+C+Z+N vs EV TNF+Z+N	2,5	ns
CON TNF+C+Z+N vs EV TNF+C	72	***
CON TNF+C+Z+N vs EV TNF+C+Z	-3,5	ns
CON TNF+C+Z+N vs EV TNF+C+N	61	***
CON TNF+C+Z+N vs EV TNF+C+Z+N	-2,1	ns
FADD-KO TNF vs FADD-KO TNF+Z	2,9	ns
FADD-KO TNF vs FADD-KO TNF+N	-86	***
FADD-KO TNF vs FADD-KO TNF+Z+N	-89	***
FADD-KO TNF vs FADD-KO TNF+C	-15	ns
FADD-KO TNF vs FADD-KO TNF+C+Z	-12	ns
FADD-KO TNF vs FADD-KO TNF+C+N	-86	***
FADD-KO TNF vs FADD-KO TNF+C+Z+N	-87	***
FADD-KO TNF vs TRADD-KO TNF	-83	***
FADD-KO TNF vs TRADD-KO TNF+Z	-74	***
FADD-KO TNF vs TRADD-KO TNF+N	-80	***
FADD-KO TNF vs TRADD-KO TNF+Z+N	-86	***
FADD-KO TNF vs TRADD-KO TNF+C	2,2	ns
FADD-KO TNF vs TRADD-KO TNF+C+Z	-12	ns
FADD-KO TNF vs TRADD-KO TNF+C+N	-18	ns
FADD-KO TNF vs TRADD-KO TNF+C+Z+N	-85	***
FADD-KO TNF vs RIPK1-KO TNF	-88	***
FADD-KO TNF vs RIPK1-KO TNF+Z	-91	***
FADD-KO TNF vs RIPK1-KO TNF+N	-81	***
FADD-KO TNF vs RIPK1-KO TNF+Z+N	-88	***
FADD-KO TNF vs RIPK1-KO TNF+C	-12	ns
FADD-KO TNF vs RIPK1-KO TNF+C+Z	-87	***
FADD-KO TNF vs RIPK1-KO TNF+C+N	-20	ns
FADD-KO TNF vs RIPK1-KO TNF+C+Z+N	-88	***
FADD-KO TNF vs FADD-TRADD-DKO TNF	-78	***
FADD-KO TNF vs FADD-TRADD-DKO TNF+Z	-74	***
FADD-KO TNF vs FADD-TRADD-DKO TNF+N	-80	***
FADD-KO TNF vs FADD-TRADD-DKO TNF+Z+N	-87	***
FADD-KO TNF vs FADD-TRADD-DKO TNF+C	4,2	ns
FADD-KO TNF vs FADD-TRADD-DKO TNF+C+Z	-0,60	ns
FADD-KO TNF vs FADD-TRADD-DKO TNF+C+N	-58	***
FADD-KO TNF vs FADD-TRADD-DKO TNF+C+Z+N	-84	***
FADD-KO TNF vs FADD-RIPK1-DKO TNF	-90	***

FADD-KO TNF vs FADD-RIPK1-DKO TNF+Z	-90	***
FADD-KO TNF vs FADD-RIPK1-DKO TNF+N	-84	***
FADD-KO TNF vs FADD-RIPK1-DKO TNF+Z+N	-87	***
FADD-KO TNF vs FADD-RIPK1-DKO TNF+C	-84	***
FADD-KO TNF vs FADD-RIPK1-DKO TNF+C+Z	-82	***
FADD-KO TNF vs FADD-RIPK1-DKO TNF+C+N	-80	***
FADD-KO TNF vs FADD-RIPK1-DKO TNF+C+Z+N	-88	***
FADD-KO TNF vs TRADD-RIPK1-DKO TNF	-87	***
FADD-KO TNF vs TRADD-RIPK1-DKO TNF+Z	-87	***
FADD-KO TNF vs TRADD-RIPK1-DKO TNF+N	-84	***
FADD-KO TNF vs TRADD-RIPK1-DKO TNF+Z+N	-82	***
FADD-KO TNF vs TRADD-RIPK1-DKO TNF+C	-77	***
FADD-KO TNF vs TRADD-RIPK1-DKO TNF+C+Z	-87	***
FADD-KO TNF vs TRADD-RIPK1-DKO TNF+C+N	-79	***
FADD-KO TNF vs TRADD-RIPK1-DKO TNF+C+Z+N	-88	***
FADD-KO TNF vs Casp.8-KO TNF	-78	***
FADD-KO TNF vs Casp.8-KO TNF+Z	-81	***
FADD-KO TNF vs Casp.8-KO TNF+N	-88	***
FADD-KO TNF vs Casp.8-KO TNF+Z+N	-90	***
FADD-KO TNF vs Casp.8-KO TNF+C	-52	***
FADD-KO TNF vs Casp.8-KO TNF+C+Z	-53	***
FADD-KO TNF vs Casp.8-KO TNF+C+N	-88	***
FADD-KO TNF vs Casp.8-KO TNF+C+Z+N	-86	***
FADD-KO TNF vs EV TNF	-76	***
FADD-KO TNF vs EV TNF+Z	-85	***
FADD-KO TNF vs EV TNF+N	-74	***
FADD-KO TNF vs EV TNF+Z+N	-83	***
FADD-KO TNF vs EV TNF+C	-13	ns
FADD-KO TNF vs EV TNF+C+Z	-89	***
FADD-KO TNF vs EV TNF+C+N	-24	***
FADD-KO TNF vs EV TNF+C+Z+N	-87	***
FADD-KO TNF+Z vs FADD-KO TNF+N	-89	***
FADD-KO TNF+Z vs FADD-KO TNF+Z+N	-92	***
FADD-KO TNF+Z vs FADD-KO TNF+C	-18	ns
FADD-KO TNF+Z vs FADD-KO TNF+C+Z	-15	ns
FADD-KO TNF+Z vs FADD-KO TNF+C+N	-89	***
FADD-KO TNF+Z vs FADD-KO TNF+C+Z+N	-90	***
FADD-KO TNF+Z vs TRADD-KO TNF	-86	***
FADD-KO TNF+Z vs TRADD-KO TNF+Z	-77	***
FADD-KO TNF+Z vs TRADD-KO TNF+N	-83	***
FADD-KO TNF+Z vs TRADD-KO TNF+Z+N	-89	***
FADD-KO TNF+Z vs TRADD-KO TNF+C	-0,75	ns
FADD-KO TNF+Z vs TRADD-KO TNF+C+Z	-15	ns
FADD-KO TNF+Z vs TRADD-KO TNF+C+N	-21	ns
FADD-KO TNF+Z vs TRADD-KO TNF+C+Z+N	-88	***
FADD-KO TNF+Z vs RIPK1-KO TNF	-91	***
FADD-KO TNF+Z vs RIPK1-KO TNF+Z	-93	***
FADD-KO TNF+Z vs RIPK1-KO TNF+N	-84	***
FADD-KO TNF+Z vs RIPK1-KO TNF+Z+N	-91	***
FADD-KO TNF+Z vs RIPK1-KO TNF+C	-15	ns
FADD-KO TNF+Z vs RIPK1-KO TNF+C+Z	-90	***
FADD-KO TNF+Z vs RIPK1-KO TNF+C+N	-23	ns
FADD-KO TNF+Z vs RIPK1-KO TNF+C+Z+N	-91	***
FADD-KO TNF+Z vs FADD-TRADD-DKO TNF	-81	***
FADD-KO TNF+Z vs FADD-TRADD-DKO TNF+Z	-77	***
FADD-KO TNF+Z vs FADD-TRADD-DKO TNF+N	-83	***
FADD-KO TNF+Z vs FADD-TRADD-DKO TNF+Z+N	-90	***
FADD-KO TNF+Z vs FADD-TRADD-DKO TNF+C	1,3	ns
FADD-KO TNF+Z vs FADD-TRADD-DKO TNF+C+Z	-3,5	ns
FADD-KO TNF+Z vs FADD-TRADD-DKO TNF+C+N	-61	***
FADD-KO TNF+Z vs FADD-TRADD-DKO TNF+C+Z+N	-87	***
FADD-KO TNF+Z vs FADD-RIPK1-DKO TNF	-92	***
FADD-KO TNF+Z vs FADD-RIPK1-DKO TNF+Z	-93	***
FADD-KO TNF+Z vs FADD-RIPK1-DKO TNF+N	-87	***
FADD-KO TNF+Z vs FADD-RIPK1-DKO TNF+Z+N	-90	***
FADD-KO TNF+Z vs FADD-RIPK1-DKO TNF+C	-87	***

FADD-KO TNF+Z vs FADD-RIPK1-DKO TNF+C+Z	-85	***
FADD-KO TNF+Z vs FADD-RIPK1-DKO TNF+C+N	-83	***
FADD-KO TNF+Z vs FADD-RIPK1-DKO TNF+C+Z+N	-91	***
FADD-KO TNF+Z vs TRADD-RIPK1-DKO TNF	-90	***
FADD-KO TNF+Z vs TRADD-RIPK1-DKO TNF+Z	-90	***
FADD-KO TNF+Z vs TRADD-RIPK1-DKO TNF+N	-87	***
FADD-KO TNF+Z vs TRADD-RIPK1-DKO TNF+Z+N	-85	***
FADD-KO TNF+Z vs TRADD-RIPK1-DKO TNF+C	-80	***
FADD-KO TNF+Z vs TRADD-RIPK1-DKO TNF+C+Z	-90	***
FADD-KO TNF+Z vs TRADD-RIPK1-DKO TNF+C+N	-82	***
FADD-KO TNF+Z vs TRADD-RIPK1-DKO TNF+C+Z+N	-91	***
FADD-KO TNF+Z vs Casp.8-KO TNF	-81	***
FADD-KO TNF+Z vs Casp.8-KO TNF+Z	-84	***
FADD-KO TNF+Z vs Casp.8-KO TNF+N	-91	***
FADD-KO TNF+Z vs Casp.8-KO TNF+Z+N	-93	***
FADD-KO TNF+Z vs Casp.8-KO TNF+C	-55	***
FADD-KO TNF+Z vs Casp.8-KO TNF+C+Z	-56	***
FADD-KO TNF+Z vs Casp.8-KO TNF+C+N	-91	***
FADD-KO TNF+Z vs Casp.8-KO TNF+C+Z+N	-89	***
FADD-KO TNF+Z vs EV TNF	-79	***
FADD-KO TNF+Z vs EV TNF+Z	-87	***
FADD-KO TNF+Z vs EV TNF+N	-77	***
FADD-KO TNF+Z vs EV TNF+Z+N	-85	***
FADD-KO TNF+Z vs EV TNF+C	-15	ns
FADD-KO TNF+Z vs EV TNF+C+Z	-91	***
FADD-KO TNF+Z vs EV TNF+C+N	-27	*
FADD-KO TNF+Z vs EV TNF+C+Z+N	-90	***
FADD-KO TNF+N vs FADD-KO TNF+Z+N	-3,2	ns
FADD-KO TNF+N vs FADD-KO TNF+C	71	***
FADD-KO TNF+N vs FADD-KO TNF+C+Z	74	***
FADD-KO TNF+N vs FADD-KO TNF+C+N	0,11	ns
FADD-KO TNF+N vs FADD-KO TNF+C+Z+N	-1,4	ns
FADD-KO TNF+N vs TRADD-KO TNF	3,0	ns
FADD-KO TNF+N vs TRADD-KO TNF+Z	12	**
FADD-KO TNF+N vs TRADD-KO TNF+N	6,5	ns
FADD-KO TNF+N vs TRADD-KO TNF+Z+N	-0,080	ns
FADD-KO TNF+N vs TRADD-KO TNF+C	88	***
FADD-KO TNF+N vs TRADD-KO TNF+C+Z	74	***
FADD-KO TNF+N vs TRADD-KO TNF+C+N	68	***
FADD-KO TNF+N vs TRADD-KO TNF+C+Z+N	1,3	ns
FADD-KO TNF+N vs RIPK1-KO TNF	-2,1	ns
FADD-KO TNF+N vs RIPK1-KO TNF+Z	-4,5	ns
FADD-KO TNF+N vs RIPK1-KO TNF+N	4,7	ns
FADD-KO TNF+N vs RIPK1-KO TNF+Z+N	-1,7	ns
FADD-KO TNF+N vs RIPK1-KO TNF+C	74	***
FADD-KO TNF+N vs RIPK1-KO TNF+C+Z	-0,90	ns
FADD-KO TNF+N vs RIPK1-KO TNF+C+N	66	***
FADD-KO TNF+N vs RIPK1-KO TNF+C+Z+N	-2,3	ns
FADD-KO TNF+N vs FADD-TRADD-DKO TNF	7,6	ns
FADD-KO TNF+N vs FADD-TRADD-DKO TNF+Z	12	ns
FADD-KO TNF+N vs FADD-TRADD-DKO TNF+N	6,4	ns
FADD-KO TNF+N vs FADD-TRADD-DKO TNF+Z+N	-1,4	ns
FADD-KO TNF+N vs FADD-TRADD-DKO TNF+C	90	***
FADD-KO TNF+N vs FADD-TRADD-DKO TNF+C+Z	85	***
FADD-KO TNF+N vs FADD-TRADD-DKO TNF+C+N	28	***
FADD-KO TNF+N vs FADD-TRADD-DKO TNF+C+Z+N	2,1	ns
FADD-KO TNF+N vs FADD-RIPK1-DKO TNF	-3,5	ns
FADD-KO TNF+N vs FADD-RIPK1-DKO TNF+Z	-4,1	ns
FADD-KO TNF+N vs FADD-RIPK1-DKO TNF+N	1,6	ns
FADD-KO TNF+N vs FADD-RIPK1-DKO TNF+Z+N	-0,56	ns
FADD-KO TNF+N vs FADD-RIPK1-DKO TNF+C	2,4	ns
FADD-KO TNF+N vs FADD-RIPK1-DKO TNF+C+Z	4,4	ns
FADD-KO TNF+N vs FADD-RIPK1-DKO TNF+C+N	6,3	ns
FADD-KO TNF+N vs FADD-RIPK1-DKO TNF+C+Z+N	-1,7	ns
FADD-KO TNF+N vs TRADD-RIPK1-DKO TNF	-0,92	ns
FADD-KO TNF+N vs TRADD-RIPK1-DKO TNF+Z	-1,2	ns

FADD-KO TNF+N vs TRADD-RIPK1-DKO TNF+N	2,4	ns
FADD-KO TNF+N vs TRADD-RIPK1-DKO TNF+Z+N	4,1	ns
FADD-KO TNF+N vs TRADD-RIPK1-DKO TNF+C	8,9	ns
FADD-KO TNF+N vs TRADD-RIPK1-DKO TNF+C+Z	-0,89	ns
FADD-KO TNF+N vs TRADD-RIPK1-DKO TNF+C+N	6,7	ns
FADD-KO TNF+N vs TRADD-RIPK1-DKO TNF+C+Z+N	-1,6	ns
FADD-KO TNF+N vs Casp.8-KO TNF	7,7	ns
FADD-KO TNF+N vs Casp.8-KO TNF+Z	5,5	ns
FADD-KO TNF+N vs Casp.8-KO TNF+N	-1,9	ns
FADD-KO TNF+N vs Casp.8-KO TNF+Z+N	-3,5	ns
FADD-KO TNF+N vs Casp.8-KO TNF+C	34	***
FADD-KO TNF+N vs Casp.8-KO TNF+C+Z	33	***
FADD-KO TNF+N vs Casp.8-KO TNF+C+N	-1,7	ns
FADD-KO TNF+N vs Casp.8-KO TNF+C+Z+N	-0,23	ns
FADD-KO TNF+N vs EV TNF	9,8	ns
FADD-KO TNF+N vs EV TNF+Z	1,6	ns
FADD-KO TNF+N vs EV TNF+N	12	ns
FADD-KO TNF+N vs EV TNF+Z+N	3,6	ns
FADD-KO TNF+N vs EV TNF+C	74	***
FADD-KO TNF+N vs EV TNF+C+Z	-2,5	ns
FADD-KO TNF+N vs EV TNF+C+N	62	***
FADD-KO TNF+N vs EV TNF+C+Z+N	-1,0	ns
FADD-KO TNF+Z+N vs FADD-KO TNF+C	74	***
FADD-KO TNF+Z+N vs FADD-KO TNF+C+Z	77	***
FADD-KO TNF+Z+N vs FADD-KO TNF+C+N	3,3	ns
FADD-KO TNF+Z+N vs FADD-KO TNF+C+Z+N	1,8	ns
FADD-KO TNF+Z+N vs TRADD-KO TNF	6,2	ns
FADD-KO TNF+Z+N vs TRADD-KO TNF+Z	16	***
FADD-KO TNF+Z+N vs TRADD-KO TNF+N	9,7	ns
FADD-KO TNF+Z+N vs TRADD-KO TNF+Z+N	3,1	ns
FADD-KO TNF+Z+N vs TRADD-KO TNF+C	91	***
FADD-KO TNF+Z+N vs TRADD-KO TNF+C+Z	77	***
FADD-KO TNF+Z+N vs TRADD-KO TNF+C+N	71	***
FADD-KO TNF+Z+N vs TRADD-KO TNF+C+Z+N	4,5	ns
FADD-KO TNF+Z+N vs RIPK1-KO TNF	1,1	ns
FADD-KO TNF+Z+N vs RIPK1-KO TNF+Z	-1,3	ns
FADD-KO TNF+Z+N vs RIPK1-KO TNF+N	7,9	ns
FADD-KO TNF+Z+N vs RIPK1-KO TNF+Z+N	1,5	ns
FADD-KO TNF+Z+N vs RIPK1-KO TNF+C	78	***
FADD-KO TNF+Z+N vs RIPK1-KO TNF+C+Z	2,3	ns
FADD-KO TNF+Z+N vs RIPK1-KO TNF+C+N	69	***
FADD-KO TNF+Z+N vs RIPK1-KO TNF+C+Z+N	0,91	ns
FADD-KO TNF+Z+N vs FADD-TRADD-DKO TNF	11	ns
FADD-KO TNF+Z+N vs FADD-TRADD-DKO TNF+Z	15	**
FADD-KO TNF+Z+N vs FADD-TRADD-DKO TNF+N	9,6	ns
FADD-KO TNF+Z+N vs FADD-TRADD-DKO TNF+Z+N	1,8	ns
FADD-KO TNF+Z+N vs FADD-TRADD-DKO TNF+C	93	***
FADD-KO TNF+Z+N vs FADD-TRADD-DKO TNF+C+Z	89	***
FADD-KO TNF+Z+N vs FADD-TRADD-DKO TNF+C+N	31	***
FADD-KO TNF+Z+N vs FADD-TRADD-DKO TNF+C+Z+N	5,3	ns
FADD-KO TNF+Z+N vs FADD-RIPK1-DKO TNF	-0,30	ns
FADD-KO TNF+Z+N vs FADD-RIPK1-DKO TNF+Z	-0,85	ns
FADD-KO TNF+Z+N vs FADD-RIPK1-DKO TNF+N	4,8	ns
FADD-KO TNF+Z+N vs FADD-RIPK1-DKO TNF+Z+N	2,6	ns
FADD-KO TNF+Z+N vs FADD-RIPK1-DKO TNF+C	5,6	ns
FADD-KO TNF+Z+N vs FADD-RIPK1-DKO TNF+C+Z	7,6	ns
FADD-KO TNF+Z+N vs FADD-RIPK1-DKO TNF+C+N	9,5	ns
FADD-KO TNF+Z+N vs FADD-RIPK1-DKO TNF+C+Z+N	1,5	ns
FADD-KO TNF+Z+N vs TRADD-RIPK1-DKO TNF	2,3	ns
FADD-KO TNF+Z+N vs TRADD-RIPK1-DKO TNF+Z	2,1	ns
FADD-KO TNF+Z+N vs TRADD-RIPK1-DKO TNF+N	5,6	ns
FADD-KO TNF+Z+N vs TRADD-RIPK1-DKO TNF+Z+N	7,3	ns
FADD-KO TNF+Z+N vs TRADD-RIPK1-DKO TNF+C	12	**
FADD-KO TNF+Z+N vs TRADD-RIPK1-DKO TNF+C+Z	2,3	ns
FADD-KO TNF+Z+N vs TRADD-RIPK1-DKO TNF+C+N	9,9	ns
FADD-KO TNF+Z+N vs TRADD-RIPK1-DKO TNF+C+Z+N	1,6	ns

FADD-KO TNF+Z+N vs Casp.8-KO TNF	11	ns
FADD-KO TNF+Z+N vs Casp.8-KO TNF+Z	8,7	ns
FADD-KO TNF+Z+N vs Casp.8-KO TNF+N	1,3	ns
FADD-KO TNF+Z+N vs Casp.8-KO TNF+Z+N	-0,31	ns
FADD-KO TNF+Z+N vs Casp.8-KO TNF+C	37	***
FADD-KO TNF+Z+N vs Casp.8-KO TNF+C+Z	36	***
FADD-KO TNF+Z+N vs Casp.8-KO TNF+C+N	1,5	ns
FADD-KO TNF+Z+N vs Casp.8-KO TNF+C+Z+N	3,0	ns
FADD-KO TNF+Z+N vs EV TNF	13	ns
FADD-KO TNF+Z+N vs EV TNF+Z	4,8	ns
FADD-KO TNF+Z+N vs EV TNF+N	15	ns
FADD-KO TNF+Z+N vs EV TNF+Z+N	6,8	ns
FADD-KO TNF+Z+N vs EV TNF+C	77	***
FADD-KO TNF+Z+N vs EV TNF+C+Z	0,74	ns
FADD-KO TNF+Z+N vs EV TNF+C+N	65	***
FADD-KO TNF+Z+N vs EV TNF+C+Z+N	2,2	ns
FADD-KO TNF+C vs FADD-KO TNF+C+Z	2,7	ns
FADD-KO TNF+C vs FADD-KO TNF+C+N	-71	***
FADD-KO TNF+C vs FADD-KO TNF+C+Z+N	-72	***
FADD-KO TNF+C vs TRADD-KO TNF	-68	***
FADD-KO TNF+C vs TRADD-KO TNF+Z	-59	***
FADD-KO TNF+C vs TRADD-KO TNF+N	-65	***
FADD-KO TNF+C vs TRADD-KO TNF+Z+N	-71	***
FADD-KO TNF+C vs TRADD-KO TNF+C	17	ns
FADD-KO TNF+C vs TRADD-KO TNF+C+Z	3,0	ns
FADD-KO TNF+C vs TRADD-KO TNF+C+N	-3,0	ns
FADD-KO TNF+C vs TRADD-KO TNF+C+Z+N	-70	***
FADD-KO TNF+C vs RIPK1-KO TNF	-73	***
FADD-KO TNF+C vs RIPK1-KO TNF+Z	-76	***
FADD-KO TNF+C vs RIPK1-KO TNF+N	-66	***
FADD-KO TNF+C vs RIPK1-KO TNF+Z+N	-73	***
FADD-KO TNF+C vs RIPK1-KO TNF+C	3,3	ns
FADD-KO TNF+C vs RIPK1-KO TNF+C+Z	-72	***
FADD-KO TNF+C vs RIPK1-KO TNF+C+N	-5,0	ns
FADD-KO TNF+C vs RIPK1-KO TNF+C+Z+N	-73	***
FADD-KO TNF+C vs FADD-TRADD-DKO TNF	-63	***
FADD-KO TNF+C vs FADD-TRADD-DKO TNF+Z	-59	***
FADD-KO TNF+C vs FADD-TRADD-DKO TNF+N	-65	***
FADD-KO TNF+C vs FADD-TRADD-DKO TNF+Z+N	-72	***
FADD-KO TNF+C vs FADD-TRADD-DKO TNF+C	19	ns
FADD-KO TNF+C vs FADD-TRADD-DKO TNF+C+Z	14	ns
FADD-KO TNF+C vs FADD-TRADD-DKO TNF+C+N	-43	***
FADD-KO TNF+C vs FADD-TRADD-DKO TNF+C+Z+N	-69	***
FADD-KO TNF+C vs FADD-RIPK1-DKO TNF	-75	***
FADD-KO TNF+C vs FADD-RIPK1-DKO TNF+Z	-75	***
FADD-KO TNF+C vs FADD-RIPK1-DKO TNF+N	-69	***
FADD-KO TNF+C vs FADD-RIPK1-DKO TNF+Z+N	-72	***
FADD-KO TNF+C vs FADD-RIPK1-DKO TNF+C	-69	***
FADD-KO TNF+C vs FADD-RIPK1-DKO TNF+C+Z	-67	***
FADD-KO TNF+C vs FADD-RIPK1-DKO TNF+C+N	-65	***
FADD-KO TNF+C vs FADD-RIPK1-DKO TNF+C+Z+N	-73	***
FADD-KO TNF+C vs TRADD-RIPK1-DKO TNF	-72	***
FADD-KO TNF+C vs TRADD-RIPK1-DKO TNF+Z	-72	***
FADD-KO TNF+C vs TRADD-RIPK1-DKO TNF+N	-69	***
FADD-KO TNF+C vs TRADD-RIPK1-DKO TNF+Z+N	-67	***
FADD-KO TNF+C vs TRADD-RIPK1-DKO TNF+C	-62	***
FADD-KO TNF+C vs TRADD-RIPK1-DKO TNF+C+Z	-72	***
FADD-KO TNF+C vs TRADD-RIPK1-DKO TNF+C+N	-64	***
FADD-KO TNF+C vs TRADD-RIPK1-DKO TNF+C+Z+N	-73	***
FADD-KO TNF+C vs Casp.8-KO TNF	-63	***
FADD-KO TNF+C vs Casp.8-KO TNF+Z	-66	***
FADD-KO TNF+C vs Casp.8-KO TNF+N	-73	***
FADD-KO TNF+C vs Casp.8-KO TNF+Z+N	-75	***
FADD-KO TNF+C vs Casp.8-KO TNF+C	-37	***
FADD-KO TNF+C vs Casp.8-KO TNF+C+Z	-38	***
FADD-KO TNF+C vs Casp.8-KO TNF+C+N	-73	***

FADD-KO TNF+C vs Casp.8-KO TNF+C+Z+N	-71	***
FADD-KO TNF+C vs EV TNF	-61	***
FADD-KO TNF+C vs EV TNF+Z	-69	***
FADD-KO TNF+C vs EV TNF+N	-59	***
FADD-KO TNF+C vs EV TNF+Z+N	-67	***
FADD-KO TNF+C vs EV TNF+C	2,5	ns
FADD-KO TNF+C vs EV TNF+C+Z	-74	***
FADD-KO TNF+C vs EV TNF+C+N	-8,8	ns
FADD-KO TNF+C vs EV TNF+C+Z+N	-72	***
FADD-KO TNF+C+Z vs FADD-KO TNF+C+N	-74	***
FADD-KO TNF+C+Z vs FADD-KO TNF+C+Z+N	-75	***
FADD-KO TNF+C+Z vs TRADD-KO TNF	-71	***
FADD-KO TNF+C+Z vs TRADD-KO TNF+Z	-61	***
FADD-KO TNF+C+Z vs TRADD-KO TNF+N	-67	***
FADD-KO TNF+C+Z vs TRADD-KO TNF+Z+N	-74	***
FADD-KO TNF+C+Z vs TRADD-KO TNF+C	15	ns
FADD-KO TNF+C+Z vs TRADD-KO TNF+C+Z	0,25	ns
FADD-KO TNF+C+Z vs TRADD-KO TNF+C+N	-5,8	ns
FADD-KO TNF+C+Z vs TRADD-KO TNF+C+Z+N	-72	***
FADD-KO TNF+C+Z vs RIPK1-KO TNF	-76	***
FADD-KO TNF+C+Z vs RIPK1-KO TNF+Z	-78	***
FADD-KO TNF+C+Z vs RIPK1-KO TNF+N	-69	***
FADD-KO TNF+C+Z vs RIPK1-KO TNF+Z+N	-75	***
FADD-KO TNF+C+Z vs RIPK1-KO TNF+C	0,64	ns
FADD-KO TNF+C+Z vs RIPK1-KO TNF+C+Z	-75	***
FADD-KO TNF+C+Z vs RIPK1-KO TNF+C+N	-7,8	ns
FADD-KO TNF+C+Z vs RIPK1-KO TNF+C+Z+N	-76	***
FADD-KO TNF+C+Z vs FADD-TRADD-DKO TNF	-66	***
FADD-KO TNF+C+Z vs FADD-TRADD-DKO TNF+Z	-62	***
FADD-KO TNF+C+Z vs FADD-TRADD-DKO TNF+N	-67	***
FADD-KO TNF+C+Z vs FADD-TRADD-DKO TNF+Z+N	-75	***
FADD-KO TNF+C+Z vs FADD-TRADD-DKO TNF+C	17	ns
FADD-KO TNF+C+Z vs FADD-TRADD-DKO TNF+C+Z	12	ns
FADD-KO TNF+C+Z vs FADD-TRADD-DKO TNF+C+N	-46	***
FADD-KO TNF+C+Z vs FADD-TRADD-DKO TNF+C+Z+N	-72	***
FADD-KO TNF+C+Z vs FADD-RIPK1-DKO TNF	-77	***
FADD-KO TNF+C+Z vs FADD-RIPK1-DKO TNF+Z	-78	***
FADD-KO TNF+C+Z vs FADD-RIPK1-DKO TNF+N	-72	***
FADD-KO TNF+C+Z vs FADD-RIPK1-DKO TNF+Z+N	-74	***
FADD-KO TNF+C+Z vs FADD-RIPK1-DKO TNF+C	-71	***
FADD-KO TNF+C+Z vs FADD-RIPK1-DKO TNF+C+Z	-69	***
FADD-KO TNF+C+Z vs FADD-RIPK1-DKO TNF+C+N	-67	***
FADD-KO TNF+C+Z vs FADD-RIPK1-DKO TNF+C+Z+N	-75	***
FADD-KO TNF+C+Z vs TRADD-RIPK1-DKO TNF	-75	***
FADD-KO TNF+C+Z vs TRADD-RIPK1-DKO TNF+Z	-75	***
FADD-KO TNF+C+Z vs TRADD-RIPK1-DKO TNF+N	-71	***
FADD-KO TNF+C+Z vs TRADD-RIPK1-DKO TNF+Z+N	-70	***
FADD-KO TNF+C+Z vs TRADD-RIPK1-DKO TNF+C	-65	***
FADD-KO TNF+C+Z vs TRADD-RIPK1-DKO TNF+C+Z	-75	***
FADD-KO TNF+C+Z vs TRADD-RIPK1-DKO TNF+C+N	-67	***
FADD-KO TNF+C+Z vs TRADD-RIPK1-DKO TNF+C+Z+N	-75	***
FADD-KO TNF+C+Z vs Casp.8-KO TNF	-66	***
FADD-KO TNF+C+Z vs Casp.8-KO TNF+Z	-68	***
FADD-KO TNF+C+Z vs Casp.8-KO TNF+N	-76	***
FADD-KO TNF+C+Z vs Casp.8-KO TNF+Z+N	-77	***
FADD-KO TNF+C+Z vs Casp.8-KO TNF+C	-40	***
FADD-KO TNF+C+Z vs Casp.8-KO TNF+C+Z	-41	***
FADD-KO TNF+C+Z vs Casp.8-KO TNF+C+N	-75	***
FADD-KO TNF+C+Z vs Casp.8-KO TNF+C+Z+N	-74	***
FADD-KO TNF+C+Z vs EV TNF	-64	***
FADD-KO TNF+C+Z vs EV TNF+Z	-72	***
FADD-KO TNF+C+Z vs EV TNF+N	-62	***
FADD-KO TNF+C+Z vs EV TNF+Z+N	-70	***
FADD-KO TNF+C+Z vs EV TNF+C	-0,21	ns
FADD-KO TNF+C+Z vs EV TNF+C+Z	-76	***
FADD-KO TNF+C+Z vs EV TNF+C+N	-12	ns

FADD-KO TNF+C+Z vs EV TNF+C+Z+N	-75	***
FADD-KO TNF+C+N vs FADD-KO TNF+C+Z+N	-1,5	ns
FADD-KO TNF+C+N vs TRADD-KO TNF	2,9	ns
FADD-KO TNF+C+N vs TRADD-KO TNF+Z	12	*
FADD-KO TNF+C+N vs TRADD-KO TNF+N	6,4	ns
FADD-KO TNF+C+N vs TRADD-KO TNF+Z+N	-0,19	ns
FADD-KO TNF+C+N vs TRADD-KO TNF+C	88	***
FADD-KO TNF+C+N vs TRADD-KO TNF+C+Z	74	***
FADD-KO TNF+C+N vs TRADD-KO TNF+C+N	68	***
FADD-KO TNF+C+N vs TRADD-KO TNF+C+Z+N	1,2	ns
FADD-KO TNF+C+N vs RIPK1-KO TNF	-2,3	ns
FADD-KO TNF+C+N vs RIPK1-KO TNF+Z	-4,6	ns
FADD-KO TNF+C+N vs RIPK1-KO TNF+N	4,6	ns
FADD-KO TNF+C+N vs RIPK1-KO TNF+Z+N	-1,8	ns
FADD-KO TNF+C+N vs RIPK1-KO TNF+C	74	***
FADD-KO TNF+C+N vs RIPK1-KO TNF+C+Z	-1,0	ns
FADD-KO TNF+C+N vs RIPK1-KO TNF+C+N	66	***
FADD-KO TNF+C+N vs RIPK1-KO TNF+C+Z+N	-2,4	ns
FADD-KO TNF+C+N vs FADD-TRADD-DKO TNF	7,5	ns
FADD-KO TNF+C+N vs FADD-TRADD-DKO TNF+Z	12	ns
FADD-KO TNF+C+N vs FADD-TRADD-DKO TNF+N	6,3	ns
FADD-KO TNF+C+N vs FADD-TRADD-DKO TNF+Z+N	-1,5	ns
FADD-KO TNF+C+N vs FADD-TRADD-DKO TNF+C	90	***
FADD-KO TNF+C+N vs FADD-TRADD-DKO TNF+C+Z	85	***
FADD-KO TNF+C+N vs FADD-TRADD-DKO TNF+C+N	28	***
FADD-KO TNF+C+N vs FADD-TRADD-DKO TNF+C+Z+N	1,9	ns
FADD-KO TNF+C+N vs FADD-RIPK1-DKO TNF	-3,6	ns
FADD-KO TNF+C+N vs FADD-RIPK1-DKO TNF+Z	-4,2	ns
FADD-KO TNF+C+N vs FADD-RIPK1-DKO TNF+N	1,5	ns
FADD-KO TNF+C+N vs FADD-RIPK1-DKO TNF+Z+N	-0,68	ns
FADD-KO TNF+C+N vs FADD-RIPK1-DKO TNF+C	2,3	ns
FADD-KO TNF+C+N vs FADD-RIPK1-DKO TNF+C+Z	4,3	ns
FADD-KO TNF+C+N vs FADD-RIPK1-DKO TNF+C+N	6,2	ns
FADD-KO TNF+C+N vs FADD-RIPK1-DKO TNF+C+Z+N	-1,8	ns
FADD-KO TNF+C+N vs TRADD-RIPK1-DKO TNF	-1,0	ns
FADD-KO TNF+C+N vs TRADD-RIPK1-DKO TNF+Z	-1,3	ns
FADD-KO TNF+C+N vs TRADD-RIPK1-DKO TNF+N	2,3	ns
FADD-KO TNF+C+N vs TRADD-RIPK1-DKO TNF+Z+N	3,9	ns
FADD-KO TNF+C+N vs TRADD-RIPK1-DKO TNF+C	8,8	ns
FADD-KO TNF+C+N vs TRADD-RIPK1-DKO TNF+C+Z	-1,0	ns
FADD-KO TNF+C+N vs TRADD-RIPK1-DKO TNF+C+N	6,5	ns
FADD-KO TNF+C+N vs TRADD-RIPK1-DKO TNF+C+Z+N	-1,7	ns
FADD-KO TNF+C+N vs Casp.8-KO TNF	7,6	ns
FADD-KO TNF+C+N vs Casp.8-KO TNF+Z	5,4	ns
FADD-KO TNF+C+N vs Casp.8-KO TNF+N	-2,0	ns
FADD-KO TNF+C+N vs Casp.8-KO TNF+Z+N	-3,6	ns
FADD-KO TNF+C+N vs Casp.8-KO TNF+C	34	***
FADD-KO TNF+C+N vs Casp.8-KO TNF+C+Z	33	***
FADD-KO TNF+C+N vs Casp.8-KO TNF+C+N	-1,8	ns
FADD-KO TNF+C+N vs Casp.8-KO TNF+C+Z+N	-0,34	ns
FADD-KO TNF+C+N vs EV TNF	9,7	ns
FADD-KO TNF+C+N vs EV TNF+Z	1,5	ns
FADD-KO TNF+C+N vs EV TNF+N	12	ns
FADD-KO TNF+C+N vs EV TNF+Z+N	3,5	ns
FADD-KO TNF+C+N vs EV TNF+C	73	***
FADD-KO TNF+C+N vs EV TNF+C+Z	-2,6	ns
FADD-KO TNF+C+N vs EV TNF+C+N	62	***
FADD-KO TNF+C+N vs EV TNF+C+Z+N	-1,2	ns
FADD-KO TNF+C+Z+N vs TRADD-KO TNF	4,4	ns
FADD-KO TNF+C+Z+N vs TRADD-KO TNF+Z	14	***
FADD-KO TNF+C+Z+N vs TRADD-KO TNF+N	7,8	ns
FADD-KO TNF+C+Z+N vs TRADD-KO TNF+Z+N	1,3	ns
FADD-KO TNF+C+Z+N vs TRADD-KO TNF+C	90	***
FADD-KO TNF+C+Z+N vs TRADD-KO TNF+C+Z	75	***
FADD-KO TNF+C+Z+N vs TRADD-KO TNF+C+N	69	***
FADD-KO TNF+C+Z+N vs TRADD-KO TNF+C+Z+N	2,7	ns

FADD-KO TNF+C+Z+N vs RIPK1-KO TNF	-0,78	ns
FADD-KO TNF+C+Z+N vs RIPK1-KO TNF+Z	-3,1	ns
FADD-KO TNF+C+Z+N vs RIPK1-KO TNF+N	6,1	ns
FADD-KO TNF+C+Z+N vs RIPK1-KO TNF+Z+N	-0,34	ns
FADD-KO TNF+C+Z+N vs RIPK1-KO TNF+C	76	***
FADD-KO TNF+C+Z+N vs RIPK1-KO TNF+C+Z	0,47	ns
FADD-KO TNF+C+Z+N vs RIPK1-KO TNF+C+N	67	***
FADD-KO TNF+C+Z+N vs RIPK1-KO TNF+C+Z+N	-0,93	ns
FADD-KO TNF+C+Z+N vs FADD-TRADD-DKO TNF	9,0	ns
FADD-KO TNF+C+Z+N vs FADD-TRADD-DKO TNF+Z	13	ns
FADD-KO TNF+C+Z+N vs FADD-TRADD-DKO TNF+N	7,8	ns
FADD-KO TNF+C+Z+N vs FADD-TRADD-DKO TNF+Z+N	-0,025	ns
FADD-KO TNF+C+Z+N vs FADD-TRADD-DKO TNF+C	92	***
FADD-KO TNF+C+Z+N vs FADD-TRADD-DKO TNF+C+Z	87	***
FADD-KO TNF+C+Z+N vs FADD-TRADD-DKO TNF+C+N	29	***
FADD-KO TNF+C+Z+N vs FADD-TRADD-DKO TNF+C+Z+N	3,4	ns
FADD-KO TNF+C+Z+N vs FADD-RIPK1-DKO TNF	-2,1	ns
FADD-KO TNF+C+Z+N vs FADD-RIPK1-DKO TNF+Z	-2,7	ns
FADD-KO TNF+C+Z+N vs FADD-RIPK1-DKO TNF+N	3,0	ns
FADD-KO TNF+C+Z+N vs FADD-RIPK1-DKO TNF+Z+N	0,80	ns
FADD-KO TNF+C+Z+N vs FADD-RIPK1-DKO TNF+C	3,8	ns
FADD-KO TNF+C+Z+N vs FADD-RIPK1-DKO TNF+C+Z	5,8	ns
FADD-KO TNF+C+Z+N vs FADD-RIPK1-DKO TNF+C+N	7,7	ns
FADD-KO TNF+C+Z+N vs FADD-RIPK1-DKO TNF+C+Z+N	-0,31	ns
FADD-KO TNF+C+Z+N vs TRADD-RIPK1-DKO TNF	0,44	ns
FADD-KO TNF+C+Z+N vs TRADD-RIPK1-DKO TNF+Z	0,21	ns
FADD-KO TNF+C+Z+N vs TRADD-RIPK1-DKO TNF+N	3,8	ns
FADD-KO TNF+C+Z+N vs TRADD-RIPK1-DKO TNF+Z+N	5,4	ns
FADD-KO TNF+C+Z+N vs TRADD-RIPK1-DKO TNF+C	10	ns
FADD-KO TNF+C+Z+N vs TRADD-RIPK1-DKO TNF+C+Z	0,47	ns
FADD-KO TNF+C+Z+N vs TRADD-RIPK1-DKO TNF+C+N	8,0	ns
FADD-KO TNF+C+Z+N vs TRADD-RIPK1-DKO TNF+C+Z+N	-0,20	ns
FADD-KO TNF+C+Z+N vs Casp.8-KO TNF	9,1	ns
FADD-KO TNF+C+Z+N vs Casp.8-KO TNF+Z	6,8	ns
FADD-KO TNF+C+Z+N vs Casp.8-KO TNF+N	-0,57	ns
FADD-KO TNF+C+Z+N vs Casp.8-KO TNF+Z+N	-2,2	ns
FADD-KO TNF+C+Z+N vs Casp.8-KO TNF+C	35	***
FADD-KO TNF+C+Z+N vs Casp.8-KO TNF+C+Z	34	***
FADD-KO TNF+C+Z+N vs Casp.8-KO TNF+C+N	-0,33	ns
FADD-KO TNF+C+Z+N vs Casp.8-KO TNF+C+Z+N	1,1	ns
FADD-KO TNF+C+Z+N vs EV TNF	11	ns
FADD-KO TNF+C+Z+N vs EV TNF+Z	2,9	ns
FADD-KO TNF+C+Z+N vs EV TNF+N	13	ns
FADD-KO TNF+C+Z+N vs EV TNF+Z+N	4,9	ns
FADD-KO TNF+C+Z+N vs EV TNF+C	75	***
FADD-KO TNF+C+Z+N vs EV TNF+C+Z	-1,1	ns
FADD-KO TNF+C+Z+N vs EV TNF+C+N	64	***
FADD-KO TNF+C+Z+N vs EV TNF+C+Z+N	0,31	ns
TRADD-KO TNF vs TRADD-KO TNF+Z	9,3	ns
TRADD-KO TNF vs TRADD-KO TNF+N	3,5	ns
TRADD-KO TNF vs TRADD-KO TNF+Z+N	-3,1	ns
TRADD-KO TNF vs TRADD-KO TNF+C	85	***
TRADD-KO TNF vs TRADD-KO TNF+C+Z	71	***
TRADD-KO TNF vs TRADD-KO TNF+C+N	65	***
TRADD-KO TNF vs TRADD-KO TNF+C+Z+N	-1,7	ns
TRADD-KO TNF vs RIPK1-KO TNF	-5,1	ns
TRADD-KO TNF vs RIPK1-KO TNF+Z	-7,5	ns
TRADD-KO TNF vs RIPK1-KO TNF+N	1,7	ns
TRADD-KO TNF vs RIPK1-KO TNF+Z+N	-4,7	ns
TRADD-KO TNF vs RIPK1-KO TNF+C	71	***
TRADD-KO TNF vs RIPK1-KO TNF+C+Z	-3,9	ns
TRADD-KO TNF vs RIPK1-KO TNF+C+N	63	***
TRADD-KO TNF vs RIPK1-KO TNF+C+Z+N	-5,3	ns
TRADD-KO TNF vs FADD-TRADD-DKO TNF	4,6	ns
TRADD-KO TNF vs FADD-TRADD-DKO TNF+Z	9,0	ns
TRADD-KO TNF vs FADD-TRADD-DKO TNF+N	3,4	ns

TRADD-KO TNF vs FADD-TRADD-DKO TNF+Z+N	-4,4	ns
TRADD-KO TNF vs FADD-TRADD-DKO TNF+C	87	***
TRADD-KO TNF vs FADD-TRADD-DKO TNF+C+Z	82	***
TRADD-KO TNF vs FADD-TRADD-DKO TNF+C+N	25	***
TRADD-KO TNF vs FADD-TRADD-DKO TNF+C+Z+N	-0,94	ns
TRADD-KO TNF vs FADD-RIPK1-DKO TNF	-6,5	ns
TRADD-KO TNF vs FADD-RIPK1-DKO TNF+Z	-7,1	ns
TRADD-KO TNF vs FADD-RIPK1-DKO TNF+N	-1,4	ns
TRADD-KO TNF vs FADD-RIPK1-DKO TNF+Z+N	-3,6	ns
TRADD-KO TNF vs FADD-RIPK1-DKO TNF+C	-0,58	ns
TRADD-KO TNF vs FADD-RIPK1-DKO TNF+C+Z	1,4	ns
TRADD-KO TNF vs FADD-RIPK1-DKO TNF+C+N	3,3	ns
TRADD-KO TNF vs FADD-RIPK1-DKO TNF+C+Z+N	-4,7	ns
TRADD-KO TNF vs TRADD-RIPK1-DKO TNF	-3,9	ns
TRADD-KO TNF vs TRADD-RIPK1-DKO TNF+Z	-4,1	ns
TRADD-KO TNF vs TRADD-RIPK1-DKO TNF+N	-0,58	ns
TRADD-KO TNF vs TRADD-RIPK1-DKO TNF+Z+N	1,1	ns
TRADD-KO TNF vs TRADD-RIPK1-DKO TNF+C	5,9	ns
TRADD-KO TNF vs TRADD-RIPK1-DKO TNF+C+Z	-3,9	ns
TRADD-KO TNF vs TRADD-RIPK1-DKO TNF+C+N	3,7	ns
TRADD-KO TNF vs TRADD-RIPK1-DKO TNF+C+Z+N	-4,6	ns
TRADD-KO TNF vs Casp.8-KO TNF	4,7	ns
TRADD-KO TNF vs Casp.8-KO TNF+Z	2,5	ns
TRADD-KO TNF vs Casp.8-KO TNF+N	-4,9	ns
TRADD-KO TNF vs Casp.8-KO TNF+Z+N	-6,5	ns
TRADD-KO TNF vs Casp.8-KO TNF+C	31	***
TRADD-KO TNF vs Casp.8-KO TNF+C+Z	30	***
TRADD-KO TNF vs Casp.8-KO TNF+C+N	-4,7	ns
TRADD-KO TNF vs Casp.8-KO TNF+C+Z+N	-3,2	ns
TRADD-KO TNF vs EV TNF	6,8	ns
TRADD-KO TNF vs EV TNF+Z	-1,4	ns
TRADD-KO TNF vs EV TNF+N	8,9	ns
TRADD-KO TNF vs EV TNF+Z+N	0,57	ns
TRADD-KO TNF vs EV TNF+C	71	***
TRADD-KO TNF vs EV TNF+C+Z	-5,5	ns
TRADD-KO TNF vs EV TNF+C+N	59	***
TRADD-KO TNF vs EV TNF+C+Z+N	-4,0	ns
TRADD-KO TNF+Z vs TRADD-KO TNF+N	-5,9	ns
TRADD-KO TNF+Z vs TRADD-KO TNF+Z+N	-12	**
TRADD-KO TNF+Z vs TRADD-KO TNF+C	76	***
TRADD-KO TNF+Z vs TRADD-KO TNF+C+Z	62	***
TRADD-KO TNF+Z vs TRADD-KO TNF+C+N	56	***
TRADD-KO TNF+Z vs TRADD-KO TNF+C+Z+N	-11	ns
TRADD-KO TNF+Z vs RIPK1-KO TNF	-14	***
TRADD-KO TNF+Z vs RIPK1-KO TNF+Z	-17	***
TRADD-KO TNF+Z vs RIPK1-KO TNF+N	-7,6	ns
TRADD-KO TNF+Z vs RIPK1-KO TNF+Z+N	-14	***
TRADD-KO TNF+Z vs RIPK1-KO TNF+C	62	***
TRADD-KO TNF+Z vs RIPK1-KO TNF+C+Z	-13	**
TRADD-KO TNF+Z vs RIPK1-KO TNF+C+N	54	***
TRADD-KO TNF+Z vs RIPK1-KO TNF+C+Z+N	-15	***
TRADD-KO TNF+Z vs FADD-TRADD-DKO TNF	-4,7	ns
TRADD-KO TNF+Z vs FADD-TRADD-DKO TNF+Z	-0,36	ns
TRADD-KO TNF+Z vs FADD-TRADD-DKO TNF+N	-5,9	ns
TRADD-KO TNF+Z vs FADD-TRADD-DKO TNF+Z+N	-14	**
TRADD-KO TNF+Z vs FADD-TRADD-DKO TNF+C	78	***
TRADD-KO TNF+Z vs FADD-TRADD-DKO TNF+C+Z	73	***
TRADD-KO TNF+Z vs FADD-TRADD-DKO TNF+C+N	16	*
TRADD-KO TNF+Z vs FADD-TRADD-DKO TNF+C+Z+N	-10	ns
TRADD-KO TNF+Z vs FADD-RIPK1-DKO TNF	-16	***
TRADD-KO TNF+Z vs FADD-RIPK1-DKO TNF+Z	-16	***
TRADD-KO TNF+Z vs FADD-RIPK1-DKO TNF+N	-11	ns
TRADD-KO TNF+Z vs FADD-RIPK1-DKO TNF+Z+N	-13	*
TRADD-KO TNF+Z vs FADD-RIPK1-DKO TNF+C	-9,9	ns
TRADD-KO TNF+Z vs FADD-RIPK1-DKO TNF+C+Z	-7,9	ns
TRADD-KO TNF+Z vs FADD-RIPK1-DKO TNF+C+N	-6,0	ns

TRADD-KO TNF+Z vs FADD-RIPK1-DKO TNF+C+Z+N	-14	*
TRADD-KO TNF+Z vs TRADD-RIPK1-DKO TNF	-13	***
TRADD-KO TNF+Z vs TRADD-RIPK1-DKO TNF+Z	-13	**
TRADD-KO TNF+Z vs TRADD-RIPK1-DKO TNF+N	-9,9	ns
TRADD-KO TNF+Z vs TRADD-RIPK1-DKO TNF+Z+N	-8,3	ns
TRADD-KO TNF+Z vs TRADD-RIPK1-DKO TNF+C	-3,5	ns
TRADD-KO TNF+Z vs TRADD-RIPK1-DKO TNF+C+Z	-13	**
TRADD-KO TNF+Z vs TRADD-RIPK1-DKO TNF+C+N	-5,7	ns
TRADD-KO TNF+Z vs TRADD-RIPK1-DKO TNF+C+Z+N	-14	**
TRADD-KO TNF+Z vs Casp.8-KO TNF	-4,6	ns
TRADD-KO TNF+Z vs Casp.8-KO TNF+Z	-6,9	ns
TRADD-KO TNF+Z vs Casp.8-KO TNF+N	-14	**
TRADD-KO TNF+Z vs Casp.8-KO TNF+Z+N	-16	***
TRADD-KO TNF+Z vs Casp.8-KO TNF+C	22	***
TRADD-KO TNF+Z vs Casp.8-KO TNF+C+Z	20	***
TRADD-KO TNF+Z vs Casp.8-KO TNF+C+N	-14	**
TRADD-KO TNF+Z vs Casp.8-KO TNF+C+Z+N	-13	*
TRADD-KO TNF+Z vs EV TNF	-2,5	ns
TRADD-KO TNF+Z vs EV TNF+Z	-11	ns
TRADD-KO TNF+Z vs EV TNF+N	-0,44	ns
TRADD-KO TNF+Z vs EV TNF+Z+N	-8,8	ns
TRADD-KO TNF+Z vs EV TNF+C	61	***
TRADD-KO TNF+Z vs EV TNF+C+Z	-15	*
TRADD-KO TNF+Z vs EV TNF+C+N	50	***
TRADD-KO TNF+Z vs EV TNF+C+Z+N	-13	ns
TRADD-KO TNF+N vs TRADD-KO TNF+Z+N	-6,5	ns
TRADD-KO TNF+N vs TRADD-KO TNF+C	82	***
TRADD-KO TNF+N vs TRADD-KO TNF+C+Z	68	***
TRADD-KO TNF+N vs TRADD-KO TNF+C+N	62	***
TRADD-KO TNF+N vs TRADD-KO TNF+C+Z+N	-5,2	ns
TRADD-KO TNF+N vs RIPK1-KO TNF	-8,6	ns
TRADD-KO TNF+N vs RIPK1-KO TNF+Z	-11	ns
TRADD-KO TNF+N vs RIPK1-KO TNF+N	-1,8	ns
TRADD-KO TNF+N vs RIPK1-KO TNF+Z+N	-8,2	ns
TRADD-KO TNF+N vs RIPK1-KO TNF+C	68	***
TRADD-KO TNF+N vs RIPK1-KO TNF+C+Z	-7,4	ns
TRADD-KO TNF+N vs RIPK1-KO TNF+C+N	60	***
TRADD-KO TNF+N vs RIPK1-KO TNF+C+Z+N	-8,8	ns
TRADD-KO TNF+N vs FADD-TRADD-DKO TNF	1,1	ns
TRADD-KO TNF+N vs FADD-TRADD-DKO TNF+Z	5,5	ns
TRADD-KO TNF+N vs FADD-TRADD-DKO TNF+N	-0,054	ns
TRADD-KO TNF+N vs FADD-TRADD-DKO TNF+Z+N	-7,9	ns
TRADD-KO TNF+N vs FADD-TRADD-DKO TNF+C	84	***
TRADD-KO TNF+N vs FADD-TRADD-DKO TNF+C+Z	79	***
TRADD-KO TNF+N vs FADD-TRADD-DKO TNF+C+N	21	***
TRADD-KO TNF+N vs FADD-TRADD-DKO TNF+C+Z+N	-4,4	ns
TRADD-KO TNF+N vs FADD-RIPK1-DKO TNF	-10	ns
TRADD-KO TNF+N vs FADD-RIPK1-DKO TNF+Z	-11	ns
TRADD-KO TNF+N vs FADD-RIPK1-DKO TNF+N	-4,8	ns
TRADD-KO TNF+N vs FADD-RIPK1-DKO TNF+Z+N	-7,0	ns
TRADD-KO TNF+N vs FADD-RIPK1-DKO TNF+C	-4,0	ns
TRADD-KO TNF+N vs FADD-RIPK1-DKO TNF+C+Z	-2,0	ns
TRADD-KO TNF+N vs FADD-RIPK1-DKO TNF+C+N	-0,15	ns
TRADD-KO TNF+N vs FADD-RIPK1-DKO TNF+C+Z+N	-8,1	ns
TRADD-KO TNF+N vs TRADD-RIPK1-DKO TNF	-7,4	ns
TRADD-KO TNF+N vs TRADD-RIPK1-DKO TNF+Z	-7,6	ns
TRADD-KO TNF+N vs TRADD-RIPK1-DKO TNF+N	-4,0	ns
TRADD-KO TNF+N vs TRADD-RIPK1-DKO TNF+Z+N	-2,4	ns
TRADD-KO TNF+N vs TRADD-RIPK1-DKO TNF+C	2,4	ns
TRADD-KO TNF+N vs TRADD-RIPK1-DKO TNF+C+Z	-7,4	ns
TRADD-KO TNF+N vs TRADD-RIPK1-DKO TNF+C+N	0,19	ns
TRADD-KO TNF+N vs TRADD-RIPK1-DKO TNF+C+Z+N	-8,0	ns
TRADD-KO TNF+N vs Casp.8-KO TNF	1,3	ns
TRADD-KO TNF+N vs Casp.8-KO TNF+Z	-1,0	ns
TRADD-KO TNF+N vs Casp.8-KO TNF+N	-8,4	ns
TRADD-KO TNF+N vs Casp.8-KO TNF+Z+N	-10	ns

TRADD-KO TNF+N vs Casp.8-KO TNF+C	28	***
TRADD-KO TNF+N vs Casp.8-KO TNF+C+Z	26	***
TRADD-KO TNF+N vs Casp.8-KO TNF+C+N	-8,2	ns
TRADD-KO TNF+N vs Casp.8-KO TNF+C+Z+N	-6,7	ns
TRADD-KO TNF+N vs EV TNF	3,3	ns
TRADD-KO TNF+N vs EV TNF+Z	-4,9	ns
TRADD-KO TNF+N vs EV TNF+N	5,4	ns
TRADD-KO TNF+N vs EV TNF+Z+N	-2,9	ns
TRADD-KO TNF+N vs EV TNF+C	67	***
TRADD-KO TNF+N vs EV TNF+C+Z	-8,9	ns
TRADD-KO TNF+N vs EV TNF+C+N	56	***
TRADD-KO TNF+N vs EV TNF+C+Z+N	-7,5	ns
TRADD-KO TNF+Z+N vs TRADD-KO TNF+C	88	***
TRADD-KO TNF+Z+N vs TRADD-KO TNF+C+Z	74	***
TRADD-KO TNF+Z+N vs TRADD-KO TNF+C+N	68	***
TRADD-KO TNF+Z+N vs TRADD-KO TNF+C+Z+N	1,4	ns
TRADD-KO TNF+Z+N vs RIPK1-KO TNF	-2,1	ns
TRADD-KO TNF+Z+N vs RIPK1-KO TNF+Z	-4,4	ns
TRADD-KO TNF+Z+N vs RIPK1-KO TNF+N	4,8	ns
TRADD-KO TNF+Z+N vs RIPK1-KO TNF+Z+N	-1,6	ns
TRADD-KO TNF+Z+N vs RIPK1-KO TNF+C	74	***
TRADD-KO TNF+Z+N vs RIPK1-KO TNF+C+Z	-0,82	ns
TRADD-KO TNF+Z+N vs RIPK1-KO TNF+C+N	66	***
TRADD-KO TNF+Z+N vs RIPK1-KO TNF+C+Z+N	-2,2	ns
TRADD-KO TNF+Z+N vs FADD-TRADD-DKO TNF	7,7	ns
TRADD-KO TNF+Z+N vs FADD-TRADD-DKO TNF+Z	12	ns
TRADD-KO TNF+Z+N vs FADD-TRADD-DKO TNF+N	6,5	ns
TRADD-KO TNF+Z+N vs FADD-TRADD-DKO TNF+Z+N	-1,3	ns
TRADD-KO TNF+Z+N vs FADD-TRADD-DKO TNF+C	90	***
TRADD-KO TNF+Z+N vs FADD-TRADD-DKO TNF+C+Z	86	***
TRADD-KO TNF+Z+N vs FADD-TRADD-DKO TNF+C+N	28	***
TRADD-KO TNF+Z+N vs FADD-TRADD-DKO TNF+C+Z+N	2,1	ns
TRADD-KO TNF+Z+N vs FADD-RIPK1-DKO TNF	-3,4	ns
TRADD-KO TNF+Z+N vs FADD-RIPK1-DKO TNF+Z	-4,0	ns
TRADD-KO TNF+Z+N vs FADD-RIPK1-DKO TNF+N	1,7	ns
TRADD-KO TNF+Z+N vs FADD-RIPK1-DKO TNF+Z+N	-0,48	ns
TRADD-KO TNF+Z+N vs FADD-RIPK1-DKO TNF+C	2,5	ns
TRADD-KO TNF+Z+N vs FADD-RIPK1-DKO TNF+C+Z	4,5	ns
TRADD-KO TNF+Z+N vs FADD-RIPK1-DKO TNF+C+N	6,4	ns
TRADD-KO TNF+Z+N vs FADD-RIPK1-DKO TNF+C+Z+N	-1,6	ns
TRADD-KO TNF+Z+N vs TRADD-RIPK1-DKO TNF	-0,84	ns
TRADD-KO TNF+Z+N vs TRADD-RIPK1-DKO TNF+Z	-1,1	ns
TRADD-KO TNF+Z+N vs TRADD-RIPK1-DKO TNF+N	2,5	ns
TRADD-KO TNF+Z+N vs TRADD-RIPK1-DKO TNF+Z+N	4,1	ns
TRADD-KO TNF+Z+N vs TRADD-RIPK1-DKO TNF+C	9,0	ns
TRADD-KO TNF+Z+N vs TRADD-RIPK1-DKO TNF+C+Z	-0,81	ns
TRADD-KO TNF+Z+N vs TRADD-RIPK1-DKO TNF+C+N	6,7	ns
TRADD-KO TNF+Z+N vs TRADD-RIPK1-DKO TNF+C+Z+N	-1,5	ns
TRADD-KO TNF+Z+N vs Casp.8-KO TNF	7,8	ns
TRADD-KO TNF+Z+N vs Casp.8-KO TNF+Z	5,6	ns
TRADD-KO TNF+Z+N vs Casp.8-KO TNF+N	-1,8	ns
TRADD-KO TNF+Z+N vs Casp.8-KO TNF+Z+N	-3,4	ns
TRADD-KO TNF+Z+N vs Casp.8-KO TNF+C	34	***
TRADD-KO TNF+Z+N vs Casp.8-KO TNF+C+Z	33	***
TRADD-KO TNF+Z+N vs Casp.8-KO TNF+C+N	-1,6	ns
TRADD-KO TNF+Z+N vs Casp.8-KO TNF+C+Z+N	-0,15	ns
TRADD-KO TNF+Z+N vs EV TNF	9,9	ns
TRADD-KO TNF+Z+N vs EV TNF+Z	1,7	ns
TRADD-KO TNF+Z+N vs EV TNF+N	12	ns
TRADD-KO TNF+Z+N vs EV TNF+Z+N	3,7	ns
TRADD-KO TNF+Z+N vs EV TNF+C	74	***
TRADD-KO TNF+Z+N vs EV TNF+C+Z	-2,4	ns
TRADD-KO TNF+Z+N vs EV TNF+C+N	62	***
TRADD-KO TNF+Z+N vs EV TNF+C+Z+N	-0,97	ns
TRADD-KO TNF+C vs TRADD-KO TNF+C+Z	-14	ns
TRADD-KO TNF+C vs TRADD-KO TNF+C+N	-20	ns

TRADD-KO TNF+C vs TRADD-KO TNF+C+Z+N	-87	***
TRADD-KO TNF+C vs RIPK1-KO TNF	-90	***
TRADD-KO TNF+C vs RIPK1-KO TNF+Z	-93	***
TRADD-KO TNF+C vs RIPK1-KO TNF+N	-84	***
TRADD-KO TNF+C vs RIPK1-KO TNF+Z+N	-90	***
TRADD-KO TNF+C vs RIPK1-KO TNF+C	-14	ns
TRADD-KO TNF+C vs RIPK1-KO TNF+C+Z	-89	***
TRADD-KO TNF+C vs RIPK1-KO TNF+C+N	-22	ns
TRADD-KO TNF+C vs RIPK1-KO TNF+C+Z+N	-91	***
TRADD-KO TNF+C vs FADD-TRADD-DKO TNF	-81	***
TRADD-KO TNF+C vs FADD-TRADD-DKO TNF+Z	-76	***
TRADD-KO TNF+C vs FADD-TRADD-DKO TNF+N	-82	***
TRADD-KO TNF+C vs FADD-TRADD-DKO TNF+Z+N	-90	***
TRADD-KO TNF+C vs FADD-TRADD-DKO TNF+C	2,0	ns
TRADD-KO TNF+C vs FADD-TRADD-DKO TNF+C+Z	-2,8	ns
TRADD-KO TNF+C vs FADD-TRADD-DKO TNF+C+N	-60	***
TRADD-KO TNF+C vs FADD-TRADD-DKO TNF+C+Z+N	-86	***
TRADD-KO TNF+C vs FADD-RIPK1-DKO TNF	-92	***
TRADD-KO TNF+C vs FADD-RIPK1-DKO TNF+Z	-92	***
TRADD-KO TNF+C vs FADD-RIPK1-DKO TNF+N	-87	***
TRADD-KO TNF+C vs FADD-RIPK1-DKO TNF+Z+N	-89	***
TRADD-KO TNF+C vs FADD-RIPK1-DKO TNF+C	-86	***
TRADD-KO TNF+C vs FADD-RIPK1-DKO TNF+C+Z	-84	***
TRADD-KO TNF+C vs FADD-RIPK1-DKO TNF+C+N	-82	***
TRADD-KO TNF+C vs FADD-RIPK1-DKO TNF+C+Z+N	-90	***
TRADD-KO TNF+C vs TRADD-RIPK1-DKO TNF	-89	***
TRADD-KO TNF+C vs TRADD-RIPK1-DKO TNF+Z	-89	***
TRADD-KO TNF+C vs TRADD-RIPK1-DKO TNF+N	-86	***
TRADD-KO TNF+C vs TRADD-RIPK1-DKO TNF+Z+N	-84	***
TRADD-KO TNF+C vs TRADD-RIPK1-DKO TNF+C	-79	***
TRADD-KO TNF+C vs TRADD-RIPK1-DKO TNF+C+Z	-89	***
TRADD-KO TNF+C vs TRADD-RIPK1-DKO TNF+C+N	-82	***
TRADD-KO TNF+C vs TRADD-RIPK1-DKO TNF+C+Z+N	-90	***
TRADD-KO TNF+C vs Casp.8-KO TNF	-81	***
TRADD-KO TNF+C vs Casp.8-KO TNF+Z	-83	***
TRADD-KO TNF+C vs Casp.8-KO TNF+N	-90	***
TRADD-KO TNF+C vs Casp.8-KO TNF+Z+N	-92	***
TRADD-KO TNF+C vs Casp.8-KO TNF+C	-54	***
TRADD-KO TNF+C vs Casp.8-KO TNF+C+Z	-55	***
TRADD-KO TNF+C vs Casp.8-KO TNF+C+N	-90	***
TRADD-KO TNF+C vs Casp.8-KO TNF+C+Z+N	-88	***
TRADD-KO TNF+C vs EV TNF	-78	***
TRADD-KO TNF+C vs EV TNF+Z	-87	***
TRADD-KO TNF+C vs EV TNF+N	-76	***
TRADD-KO TNF+C vs EV TNF+Z+N	-85	***
TRADD-KO TNF+C vs EV TNF+C	-15	ns
TRADD-KO TNF+C vs EV TNF+C+Z	-91	***
TRADD-KO TNF+C vs EV TNF+C+N	-26	**
TRADD-KO TNF+C vs EV TNF+C+Z+N	-89	***
TRADD-KO TNF+C+Z vs TRADD-KO TNF+C+N	-6,0	ns
TRADD-KO TNF+C+Z vs TRADD-KO TNF+C+Z+N	-73	***
TRADD-KO TNF+C+Z vs RIPK1-KO TNF	-76	***
TRADD-KO TNF+C+Z vs RIPK1-KO TNF+Z	-78	***
TRADD-KO TNF+C+Z vs RIPK1-KO TNF+N	-69	***
TRADD-KO TNF+C+Z vs RIPK1-KO TNF+Z+N	-76	***
TRADD-KO TNF+C+Z vs RIPK1-KO TNF+C	0,39	ns
TRADD-KO TNF+C+Z vs RIPK1-KO TNF+C+Z	-75	***
TRADD-KO TNF+C+Z vs RIPK1-KO TNF+C+N	-8,0	ns
TRADD-KO TNF+C+Z vs RIPK1-KO TNF+C+Z+N	-76	***
TRADD-KO TNF+C+Z vs FADD-TRADD-DKO TNF	-66	***
TRADD-KO TNF+C+Z vs FADD-TRADD-DKO TNF+Z	-62	***
TRADD-KO TNF+C+Z vs FADD-TRADD-DKO TNF+N	-68	***
TRADD-KO TNF+C+Z vs FADD-TRADD-DKO TNF+Z+N	-75	***
TRADD-KO TNF+C+Z vs FADD-TRADD-DKO TNF+C	16	ns
TRADD-KO TNF+C+Z vs FADD-TRADD-DKO TNF+C+Z	12	ns
TRADD-KO TNF+C+Z vs FADD-TRADD-DKO TNF+C+N	-46	***

TRADD-KO TNF+C+Z vs FADD-TRADD-DKO TNF+C+Z+N	-72	***
TRADD-KO TNF+C+Z vs FADD-RIPK1-DKO TNF	-77	***
TRADD-KO TNF+C+Z vs FADD-RIPK1-DKO TNF+Z	-78	***
TRADD-KO TNF+C+Z vs FADD-RIPK1-DKO TNF+N	-72	***
TRADD-KO TNF+C+Z vs FADD-RIPK1-DKO TNF+Z+N	-75	***
TRADD-KO TNF+C+Z vs FADD-RIPK1-DKO TNF+C	-72	***
TRADD-KO TNF+C+Z vs FADD-RIPK1-DKO TNF+C+Z	-70	***
TRADD-KO TNF+C+Z vs FADD-RIPK1-DKO TNF+C+N	-68	***
TRADD-KO TNF+C+Z vs FADD-RIPK1-DKO TNF+C+Z+N	-76	***
TRADD-KO TNF+C+Z vs TRADD-RIPK1-DKO TNF	-75	***
TRADD-KO TNF+C+Z vs TRADD-RIPK1-DKO TNF+Z	-75	***
TRADD-KO TNF+C+Z vs TRADD-RIPK1-DKO TNF+N	-72	***
TRADD-KO TNF+C+Z vs TRADD-RIPK1-DKO TNF+Z+N	-70	***
TRADD-KO TNF+C+Z vs TRADD-RIPK1-DKO TNF+C	-65	***
TRADD-KO TNF+C+Z vs TRADD-RIPK1-DKO TNF+C+Z	-75	***
TRADD-KO TNF+C+Z vs TRADD-RIPK1-DKO TNF+C+N	-67	***
TRADD-KO TNF+C+Z vs TRADD-RIPK1-DKO TNF+C+Z+N	-76	***
TRADD-KO TNF+C+Z vs Casp.8-KO TNF	-66	***
TRADD-KO TNF+C+Z vs Casp.8-KO TNF+Z	-69	***
TRADD-KO TNF+C+Z vs Casp.8-KO TNF+N	-76	***
TRADD-KO TNF+C+Z vs Casp.8-KO TNF+Z+N	-78	***
TRADD-KO TNF+C+Z vs Casp.8-KO TNF+C	-40	***
TRADD-KO TNF+C+Z vs Casp.8-KO TNF+C+Z	-41	***
TRADD-KO TNF+C+Z vs Casp.8-KO TNF+C+N	-76	***
TRADD-KO TNF+C+Z vs Casp.8-KO TNF+C+Z+N	-74	***
TRADD-KO TNF+C+Z vs EV TNF	-64	***
TRADD-KO TNF+C+Z vs EV TNF+Z	-72	***
TRADD-KO TNF+C+Z vs EV TNF+N	-62	***
TRADD-KO TNF+C+Z vs EV TNF+Z+N	-70	***
TRADD-KO TNF+C+Z vs EV TNF+C	-0,46	ns
TRADD-KO TNF+C+Z vs EV TNF+C+Z	-76	***
TRADD-KO TNF+C+Z vs EV TNF+C+N	-12	ns
TRADD-KO TNF+C+Z vs EV TNF+C+Z+N	-75	***
TRADD-KO TNF+C+N vs TRADD-KO TNF+C+Z+N	-67	***
TRADD-KO TNF+C+N vs RIPK1-KO TNF	-70	***
TRADD-KO TNF+C+N vs RIPK1-KO TNF+Z	-72	***
TRADD-KO TNF+C+N vs RIPK1-KO TNF+N	-63	***
TRADD-KO TNF+C+N vs RIPK1-KO TNF+Z+N	-70	***
TRADD-KO TNF+C+N vs RIPK1-KO TNF+C	6,4	ns
TRADD-KO TNF+C+N vs RIPK1-KO TNF+C+Z	-69	***
TRADD-KO TNF+C+N vs RIPK1-KO TNF+C+N	-2,0	ns
TRADD-KO TNF+C+N vs RIPK1-KO TNF+C+Z+N	-70	***
TRADD-KO TNF+C+N vs FADD-TRADD-DKO TNF	-60	***
TRADD-KO TNF+C+N vs FADD-TRADD-DKO TNF+Z	-56	***
TRADD-KO TNF+C+N vs FADD-TRADD-DKO TNF+N	-62	***
TRADD-KO TNF+C+N vs FADD-TRADD-DKO TNF+Z+N	-69	***
TRADD-KO TNF+C+N vs FADD-TRADD-DKO TNF+C	22	ns
TRADD-KO TNF+C+N vs FADD-TRADD-DKO TNF+C+Z	18	ns
TRADD-KO TNF+C+N vs FADD-TRADD-DKO TNF+C+N	-40	***
TRADD-KO TNF+C+N vs FADD-TRADD-DKO TNF+C+Z+N	-66	***
TRADD-KO TNF+C+N vs FADD-RIPK1-DKO TNF	-71	***
TRADD-KO TNF+C+N vs FADD-RIPK1-DKO TNF+Z	-72	***
TRADD-KO TNF+C+N vs FADD-RIPK1-DKO TNF+N	-66	***
TRADD-KO TNF+C+N vs FADD-RIPK1-DKO TNF+Z+N	-69	***
TRADD-KO TNF+C+N vs FADD-RIPK1-DKO TNF+C	-66	***
TRADD-KO TNF+C+N vs FADD-RIPK1-DKO TNF+C+Z	-64	***
TRADD-KO TNF+C+N vs FADD-RIPK1-DKO TNF+C+N	-62	***
TRADD-KO TNF+C+N vs FADD-RIPK1-DKO TNF+C+Z+N	-70	***
TRADD-KO TNF+C+N vs TRADD-RIPK1-DKO TNF	-69	***
TRADD-KO TNF+C+N vs TRADD-RIPK1-DKO TNF+Z	-69	***
TRADD-KO TNF+C+N vs TRADD-RIPK1-DKO TNF+N	-66	***
TRADD-KO TNF+C+N vs TRADD-RIPK1-DKO TNF+Z+N	-64	***
TRADD-KO TNF+C+N vs TRADD-RIPK1-DKO TNF+C	-59	***
TRADD-KO TNF+C+N vs TRADD-RIPK1-DKO TNF+C+Z	-69	***
TRADD-KO TNF+C+N vs TRADD-RIPK1-DKO TNF+C+N	-61	***
TRADD-KO TNF+C+N vs TRADD-RIPK1-DKO TNF+C+Z+N	-70	***

TRADD-KO TNF+C+N vs Casp.8-KO TNF	-60	***
TRADD-KO TNF+C+N vs Casp.8-KO TNF+Z	-63	***
TRADD-KO TNF+C+N vs Casp.8-KO TNF+N	-70	***
TRADD-KO TNF+C+N vs Casp.8-KO TNF+Z+N	-72	***
TRADD-KO TNF+C+N vs Casp.8-KO TNF+C	-34	***
TRADD-KO TNF+C+N vs Casp.8-KO TNF+C+Z	-35	***
TRADD-KO TNF+C+N vs Casp.8-KO TNF+C+N	-70	***
TRADD-KO TNF+C+N vs Casp.8-KO TNF+C+Z+N	-68	***
TRADD-KO TNF+C+N vs EV TNF	-58	***
TRADD-KO TNF+C+N vs EV TNF+Z	-66	***
TRADD-KO TNF+C+N vs EV TNF+N	-56	***
TRADD-KO TNF+C+N vs EV TNF+Z+N	-64	***
TRADD-KO TNF+C+N vs EV TNF+C	5,5	ns
TRADD-KO TNF+C+N vs EV TNF+C+Z	-70	***
TRADD-KO TNF+C+N vs EV TNF+C+N	-5,8	ns
TRADD-KO TNF+C+N vs EV TNF+C+Z+N	-69	***
TRADD-KO TNF+C+Z+N vs RIPK1-KO TNF	-3,4	ns
TRADD-KO TNF+C+Z+N vs RIPK1-KO TNF+Z	-5,8	ns
TRADD-KO TNF+C+Z+N vs RIPK1-KO TNF+N	3,4	ns
TRADD-KO TNF+C+Z+N vs RIPK1-KO TNF+Z+N	-3,0	ns
TRADD-KO TNF+C+Z+N vs RIPK1-KO TNF+C	73	***
TRADD-KO TNF+C+Z+N vs RIPK1-KO TNF+C+Z	-2,2	ns
TRADD-KO TNF+C+Z+N vs RIPK1-KO TNF+C+N	65	***
TRADD-KO TNF+C+Z+N vs RIPK1-KO TNF+C+Z+N	-3,6	ns
TRADD-KO TNF+C+Z+N vs FADD-TRADD-DKO TNF	6,3	ns
TRADD-KO TNF+C+Z+N vs FADD-TRADD-DKO TNF+Z	11	ns
TRADD-KO TNF+C+Z+N vs FADD-TRADD-DKO TNF+N	5,1	ns
TRADD-KO TNF+C+Z+N vs FADD-TRADD-DKO TNF+Z+N	-2,7	ns
TRADD-KO TNF+C+Z+N vs FADD-TRADD-DKO TNF+C	89	***
TRADD-KO TNF+C+Z+N vs FADD-TRADD-DKO TNF+C+Z	84	***
TRADD-KO TNF+C+Z+N vs FADD-TRADD-DKO TNF+C+N	27	***
TRADD-KO TNF+C+Z+N vs FADD-TRADD-DKO TNF+C+Z+N	0,76	ns
TRADD-KO TNF+C+Z+N vs FADD-RIPK1-DKO TNF	-4,8	ns
TRADD-KO TNF+C+Z+N vs FADD-RIPK1-DKO TNF+Z	-5,4	ns
TRADD-KO TNF+C+Z+N vs FADD-RIPK1-DKO TNF+N	0,34	ns
TRADD-KO TNF+C+Z+N vs FADD-RIPK1-DKO TNF+Z+N	-1,9	ns
TRADD-KO TNF+C+Z+N vs FADD-RIPK1-DKO TNF+C	1,1	ns
TRADD-KO TNF+C+Z+N vs FADD-RIPK1-DKO TNF+C+Z	3,1	ns
TRADD-KO TNF+C+Z+N vs FADD-RIPK1-DKO TNF+C+N	5,0	ns
TRADD-KO TNF+C+Z+N vs FADD-RIPK1-DKO TNF+C+Z+N	-3,0	ns
TRADD-KO TNF+C+Z+N vs TRADD-RIPK1-DKO TNF	-2,2	ns
TRADD-KO TNF+C+Z+N vs TRADD-RIPK1-DKO TNF+Z	-2,4	ns
TRADD-KO TNF+C+Z+N vs TRADD-RIPK1-DKO TNF+N	1,1	ns
TRADD-KO TNF+C+Z+N vs TRADD-RIPK1-DKO TNF+Z+N	2,8	ns
TRADD-KO TNF+C+Z+N vs TRADD-RIPK1-DKO TNF+C	7,6	ns
TRADD-KO TNF+C+Z+N vs TRADD-RIPK1-DKO TNF+C+Z	-2,2	ns
TRADD-KO TNF+C+Z+N vs TRADD-RIPK1-DKO TNF+C+N	5,4	ns
TRADD-KO TNF+C+Z+N vs TRADD-RIPK1-DKO TNF+C+Z+N	-2,9	ns
TRADD-KO TNF+C+Z+N vs Casp.8-KO TNF	6,4	ns
TRADD-KO TNF+C+Z+N vs Casp.8-KO TNF+Z	4,2	ns
TRADD-KO TNF+C+Z+N vs Casp.8-KO TNF+N	-3,2	ns
TRADD-KO TNF+C+Z+N vs Casp.8-KO TNF+Z+N	-4,8	ns
TRADD-KO TNF+C+Z+N vs Casp.8-KO TNF+C	33	***
TRADD-KO TNF+C+Z+N vs Casp.8-KO TNF+C+Z	32	***
TRADD-KO TNF+C+Z+N vs Casp.8-KO TNF+C+N	-3,0	ns
TRADD-KO TNF+C+Z+N vs Casp.8-KO TNF+C+Z+N	-1,5	ns
TRADD-KO TNF+C+Z+N vs EV TNF	8,5	ns
TRADD-KO TNF+C+Z+N vs EV TNF+Z	0,27	ns
TRADD-KO TNF+C+Z+N vs EV TNF+N	11	ns
TRADD-KO TNF+C+Z+N vs EV TNF+Z+N	2,3	ns
TRADD-KO TNF+C+Z+N vs EV TNF+C	72	***
TRADD-KO TNF+C+Z+N vs EV TNF+C+Z	-3,8	ns
TRADD-KO TNF+C+Z+N vs EV TNF+C+N	61	***
TRADD-KO TNF+C+Z+N vs EV TNF+C+Z+N	-2,3	ns
RIPK1-KO TNF vs RIPK1-KO TNF+Z	-2,4	ns
RIPK1-KO TNF vs RIPK1-KO TNF+N	6,9	ns

RIPK1-KO TNF vs RIPK1-KO TNF+Z+N	0,43	ns
RIPK1-KO TNF vs RIPK1-KO TNF+C	77	***
RIPK1-KO TNF vs RIPK1-KO TNF+C+Z	1,2	ns
RIPK1-KO TNF vs RIPK1-KO TNF+C+N	68	***
RIPK1-KO TNF vs RIPK1-KO TNF+C+Z+N	-0,16	ns
RIPK1-KO TNF vs FADD-TRADD-DKO TNF	9,8	ns
RIPK1-KO TNF vs FADD-TRADD-DKO TNF+Z	14	*
RIPK1-KO TNF vs FADD-TRADD-DKO TNF+N	8,6	ns
RIPK1-KO TNF vs FADD-TRADD-DKO TNF+Z+N	0,75	ns
RIPK1-KO TNF vs FADD-TRADD-DKO TNF+C	92	***
RIPK1-KO TNF vs FADD-TRADD-DKO TNF+C+Z	88	***
RIPK1-KO TNF vs FADD-TRADD-DKO TNF+C+N	30	***
RIPK1-KO TNF vs FADD-TRADD-DKO TNF+C+Z+N	4,2	ns
RIPK1-KO TNF vs FADD-RIPK1-DKO TNF	-1,4	ns
RIPK1-KO TNF vs FADD-RIPK1-DKO TNF+Z	-1,9	ns
RIPK1-KO TNF vs FADD-RIPK1-DKO TNF+N	3,8	ns
RIPK1-KO TNF vs FADD-RIPK1-DKO TNF+Z+N	1,6	ns
RIPK1-KO TNF vs FADD-RIPK1-DKO TNF+C	4,6	ns
RIPK1-KO TNF vs FADD-RIPK1-DKO TNF+C+Z	6,6	ns
RIPK1-KO TNF vs FADD-RIPK1-DKO TNF+C+N	8,5	ns
RIPK1-KO TNF vs FADD-RIPK1-DKO TNF+C+Z+N	0,47	ns
RIPK1-KO TNF vs TRADD-RIPK1-DKO TNF	1,2	ns
RIPK1-KO TNF vs TRADD-RIPK1-DKO TNF+Z	0,99	ns
RIPK1-KO TNF vs TRADD-RIPK1-DKO TNF+N	4,6	ns
RIPK1-KO TNF vs TRADD-RIPK1-DKO TNF+Z+N	6,2	ns
RIPK1-KO TNF vs TRADD-RIPK1-DKO TNF+C	11	ns
RIPK1-KO TNF vs TRADD-RIPK1-DKO TNF+C+Z	1,3	ns
RIPK1-KO TNF vs TRADD-RIPK1-DKO TNF+C+N	8,8	ns
RIPK1-KO TNF vs TRADD-RIPK1-DKO TNF+C+Z+N	0,57	ns
RIPK1-KO TNF vs Casp.8-KO TNF	9,9	ns
RIPK1-KO TNF vs Casp.8-KO TNF+Z	7,6	ns
RIPK1-KO TNF vs Casp.8-KO TNF+N	0,21	ns
RIPK1-KO TNF vs Casp.8-KO TNF+Z+N	-1,4	ns
RIPK1-KO TNF vs Casp.8-KO TNF+C	36	***
RIPK1-KO TNF vs Casp.8-KO TNF+C+Z	35	***
RIPK1-KO TNF vs Casp.8-KO TNF+C+N	0,44	ns
RIPK1-KO TNF vs Casp.8-KO TNF+C+Z+N	1,9	ns
RIPK1-KO TNF vs EV TNF	12	ns
RIPK1-KO TNF vs EV TNF+Z	3,7	ns
RIPK1-KO TNF vs EV TNF+N	14	ns
RIPK1-KO TNF vs EV TNF+Z+N	5,7	ns
RIPK1-KO TNF vs EV TNF+C	76	***
RIPK1-KO TNF vs EV TNF+C+Z	-0,32	ns
RIPK1-KO TNF vs EV TNF+C+N	64	***
RIPK1-KO TNF vs EV TNF+C+Z+N	1,1	ns
RIPK1-KO TNF+Z vs RIPK1-KO TNF+N	9,2	ns
RIPK1-KO TNF+Z vs RIPK1-KO TNF+Z+N	2,8	ns
RIPK1-KO TNF+Z vs RIPK1-KO TNF+C	79	***
RIPK1-KO TNF+Z vs RIPK1-KO TNF+C+Z	3,6	ns
RIPK1-KO TNF+Z vs RIPK1-KO TNF+C+N	70	***
RIPK1-KO TNF+Z vs RIPK1-KO TNF+C+Z+N	2,2	ns
RIPK1-KO TNF+Z vs FADD-TRADD-DKO TNF	12	ns
RIPK1-KO TNF+Z vs FADD-TRADD-DKO TNF+Z	16	**
RIPK1-KO TNF+Z vs FADD-TRADD-DKO TNF+N	11	ns
RIPK1-KO TNF+Z vs FADD-TRADD-DKO TNF+Z+N	3,1	ns
RIPK1-KO TNF+Z vs FADD-TRADD-DKO TNF+C	95	***
RIPK1-KO TNF+Z vs FADD-TRADD-DKO TNF+C+Z	90	***
RIPK1-KO TNF+Z vs FADD-TRADD-DKO TNF+C+N	32	***
RIPK1-KO TNF+Z vs FADD-TRADD-DKO TNF+C+Z+N	6,6	ns
RIPK1-KO TNF+Z vs FADD-RIPK1-DKO TNF	1,0	ns
RIPK1-KO TNF+Z vs FADD-RIPK1-DKO TNF+Z	0,44	ns
RIPK1-KO TNF+Z vs FADD-RIPK1-DKO TNF+N	6,1	ns
RIPK1-KO TNF+Z vs FADD-RIPK1-DKO TNF+Z+N	3,9	ns
RIPK1-KO TNF+Z vs FADD-RIPK1-DKO TNF+C	6,9	ns
RIPK1-KO TNF+Z vs FADD-RIPK1-DKO TNF+C+Z	8,9	ns
RIPK1-KO TNF+Z vs FADD-RIPK1-DKO TNF+C+N	11	ns

RIPK1-KO TNF+Z vs FADD-RIPK1-DKO TNF+C+Z+N	2,8	ns
RIPK1-KO TNF+Z vs TRADD-RIPK1-DKO TNF	3,6	ns
RIPK1-KO TNF+Z vs TRADD-RIPK1-DKO TNF+Z	3,3	ns
RIPK1-KO TNF+Z vs TRADD-RIPK1-DKO TNF+N	6,9	ns
RIPK1-KO TNF+Z vs TRADD-RIPK1-DKO TNF+Z+N	8,6	ns
RIPK1-KO TNF+Z vs TRADD-RIPK1-DKO TNF+C	13	**
RIPK1-KO TNF+Z vs TRADD-RIPK1-DKO TNF+C+Z	3,6	ns
RIPK1-KO TNF+Z vs TRADD-RIPK1-DKO TNF+C+N	11	ns
RIPK1-KO TNF+Z vs TRADD-RIPK1-DKO TNF+C+Z+N	2,9	ns
RIPK1-KO TNF+Z vs Casp.8-KO TNF	12	ns
RIPK1-KO TNF+Z vs Casp.8-KO TNF+Z	10	ns
RIPK1-KO TNF+Z vs Casp.8-KO TNF+N	2,6	ns
RIPK1-KO TNF+Z vs Casp.8-KO TNF+Z+N	0,99	ns
RIPK1-KO TNF+Z vs Casp.8-KO TNF+C	39	***
RIPK1-KO TNF+Z vs Casp.8-KO TNF+C+Z	37	***
RIPK1-KO TNF+Z vs Casp.8-KO TNF+C+N	2,8	ns
RIPK1-KO TNF+Z vs Casp.8-KO TNF+C+Z+N	4,3	ns
RIPK1-KO TNF+Z vs EV TNF	14	ns
RIPK1-KO TNF+Z vs EV TNF+Z	6,1	ns
RIPK1-KO TNF+Z vs EV TNF+N	16	ns
RIPK1-KO TNF+Z vs EV TNF+Z+N	8,1	ns
RIPK1-KO TNF+Z vs EV TNF+C	78	***
RIPK1-KO TNF+Z vs EV TNF+C+Z	2,0	ns
RIPK1-KO TNF+Z vs EV TNF+C+N	67	***
RIPK1-KO TNF+Z vs EV TNF+C+Z+N	3,5	ns
RIPK1-KO TNF+N vs RIPK1-KO TNF+Z+N	-6,4	ns
RIPK1-KO TNF+N vs RIPK1-KO TNF+C	70	***
RIPK1-KO TNF+N vs RIPK1-KO TNF+C+Z	-5,6	ns
RIPK1-KO TNF+N vs RIPK1-KO TNF+C+N	61	***
RIPK1-KO TNF+N vs RIPK1-KO TNF+C+Z+N	-7,0	ns
RIPK1-KO TNF+N vs FADD-TRADD-DKO TNF	2,9	ns
RIPK1-KO TNF+N vs FADD-TRADD-DKO TNF+Z	7,3	ns
RIPK1-KO TNF+N vs FADD-TRADD-DKO TNF+N	1,7	ns
RIPK1-KO TNF+N vs FADD-TRADD-DKO TNF+Z+N	-6,1	ns
RIPK1-KO TNF+N vs FADD-TRADD-DKO TNF+C	86	***
RIPK1-KO TNF+N vs FADD-TRADD-DKO TNF+C+Z	81	***
RIPK1-KO TNF+N vs FADD-TRADD-DKO TNF+C+N	23	***
RIPK1-KO TNF+N vs FADD-TRADD-DKO TNF+C+Z+N	-2,7	ns
RIPK1-KO TNF+N vs FADD-RIPK1-DKO TNF	-8,2	ns
RIPK1-KO TNF+N vs FADD-RIPK1-DKO TNF+Z	-8,8	ns
RIPK1-KO TNF+N vs FADD-RIPK1-DKO TNF+N	-3,1	ns
RIPK1-KO TNF+N vs FADD-RIPK1-DKO TNF+Z+N	-5,3	ns
RIPK1-KO TNF+N vs FADD-RIPK1-DKO TNF+C	-2,3	ns
RIPK1-KO TNF+N vs FADD-RIPK1-DKO TNF+C+Z	-0,28	ns
RIPK1-KO TNF+N vs FADD-RIPK1-DKO TNF+C+N	1,6	ns
RIPK1-KO TNF+N vs FADD-RIPK1-DKO TNF+C+Z+N	-6,4	ns
RIPK1-KO TNF+N vs TRADD-RIPK1-DKO TNF	-5,6	ns
RIPK1-KO TNF+N vs TRADD-RIPK1-DKO TNF+Z	-5,9	ns
RIPK1-KO TNF+N vs TRADD-RIPK1-DKO TNF+N	-2,3	ns
RIPK1-KO TNF+N vs TRADD-RIPK1-DKO TNF+Z+N	-0,66	ns
RIPK1-KO TNF+N vs TRADD-RIPK1-DKO TNF+C	4,2	ns
RIPK1-KO TNF+N vs TRADD-RIPK1-DKO TNF+C+Z	-5,6	ns
RIPK1-KO TNF+N vs TRADD-RIPK1-DKO TNF+C+N	1,9	ns
RIPK1-KO TNF+N vs TRADD-RIPK1-DKO TNF+C+Z+N	-6,3	ns
RIPK1-KO TNF+N vs Casp.8-KO TNF	3,0	ns
RIPK1-KO TNF+N vs Casp.8-KO TNF+Z	0,76	ns
RIPK1-KO TNF+N vs Casp.8-KO TNF+N	-6,6	ns
RIPK1-KO TNF+N vs Casp.8-KO TNF+Z+N	-8,2	ns
RIPK1-KO TNF+N vs Casp.8-KO TNF+C	29	***
RIPK1-KO TNF+N vs Casp.8-KO TNF+C+Z	28	***
RIPK1-KO TNF+N vs Casp.8-KO TNF+C+N	-6,4	ns
RIPK1-KO TNF+N vs Casp.8-KO TNF+C+Z+N	-4,9	ns
RIPK1-KO TNF+N vs EV TNF	5,1	ns
RIPK1-KO TNF+N vs EV TNF+Z	-3,1	ns
RIPK1-KO TNF+N vs EV TNF+N	7,2	ns
RIPK1-KO TNF+N vs EV TNF+Z+N	-1,1	ns

RIPK1-KO TNF+N vs EV TNF+C	69	***
RIPK1-KO TNF+N vs EV TNF+C+Z	-7,2	ns
RIPK1-KO TNF+N vs EV TNF+C+N	58	***
RIPK1-KO TNF+N vs EV TNF+C+Z+N	-5,8	ns
RIPK1-KO TNF+Z+N vs RIPK1-KO TNF+C	76	***
RIPK1-KO TNF+Z+N vs RIPK1-KO TNF+C+Z	0,81	ns
RIPK1-KO TNF+Z+N vs RIPK1-KO TNF+C+N	68	***
RIPK1-KO TNF+Z+N vs RIPK1-KO TNF+C+Z+N	-0,59	ns
RIPK1-KO TNF+Z+N vs FADD-TRADD-DKO TNF	9,3	ns
RIPK1-KO TNF+Z+N vs FADD-TRADD-DKO TNF+Z	14	ns
RIPK1-KO TNF+Z+N vs FADD-TRADD-DKO TNF+N	8,1	ns
RIPK1-KO TNF+Z+N vs FADD-TRADD-DKO TNF+Z+N	0,32	ns
RIPK1-KO TNF+Z+N vs FADD-TRADD-DKO TNF+C	92	***
RIPK1-KO TNF+Z+N vs FADD-TRADD-DKO TNF+C+Z	87	***
RIPK1-KO TNF+Z+N vs FADD-TRADD-DKO TNF+C+N	30	***
RIPK1-KO TNF+Z+N vs FADD-TRADD-DKO TNF+C+Z+N	3,8	ns
RIPK1-KO TNF+Z+N vs FADD-RIPK1-DKO TNF	-1,8	ns
RIPK1-KO TNF+Z+N vs FADD-RIPK1-DKO TNF+Z	-2,4	ns
RIPK1-KO TNF+Z+N vs FADD-RIPK1-DKO TNF+N	3,3	ns
RIPK1-KO TNF+Z+N vs FADD-RIPK1-DKO TNF+Z+N	1,1	ns
RIPK1-KO TNF+Z+N vs FADD-RIPK1-DKO TNF+C	4,1	ns
RIPK1-KO TNF+Z+N vs FADD-RIPK1-DKO TNF+C+Z	6,1	ns
RIPK1-KO TNF+Z+N vs FADD-RIPK1-DKO TNF+C+N	8,0	ns
RIPK1-KO TNF+Z+N vs FADD-RIPK1-DKO TNF+C+Z+N	0,032	ns
RIPK1-KO TNF+Z+N vs TRADD-RIPK1-DKO TNF	0,78	ns
RIPK1-KO TNF+Z+N vs TRADD-RIPK1-DKO TNF+Z	0,55	ns
RIPK1-KO TNF+Z+N vs TRADD-RIPK1-DKO TNF+N	4,1	ns
RIPK1-KO TNF+Z+N vs TRADD-RIPK1-DKO TNF+Z+N	5,8	ns
RIPK1-KO TNF+Z+N vs TRADD-RIPK1-DKO TNF+C	11	ns
RIPK1-KO TNF+Z+N vs TRADD-RIPK1-DKO TNF+C+Z	0,82	ns
RIPK1-KO TNF+Z+N vs TRADD-RIPK1-DKO TNF+C+N	8,4	ns
RIPK1-KO TNF+Z+N vs TRADD-RIPK1-DKO TNF+C+Z+N	0,14	ns
RIPK1-KO TNF+Z+N vs Casp.8-KO TNF	9,4	ns
RIPK1-KO TNF+Z+N vs Casp.8-KO TNF+Z	7,2	ns
RIPK1-KO TNF+Z+N vs Casp.8-KO TNF+N	-0,23	ns
RIPK1-KO TNF+Z+N vs Casp.8-KO TNF+Z+N	-1,8	ns
RIPK1-KO TNF+Z+N vs Casp.8-KO TNF+C	36	***
RIPK1-KO TNF+Z+N vs Casp.8-KO TNF+C+Z	35	***
RIPK1-KO TNF+Z+N vs Casp.8-KO TNF+C+N	0,0078	ns
RIPK1-KO TNF+Z+N vs Casp.8-KO TNF+C+Z+N	1,5	ns
RIPK1-KO TNF+Z+N vs EV TNF	12	ns
RIPK1-KO TNF+Z+N vs EV TNF+Z	3,3	ns
RIPK1-KO TNF+Z+N vs EV TNF+N	14	ns
RIPK1-KO TNF+Z+N vs EV TNF+Z+N	5,3	ns
RIPK1-KO TNF+Z+N vs EV TNF+C	75	***
RIPK1-KO TNF+Z+N vs EV TNF+C+Z	-0,76	ns
RIPK1-KO TNF+Z+N vs EV TNF+C+N	64	***
RIPK1-KO TNF+Z+N vs EV TNF+C+Z+N	0,66	ns
RIPK1-KO TNF+C vs RIPK1-KO TNF+C+Z	-75	***
RIPK1-KO TNF+C vs RIPK1-KO TNF+C+N	-8,4	ns
RIPK1-KO TNF+C vs RIPK1-KO TNF+C+Z+N	-77	***
RIPK1-KO TNF+C vs FADD-TRADD-DKO TNF	-67	***
RIPK1-KO TNF+C vs FADD-TRADD-DKO TNF+Z	-62	***
RIPK1-KO TNF+C vs FADD-TRADD-DKO TNF+N	-68	***
RIPK1-KO TNF+C vs FADD-TRADD-DKO TNF+Z+N	-76	***
RIPK1-KO TNF+C vs FADD-TRADD-DKO TNF+C	16	ns
RIPK1-KO TNF+C vs FADD-TRADD-DKO TNF+C+Z	11	ns
RIPK1-KO TNF+C vs FADD-TRADD-DKO TNF+C+N	-47	***
RIPK1-KO TNF+C vs FADD-TRADD-DKO TNF+C+Z+N	-72	***
RIPK1-KO TNF+C vs FADD-RIPK1-DKO TNF	-78	***
RIPK1-KO TNF+C vs FADD-RIPK1-DKO TNF+Z	-78	***
RIPK1-KO TNF+C vs FADD-RIPK1-DKO TNF+N	-73	***
RIPK1-KO TNF+C vs FADD-RIPK1-DKO TNF+Z+N	-75	***
RIPK1-KO TNF+C vs FADD-RIPK1-DKO TNF+C	-72	***
RIPK1-KO TNF+C vs FADD-RIPK1-DKO TNF+C+Z	-70	***
RIPK1-KO TNF+C vs FADD-RIPK1-DKO TNF+C+N	-68	***

RIPK1-KO TNF+C vs FADD-RIPK1-DKO TNF+C+Z+N	-76	***
RIPK1-KO TNF+C vs TRADD-RIPK1-DKO TNF	-75	***
RIPK1-KO TNF+C vs TRADD-RIPK1-DKO TNF+Z	-76	***
RIPK1-KO TNF+C vs TRADD-RIPK1-DKO TNF+N	-72	***
RIPK1-KO TNF+C vs TRADD-RIPK1-DKO TNF+Z+N	-70	***
RIPK1-KO TNF+C vs TRADD-RIPK1-DKO TNF+C	-65	***
RIPK1-KO TNF+C vs TRADD-RIPK1-DKO TNF+C+Z	-75	***
RIPK1-KO TNF+C vs TRADD-RIPK1-DKO TNF+C+N	-68	***
RIPK1-KO TNF+C vs TRADD-RIPK1-DKO TNF+C+Z+N	-76	***
RIPK1-KO TNF+C vs Casp.8-KO TNF	-67	***
RIPK1-KO TNF+C vs Casp.8-KO TNF+Z	-69	***
RIPK1-KO TNF+C vs Casp.8-KO TNF+N	-76	***
RIPK1-KO TNF+C vs Casp.8-KO TNF+Z+N	-78	***
RIPK1-KO TNF+C vs Casp.8-KO TNF+C	-40	***
RIPK1-KO TNF+C vs Casp.8-KO TNF+C+Z	-42	***
RIPK1-KO TNF+C vs Casp.8-KO TNF+C+N	-76	***
RIPK1-KO TNF+C vs Casp.8-KO TNF+C+Z+N	-75	***
RIPK1-KO TNF+C vs EV TNF	-65	***
RIPK1-KO TNF+C vs EV TNF+Z	-73	***
RIPK1-KO TNF+C vs EV TNF+N	-62	***
RIPK1-KO TNF+C vs EV TNF+Z+N	-71	***
RIPK1-KO TNF+C vs EV TNF+C	-0,86	ns
RIPK1-KO TNF+C vs EV TNF+C+Z	-77	***
RIPK1-KO TNF+C vs EV TNF+C+N	-12	ns
RIPK1-KO TNF+C vs EV TNF+C+Z+N	-75	***
RIPK1-KO TNF+C+Z vs RIPK1-KO TNF+C+N	67	***
RIPK1-KO TNF+C+Z vs RIPK1-KO TNF+C+Z+N	-1,4	ns
RIPK1-KO TNF+C+Z vs FADD-TRADD-DKO TNF	8,5	ns
RIPK1-KO TNF+C+Z vs FADD-TRADD-DKO TNF+Z	13	ns
RIPK1-KO TNF+C+Z vs FADD-TRADD-DKO TNF+N	7,3	ns
RIPK1-KO TNF+C+Z vs FADD-TRADD-DKO TNF+Z+N	-0,49	ns
RIPK1-KO TNF+C+Z vs FADD-TRADD-DKO TNF+C	91	***
RIPK1-KO TNF+C+Z vs FADD-TRADD-DKO TNF+C+Z	86	***
RIPK1-KO TNF+C+Z vs FADD-TRADD-DKO TNF+C+N	29	***
RIPK1-KO TNF+C+Z vs FADD-TRADD-DKO TNF+C+Z+N	3,0	ns
RIPK1-KO TNF+C+Z vs FADD-RIPK1-DKO TNF	-2,6	ns
RIPK1-KO TNF+C+Z vs FADD-RIPK1-DKO TNF+Z	-3,2	ns
RIPK1-KO TNF+C+Z vs FADD-RIPK1-DKO TNF+N	2,5	ns
RIPK1-KO TNF+C+Z vs FADD-RIPK1-DKO TNF+Z+N	0,33	ns
RIPK1-KO TNF+C+Z vs FADD-RIPK1-DKO TNF+C	3,3	ns
RIPK1-KO TNF+C+Z vs FADD-RIPK1-DKO TNF+C+Z	5,3	ns
RIPK1-KO TNF+C+Z vs FADD-RIPK1-DKO TNF+C+N	7,2	ns
RIPK1-KO TNF+C+Z vs FADD-RIPK1-DKO TNF+C+Z+N	-0,78	ns
RIPK1-KO TNF+C+Z vs TRADD-RIPK1-DKO TNF	-0,028	ns
RIPK1-KO TNF+C+Z vs TRADD-RIPK1-DKO TNF+Z	-0,26	ns
RIPK1-KO TNF+C+Z vs TRADD-RIPK1-DKO TNF+N	3,3	ns
RIPK1-KO TNF+C+Z vs TRADD-RIPK1-DKO TNF+Z+N	5,0	ns
RIPK1-KO TNF+C+Z vs TRADD-RIPK1-DKO TNF+C	9,8	ns
RIPK1-KO TNF+C+Z vs TRADD-RIPK1-DKO TNF+C+Z	0,0083	ns
RIPK1-KO TNF+C+Z vs TRADD-RIPK1-DKO TNF+C+N	7,6	ns
RIPK1-KO TNF+C+Z vs TRADD-RIPK1-DKO TNF+C+Z+N	-0,67	ns
RIPK1-KO TNF+C+Z vs Casp.8-KO TNF	8,6	ns
RIPK1-KO TNF+C+Z vs Casp.8-KO TNF+Z	6,4	ns
RIPK1-KO TNF+C+Z vs Casp.8-KO TNF+N	-1,0	ns
RIPK1-KO TNF+C+Z vs Casp.8-KO TNF+Z+N	-2,6	ns
RIPK1-KO TNF+C+Z vs Casp.8-KO TNF+C	35	***
RIPK1-KO TNF+C+Z vs Casp.8-KO TNF+C+Z	34	***
RIPK1-KO TNF+C+Z vs Casp.8-KO TNF+C+N	-0,80	ns
RIPK1-KO TNF+C+Z vs Casp.8-KO TNF+C+Z+N	0,67	ns
RIPK1-KO TNF+C+Z vs EV TNF	11	ns
RIPK1-KO TNF+C+Z vs EV TNF+Z	2,5	ns
RIPK1-KO TNF+C+Z vs EV TNF+N	13	ns
RIPK1-KO TNF+C+Z vs EV TNF+Z+N	4,5	ns
RIPK1-KO TNF+C+Z vs EV TNF+C	74	***
RIPK1-KO TNF+C+Z vs EV TNF+C+Z	-1,6	ns
RIPK1-KO TNF+C+Z vs EV TNF+C+N	63	***

RIPK1-KO TNF+C+Z vs EV TNF+C+Z+N	-0,15	ns
RIPK1-KO TNF+C+N vs RIPK1-KO TNF+C+Z+N	-68	***
RIPK1-KO TNF+C+N vs FADD-TRADD-DKO TNF	-58	***
RIPK1-KO TNF+C+N vs FADD-TRADD-DKO TNF+Z	-54	***
RIPK1-KO TNF+C+N vs FADD-TRADD-DKO TNF+N	-60	***
RIPK1-KO TNF+C+N vs FADD-TRADD-DKO TNF+Z+N	-67	***
RIPK1-KO TNF+C+N vs FADD-TRADD-DKO TNF+C	24	ns
RIPK1-KO TNF+C+N vs FADD-TRADD-DKO TNF+C+Z	20	ns
RIPK1-KO TNF+C+N vs FADD-TRADD-DKO TNF+C+N	-38	***
RIPK1-KO TNF+C+N vs FADD-TRADD-DKO TNF+C+Z+N	-64	***
RIPK1-KO TNF+C+N vs FADD-RIPK1-DKO TNF	-69	***
RIPK1-KO TNF+C+N vs FADD-RIPK1-DKO TNF+Z	-70	***
RIPK1-KO TNF+C+N vs FADD-RIPK1-DKO TNF+N	-64	***
RIPK1-KO TNF+C+N vs FADD-RIPK1-DKO TNF+Z+N	-67	***
RIPK1-KO TNF+C+N vs FADD-RIPK1-DKO TNF+C	-64	***
RIPK1-KO TNF+C+N vs FADD-RIPK1-DKO TNF+C+Z	-62	***
RIPK1-KO TNF+C+N vs FADD-RIPK1-DKO TNF+C+N	-60	***
RIPK1-KO TNF+C+N vs FADD-RIPK1-DKO TNF+C+Z+N	-68	***
RIPK1-KO TNF+C+N vs TRADD-RIPK1-DKO TNF	-67	***
RIPK1-KO TNF+C+N vs TRADD-RIPK1-DKO TNF+Z	-67	***
RIPK1-KO TNF+C+N vs TRADD-RIPK1-DKO TNF+N	-64	***
RIPK1-KO TNF+C+N vs TRADD-RIPK1-DKO TNF+Z+N	-62	***
RIPK1-KO TNF+C+N vs TRADD-RIPK1-DKO TNF+C	-57	***
RIPK1-KO TNF+C+N vs TRADD-RIPK1-DKO TNF+C+Z	-67	***
RIPK1-KO TNF+C+N vs TRADD-RIPK1-DKO TNF+C+N	-59	***
RIPK1-KO TNF+C+N vs TRADD-RIPK1-DKO TNF+C+Z+N	-68	***
RIPK1-KO TNF+C+N vs Casp.8-KO TNF	-58	***
RIPK1-KO TNF+C+N vs Casp.8-KO TNF+Z	-61	***
RIPK1-KO TNF+C+N vs Casp.8-KO TNF+N	-68	***
RIPK1-KO TNF+C+N vs Casp.8-KO TNF+Z+N	-70	***
RIPK1-KO TNF+C+N vs Casp.8-KO TNF+C	-32	***
RIPK1-KO TNF+C+N vs Casp.8-KO TNF+C+Z	-33	***
RIPK1-KO TNF+C+N vs Casp.8-KO TNF+C+N	-68	***
RIPK1-KO TNF+C+N vs Casp.8-KO TNF+C+Z+N	-66	***
RIPK1-KO TNF+C+N vs EV TNF	-56	***
RIPK1-KO TNF+C+N vs EV TNF+Z	-64	***
RIPK1-KO TNF+C+N vs EV TNF+N	-54	***
RIPK1-KO TNF+C+N vs EV TNF+Z+N	-62	***
RIPK1-KO TNF+C+N vs EV TNF+C	7,5	ns
RIPK1-KO TNF+C+N vs EV TNF+C+Z	-68	***
RIPK1-KO TNF+C+N vs EV TNF+C+N	-3,8	ns
RIPK1-KO TNF+C+N vs EV TNF+C+Z+N	-67	***
RIPK1-KO TNF+C+Z+N vs FADD-TRADD-DKO TNF	9,9	ns
RIPK1-KO TNF+C+Z+N vs FADD-TRADD-DKO TNF+Z	14	ns
RIPK1-KO TNF+C+Z+N vs FADD-TRADD-DKO TNF+N	8,7	ns
RIPK1-KO TNF+C+Z+N vs FADD-TRADD-DKO TNF+Z+N	0,91	ns
RIPK1-KO TNF+C+Z+N vs FADD-TRADD-DKO TNF+C	93	***
RIPK1-KO TNF+C+Z+N vs FADD-TRADD-DKO TNF+C+Z	88	***
RIPK1-KO TNF+C+Z+N vs FADD-TRADD-DKO TNF+C+N	30	***
RIPK1-KO TNF+C+Z+N vs FADD-TRADD-DKO TNF+C+Z+N	4,4	ns
RIPK1-KO TNF+C+Z+N vs FADD-RIPK1-DKO TNF	-1,2	ns
RIPK1-KO TNF+C+Z+N vs FADD-RIPK1-DKO TNF+Z	-1,8	ns
RIPK1-KO TNF+C+Z+N vs FADD-RIPK1-DKO TNF+N	3,9	ns
RIPK1-KO TNF+C+Z+N vs FADD-RIPK1-DKO TNF+Z+N	1,7	ns
RIPK1-KO TNF+C+Z+N vs FADD-RIPK1-DKO TNF+C	4,7	ns
RIPK1-KO TNF+C+Z+N vs FADD-RIPK1-DKO TNF+C+Z	6,7	ns
RIPK1-KO TNF+C+Z+N vs FADD-RIPK1-DKO TNF+C+N	8,6	ns
RIPK1-KO TNF+C+Z+N vs FADD-RIPK1-DKO TNF+C+Z+N	0,62	ns
RIPK1-KO TNF+C+Z+N vs TRADD-RIPK1-DKO TNF	1,4	ns
RIPK1-KO TNF+C+Z+N vs TRADD-RIPK1-DKO TNF+Z	1,1	ns
RIPK1-KO TNF+C+Z+N vs TRADD-RIPK1-DKO TNF+N	4,7	ns
RIPK1-KO TNF+C+Z+N vs TRADD-RIPK1-DKO TNF+Z+N	6,4	ns
RIPK1-KO TNF+C+Z+N vs TRADD-RIPK1-DKO TNF+C	11	ns
RIPK1-KO TNF+C+Z+N vs TRADD-RIPK1-DKO TNF+C+Z	1,4	ns
RIPK1-KO TNF+C+Z+N vs TRADD-RIPK1-DKO TNF+C+N	9,0	ns
RIPK1-KO TNF+C+Z+N vs TRADD-RIPK1-DKO TNF+C+Z+N	0,73	ns

RIPK1-KO TNF+C+Z+N vs Casp.8-KO TNF	10	ns
RIPK1-KO TNF+C+Z+N vs Casp.8-KO TNF+Z	7,8	ns
RIPK1-KO TNF+C+Z+N vs Casp.8-KO TNF+N	0,37	ns
RIPK1-KO TNF+C+Z+N vs Casp.8-KO TNF+Z+N	-1,2	ns
RIPK1-KO TNF+C+Z+N vs Casp.8-KO TNF+C	36	***
RIPK1-KO TNF+C+Z+N vs Casp.8-KO TNF+C+Z	35	***
RIPK1-KO TNF+C+Z+N vs Casp.8-KO TNF+C+N	0,60	ns
RIPK1-KO TNF+C+Z+N vs Casp.8-KO TNF+C+Z+N	2,1	ns
RIPK1-KO TNF+C+Z+N vs EV TNF	12	ns
RIPK1-KO TNF+C+Z+N vs EV TNF+Z	3,9	ns
RIPK1-KO TNF+C+Z+N vs EV TNF+N	14	ns
RIPK1-KO TNF+C+Z+N vs EV TNF+Z+N	5,9	ns
RIPK1-KO TNF+C+Z+N vs EV TNF+C	76	***
RIPK1-KO TNF+C+Z+N vs EV TNF+C+Z	-0,17	ns
RIPK1-KO TNF+C+Z+N vs EV TNF+C+N	65	***
RIPK1-KO TNF+C+Z+N vs EV TNF+C+Z+N	1,2	ns
FADD-TRADD-DKO TNF vs FADD-TRADD-DKO TNF+Z	4,4	ns
FADD-TRADD-DKO TNF vs FADD-TRADD-DKO TNF+N	-1,2	ns
FADD-TRADD-DKO TNF vs FADD-TRADD-DKO TNF+Z+N	-9,0	ns
FADD-TRADD-DKO TNF vs FADD-TRADD-DKO TNF+C	83	***
FADD-TRADD-DKO TNF vs FADD-TRADD-DKO TNF+C+Z	78	***
FADD-TRADD-DKO TNF vs FADD-TRADD-DKO TNF+C+N	20	***
FADD-TRADD-DKO TNF vs FADD-TRADD-DKO TNF+C+Z+N	-5,6	ns
FADD-TRADD-DKO TNF vs FADD-RIPK1-DKO TNF	-11	ns
FADD-TRADD-DKO TNF vs FADD-RIPK1-DKO TNF+Z	-12	ns
FADD-TRADD-DKO TNF vs FADD-RIPK1-DKO TNF+N	-6,0	ns
FADD-TRADD-DKO TNF vs FADD-RIPK1-DKO TNF+Z+N	-8,2	ns
FADD-TRADD-DKO TNF vs FADD-RIPK1-DKO TNF+C	-5,2	ns
FADD-TRADD-DKO TNF vs FADD-RIPK1-DKO TNF+C+Z	-3,2	ns
FADD-TRADD-DKO TNF vs FADD-RIPK1-DKO TNF+C+N	-1,3	ns
FADD-TRADD-DKO TNF vs FADD-RIPK1-DKO TNF+C+Z+N	-9,3	ns
FADD-TRADD-DKO TNF vs TRADD-RIPK1-DKO TNF	-8,5	ns
FADD-TRADD-DKO TNF vs TRADD-RIPK1-DKO TNF+Z	-8,8	ns
FADD-TRADD-DKO TNF vs TRADD-RIPK1-DKO TNF+N	-5,2	ns
FADD-TRADD-DKO TNF vs TRADD-RIPK1-DKO TNF+Z+N	-3,6	ns
FADD-TRADD-DKO TNF vs TRADD-RIPK1-DKO TNF+C	1,3	ns
FADD-TRADD-DKO TNF vs TRADD-RIPK1-DKO TNF+C+Z	-8,5	ns
FADD-TRADD-DKO TNF vs TRADD-RIPK1-DKO TNF+C+N	-0,95	ns
FADD-TRADD-DKO TNF vs TRADD-RIPK1-DKO TNF+C+Z+N	-9,2	ns
FADD-TRADD-DKO TNF vs Casp.8-KO TNF	0,13	ns
FADD-TRADD-DKO TNF vs Casp.8-KO TNF+Z	-2,1	ns
FADD-TRADD-DKO TNF vs Casp.8-KO TNF+N	-9,5	ns
FADD-TRADD-DKO TNF vs Casp.8-KO TNF+Z+N	-11	ns
FADD-TRADD-DKO TNF vs Casp.8-KO TNF+C	27	***
FADD-TRADD-DKO TNF vs Casp.8-KO TNF+C+Z	25	***
FADD-TRADD-DKO TNF vs Casp.8-KO TNF+C+N	-9,3	ns
FADD-TRADD-DKO TNF vs Casp.8-KO TNF+C+Z+N	-7,8	ns
FADD-TRADD-DKO TNF vs EV TNF	2,2	ns
FADD-TRADD-DKO TNF vs EV TNF+Z	-6,0	ns
FADD-TRADD-DKO TNF vs EV TNF+N	4,3	ns
FADD-TRADD-DKO TNF vs EV TNF+Z+N	-4,0	ns
FADD-TRADD-DKO TNF vs EV TNF+C	66	***
FADD-TRADD-DKO TNF vs EV TNF+C+Z	-10	ns
FADD-TRADD-DKO TNF vs EV TNF+C+N	55	***
FADD-TRADD-DKO TNF vs EV TNF+C+Z+N	-8,7	ns
FADD-TRADD-DKO TNF+Z vs FADD-TRADD-DKO TNF+N	-5,6	ns
FADD-TRADD-DKO TNF+Z vs FADD-TRADD-DKO TNF+Z+N	-13	ns
FADD-TRADD-DKO TNF+Z vs FADD-TRADD-DKO TNF+C	78	***
FADD-TRADD-DKO TNF+Z vs FADD-TRADD-DKO TNF+C+Z	74	***
FADD-TRADD-DKO TNF+Z vs FADD-TRADD-DKO TNF+C+N	16	ns
FADD-TRADD-DKO TNF+Z vs FADD-TRADD-DKO TNF+C+Z+N	-9,9	ns
FADD-TRADD-DKO TNF+Z vs FADD-RIPK1-DKO TNF	-15	**
FADD-TRADD-DKO TNF+Z vs FADD-RIPK1-DKO TNF+Z	-16	**
FADD-TRADD-DKO TNF+Z vs FADD-RIPK1-DKO TNF+N	-10	ns
FADD-TRADD-DKO TNF+Z vs FADD-RIPK1-DKO TNF+Z+N	-13	ns
FADD-TRADD-DKO TNF+Z vs FADD-RIPK1-DKO TNF+C	-9,6	ns

FADD-TRADD-DKO TNF+Z vs FADD-RIPK1-DKO TNF+C+Z	-7,6	ns
FADD-TRADD-DKO TNF+Z vs FADD-RIPK1-DKO TNF+C+N	-5,7	ns
FADD-TRADD-DKO TNF+Z vs FADD-RIPK1-DKO TNF+C+Z+N	-14	ns
FADD-TRADD-DKO TNF+Z vs TRADD-RIPK1-DKO TNF	-13	ns
FADD-TRADD-DKO TNF+Z vs TRADD-RIPK1-DKO TNF+Z	-13	ns
FADD-TRADD-DKO TNF+Z vs TRADD-RIPK1-DKO TNF+N	-9,6	ns
FADD-TRADD-DKO TNF+Z vs TRADD-RIPK1-DKO TNF+Z+N	-7,9	ns
FADD-TRADD-DKO TNF+Z vs TRADD-RIPK1-DKO TNF+C	-3,1	ns
FADD-TRADD-DKO TNF+Z vs TRADD-RIPK1-DKO TNF+C+Z	-13	ns
FADD-TRADD-DKO TNF+Z vs TRADD-RIPK1-DKO TNF+C+N	-5,3	ns
FADD-TRADD-DKO TNF+Z vs TRADD-RIPK1-DKO TNF+C+Z+N	-14	ns
FADD-TRADD-DKO TNF+Z vs Casp.8-KO TNF	-4,3	ns
FADD-TRADD-DKO TNF+Z vs Casp.8-KO TNF+Z	-6,5	ns
FADD-TRADD-DKO TNF+Z vs Casp.8-KO TNF+N	-14	ns
FADD-TRADD-DKO TNF+Z vs Casp.8-KO TNF+Z+N	-16	ns
FADD-TRADD-DKO TNF+Z vs Casp.8-KO TNF+C	22	***
FADD-TRADD-DKO TNF+Z vs Casp.8-KO TNF+C+Z	21	***
FADD-TRADD-DKO TNF+Z vs Casp.8-KO TNF+C+N	-14	ns
FADD-TRADD-DKO TNF+Z vs Casp.8-KO TNF+C+Z+N	-12	ns
FADD-TRADD-DKO TNF+Z vs EV TNF	-2,2	ns
FADD-TRADD-DKO TNF+Z vs EV TNF+Z	-10	ns
FADD-TRADD-DKO TNF+Z vs EV TNF+N	-0,083	ns
FADD-TRADD-DKO TNF+Z vs EV TNF+Z+N	-8,4	ns
FADD-TRADD-DKO TNF+Z vs EV TNF+C	62	***
FADD-TRADD-DKO TNF+Z vs EV TNF+C+Z	-14	ns
FADD-TRADD-DKO TNF+Z vs EV TNF+C+N	50	***
FADD-TRADD-DKO TNF+Z vs EV TNF+C+Z+N	-13	ns
FADD-TRADD-DKO TNF+N vs FADD-TRADD-DKO TNF+Z+N	-7,8	ns
FADD-TRADD-DKO TNF+N vs FADD-TRADD-DKO TNF+C	84	***
FADD-TRADD-DKO TNF+N vs FADD-TRADD-DKO TNF+C+Z	79	***
FADD-TRADD-DKO TNF+N vs FADD-TRADD-DKO TNF+C+N	21	***
FADD-TRADD-DKO TNF+N vs FADD-TRADD-DKO TNF+C+Z+N	-4,4	ns
FADD-TRADD-DKO TNF+N vs FADD-RIPK1-DKO TNF	-9,9	ns
FADD-TRADD-DKO TNF+N vs FADD-RIPK1-DKO TNF+Z	-10	ns
FADD-TRADD-DKO TNF+N vs FADD-RIPK1-DKO TNF+N	-4,8	ns
FADD-TRADD-DKO TNF+N vs FADD-RIPK1-DKO TNF+Z+N	-7,0	ns
FADD-TRADD-DKO TNF+N vs FADD-RIPK1-DKO TNF+C	-4,0	ns
FADD-TRADD-DKO TNF+N vs FADD-RIPK1-DKO TNF+C+Z	-2,0	ns
FADD-TRADD-DKO TNF+N vs FADD-RIPK1-DKO TNF+C+N	-0,093	ns
FADD-TRADD-DKO TNF+N vs FADD-RIPK1-DKO TNF+C+Z+N	-8,1	ns
FADD-TRADD-DKO TNF+N vs TRADD-RIPK1-DKO TNF	-7,3	ns
FADD-TRADD-DKO TNF+N vs TRADD-RIPK1-DKO TNF+Z	-7,6	ns
FADD-TRADD-DKO TNF+N vs TRADD-RIPK1-DKO TNF+N	-4,0	ns
FADD-TRADD-DKO TNF+N vs TRADD-RIPK1-DKO TNF+Z+N	-2,4	ns
FADD-TRADD-DKO TNF+N vs TRADD-RIPK1-DKO TNF+C	2,5	ns
FADD-TRADD-DKO TNF+N vs TRADD-RIPK1-DKO TNF+C+Z	-7,3	ns
FADD-TRADD-DKO TNF+N vs TRADD-RIPK1-DKO TNF+C+N	0,24	ns
FADD-TRADD-DKO TNF+N vs TRADD-RIPK1-DKO TNF+C+Z+N	-8,0	ns
FADD-TRADD-DKO TNF+N vs Casp.8-KO TNF	1,3	ns
FADD-TRADD-DKO TNF+N vs Casp.8-KO TNF+Z	-0,94	ns
FADD-TRADD-DKO TNF+N vs Casp.8-KO TNF+N	-8,3	ns
FADD-TRADD-DKO TNF+N vs Casp.8-KO TNF+Z+N	-9,9	ns
FADD-TRADD-DKO TNF+N vs Casp.8-KO TNF+C	28	***
FADD-TRADD-DKO TNF+N vs Casp.8-KO TNF+C+Z	26	***
FADD-TRADD-DKO TNF+N vs Casp.8-KO TNF+C+N	-8,1	ns
FADD-TRADD-DKO TNF+N vs Casp.8-KO TNF+C+Z+N	-6,6	ns
FADD-TRADD-DKO TNF+N vs EV TNF	3,4	ns
FADD-TRADD-DKO TNF+N vs EV TNF+Z	-4,8	ns
FADD-TRADD-DKO TNF+N vs EV TNF+N	5,5	ns
FADD-TRADD-DKO TNF+N vs EV TNF+Z+N	-2,8	ns
FADD-TRADD-DKO TNF+N vs EV TNF+C	67	***
FADD-TRADD-DKO TNF+N vs EV TNF+C+Z	-8,9	ns
FADD-TRADD-DKO TNF+N vs EV TNF+C+N	56	***
FADD-TRADD-DKO TNF+N vs EV TNF+C+Z+N	-7,5	ns
FADD-TRADD-DKO TNF+Z+N vs FADD-TRADD-DKO TNF+C	92	***
FADD-TRADD-DKO TNF+Z+N vs FADD-TRADD-DKO TNF+C+Z	87	***

FADD-TRADD-DKO TNF+Z+N vs FADD-TRADD-DKO TNF+C+N	29	***
FADD-TRADD-DKO TNF+Z+N vs FADD-TRADD-DKO TNF+C+Z+N	3,4	ns
FADD-TRADD-DKO TNF+Z+N vs FADD-RIPK1-DKO TNF	-2,1	ns
FADD-TRADD-DKO TNF+Z+N vs FADD-RIPK1-DKO TNF+Z	-2,7	ns
FADD-TRADD-DKO TNF+Z+N vs FADD-RIPK1-DKO TNF+N	3,0	ns
FADD-TRADD-DKO TNF+Z+N vs FADD-RIPK1-DKO TNF+Z+N	0,82	ns
FADD-TRADD-DKO TNF+Z+N vs FADD-RIPK1-DKO TNF+C	3,8	ns
FADD-TRADD-DKO TNF+Z+N vs FADD-RIPK1-DKO TNF+C+Z	5,8	ns
FADD-TRADD-DKO TNF+Z+N vs FADD-RIPK1-DKO TNF+C+N	7,7	ns
FADD-TRADD-DKO TNF+Z+N vs FADD-RIPK1-DKO TNF+C+Z+N	-0,28	ns
FADD-TRADD-DKO TNF+Z+N vs TRADD-RIPK1-DKO TNF	0,46	ns
FADD-TRADD-DKO TNF+Z+N vs TRADD-RIPK1-DKO TNF+Z	0,24	ns
FADD-TRADD-DKO TNF+Z+N vs TRADD-RIPK1-DKO TNF+N	3,8	ns
FADD-TRADD-DKO TNF+Z+N vs TRADD-RIPK1-DKO TNF+Z+N	5,4	ns
FADD-TRADD-DKO TNF+Z+N vs TRADD-RIPK1-DKO TNF+C	10	ns
FADD-TRADD-DKO TNF+Z+N vs TRADD-RIPK1-DKO TNF+C+Z	0,50	ns
FADD-TRADD-DKO TNF+Z+N vs TRADD-RIPK1-DKO TNF+C+N	8,0	ns
FADD-TRADD-DKO TNF+Z+N vs TRADD-RIPK1-DKO TNF+C+Z+N	-0,18	ns
FADD-TRADD-DKO TNF+Z+N vs Casp.8-KO TNF	9,1	ns
FADD-TRADD-DKO TNF+Z+N vs Casp.8-KO TNF+Z	6,9	ns
FADD-TRADD-DKO TNF+Z+N vs Casp.8-KO TNF+N	-0,54	ns
FADD-TRADD-DKO TNF+Z+N vs Casp.8-KO TNF+Z+N	-2,1	ns
FADD-TRADD-DKO TNF+Z+N vs Casp.8-KO TNF+C	36	***
FADD-TRADD-DKO TNF+Z+N vs Casp.8-KO TNF+C+Z	34	***
FADD-TRADD-DKO TNF+Z+N vs Casp.8-KO TNF+C+N	-0,31	ns
FADD-TRADD-DKO TNF+Z+N vs Casp.8-KO TNF+C+Z+N	1,2	ns
FADD-TRADD-DKO TNF+Z+N vs EV TNF	11	ns
FADD-TRADD-DKO TNF+Z+N vs EV TNF+Z	3,0	ns
FADD-TRADD-DKO TNF+Z+N vs EV TNF+N	13	ns
FADD-TRADD-DKO TNF+Z+N vs EV TNF+Z+N	5,0	ns
FADD-TRADD-DKO TNF+Z+N vs EV TNF+C	75	***
FADD-TRADD-DKO TNF+Z+N vs EV TNF+C+Z	-1,1	ns
FADD-TRADD-DKO TNF+Z+N vs EV TNF+C+N	64	***
FADD-TRADD-DKO TNF+Z+N vs EV TNF+C+Z+N	0,34	ns
FADD-TRADD-DKO TNF+C vs FADD-TRADD-DKO TNF+C+Z	-4,8	ns
FADD-TRADD-DKO TNF+C vs FADD-TRADD-DKO TNF+C+N	-62	***
FADD-TRADD-DKO TNF+C vs FADD-TRADD-DKO TNF+C+Z+N	-88	***
FADD-TRADD-DKO TNF+C vs FADD-RIPK1-DKO TNF	-94	***
FADD-TRADD-DKO TNF+C vs FADD-RIPK1-DKO TNF+Z	-94	***
FADD-TRADD-DKO TNF+C vs FADD-RIPK1-DKO TNF+N	-89	***
FADD-TRADD-DKO TNF+C vs FADD-RIPK1-DKO TNF+Z+N	-91	***
FADD-TRADD-DKO TNF+C vs FADD-RIPK1-DKO TNF+C	-88	***
FADD-TRADD-DKO TNF+C vs FADD-RIPK1-DKO TNF+C+Z	-86	***
FADD-TRADD-DKO TNF+C vs FADD-RIPK1-DKO TNF+C+N	-84	***
FADD-TRADD-DKO TNF+C vs FADD-RIPK1-DKO TNF+C+Z+N	-92	***
FADD-TRADD-DKO TNF+C vs TRADD-RIPK1-DKO TNF	-91	***
FADD-TRADD-DKO TNF+C vs TRADD-RIPK1-DKO TNF+Z	-91	***
FADD-TRADD-DKO TNF+C vs TRADD-RIPK1-DKO TNF+N	-88	***
FADD-TRADD-DKO TNF+C vs TRADD-RIPK1-DKO TNF+Z+N	-86	***
FADD-TRADD-DKO TNF+C vs TRADD-RIPK1-DKO TNF+C	-81	***
FADD-TRADD-DKO TNF+C vs TRADD-RIPK1-DKO TNF+C+Z	-91	***
FADD-TRADD-DKO TNF+C vs TRADD-RIPK1-DKO TNF+C+N	-84	***
FADD-TRADD-DKO TNF+C vs TRADD-RIPK1-DKO TNF+C+Z+N	-92	***
FADD-TRADD-DKO TNF+C vs Casp.8-KO TNF	-83	***
FADD-TRADD-DKO TNF+C vs Casp.8-KO TNF+Z	-85	***
FADD-TRADD-DKO TNF+C vs Casp.8-KO TNF+N	-92	***
FADD-TRADD-DKO TNF+C vs Casp.8-KO TNF+Z+N	-94	***
FADD-TRADD-DKO TNF+C vs Casp.8-KO TNF+C	-56	***
FADD-TRADD-DKO TNF+C vs Casp.8-KO TNF+C+Z	-57	***
FADD-TRADD-DKO TNF+C vs Casp.8-KO TNF+C+N	-92	***
FADD-TRADD-DKO TNF+C vs Casp.8-KO TNF+C+Z+N	-90	***
FADD-TRADD-DKO TNF+C vs EV TNF	-80	***
FADD-TRADD-DKO TNF+C vs EV TNF+Z	-89	***
FADD-TRADD-DKO TNF+C vs EV TNF+N	-78	***
FADD-TRADD-DKO TNF+C vs EV TNF+Z+N	-87	***
FADD-TRADD-DKO TNF+C vs EV TNF+C	-17	ns

FADD-TRADD-DKO TNF+C vs EV TNF+C+Z	-93	***
FADD-TRADD-DKO TNF+C vs EV TNF+C+N	-28	**
FADD-TRADD-DKO TNF+C vs EV TNF+C+Z+N	-91	***
FADD-TRADD-DKO TNF+C+Z vs FADD-TRADD-DKO TNF+C+N	-58	***
FADD-TRADD-DKO TNF+C+Z vs FADD-TRADD-DKO TNF+C+Z+N	-83	***
FADD-TRADD-DKO TNF+C+Z vs FADD-RIPK1-DKO TNF	-89	***
FADD-TRADD-DKO TNF+C+Z vs FADD-RIPK1-DKO TNF+Z	-90	***
FADD-TRADD-DKO TNF+C+Z vs FADD-RIPK1-DKO TNF+N	-84	***
FADD-TRADD-DKO TNF+C+Z vs FADD-RIPK1-DKO TNF+Z+N	-86	***
FADD-TRADD-DKO TNF+C+Z vs FADD-RIPK1-DKO TNF+C	-83	***
FADD-TRADD-DKO TNF+C+Z vs FADD-RIPK1-DKO TNF+C+Z	-81	***
FADD-TRADD-DKO TNF+C+Z vs FADD-RIPK1-DKO TNF+C+N	-79	***
FADD-TRADD-DKO TNF+C+Z vs FADD-RIPK1-DKO TNF+C+Z+N	-87	***
FADD-TRADD-DKO TNF+C+Z vs TRADD-RIPK1-DKO TNF	-86	***
FADD-TRADD-DKO TNF+C+Z vs TRADD-RIPK1-DKO TNF+Z	-87	***
FADD-TRADD-DKO TNF+C+Z vs TRADD-RIPK1-DKO TNF+N	-83	***
FADD-TRADD-DKO TNF+C+Z vs TRADD-RIPK1-DKO TNF+Z+N	-81	***
FADD-TRADD-DKO TNF+C+Z vs TRADD-RIPK1-DKO TNF+C	-77	***
FADD-TRADD-DKO TNF+C+Z vs TRADD-RIPK1-DKO TNF+C+Z	-86	***
FADD-TRADD-DKO TNF+C+Z vs TRADD-RIPK1-DKO TNF+C+N	-79	***
FADD-TRADD-DKO TNF+C+Z vs TRADD-RIPK1-DKO TNF+C+Z+N	-87	***
FADD-TRADD-DKO TNF+C+Z vs Casp.8-KO TNF	-78	***
FADD-TRADD-DKO TNF+C+Z vs Casp.8-KO TNF+Z	-80	***
FADD-TRADD-DKO TNF+C+Z vs Casp.8-KO TNF+N	-87	***
FADD-TRADD-DKO TNF+C+Z vs Casp.8-KO TNF+Z+N	-89	***
FADD-TRADD-DKO TNF+C+Z vs Casp.8-KO TNF+C	-51	***
FADD-TRADD-DKO TNF+C+Z vs Casp.8-KO TNF+C+Z	-53	***
FADD-TRADD-DKO TNF+C+Z vs Casp.8-KO TNF+C+N	-87	***
FADD-TRADD-DKO TNF+C+Z vs Casp.8-KO TNF+C+Z+N	-86	***
FADD-TRADD-DKO TNF+C+Z vs EV TNF	-76	***
FADD-TRADD-DKO TNF+C+Z vs EV TNF+Z	-84	***
FADD-TRADD-DKO TNF+C+Z vs EV TNF+N	-74	***
FADD-TRADD-DKO TNF+C+Z vs EV TNF+Z+N	-82	***
FADD-TRADD-DKO TNF+C+Z vs EV TNF+C	-12	ns
FADD-TRADD-DKO TNF+C+Z vs EV TNF+C+Z	-88	***
FADD-TRADD-DKO TNF+C+Z vs EV TNF+C+N	-23	ns
FADD-TRADD-DKO TNF+C+Z vs EV TNF+C+Z+N	-87	***
FADD-TRADD-DKO TNF+C+N vs FADD-TRADD-DKO TNF+C+Z+N	-26	***
FADD-TRADD-DKO TNF+C+N vs FADD-RIPK1-DKO TNF	-31	***
FADD-TRADD-DKO TNF+C+N vs FADD-RIPK1-DKO TNF+Z	-32	***
FADD-TRADD-DKO TNF+C+N vs FADD-RIPK1-DKO TNF+N	-26	***
FADD-TRADD-DKO TNF+C+N vs FADD-RIPK1-DKO TNF+Z+N	-28	***
FADD-TRADD-DKO TNF+C+N vs FADD-RIPK1-DKO TNF+C	-25	***
FADD-TRADD-DKO TNF+C+N vs FADD-RIPK1-DKO TNF+C+Z	-23	***
FADD-TRADD-DKO TNF+C+N vs FADD-RIPK1-DKO TNF+C+N	-22	***
FADD-TRADD-DKO TNF+C+N vs FADD-RIPK1-DKO TNF+C+Z+N	-30	***
FADD-TRADD-DKO TNF+C+N vs TRADD-RIPK1-DKO TNF	-29	***
FADD-TRADD-DKO TNF+C+N vs TRADD-RIPK1-DKO TNF+Z	-29	***
FADD-TRADD-DKO TNF+C+N vs TRADD-RIPK1-DKO TNF+N	-25	***
FADD-TRADD-DKO TNF+C+N vs TRADD-RIPK1-DKO TNF+Z+N	-24	***
FADD-TRADD-DKO TNF+C+N vs TRADD-RIPK1-DKO TNF+C	-19	***
FADD-TRADD-DKO TNF+C+N vs TRADD-RIPK1-DKO TNF+C+Z	-29	***
FADD-TRADD-DKO TNF+C+N vs TRADD-RIPK1-DKO TNF+C+N	-21	***
FADD-TRADD-DKO TNF+C+N vs TRADD-RIPK1-DKO TNF+C+Z+N	-29	***
FADD-TRADD-DKO TNF+C+N vs Casp.8-KO TNF	-20	**
FADD-TRADD-DKO TNF+C+N vs Casp.8-KO TNF+Z	-22	***
FADD-TRADD-DKO TNF+C+N vs Casp.8-KO TNF+N	-30	***
FADD-TRADD-DKO TNF+C+N vs Casp.8-KO TNF+Z+N	-31	***
FADD-TRADD-DKO TNF+C+N vs Casp.8-KO TNF+C	6,3	ns
FADD-TRADD-DKO TNF+C+N vs Casp.8-KO TNF+C+Z	5,0	ns
FADD-TRADD-DKO TNF+C+N vs Casp.8-KO TNF+C+N	-30	***
FADD-TRADD-DKO TNF+C+N vs Casp.8-KO TNF+C+Z+N	-28	***
FADD-TRADD-DKO TNF+C+N vs EV TNF	-18	ns
FADD-TRADD-DKO TNF+C+N vs EV TNF+Z	-26	*
FADD-TRADD-DKO TNF+C+N vs EV TNF+N	-16	ns
FADD-TRADD-DKO TNF+C+N vs EV TNF+Z+N	-24	ns

FADD-TRADD-DKO TNF+C+N vs EV TNF+C	46	***
FADD-TRADD-DKO TNF+C+N vs EV TNF+C+Z	-30	***
FADD-TRADD-DKO TNF+C+N vs EV TNF+C+N	34	***
FADD-TRADD-DKO TNF+C+N vs EV TNF+C+Z+N	-29	***
FADD-TRADD-DKO TNF+C+Z+N vs FADD-RIPK1-DKO TNF	-5,6	ns
FADD-TRADD-DKO TNF+C+Z+N vs FADD-RIPK1-DKO TNF+Z	-6,1	ns
FADD-TRADD-DKO TNF+C+Z+N vs FADD-RIPK1-DKO TNF+N	-0,42	ns
FADD-TRADD-DKO TNF+C+Z+N vs FADD-RIPK1-DKO TNF+Z+N	-2,6	ns
FADD-TRADD-DKO TNF+C+Z+N vs FADD-RIPK1-DKO TNF+C	0,36	ns
FADD-TRADD-DKO TNF+C+Z+N vs FADD-RIPK1-DKO TNF+C+Z	2,4	ns
FADD-TRADD-DKO TNF+C+Z+N vs FADD-RIPK1-DKO TNF+C+N	4,3	ns
FADD-TRADD-DKO TNF+C+Z+N vs FADD-RIPK1-DKO TNF+C+Z+N	-3,7	ns
FADD-TRADD-DKO TNF+C+Z+N vs TRADD-RIPK1-DKO TNF	-3,0	ns
FADD-TRADD-DKO TNF+C+Z+N vs TRADD-RIPK1-DKO TNF+Z	-3,2	ns
FADD-TRADD-DKO TNF+C+Z+N vs TRADD-RIPK1-DKO TNF+N	0,36	ns
FADD-TRADD-DKO TNF+C+Z+N vs TRADD-RIPK1-DKO TNF+Z+N	2,0	ns
FADD-TRADD-DKO TNF+C+Z+N vs TRADD-RIPK1-DKO TNF+C	6,8	ns
FADD-TRADD-DKO TNF+C+Z+N vs TRADD-RIPK1-DKO TNF+C+Z	-2,9	ns
FADD-TRADD-DKO TNF+C+Z+N vs TRADD-RIPK1-DKO TNF+C+N	4,6	ns
FADD-TRADD-DKO TNF+C+Z+N vs TRADD-RIPK1-DKO TNF+C+Z+N	-3,6	ns
FADD-TRADD-DKO TNF+C+Z+N vs Casp.8-KO TNF	5,7	ns
FADD-TRADD-DKO TNF+C+Z+N vs Casp.8-KO TNF+Z	3,4	ns
FADD-TRADD-DKO TNF+C+Z+N vs Casp.8-KO TNF+N	-4,0	ns
FADD-TRADD-DKO TNF+C+Z+N vs Casp.8-KO TNF+Z+N	-5,6	ns
FADD-TRADD-DKO TNF+C+Z+N vs Casp.8-KO TNF+C	32	***
FADD-TRADD-DKO TNF+C+Z+N vs Casp.8-KO TNF+C+Z	31	***
FADD-TRADD-DKO TNF+C+Z+N vs Casp.8-KO TNF+C+N	-3,8	ns
FADD-TRADD-DKO TNF+C+Z+N vs Casp.8-KO TNF+C+Z+N	-2,3	ns
FADD-TRADD-DKO TNF+C+Z+N vs EV TNF	7,8	ns
FADD-TRADD-DKO TNF+C+Z+N vs EV TNF+Z	-0,48	ns
FADD-TRADD-DKO TNF+C+Z+N vs EV TNF+N	9,8	ns
FADD-TRADD-DKO TNF+C+Z+N vs EV TNF+Z+N	1,5	ns
FADD-TRADD-DKO TNF+C+Z+N vs EV TNF+C	71	***
FADD-TRADD-DKO TNF+C+Z+N vs EV TNF+C+Z	-4,5	ns
FADD-TRADD-DKO TNF+C+Z+N vs EV TNF+C+N	60	***
FADD-TRADD-DKO TNF+C+Z+N vs EV TNF+C+Z+N	-3,1	ns
FADD-RIPK1-DKO TNF vs FADD-RIPK1-DKO TNF+Z	-0,56	ns
FADD-RIPK1-DKO TNF vs FADD-RIPK1-DKO TNF+N	5,1	ns
FADD-RIPK1-DKO TNF vs FADD-RIPK1-DKO TNF+Z+N	2,9	ns
FADD-RIPK1-DKO TNF vs FADD-RIPK1-DKO TNF+C	5,9	ns
FADD-RIPK1-DKO TNF vs FADD-RIPK1-DKO TNF+C+Z	7,9	ns
FADD-RIPK1-DKO TNF vs FADD-RIPK1-DKO TNF+C+N	9,8	ns
FADD-RIPK1-DKO TNF vs FADD-RIPK1-DKO TNF+C+Z+N	1,8	ns
FADD-RIPK1-DKO TNF vs TRADD-RIPK1-DKO TNF	2,6	ns
FADD-RIPK1-DKO TNF vs TRADD-RIPK1-DKO TNF+Z	2,3	ns
FADD-RIPK1-DKO TNF vs TRADD-RIPK1-DKO TNF+N	5,9	ns
FADD-RIPK1-DKO TNF vs TRADD-RIPK1-DKO TNF+Z+N	7,6	ns
FADD-RIPK1-DKO TNF vs TRADD-RIPK1-DKO TNF+C	12	*
FADD-RIPK1-DKO TNF vs TRADD-RIPK1-DKO TNF+C+Z	2,6	ns
FADD-RIPK1-DKO TNF vs TRADD-RIPK1-DKO TNF+C+N	10	ns
FADD-RIPK1-DKO TNF vs TRADD-RIPK1-DKO TNF+C+Z+N	1,9	ns
FADD-RIPK1-DKO TNF vs Casp.8-KO TNF	11	ns
FADD-RIPK1-DKO TNF vs Casp.8-KO TNF+Z	9,0	ns
FADD-RIPK1-DKO TNF vs Casp.8-KO TNF+N	1,6	ns
FADD-RIPK1-DKO TNF vs Casp.8-KO TNF+Z+N	-0,013	ns
FADD-RIPK1-DKO TNF vs Casp.8-KO TNF+C	38	***
FADD-RIPK1-DKO TNF vs Casp.8-KO TNF+C+Z	36	***
FADD-RIPK1-DKO TNF vs Casp.8-KO TNF+C+N	1,8	ns
FADD-RIPK1-DKO TNF vs Casp.8-KO TNF+C+Z+N	3,3	ns
FADD-RIPK1-DKO TNF vs EV TNF	13	ns
FADD-RIPK1-DKO TNF vs EV TNF+Z	5,1	ns
FADD-RIPK1-DKO TNF vs EV TNF+N	15	ns
FADD-RIPK1-DKO TNF vs EV TNF+Z+N	7,1	ns
FADD-RIPK1-DKO TNF vs EV TNF+C	77	***
FADD-RIPK1-DKO TNF vs EV TNF+C+Z	1,0	ns
FADD-RIPK1-DKO TNF vs EV TNF+C+N	66	***

FADD-RIPK1-DKO TNF vs EV TNF+C+Z+N	2,5	ns
FADD-RIPK1-DKO TNF+Z vs FADD-RIPK1-DKO TNF+N	5,7	ns
FADD-RIPK1-DKO TNF+Z vs FADD-RIPK1-DKO TNF+Z+N	3,5	ns
FADD-RIPK1-DKO TNF+Z vs FADD-RIPK1-DKO TNF+C	6,5	ns
FADD-RIPK1-DKO TNF+Z vs FADD-RIPK1-DKO TNF+C+Z	8,5	ns
FADD-RIPK1-DKO TNF+Z vs FADD-RIPK1-DKO TNF+C+N	10	ns
FADD-RIPK1-DKO TNF+Z vs FADD-RIPK1-DKO TNF+C+Z+N	2,4	ns
FADD-RIPK1-DKO TNF+Z vs TRADD-RIPK1-DKO TNF	3,1	ns
FADD-RIPK1-DKO TNF+Z vs TRADD-RIPK1-DKO TNF+Z	2,9	ns
FADD-RIPK1-DKO TNF+Z vs TRADD-RIPK1-DKO TNF+N	6,5	ns
FADD-RIPK1-DKO TNF+Z vs TRADD-RIPK1-DKO TNF+Z+N	8,1	ns
FADD-RIPK1-DKO TNF+Z vs TRADD-RIPK1-DKO TNF+C	13	*
FADD-RIPK1-DKO TNF+Z vs TRADD-RIPK1-DKO TNF+C+Z	3,2	ns
FADD-RIPK1-DKO TNF+Z vs TRADD-RIPK1-DKO TNF+C+N	11	ns
FADD-RIPK1-DKO TNF+Z vs TRADD-RIPK1-DKO TNF+C+Z+N	2,5	ns
FADD-RIPK1-DKO TNF+Z vs Casp.8-KO TNF	12	ns
FADD-RIPK1-DKO TNF+Z vs Casp.8-KO TNF+Z	9,5	ns
FADD-RIPK1-DKO TNF+Z vs Casp.8-KO TNF+N	2,1	ns
FADD-RIPK1-DKO TNF+Z vs Casp.8-KO TNF+Z+N	0,54	ns
FADD-RIPK1-DKO TNF+Z vs Casp.8-KO TNF+C	38	***
FADD-RIPK1-DKO TNF+Z vs Casp.8-KO TNF+C+Z	37	***
FADD-RIPK1-DKO TNF+Z vs Casp.8-KO TNF+C+N	2,4	ns
FADD-RIPK1-DKO TNF+Z vs Casp.8-KO TNF+C+Z+N	3,8	ns
FADD-RIPK1-DKO TNF+Z vs EV TNF	14	ns
FADD-RIPK1-DKO TNF+Z vs EV TNF+Z	5,6	ns
FADD-RIPK1-DKO TNF+Z vs EV TNF+N	16	ns
FADD-RIPK1-DKO TNF+Z vs EV TNF+Z+N	7,6	ns
FADD-RIPK1-DKO TNF+Z vs EV TNF+C	78	***
FADD-RIPK1-DKO TNF+Z vs EV TNF+C+Z	1,6	ns
FADD-RIPK1-DKO TNF+Z vs EV TNF+C+N	66	***
FADD-RIPK1-DKO TNF+Z vs EV TNF+C+Z+N	3,0	ns
FADD-RIPK1-DKO TNF+N vs FADD-RIPK1-DKO TNF+Z+N	-2,2	ns
FADD-RIPK1-DKO TNF+N vs FADD-RIPK1-DKO TNF+C	0,78	ns
FADD-RIPK1-DKO TNF+N vs FADD-RIPK1-DKO TNF+C+Z	2,8	ns
FADD-RIPK1-DKO TNF+N vs FADD-RIPK1-DKO TNF+C+N	4,7	ns
FADD-RIPK1-DKO TNF+N vs FADD-RIPK1-DKO TNF+C+Z+N	-3,3	ns
FADD-RIPK1-DKO TNF+N vs TRADD-RIPK1-DKO TNF	-2,6	ns
FADD-RIPK1-DKO TNF+N vs TRADD-RIPK1-DKO TNF+Z	-2,8	ns
FADD-RIPK1-DKO TNF+N vs TRADD-RIPK1-DKO TNF+N	0,78	ns
FADD-RIPK1-DKO TNF+N vs TRADD-RIPK1-DKO TNF+Z+N	2,4	ns
FADD-RIPK1-DKO TNF+N vs TRADD-RIPK1-DKO TNF+C	7,3	ns
FADD-RIPK1-DKO TNF+N vs TRADD-RIPK1-DKO TNF+C+Z	-2,5	ns
FADD-RIPK1-DKO TNF+N vs TRADD-RIPK1-DKO TNF+C+N	5,0	ns
FADD-RIPK1-DKO TNF+N vs TRADD-RIPK1-DKO TNF+C+Z+N	-3,2	ns
FADD-RIPK1-DKO TNF+N vs Casp.8-KO TNF	6,1	ns
FADD-RIPK1-DKO TNF+N vs Casp.8-KO TNF+Z	3,8	ns
FADD-RIPK1-DKO TNF+N vs Casp.8-KO TNF+N	-3,6	ns
FADD-RIPK1-DKO TNF+N vs Casp.8-KO TNF+Z+N	-5,2	ns
FADD-RIPK1-DKO TNF+N vs Casp.8-KO TNF+C	32	***
FADD-RIPK1-DKO TNF+N vs Casp.8-KO TNF+C+Z	31	***
FADD-RIPK1-DKO TNF+N vs Casp.8-KO TNF+C+N	-3,3	ns
FADD-RIPK1-DKO TNF+N vs Casp.8-KO TNF+C+Z+N	-1,9	ns
FADD-RIPK1-DKO TNF+N vs EV TNF	8,2	ns
FADD-RIPK1-DKO TNF+N vs EV TNF+Z	-0,067	ns
FADD-RIPK1-DKO TNF+N vs EV TNF+N	10	ns
FADD-RIPK1-DKO TNF+N vs EV TNF+Z+N	1,9	ns
FADD-RIPK1-DKO TNF+N vs EV TNF+C	72	***
FADD-RIPK1-DKO TNF+N vs EV TNF+C+Z	-4,1	ns
FADD-RIPK1-DKO TNF+N vs EV TNF+C+N	61	***
FADD-RIPK1-DKO TNF+N vs EV TNF+C+Z+N	-2,7	ns
FADD-RIPK1-DKO TNF+Z+N vs FADD-RIPK1-DKO TNF+C	3,0	ns
FADD-RIPK1-DKO TNF+Z+N vs FADD-RIPK1-DKO TNF+C+Z	5,0	ns
FADD-RIPK1-DKO TNF+Z+N vs FADD-RIPK1-DKO TNF+C+N	6,9	ns
FADD-RIPK1-DKO TNF+Z+N vs FADD-RIPK1-DKO TNF+C+Z+N	-1,1	ns
FADD-RIPK1-DKO TNF+Z+N vs TRADD-RIPK1-DKO TNF	-0,36	ns
FADD-RIPK1-DKO TNF+Z+N vs TRADD-RIPK1-DKO TNF+Z	-0,59	ns

FADD-RIPK1-DKO TNF+Z+N vs TRADD-RIPK1-DKO TNF+N	3,0	ns
FADD-RIPK1-DKO TNF+Z+N vs TRADD-RIPK1-DKO TNF+Z+N	4,6	ns
FADD-RIPK1-DKO TNF+Z+N vs TRADD-RIPK1-DKO TNF+C	9,5	ns
FADD-RIPK1-DKO TNF+Z+N vs TRADD-RIPK1-DKO TNF+C+Z	-0,32	ns
FADD-RIPK1-DKO TNF+Z+N vs TRADD-RIPK1-DKO TNF+C+N	7,2	ns
FADD-RIPK1-DKO TNF+Z+N vs TRADD-RIPK1-DKO TNF+C+Z+N	-1,0	ns
FADD-RIPK1-DKO TNF+Z+N vs Casp.8-KO TNF	8,3	ns
FADD-RIPK1-DKO TNF+Z+N vs Casp.8-KO TNF+Z	6,0	ns
FADD-RIPK1-DKO TNF+Z+N vs Casp.8-KO TNF+N	-1,4	ns
FADD-RIPK1-DKO TNF+Z+N vs Casp.8-KO TNF+Z+N	-2,9	ns
FADD-RIPK1-DKO TNF+Z+N vs Casp.8-KO TNF+C	35	***
FADD-RIPK1-DKO TNF+Z+N vs Casp.8-KO TNF+C+Z	33	***
FADD-RIPK1-DKO TNF+Z+N vs Casp.8-KO TNF+C+N	-1,1	ns
FADD-RIPK1-DKO TNF+Z+N vs Casp.8-KO TNF+C+Z+N	0,33	ns
FADD-RIPK1-DKO TNF+Z+N vs EV TNF	10	ns
FADD-RIPK1-DKO TNF+Z+N vs EV TNF+Z	2,1	ns
FADD-RIPK1-DKO TNF+Z+N vs EV TNF+N	12	ns
FADD-RIPK1-DKO TNF+Z+N vs EV TNF+Z+N	4,1	ns
FADD-RIPK1-DKO TNF+Z+N vs EV TNF+C	74	***
FADD-RIPK1-DKO TNF+Z+N vs EV TNF+C+Z	-1,9	ns
FADD-RIPK1-DKO TNF+Z+N vs EV TNF+C+N	63	***
FADD-RIPK1-DKO TNF+Z+N vs EV TNF+C+Z+N	-0,49	ns
FADD-RIPK1-DKO TNF+C vs FADD-RIPK1-DKO TNF+C+Z	2,0	ns
FADD-RIPK1-DKO TNF+C vs FADD-RIPK1-DKO TNF+C+N	3,9	ns
FADD-RIPK1-DKO TNF+C vs FADD-RIPK1-DKO TNF+C+Z+N	-4,1	ns
FADD-RIPK1-DKO TNF+C vs TRADD-RIPK1-DKO TNF	-3,3	ns
FADD-RIPK1-DKO TNF+C vs TRADD-RIPK1-DKO TNF+Z	-3,6	ns
FADD-RIPK1-DKO TNF+C vs TRADD-RIPK1-DKO TNF+N	0,00	ns
FADD-RIPK1-DKO TNF+C vs TRADD-RIPK1-DKO TNF+Z+N	1,6	ns
FADD-RIPK1-DKO TNF+C vs TRADD-RIPK1-DKO TNF+C	6,5	ns
FADD-RIPK1-DKO TNF+C vs TRADD-RIPK1-DKO TNF+C+Z	-3,3	ns
FADD-RIPK1-DKO TNF+C vs TRADD-RIPK1-DKO TNF+C+N	4,2	ns
FADD-RIPK1-DKO TNF+C vs TRADD-RIPK1-DKO TNF+C+Z+N	-4,0	ns
FADD-RIPK1-DKO TNF+C vs Casp.8-KO TNF	5,3	ns
FADD-RIPK1-DKO TNF+C vs Casp.8-KO TNF+Z	3,1	ns
FADD-RIPK1-DKO TNF+C vs Casp.8-KO TNF+N	-4,3	ns
FADD-RIPK1-DKO TNF+C vs Casp.8-KO TNF+Z+N	-5,9	ns
FADD-RIPK1-DKO TNF+C vs Casp.8-KO TNF+C	32	***
FADD-RIPK1-DKO TNF+C vs Casp.8-KO TNF+C+Z	30	***
FADD-RIPK1-DKO TNF+C vs Casp.8-KO TNF+C+N	-4,1	ns
FADD-RIPK1-DKO TNF+C vs Casp.8-KO TNF+C+Z+N	-2,6	ns
FADD-RIPK1-DKO TNF+C vs EV TNF	7,4	ns
FADD-RIPK1-DKO TNF+C vs EV TNF+Z	-0,85	ns
FADD-RIPK1-DKO TNF+C vs EV TNF+N	9,5	ns
FADD-RIPK1-DKO TNF+C vs EV TNF+Z+N	1,2	ns
FADD-RIPK1-DKO TNF+C vs EV TNF+C	71	***
FADD-RIPK1-DKO TNF+C vs EV TNF+C+Z	-4,9	ns
FADD-RIPK1-DKO TNF+C vs EV TNF+C+N	60	***
FADD-RIPK1-DKO TNF+C vs EV TNF+C+Z+N	-3,5	ns
FADD-RIPK1-DKO TNF+C+Z vs FADD-RIPK1-DKO TNF+C+N	1,9	ns
FADD-RIPK1-DKO TNF+C+Z vs FADD-RIPK1-DKO TNF+C+Z+N	-6,1	ns
FADD-RIPK1-DKO TNF+C+Z vs TRADD-RIPK1-DKO TNF	-5,4	ns
FADD-RIPK1-DKO TNF+C+Z vs TRADD-RIPK1-DKO TNF+Z	-5,6	ns
FADD-RIPK1-DKO TNF+C+Z vs TRADD-RIPK1-DKO TNF+N	-2,0	ns
FADD-RIPK1-DKO TNF+C+Z vs TRADD-RIPK1-DKO TNF+Z+N	-0,38	ns
FADD-RIPK1-DKO TNF+C+Z vs TRADD-RIPK1-DKO TNF+C	4,5	ns
FADD-RIPK1-DKO TNF+C+Z vs TRADD-RIPK1-DKO TNF+C+Z	-5,3	ns
FADD-RIPK1-DKO TNF+C+Z vs TRADD-RIPK1-DKO TNF+C+N	2,2	ns
FADD-RIPK1-DKO TNF+C+Z vs TRADD-RIPK1-DKO TNF+C+Z+N	-6,0	ns
FADD-RIPK1-DKO TNF+C+Z vs Casp.8-KO TNF	3,3	ns
FADD-RIPK1-DKO TNF+C+Z vs Casp.8-KO TNF+Z	1,0	ns
FADD-RIPK1-DKO TNF+C+Z vs Casp.8-KO TNF+N	-6,4	ns
FADD-RIPK1-DKO TNF+C+Z vs Casp.8-KO TNF+Z+N	-7,9	ns
FADD-RIPK1-DKO TNF+C+Z vs Casp.8-KO TNF+C	30	***
FADD-RIPK1-DKO TNF+C+Z vs Casp.8-KO TNF+C+Z	28	***
FADD-RIPK1-DKO TNF+C+Z vs Casp.8-KO TNF+C+N	-6,1	ns

FADD-RIPK1-DKO TNF+C+Z vs Casp.8-KO TNF+C+Z+N	-4,7	ns
FADD-RIPK1-DKO TNF+C+Z vs EV TNF	5,4	ns
FADD-RIPK1-DKO TNF+C+Z vs EV TNF+Z	-2,9	ns
FADD-RIPK1-DKO TNF+C+Z vs EV TNF+N	7,5	ns
FADD-RIPK1-DKO TNF+C+Z vs EV TNF+Z+N	-0,87	ns
FADD-RIPK1-DKO TNF+C+Z vs EV TNF+C	69	***
FADD-RIPK1-DKO TNF+C+Z vs EV TNF+C+Z	-6,9	ns
FADD-RIPK1-DKO TNF+C+Z vs EV TNF+C+N	58	***
FADD-RIPK1-DKO TNF+C+Z vs EV TNF+C+Z+N	-5,5	ns
FADD-RIPK1-DKO TNF+C+N vs FADD-RIPK1-DKO TNF+C+Z+N	-8,0	ns
FADD-RIPK1-DKO TNF+C+N vs TRADD-RIPK1-DKO TNF	-7,2	ns
FADD-RIPK1-DKO TNF+C+N vs TRADD-RIPK1-DKO TNF+Z	-7,5	ns
FADD-RIPK1-DKO TNF+C+N vs TRADD-RIPK1-DKO TNF+N	-3,9	ns
FADD-RIPK1-DKO TNF+C+N vs TRADD-RIPK1-DKO TNF+Z+N	-2,3	ns
FADD-RIPK1-DKO TNF+C+N vs TRADD-RIPK1-DKO TNF+C	2,6	ns
FADD-RIPK1-DKO TNF+C+N vs TRADD-RIPK1-DKO TNF+C+Z	-7,2	ns
FADD-RIPK1-DKO TNF+C+N vs TRADD-RIPK1-DKO TNF+C+N	0,34	ns
FADD-RIPK1-DKO TNF+C+N vs TRADD-RIPK1-DKO TNF+C+Z+N	-7,9	ns
FADD-RIPK1-DKO TNF+C+N vs Casp.8-KO TNF	1,4	ns
FADD-RIPK1-DKO TNF+C+N vs Casp.8-KO TNF+Z	-0,85	ns
FADD-RIPK1-DKO TNF+C+N vs Casp.8-KO TNF+N	-8,3	ns
FADD-RIPK1-DKO TNF+C+N vs Casp.8-KO TNF+Z+N	-9,8	ns
FADD-RIPK1-DKO TNF+C+N vs Casp.8-KO TNF+C	28	***
FADD-RIPK1-DKO TNF+C+N vs Casp.8-KO TNF+C+Z	27	***
FADD-RIPK1-DKO TNF+C+N vs Casp.8-KO TNF+C+N	-8,0	ns
FADD-RIPK1-DKO TNF+C+N vs Casp.8-KO TNF+C+Z+N	-6,6	ns
FADD-RIPK1-DKO TNF+C+N vs EV TNF	3,5	ns
FADD-RIPK1-DKO TNF+C+N vs EV TNF+Z	-4,8	ns
FADD-RIPK1-DKO TNF+C+N vs EV TNF+N	5,6	ns
FADD-RIPK1-DKO TNF+C+N vs EV TNF+Z+N	-2,8	ns
FADD-RIPK1-DKO TNF+C+N vs EV TNF+C	67	***
FADD-RIPK1-DKO TNF+C+N vs EV TNF+C+Z	-8,8	ns
FADD-RIPK1-DKO TNF+C+N vs EV TNF+C+N	56	***
FADD-RIPK1-DKO TNF+C+N vs EV TNF+C+Z+N	-7,4	ns
FADD-RIPK1-DKO TNF+C+Z+N vs TRADD-RIPK1-DKO TNF	0,75	ns
FADD-RIPK1-DKO TNF+C+Z+N vs TRADD-RIPK1-DKO TNF+Z	0,52	ns
FADD-RIPK1-DKO TNF+C+Z+N vs TRADD-RIPK1-DKO TNF+N	4,1	ns
FADD-RIPK1-DKO TNF+C+Z+N vs TRADD-RIPK1-DKO TNF+Z+N	5,7	ns
FADD-RIPK1-DKO TNF+C+Z+N vs TRADD-RIPK1-DKO TNF+C	11	ns
FADD-RIPK1-DKO TNF+C+Z+N vs TRADD-RIPK1-DKO TNF+C+Z	0,78	ns
FADD-RIPK1-DKO TNF+C+Z+N vs TRADD-RIPK1-DKO TNF+C+N	8,3	ns
FADD-RIPK1-DKO TNF+C+Z+N vs TRADD-RIPK1-DKO TNF+C+Z+N	0,11	ns
FADD-RIPK1-DKO TNF+C+Z+N vs Casp.8-KO TNF	9,4	ns
FADD-RIPK1-DKO TNF+C+Z+N vs Casp.8-KO TNF+Z	7,1	ns
FADD-RIPK1-DKO TNF+C+Z+N vs Casp.8-KO TNF+N	-0,26	ns
FADD-RIPK1-DKO TNF+C+Z+N vs Casp.8-KO TNF+Z+N	-1,8	ns
FADD-RIPK1-DKO TNF+C+Z+N vs Casp.8-KO TNF+C	36	***
FADD-RIPK1-DKO TNF+C+Z+N vs Casp.8-KO TNF+C+Z	34	***
FADD-RIPK1-DKO TNF+C+Z+N vs Casp.8-KO TNF+C+N	-0,024	ns
FADD-RIPK1-DKO TNF+C+Z+N vs Casp.8-KO TNF+C+Z+N	1,4	ns
FADD-RIPK1-DKO TNF+C+Z+N vs EV TNF	11	ns
FADD-RIPK1-DKO TNF+C+Z+N vs EV TNF+Z	3,2	ns
FADD-RIPK1-DKO TNF+C+Z+N vs EV TNF+N	14	ns
FADD-RIPK1-DKO TNF+C+Z+N vs EV TNF+Z+N	5,2	ns
FADD-RIPK1-DKO TNF+C+Z+N vs EV TNF+C	75	***
FADD-RIPK1-DKO TNF+C+Z+N vs EV TNF+C+Z	-0,79	ns
FADD-RIPK1-DKO TNF+C+Z+N vs EV TNF+C+N	64	***
FADD-RIPK1-DKO TNF+C+Z+N vs EV TNF+C+Z+N	0,62	ns
TRADD-RIPK1-DKO TNF vs TRADD-RIPK1-DKO TNF+Z	-0,23	ns
TRADD-RIPK1-DKO TNF vs TRADD-RIPK1-DKO TNF+N	3,3	ns
TRADD-RIPK1-DKO TNF vs TRADD-RIPK1-DKO TNF+Z+N	5,0	ns
TRADD-RIPK1-DKO TNF vs TRADD-RIPK1-DKO TNF+C	9,8	ns
TRADD-RIPK1-DKO TNF vs TRADD-RIPK1-DKO TNF+C+Z	0,036	ns
TRADD-RIPK1-DKO TNF vs TRADD-RIPK1-DKO TNF+C+N	7,6	ns
TRADD-RIPK1-DKO TNF vs TRADD-RIPK1-DKO TNF+C+Z+N	-0,64	ns
TRADD-RIPK1-DKO TNF vs Casp.8-KO TNF	8,7	ns

TRADD-RIPK1-DKO TNF vs Casp.8-KO TNF+Z	6,4	ns
TRADD-RIPK1-DKO TNF vs Casp.8-KO TNF+N	-1,0	ns
TRADD-RIPK1-DKO TNF vs Casp.8-KO TNF+Z+N	-2,6	ns
TRADD-RIPK1-DKO TNF vs Casp.8-KO TNF+C	35	***
TRADD-RIPK1-DKO TNF vs Casp.8-KO TNF+C+Z	34	***
TRADD-RIPK1-DKO TNF vs Casp.8-KO TNF+C+N	-0,77	ns
TRADD-RIPK1-DKO TNF vs Casp.8-KO TNF+C+Z+N	0,69	ns
TRADD-RIPK1-DKO TNF vs EV TNF	11	ns
TRADD-RIPK1-DKO TNF vs EV TNF+Z	2,5	ns
TRADD-RIPK1-DKO TNF vs EV TNF+N	13	ns
TRADD-RIPK1-DKO TNF vs EV TNF+Z+N	4,5	ns
TRADD-RIPK1-DKO TNF vs EV TNF+C	74	***
TRADD-RIPK1-DKO TNF vs EV TNF+C+Z	-1,5	ns
TRADD-RIPK1-DKO TNF vs EV TNF+C+N	63	***
TRADD-RIPK1-DKO TNF vs EV TNF+C+Z+N	-0,12	ns
TRADD-RIPK1-DKO TNF+Z vs TRADD-RIPK1-DKO TNF+N	3,6	ns
TRADD-RIPK1-DKO TNF+Z vs TRADD-RIPK1-DKO TNF+Z+N	5,2	ns
TRADD-RIPK1-DKO TNF+Z vs TRADD-RIPK1-DKO TNF+C	10	ns
TRADD-RIPK1-DKO TNF+Z vs TRADD-RIPK1-DKO TNF+C+Z	0,26	ns
TRADD-RIPK1-DKO TNF+Z vs TRADD-RIPK1-DKO TNF+C+N	7,8	ns
TRADD-RIPK1-DKO TNF+Z vs TRADD-RIPK1-DKO TNF+C+Z+N	-0,41	ns
TRADD-RIPK1-DKO TNF+Z vs Casp.8-KO TNF	8,9	ns
TRADD-RIPK1-DKO TNF+Z vs Casp.8-KO TNF+Z	6,6	ns
TRADD-RIPK1-DKO TNF+Z vs Casp.8-KO TNF+N	-0,78	ns
TRADD-RIPK1-DKO TNF+Z vs Casp.8-KO TNF+Z+N	-2,4	ns
TRADD-RIPK1-DKO TNF+Z vs Casp.8-KO TNF+C	35	***
TRADD-RIPK1-DKO TNF+Z vs Casp.8-KO TNF+C+Z	34	***
TRADD-RIPK1-DKO TNF+Z vs Casp.8-KO TNF+C+N	-0,54	ns
TRADD-RIPK1-DKO TNF+Z vs Casp.8-KO TNF+C+Z+N	0,92	ns
TRADD-RIPK1-DKO TNF+Z vs EV TNF	11	ns
TRADD-RIPK1-DKO TNF+Z vs EV TNF+Z	2,7	ns
TRADD-RIPK1-DKO TNF+Z vs EV TNF+N	13	ns
TRADD-RIPK1-DKO TNF+Z vs EV TNF+Z+N	4,7	ns
TRADD-RIPK1-DKO TNF+Z vs EV TNF+C	75	***
TRADD-RIPK1-DKO TNF+Z vs EV TNF+C+Z	-1,3	ns
TRADD-RIPK1-DKO TNF+Z vs EV TNF+C+N	63	***
TRADD-RIPK1-DKO TNF+Z vs EV TNF+C+Z+N	0,10	ns
TRADD-RIPK1-DKO TNF+N vs TRADD-RIPK1-DKO TNF+Z+N	1,6	ns
TRADD-RIPK1-DKO TNF+N vs TRADD-RIPK1-DKO TNF+C	6,5	ns
TRADD-RIPK1-DKO TNF+N vs TRADD-RIPK1-DKO TNF+C+Z	-3,3	ns
TRADD-RIPK1-DKO TNF+N vs TRADD-RIPK1-DKO TNF+C+N	4,2	ns
TRADD-RIPK1-DKO TNF+N vs TRADD-RIPK1-DKO TNF+C+Z+N	-4,0	ns
TRADD-RIPK1-DKO TNF+N vs Casp.8-KO TNF	5,3	ns
TRADD-RIPK1-DKO TNF+N vs Casp.8-KO TNF+Z	3,1	ns
TRADD-RIPK1-DKO TNF+N vs Casp.8-KO TNF+N	-4,3	ns
TRADD-RIPK1-DKO TNF+N vs Casp.8-KO TNF+Z+N	-5,9	ns
TRADD-RIPK1-DKO TNF+N vs Casp.8-KO TNF+C	32	***
TRADD-RIPK1-DKO TNF+N vs Casp.8-KO TNF+C+Z	30	***
TRADD-RIPK1-DKO TNF+N vs Casp.8-KO TNF+C+N	-4,1	ns
TRADD-RIPK1-DKO TNF+N vs Casp.8-KO TNF+C+Z+N	-2,6	ns
TRADD-RIPK1-DKO TNF+N vs EV TNF	7,4	ns
TRADD-RIPK1-DKO TNF+N vs EV TNF+Z	-0,85	ns
TRADD-RIPK1-DKO TNF+N vs EV TNF+N	9,5	ns
TRADD-RIPK1-DKO TNF+N vs EV TNF+Z+N	1,2	ns
TRADD-RIPK1-DKO TNF+N vs EV TNF+C	71	***
TRADD-RIPK1-DKO TNF+N vs EV TNF+C+Z	-4,9	ns
TRADD-RIPK1-DKO TNF+N vs EV TNF+C+N	60	***
TRADD-RIPK1-DKO TNF+N vs EV TNF+C+Z+N	-3,5	ns
TRADD-RIPK1-DKO TNF+Z+N vs TRADD-RIPK1-DKO TNF+C	4,8	ns
TRADD-RIPK1-DKO TNF+Z+N vs TRADD-RIPK1-DKO TNF+C+Z	-4,9	ns
TRADD-RIPK1-DKO TNF+Z+N vs TRADD-RIPK1-DKO TNF+C+N	2,6	ns
TRADD-RIPK1-DKO TNF+Z+N vs TRADD-RIPK1-DKO TNF+C+Z+N	-5,6	ns
TRADD-RIPK1-DKO TNF+Z+N vs Casp.8-KO TNF	3,7	ns
TRADD-RIPK1-DKO TNF+Z+N vs Casp.8-KO TNF+Z	1,4	ns
TRADD-RIPK1-DKO TNF+Z+N vs Casp.8-KO TNF+N	-6,0	ns
TRADD-RIPK1-DKO TNF+Z+N vs Casp.8-KO TNF+Z+N	-7,6	ns

TRADD-RIPK1-DKO TNF+Z+N vs Casp.8-KO TNF+C	30	***
TRADD-RIPK1-DKO TNF+Z+N vs Casp.8-KO TNF+C+Z	29	***
TRADD-RIPK1-DKO TNF+Z+N vs Casp.8-KO TNF+C+N	-5,8	ns
TRADD-RIPK1-DKO TNF+Z+N vs Casp.8-KO TNF+C+Z+N	-4,3	ns
TRADD-RIPK1-DKO TNF+Z+N vs EV TNF	5,8	ns
TRADD-RIPK1-DKO TNF+Z+N vs EV TNF+Z	-2,5	ns
TRADD-RIPK1-DKO TNF+Z+N vs EV TNF+N	7,8	ns
TRADD-RIPK1-DKO TNF+Z+N vs EV TNF+Z+N	-0,48	ns
TRADD-RIPK1-DKO TNF+Z+N vs EV TNF+C	69	***
TRADD-RIPK1-DKO TNF+Z+N vs EV TNF+C+Z	-6,5	ns
TRADD-RIPK1-DKO TNF+Z+N vs EV TNF+C+N	58	***
TRADD-RIPK1-DKO TNF+Z+N vs EV TNF+C+Z+N	-5,1	ns
TRADD-RIPK1-DKO TNF+C vs TRADD-RIPK1-DKO TNF+C+Z	-9,8	ns
TRADD-RIPK1-DKO TNF+C vs TRADD-RIPK1-DKO TNF+C+N	-2,2	ns
TRADD-RIPK1-DKO TNF+C vs TRADD-RIPK1-DKO TNF+C+Z+N	-10	ns
TRADD-RIPK1-DKO TNF+C vs Casp.8-KO TNF	-1,2	ns
TRADD-RIPK1-DKO TNF+C vs Casp.8-KO TNF+Z	-3,4	ns
TRADD-RIPK1-DKO TNF+C vs Casp.8-KO TNF+N	-11	ns
TRADD-RIPK1-DKO TNF+C vs Casp.8-KO TNF+Z+N	-12	ns
TRADD-RIPK1-DKO TNF+C vs Casp.8-KO TNF+C	25	***
TRADD-RIPK1-DKO TNF+C vs Casp.8-KO TNF+C+Z	24	***
TRADD-RIPK1-DKO TNF+C vs Casp.8-KO TNF+C+N	-11	ns
TRADD-RIPK1-DKO TNF+C vs Casp.8-KO TNF+C+Z+N	-9,1	ns
TRADD-RIPK1-DKO TNF+C vs EV TNF	0,92	ns
TRADD-RIPK1-DKO TNF+C vs EV TNF+Z	-7,3	ns
TRADD-RIPK1-DKO TNF+C vs EV TNF+N	3,0	ns
TRADD-RIPK1-DKO TNF+C vs EV TNF+Z+N	-5,3	ns
TRADD-RIPK1-DKO TNF+C vs EV TNF+C	65	***
TRADD-RIPK1-DKO TNF+C vs EV TNF+C+Z	-11	ns
TRADD-RIPK1-DKO TNF+C vs EV TNF+C+N	53	***
TRADD-RIPK1-DKO TNF+C vs EV TNF+C+Z+N	-9,9	ns
TRADD-RIPK1-DKO TNF+C+Z vs TRADD-RIPK1-DKO TNF+C+N	7,5	ns
TRADD-RIPK1-DKO TNF+C+Z vs TRADD-RIPK1-DKO TNF+C+Z+N	-0,68	ns
TRADD-RIPK1-DKO TNF+C+Z vs Casp.8-KO TNF	8,6	ns
TRADD-RIPK1-DKO TNF+C+Z vs Casp.8-KO TNF+Z	6,4	ns
TRADD-RIPK1-DKO TNF+C+Z vs Casp.8-KO TNF+N	-1,0	ns
TRADD-RIPK1-DKO TNF+C+Z vs Casp.8-KO TNF+Z+N	-2,6	ns
TRADD-RIPK1-DKO TNF+C+Z vs Casp.8-KO TNF+C	35	***
TRADD-RIPK1-DKO TNF+C+Z vs Casp.8-KO TNF+C+Z	34	***
TRADD-RIPK1-DKO TNF+C+Z vs Casp.8-KO TNF+C+N	-0,81	ns
TRADD-RIPK1-DKO TNF+C+Z vs Casp.8-KO TNF+C+Z+N	0,66	ns
TRADD-RIPK1-DKO TNF+C+Z vs EV TNF	11	ns
TRADD-RIPK1-DKO TNF+C+Z vs EV TNF+Z	2,5	ns
TRADD-RIPK1-DKO TNF+C+Z vs EV TNF+N	13	ns
TRADD-RIPK1-DKO TNF+C+Z vs EV TNF+Z+N	4,5	ns
TRADD-RIPK1-DKO TNF+C+Z vs EV TNF+C	74	***
TRADD-RIPK1-DKO TNF+C+Z vs EV TNF+C+Z	-1,6	ns
TRADD-RIPK1-DKO TNF+C+Z vs EV TNF+C+N	63	***
TRADD-RIPK1-DKO TNF+C+Z vs EV TNF+C+Z+N	-0,16	ns
TRADD-RIPK1-DKO TNF+C+N vs TRADD-RIPK1-DKO TNF+C+Z+N	-8,2	ns
TRADD-RIPK1-DKO TNF+C+N vs Casp.8-KO TNF	1,1	ns
TRADD-RIPK1-DKO TNF+C+N vs Casp.8-KO TNF+Z	-1,2	ns
TRADD-RIPK1-DKO TNF+C+N vs Casp.8-KO TNF+N	-8,6	ns
TRADD-RIPK1-DKO TNF+C+N vs Casp.8-KO TNF+Z+N	-10	ns
TRADD-RIPK1-DKO TNF+C+N vs Casp.8-KO TNF+C	27	***
TRADD-RIPK1-DKO TNF+C+N vs Casp.8-KO TNF+C+Z	26	***
TRADD-RIPK1-DKO TNF+C+N vs Casp.8-KO TNF+C+N	-8,4	ns
TRADD-RIPK1-DKO TNF+C+N vs Casp.8-KO TNF+C+Z+N	-6,9	ns
TRADD-RIPK1-DKO TNF+C+N vs EV TNF	3,2	ns
TRADD-RIPK1-DKO TNF+C+N vs EV TNF+Z	-5,1	ns
TRADD-RIPK1-DKO TNF+C+N vs EV TNF+N	5,2	ns
TRADD-RIPK1-DKO TNF+C+N vs EV TNF+Z+N	-3,1	ns
TRADD-RIPK1-DKO TNF+C+N vs EV TNF+C	67	***
TRADD-RIPK1-DKO TNF+C+N vs EV TNF+C+Z	-9,1	ns
TRADD-RIPK1-DKO TNF+C+N vs EV TNF+C+N	56	***
TRADD-RIPK1-DKO TNF+C+N vs EV TNF+C+Z+N	-7,7	ns

TRADD-RIPK1-DKO TNF+C+Z+N vs Casp.8-KO TNF	9,3	ns
TRADD-RIPK1-DKO TNF+C+Z+N vs Casp.8-KO TNF+Z	7,0	ns
TRADD-RIPK1-DKO TNF+C+Z+N vs Casp.8-KO TNF+N	-0,37	ns
TRADD-RIPK1-DKO TNF+C+Z+N vs Casp.8-KO TNF+Z+N	-1,9	ns
TRADD-RIPK1-DKO TNF+C+Z+N vs Casp.8-KO TNF+C	36	***
TRADD-RIPK1-DKO TNF+C+Z+N vs Casp.8-KO TNF+C+Z	34	***
TRADD-RIPK1-DKO TNF+C+Z+N vs Casp.8-KO TNF+C+N	-0,13	ns
TRADD-RIPK1-DKO TNF+C+Z+N vs Casp.8-KO TNF+C+Z+N	1,3	ns
TRADD-RIPK1-DKO TNF+C+Z+N vs EV TNF	11	ns
TRADD-RIPK1-DKO TNF+C+Z+N vs EV TNF+Z	3,1	ns
TRADD-RIPK1-DKO TNF+C+Z+N vs EV TNF+N	13	ns
TRADD-RIPK1-DKO TNF+C+Z+N vs EV TNF+Z+N	5,1	ns
TRADD-RIPK1-DKO TNF+C+Z+N vs EV TNF+C	75	***
TRADD-RIPK1-DKO TNF+C+Z+N vs EV TNF+C+Z	-0,90	ns
TRADD-RIPK1-DKO TNF+C+Z+N vs EV TNF+C+N	64	***
TRADD-RIPK1-DKO TNF+C+Z+N vs EV TNF+C+Z+N	0,51	ns
Casp.8-KO TNF vs Casp.8-KO TNF+Z	-2,3	ns
Casp.8-KO TNF vs Casp.8-KO TNF+N	-9,7	ns
Casp.8-KO TNF vs Casp.8-KO TNF+Z+N	-11	ns
Casp.8-KO TNF vs Casp.8-KO TNF+C	26	***
Casp.8-KO TNF vs Casp.8-KO TNF+C+Z	25	***
Casp.8-KO TNF vs Casp.8-KO TNF+C+N	-9,4	ns
Casp.8-KO TNF vs Casp.8-KO TNF+C+Z+N	-8,0	ns
Casp.8-KO TNF vs EV TNF	2,1	ns
Casp.8-KO TNF vs EV TNF+Z	-6,2	ns
Casp.8-KO TNF vs EV TNF+N	4,2	ns
Casp.8-KO TNF vs EV TNF+Z+N	-4,2	ns
Casp.8-KO TNF vs EV TNF+C	66	***
Casp.8-KO TNF vs EV TNF+C+Z	-10	ns
Casp.8-KO TNF vs EV TNF+C+N	55	***
Casp.8-KO TNF vs EV TNF+C+Z+N	-8,8	ns
Casp.8-KO TNF+Z vs Casp.8-KO TNF+N	-7,4	ns
Casp.8-KO TNF+Z vs Casp.8-KO TNF+Z+N	-9,0	ns
Casp.8-KO TNF+Z vs Casp.8-KO TNF+C	29	***
Casp.8-KO TNF+Z vs Casp.8-KO TNF+C+Z	27	***
Casp.8-KO TNF+Z vs Casp.8-KO TNF+C+N	-7,2	ns
Casp.8-KO TNF+Z vs Casp.8-KO TNF+C+Z+N	-5,7	ns
Casp.8-KO TNF+Z vs EV TNF	4,3	ns
Casp.8-KO TNF+Z vs EV TNF+Z	-3,9	ns
Casp.8-KO TNF+Z vs EV TNF+N	6,4	ns
Casp.8-KO TNF+Z vs EV TNF+Z+N	-1,9	ns
Casp.8-KO TNF+Z vs EV TNF+C	68	***
Casp.8-KO TNF+Z vs EV TNF+C+Z	-7,9	ns
Casp.8-KO TNF+Z vs EV TNF+C+N	57	***
Casp.8-KO TNF+Z vs EV TNF+C+Z+N	-6,5	ns
Casp.8-KO TNF+N vs Casp.8-KO TNF+Z+N	-1,6	ns
Casp.8-KO TNF+N vs Casp.8-KO TNF+C	36	***
Casp.8-KO TNF+N vs Casp.8-KO TNF+C+Z	35	***
Casp.8-KO TNF+N vs Casp.8-KO TNF+C+N	0,23	ns
Casp.8-KO TNF+N vs Casp.8-KO TNF+C+Z+N	1,7	ns
Casp.8-KO TNF+N vs EV TNF	12	ns
Casp.8-KO TNF+N vs EV TNF+Z	3,5	ns
Casp.8-KO TNF+N vs EV TNF+N	14	ns
Casp.8-KO TNF+N vs EV TNF+Z+N	5,5	ns
Casp.8-KO TNF+N vs EV TNF+C	75	***
Casp.8-KO TNF+N vs EV TNF+C+Z	-0,53	ns
Casp.8-KO TNF+N vs EV TNF+C+N	64	***
Casp.8-KO TNF+N vs EV TNF+C+Z+N	0,88	ns
Casp.8-KO TNF+Z+N vs Casp.8-KO TNF+C	38	***
Casp.8-KO TNF+Z+N vs Casp.8-KO TNF+C+Z	36	***
Casp.8-KO TNF+Z+N vs Casp.8-KO TNF+C+N	1,8	ns
Casp.8-KO TNF+Z+N vs Casp.8-KO TNF+C+Z+N	3,3	ns
Casp.8-KO TNF+Z+N vs EV TNF	13	ns
Casp.8-KO TNF+Z+N vs EV TNF+Z	5,1	ns
Casp.8-KO TNF+Z+N vs EV TNF+N	15	ns
Casp.8-KO TNF+Z+N vs EV TNF+Z+N	7,1	ns

Casp.8-KO TNF+Z+N vs EV TNF+C	77	***
Casp.8-KO TNF+Z+N vs EV TNF+C+Z	1,1	ns
Casp.8-KO TNF+Z+N vs EV TNF+C+N	66	***
Casp.8-KO TNF+Z+N vs EV TNF+C+Z+N	2,5	ns
Casp.8-KO TNF+C vs Casp.8-KO TNF+C+Z	-1,3	ns
Casp.8-KO TNF+C vs Casp.8-KO TNF+C+N	-36	***
Casp.8-KO TNF+C vs Casp.8-KO TNF+C+Z+N	-34	***
Casp.8-KO TNF+C vs EV TNF	-24	***
Casp.8-KO TNF+C vs EV TNF+Z	-33	***
Casp.8-KO TNF+C vs EV TNF+N	-22	ns
Casp.8-KO TNF+C vs EV TNF+Z+N	-31	***
Casp.8-KO TNF+C vs EV TNF+C	39	***
Casp.8-KO TNF+C vs EV TNF+C+Z	-37	***
Casp.8-KO TNF+C vs EV TNF+C+N	28	***
Casp.8-KO TNF+C vs EV TNF+C+Z+N	-35	***
Casp.8-KO TNF+C+Z vs Casp.8-KO TNF+C+N	-35	***
Casp.8-KO TNF+C+Z vs Casp.8-KO TNF+C+Z+N	-33	***
Casp.8-KO TNF+C+Z vs EV TNF	-23	***
Casp.8-KO TNF+C+Z vs EV TNF+Z	-31	***
Casp.8-KO TNF+C+Z vs EV TNF+N	-21	ns
Casp.8-KO TNF+C+Z vs EV TNF+Z+N	-29	**
Casp.8-KO TNF+C+Z vs EV TNF+C	41	***
Casp.8-KO TNF+C+Z vs EV TNF+C+Z	-35	***
Casp.8-KO TNF+C+Z vs EV TNF+C+N	29	***
Casp.8-KO TNF+C+Z vs EV TNF+C+Z+N	-34	***
Casp.8-KO TNF+C+N vs Casp.8-KO TNF+C+Z+N	1,5	ns
Casp.8-KO TNF+C+N vs EV TNF	12	ns
Casp.8-KO TNF+C+N vs EV TNF+Z	3,3	ns
Casp.8-KO TNF+C+N vs EV TNF+N	14	ns
Casp.8-KO TNF+C+N vs EV TNF+Z+N	5,3	ns
Casp.8-KO TNF+C+N vs EV TNF+C	75	***
Casp.8-KO TNF+C+N vs EV TNF+C+Z	-0,77	ns
Casp.8-KO TNF+C+N vs EV TNF+C+N	64	***
Casp.8-KO TNF+C+N vs EV TNF+C+Z+N	0,65	ns
Casp.8-KO TNF+C+Z+N vs EV TNF	10	ns
Casp.8-KO TNF+C+Z+N vs EV TNF+Z	1,8	ns
Casp.8-KO TNF+C+Z+N vs EV TNF+N	12	ns
Casp.8-KO TNF+C+Z+N vs EV TNF+Z+N	3,8	ns
Casp.8-KO TNF+C+Z+N vs EV TNF+C	74	***
Casp.8-KO TNF+C+Z+N vs EV TNF+C+Z	-2,2	ns
Casp.8-KO TNF+C+Z+N vs EV TNF+C+N	62	***
Casp.8-KO TNF+C+Z+N vs EV TNF+C+Z+N	-0,82	ns
EV TNF vs EV TNF+Z	-8,2	ns
EV TNF vs EV TNF+N	2,1	ns
EV TNF vs EV TNF+Z+N	-6,2	ns
EV TNF vs EV TNF+C	64	***
EV TNF vs EV TNF+C+Z	-12	ns
EV TNF vs EV TNF+C+N	52	***
EV TNF vs EV TNF+C+Z+N	-11	ns
EV TNF+Z vs EV TNF+N	10	ns
EV TNF+Z vs EV TNF+Z+N	2,0	ns
EV TNF+Z vs EV TNF+C	72	***
EV TNF+Z vs EV TNF+C+Z	-4,0	ns
EV TNF+Z vs EV TNF+C+N	61	***
EV TNF+Z vs EV TNF+C+Z+N	-2,6	ns
EV TNF+N vs EV TNF+Z+N	-8,3	ns
EV TNF+N vs EV TNF+C	62	***
EV TNF+N vs EV TNF+C+Z	-14	ns
EV TNF+N vs EV TNF+C+N	50	***
EV TNF+N vs EV TNF+C+Z+N	-13	ns
EV TNF+Z+N vs EV TNF+C	70	***
EV TNF+Z+N vs EV TNF+C+Z	-6,0	ns
EV TNF+Z+N vs EV TNF+C+N	59	***
EV TNF+Z+N vs EV TNF+C+Z+N	-4,6	ns
EV TNF+C vs EV TNF+C+Z	-76	***
EV TNF+C vs EV TNF+C+N	-11	ns

EV TNF+C vs EV TNF+C+Z+N	-75	***
EV TNF+C+Z vs EV TNF+C+N	65	***
EV TNF+C+Z vs EV TNF+C+Z+N	1,4	ns
EV TNF+C+N vs EV TNF+C+Z+N	-63	***
