# Supplementary material for: Redundant and receptor-specific activities of TRADD, RIPK1 and FADD in death receptor signaling
Source: Cell Death Dis. 2019 Feb 11;10(2):122. doi: 10.1038/s41419-019-1396-5 (PMC6370826; doi:10.1038/s41419-019-1396-5)
Supplement: Supplementary file 3 — supplemental table III [file 41419_2019_1396_MOESM3_ESM.pdf]

Supplemental table III. Complete list of comparisons of TRAIL-treated cell variants.

Viability data of with 100 ng/ml TRAIL treated cells for all HeLa-RIPK3 variants and HeLa-EV cells and all co-treatment conditions were compiled and analyzed by ANOVA (one-way, Bonferroni comparison of all pairs of columns) using the GraphPad Prism5 software.

HeLa-RIPK3 = CON; HeLa-RIPK3-FADD$_{KO}$ = FADD-KO; HeLa-RIPK3-TRADD$_{KO}$ = TRADD-KO; HeLa-RIPK3-RIPK1$_{KO}$ = RIPK1-KO; HeLa-RIPK3-Casp8$_{KO}$ = Casp.8-KO; HeLa-RIPK3-FADD/TRADD$_{DKO}$ = FADD-TRADD-DKO; HeLa-RIPK3-FADD/RIPK1$_{DKO}$ = FADD-RIPK1-DKO; HeLa-RIPK3-TRADD/RIPK1$_{DKO}$ = TRADD-RIPK1-DKO; HeLa-EV = EV; Z = ZVAD; N = necrostatin-1; C = CHX; vs = versus; ns = non specific; *** = p < 0.001; ** = p < 0.01; * = p < 0.05.

Bonferroni's Multiple Comparison Test	Mean Diff. (%)	P value
CON vs CON TRAIL	13	***
CON vs CON TRAIL+Z	21	***
CON vs CON TRAIL+N	35	***
CON vs CON TRAIL+Z+N	5,4	ns
CON vs CON TRAIL+C	93	***
CON vs CON TRAIL+C+Z	72	***
CON vs CON TRAIL+C+N	94	***
CON vs CON TRAIL+C+Z+N	1,9	ns
FADD-KO vs FADD-KO TRAIL	3,2	ns
FADD-KO vs FADD-KO TRAIL+Z	8,5	ns
FADD-KO vs FADD-KO TRAIL+N	5,1	ns
FADD-KO vs FADD-KO TRAIL+Z+N	2,8	ns
FADD-KO vs FADD-KO TRAIL+C	12	**
FADD-KO vs FADD-KO TRAIL+C+Z	11	ns
FADD-KO vs FADD-KO TRAIL+C+N	11	ns
FADD-KO vs FADD-KO TRAIL+C+Z+N	7,8	ns
TRADD-KO vs TRADD-KO TRAIL	7,1	ns
TRADD-KO vs TRADD-KO TRAIL+Z	17	**
TRADD-KO vs TRADD-KO TRAIL+N	21	***
TRADD-KO vs TRADD-KO TRAIL+Z+N	-0,25	ns
TRADD-KO vs TRADD-KO TRAIL+C	92	***
TRADD-KO vs TRADD-KO TRAIL+C+Z	89	***
TRADD-KO vs TRADD-KO TRAIL+C+N	85	***
TRADD-KO vs TRADD-KO TRAIL+C+Z+N	3,6	ns
RIPK1-KO vs RIPK1-KO TRAIL	13	**
RIPK1-KO vs RIPK1-KO TRAIL+Z	3,5	ns
RIPK1-KO vs RIPK1-KO TRAIL+N	23	***
RIPK1-KO vs RIPK1-KO TRAIL+Z+N	6,2	ns
RIPK1-KO vs RIPK1-KO TRAIL+C	91	***
RIPK1-KO vs RIPK1-KO TRAIL+C+Z	5,0	ns
RIPK1-KO vs RIPK1-KO TRAIL+C+N	89	***
RIPK1-KO vs RIPK1-KO TRAIL+C+Z+N	5,1	ns
FADD-TRADD-DKO vs FADD-TRADD-DKO TRAIL	19	***
FADD-TRADD-DKO vs FADD-TRADD-DKO TRAIL+Z	24	***
FADD-TRADD-DKO vs FADD-TRADD-DKO TRAIL+N	23	***
FADD-TRADD-DKO vs FADD-TRADD-DKO TRAIL+Z+N	5,1	ns
FADD-TRADD-DKO vs FADD-TRADD-DKO TRAIL+C	28	***
FADD-TRADD-DKO vs FADD-TRADD-DKO TRAIL+C+Z	5,2	ns
FADD-TRADD-DKO vs FADD-TRADD-DKO TRAIL+C+N	25	***
FADD-TRADD-DKO vs FADD-TRADD-DKO TRAIL+C+Z+N	6,7	ns
FADD-RIPK1-DKO vs FADD-RIPK1-DKO TRAIL	0,83	ns

FADD-RIPK1-DKO vs FADD-RIPK1-DKO TRAIL+Z	2,4	ns
FADD-RIPK1-DKO vs FADD-RIPK1-DKO TRAIL+N	7,6	ns
FADD-RIPK1-DKO vs FADD-RIPK1-DKO TRAIL+Z+N	4,4	ns
FADD-RIPK1-DKO vs FADD-RIPK1-DKO TRAIL+C	13	ns
FADD-RIPK1-DKO vs FADD-RIPK1-DKO TRAIL+C+Z	12	ns
FADD-RIPK1-DKO vs FADD-RIPK1-DKO TRAIL+C+N	13	ns
FADD-RIPK1-DKO vs FADD-RIPK1-DKO TRAIL+C+Z+N	5,3	ns
TRADD-RIPK1-DKO vs TRADD-RIPK1-DKO TRAIL	23	***
TRADD-RIPK1-DKO vs TRADD-RIPK1-DKO TRAIL+Z	8,1	ns
TRADD-RIPK1-DKO vs TRADD-RIPK1-DKO TRAIL+N	32	***
TRADD-RIPK1-DKO vs TRADD-RIPK1-DKO TRAIL+Z+N	1,8	ns
TRADD-RIPK1-DKO vs TRADD-RIPK1-DKO TRAIL+C	88	***
TRADD-RIPK1-DKO vs TRADD-RIPK1-DKO TRAIL+C+Z	1,9	ns
TRADD-RIPK1-DKO vs TRADD-RIPK1-DKO TRAIL+C+N	89	***
TRADD-RIPK1-DKO vs TRADD-RIPK1-DKO TRAIL+C+Z+N	2,9	ns
Casp.8-KO vs Casp.8-KO TRAIL	14	*
Casp.8-KO vs Casp.8-KO TRAIL+Z	23	***
Casp.8-KO vs Casp.8-KO TRAIL+N	1,4	ns
Casp.8-KO vs Casp.8-KO TRAIL+Z+N	-0,11	ns
Casp.8-KO vs Casp.8-KO TRAIL+C	35	***
Casp.8-KO vs Casp.8-KO TRAIL+C+Z	38	***
Casp.8-KO vs Casp.8-KO TRAIL+C+N	8,5	ns
Casp.8-KO vs Casp.8-KO TRAIL+C+Z+N	3,4	ns
EV vs EV TRAIL	3,1	ns
EV vs EV TRAIL+Z	2,0	ns
EV vs EV TRAIL+N	19	ns
EV vs EV TRAIL+Z+N	7,0	ns
EV vs EV TRAIL+C	79	***
EV vs EV TRAIL+C+Z	3,6	ns
EV vs EV TRAIL+C+N	83	***
EV vs EV TRAIL+C+Z+N	6,8	ns
CON TRAIL vs CON TRAIL+Z	8,3	ns
CON TRAIL vs CON TRAIL+N	22	***
CON TRAIL vs CON TRAIL+Z+N	-7,5	ns
CON TRAIL vs CON TRAIL+C	80	***
CON TRAIL vs CON TRAIL+C+Z	59	***
CON TRAIL vs CON TRAIL+C+N	81	***
CON TRAIL vs CON TRAIL+C+Z+N	-11	ns
CON TRAIL vs FADD-KO TRAIL	-9,7	ns
CON TRAIL vs FADD-KO TRAIL+Z	-4,4	ns
CON TRAIL vs FADD-KO TRAIL+N	-7,8	ns
CON TRAIL vs FADD-KO TRAIL+Z+N	-10	ns
CON TRAIL vs FADD-KO TRAIL+C	-0,49	ns
CON TRAIL vs FADD-KO TRAIL+C+Z	-2,3	ns
CON TRAIL vs FADD-KO TRAIL+C+N	-1,9	ns
CON TRAIL vs FADD-KO TRAIL+C+Z+N	-5,1	ns
CON TRAIL vs TRADD-KO TRAIL	-5,8	ns
CON TRAIL vs TRADD-KO TRAIL+Z	3,7	ns
CON TRAIL vs TRADD-KO TRAIL+N	7,9	ns
CON TRAIL vs TRADD-KO TRAIL+Z+N	-13	ns
CON TRAIL vs TRADD-KO TRAIL+C	79	***
CON TRAIL vs TRADD-KO TRAIL+C+Z	76	***
CON TRAIL vs TRADD-KO TRAIL+C+N	72	***
CON TRAIL vs TRADD-KO TRAIL+C+Z+N	-9,3	ns

CON TRAIL vs RIPK1-KO TRAIL	0,55	ns
CON TRAIL vs RIPK1-KO TRAIL+Z	-9,3	ns
CON TRAIL vs RIPK1-KO TRAIL+N	10	ns
CON TRAIL vs RIPK1-KO TRAIL+Z+N	-6,7	ns
CON TRAIL vs RIPK1-KO TRAIL+C	78	***
CON TRAIL vs RIPK1-KO TRAIL+C+Z	-7,9	ns
CON TRAIL vs RIPK1-KO TRAIL+C+N	76	***
CON TRAIL vs RIPK1-KO TRAIL+C+Z+N	-7,8	ns
CON TRAIL vs FADD-TRADD-DKO TRAIL	6,4	ns
CON TRAIL vs FADD-TRADD-DKO TRAIL+Z	11	ns
CON TRAIL vs FADD-TRADD-DKO TRAIL+N	10	ns
CON TRAIL vs FADD-TRADD-DKO TRAIL+Z+N	-7,7	ns
CON TRAIL vs FADD-TRADD-DKO TRAIL+C	16	ns
CON TRAIL vs FADD-TRADD-DKO TRAIL+C+Z	-7,7	ns
CON TRAIL vs FADD-TRADD-DKO TRAIL+C+N	12	ns
CON TRAIL vs FADD-TRADD-DKO TRAIL+C+Z+N	-6,2	ns
CON TRAIL vs FADD-RIPK1-DKO TRAIL	-12	ns
CON TRAIL vs FADD-RIPK1-DKO TRAIL+Z	-10	ns
CON TRAIL vs FADD-RIPK1-DKO TRAIL+N	-5,3	ns
CON TRAIL vs FADD-RIPK1-DKO TRAIL+Z+N	-8,5	ns
CON TRAIL vs FADD-RIPK1-DKO TRAIL+C	-0,022	ns
CON TRAIL vs FADD-RIPK1-DKO TRAIL+C+Z	-0,61	ns
CON TRAIL vs FADD-RIPK1-DKO TRAIL+C+N	-0,30	ns
CON TRAIL vs FADD-RIPK1-DKO TRAIL+C+Z+N	-7,6	ns
CON TRAIL vs TRADD-RIPK1-DKO TRAIL	10	ns
CON TRAIL vs TRADD-RIPK1-DKO TRAIL+Z	-4,8	ns
CON TRAIL vs TRADD-RIPK1-DKO TRAIL+N	19	***
CON TRAIL vs TRADD-RIPK1-DKO TRAIL+Z+N	-11	ns
CON TRAIL vs TRADD-RIPK1-DKO TRAIL+C	75	***
CON TRAIL vs TRADD-RIPK1-DKO TRAIL+C+Z	-11	ns
CON TRAIL vs TRADD-RIPK1-DKO TRAIL+C+N	76	***
CON TRAIL vs TRADD-RIPK1-DKO TRAIL+C+Z+N	-10	ns
CON TRAIL vs Casp.8-KO TRAIL	1,2	ns
CON TRAIL vs Casp.8-KO TRAIL+Z	10	ns
CON TRAIL vs Casp.8-KO TRAIL+N	-11	ns
CON TRAIL vs Casp.8-KO TRAIL+Z+N	-13	ns
CON TRAIL vs Casp.8-KO TRAIL+C	22	***
CON TRAIL vs Casp.8-KO TRAIL+C+Z	25	***
CON TRAIL vs Casp.8-KO TRAIL+C+N	-4,4	ns
CON TRAIL vs Casp.8-KO TRAIL+C+Z+N	-9,5	ns
CON TRAIL vs EV TRAIL	-9,8	ns
CON TRAIL vs EV TRAIL+Z	-11	ns
CON TRAIL vs EV TRAIL+N	6,1	ns
CON TRAIL vs EV TRAIL+Z+N	-5,9	ns
CON TRAIL vs EV TRAIL+C	66	***
CON TRAIL vs EV TRAIL+C+Z	-9,3	ns
CON TRAIL vs EV TRAIL+C+N	70	***
CON TRAIL vs EV TRAIL+C+Z+N	-6,1	ns
CON TRAIL+Z vs CON TRAIL+N	14	*
CON TRAIL+Z vs CON TRAIL+Z+N	-16	***
CON TRAIL+Z vs CON TRAIL+C	72	***
CON TRAIL+Z vs CON TRAIL+C+Z	51	***
CON TRAIL+Z vs CON TRAIL+C+N	73	***
CON TRAIL+Z vs CON TRAIL+C+Z+N	-19	***

CON TRAIL+Z vs FADD-KO TRAIL	-18	***
CON TRAIL+Z vs FADD-KO TRAIL+Z	-13	**
CON TRAIL+Z vs FADD-KO TRAIL+N	-16	***
CON TRAIL+Z vs FADD-KO TRAIL+Z+N	-18	***
CON TRAIL+Z vs FADD-KO TRAIL+C	-8,8	ns
CON TRAIL+Z vs FADD-KO TRAIL+C+Z	-11	ns
CON TRAIL+Z vs FADD-KO TRAIL+C+N	-10	ns
CON TRAIL+Z vs FADD-KO TRAIL+C+Z+N	-13	*
CON TRAIL+Z vs TRADD-KO TRAIL	-14	*
CON TRAIL+Z vs TRADD-KO TRAIL+Z	-4,6	ns
CON TRAIL+Z vs TRADD-KO TRAIL+N	-0,41	ns
CON TRAIL+Z vs TRADD-KO TRAIL+Z+N	-21	***
CON TRAIL+Z vs TRADD-KO TRAIL+C	70	***
CON TRAIL+Z vs TRADD-KO TRAIL+C+Z	68	***
CON TRAIL+Z vs TRADD-KO TRAIL+C+N	64	***
CON TRAIL+Z vs TRADD-KO TRAIL+C+Z+N	-18	**
CON TRAIL+Z vs RIPK1-KO TRAIL	-7,8	ns
CON TRAIL+Z vs RIPK1-KO TRAIL+Z	-18	***
CON TRAIL+Z vs RIPK1-KO TRAIL+N	1,7	ns
CON TRAIL+Z vs RIPK1-KO TRAIL+Z+N	-15	**
CON TRAIL+Z vs RIPK1-KO TRAIL+C	69	***
CON TRAIL+Z vs RIPK1-KO TRAIL+C+Z	-16	**
CON TRAIL+Z vs RIPK1-KO TRAIL+C+N	68	***
CON TRAIL+Z vs RIPK1-KO TRAIL+C+Z+N	-16	*
CON TRAIL+Z vs FADD-TRADD-DKO TRAIL	-1,9	ns
CON TRAIL+Z vs FADD-TRADD-DKO TRAIL+Z	2,4	ns
CON TRAIL+Z vs FADD-TRADD-DKO TRAIL+N	1,9	ns
CON TRAIL+Z vs FADD-TRADD-DKO TRAIL+Z+N	-16	**
CON TRAIL+Z vs FADD-TRADD-DKO TRAIL+C	7,3	ns
CON TRAIL+Z vs FADD-TRADD-DKO TRAIL+C+Z	-16	ns
CON TRAIL+Z vs FADD-TRADD-DKO TRAIL+C+N	3,7	ns
CON TRAIL+Z vs FADD-TRADD-DKO TRAIL+C+Z+N	-14	ns
CON TRAIL+Z vs FADD-RIPK1-DKO TRAIL	-20	***
CON TRAIL+Z vs FADD-RIPK1-DKO TRAIL+Z	-19	***
CON TRAIL+Z vs FADD-RIPK1-DKO TRAIL+N	-14	*
CON TRAIL+Z vs FADD-RIPK1-DKO TRAIL+Z+N	-17	***
CON TRAIL+Z vs FADD-RIPK1-DKO TRAIL+C	-8,3	ns
CON TRAIL+Z vs FADD-RIPK1-DKO TRAIL+C+Z	-8,9	ns
CON TRAIL+Z vs FADD-RIPK1-DKO TRAIL+C+N	-8,6	ns
CON TRAIL+Z vs FADD-RIPK1-DKO TRAIL+C+Z+N	-16	*
CON TRAIL+Z vs TRADD-RIPK1-DKO TRAIL	1,9	ns
CON TRAIL+Z vs TRADD-RIPK1-DKO TRAIL+Z	-13	ns
CON TRAIL+Z vs TRADD-RIPK1-DKO TRAIL+N	11	ns
CON TRAIL+Z vs TRADD-RIPK1-DKO TRAIL+Z+N	-19	***
CON TRAIL+Z vs TRADD-RIPK1-DKO TRAIL+C	67	***
CON TRAIL+Z vs TRADD-RIPK1-DKO TRAIL+C+Z	-19	**
CON TRAIL+Z vs TRADD-RIPK1-DKO TRAIL+C+N	67	***
CON TRAIL+Z vs TRADD-RIPK1-DKO TRAIL+C+Z+N	-18	***
CON TRAIL+Z vs Casp.8-KO TRAIL	-7,1	ns
CON TRAIL+Z vs Casp.8-KO TRAIL+Z	1,8	ns
CON TRAIL+Z vs Casp.8-KO TRAIL+N	-20	***
CON TRAIL+Z vs Casp.8-KO TRAIL+Z+N	-21	***
CON TRAIL+Z vs Casp.8-KO TRAIL+C	14	ns
CON TRAIL+Z vs Casp.8-KO TRAIL+C+Z	17	ns

CON TRAIL+Z vs Casp.8-KO TRAIL+C+N	-13	ns
CON TRAIL+Z vs Casp.8-KO TRAIL+C+Z+N	-18	**
CON TRAIL+Z vs EV TRAIL	-18	*
CON TRAIL+Z vs EV TRAIL+Z	-19	*
CON TRAIL+Z vs EV TRAIL+N	-2,2	ns
CON TRAIL+Z vs EV TRAIL+Z+N	-14	ns
CON TRAIL+Z vs EV TRAIL+C	58	***
CON TRAIL+Z vs EV TRAIL+C+Z	-18	ns
CON TRAIL+Z vs EV TRAIL+C+N	61	***
CON TRAIL+Z vs EV TRAIL+C+Z+N	-14	ns
CON TRAIL+N vs CON TRAIL+Z+N	-29	***
CON TRAIL+N vs CON TRAIL+C	58	***
CON TRAIL+N vs CON TRAIL+C+Z	37	***
CON TRAIL+N vs CON TRAIL+C+N	59	***
CON TRAIL+N vs CON TRAIL+C+Z+N	-33	***
CON TRAIL+N vs FADD-KO TRAIL	-32	***
CON TRAIL+N vs FADD-KO TRAIL+Z	-26	***
CON TRAIL+N vs FADD-KO TRAIL+N	-30	***
CON TRAIL+N vs FADD-KO TRAIL+Z+N	-32	***
CON TRAIL+N vs FADD-KO TRAIL+C	-22	***
CON TRAIL+N vs FADD-KO TRAIL+C+Z	-24	***
CON TRAIL+N vs FADD-KO TRAIL+C+N	-24	***
CON TRAIL+N vs FADD-KO TRAIL+C+Z+N	-27	***
CON TRAIL+N vs TRADD-KO TRAIL	-28	***
CON TRAIL+N vs TRADD-KO TRAIL+Z	-18	**
CON TRAIL+N vs TRADD-KO TRAIL+N	-14	ns
CON TRAIL+N vs TRADD-KO TRAIL+Z+N	-35	***
CON TRAIL+N vs TRADD-KO TRAIL+C	57	***
CON TRAIL+N vs TRADD-KO TRAIL+C+Z	54	***
CON TRAIL+N vs TRADD-KO TRAIL+C+N	50	***
CON TRAIL+N vs TRADD-KO TRAIL+C+Z+N	-31	***
CON TRAIL+N vs RIPK1-KO TRAIL	-21	***
CON TRAIL+N vs RIPK1-KO TRAIL+Z	-31	***
CON TRAIL+N vs RIPK1-KO TRAIL+N	-12	ns
CON TRAIL+N vs RIPK1-KO TRAIL+Z+N	-29	***
CON TRAIL+N vs RIPK1-KO TRAIL+C	56	***
CON TRAIL+N vs RIPK1-KO TRAIL+C+Z	-30	***
CON TRAIL+N vs RIPK1-KO TRAIL+C+N	54	***
CON TRAIL+N vs RIPK1-KO TRAIL+C+Z+N	-30	***
CON TRAIL+N vs FADD-TRADD-DKO TRAIL	-15	ns
CON TRAIL+N vs FADD-TRADD-DKO TRAIL+Z	-11	ns
CON TRAIL+N vs FADD-TRADD-DKO TRAIL+N	-12	ns
CON TRAIL+N vs FADD-TRADD-DKO TRAIL+Z+N	-30	***
CON TRAIL+N vs FADD-TRADD-DKO TRAIL+C	-6,3	ns
CON TRAIL+N vs FADD-TRADD-DKO TRAIL+C+Z	-30	***
CON TRAIL+N vs FADD-TRADD-DKO TRAIL+C+N	-9,9	ns
CON TRAIL+N vs FADD-TRADD-DKO TRAIL+C+Z+N	-28	***
CON TRAIL+N vs FADD-RIPK1-DKO TRAIL	-34	***
CON TRAIL+N vs FADD-RIPK1-DKO TRAIL+Z	-32	***
CON TRAIL+N vs FADD-RIPK1-DKO TRAIL+N	-27	***
CON TRAIL+N vs FADD-RIPK1-DKO TRAIL+Z+N	-30	***
CON TRAIL+N vs FADD-RIPK1-DKO TRAIL+C	-22	***
CON TRAIL+N vs FADD-RIPK1-DKO TRAIL+C+Z	-22	***
CON TRAIL+N vs FADD-RIPK1-DKO TRAIL+C+N	-22	***

CON TRAIL+N vs FADD-RIPK1-DKO TRAIL+C+Z+N	-29	***
CON TRAIL+N vs TRADD-RIPK1-DKO TRAIL	-12	ns
CON TRAIL+N vs TRADD-RIPK1-DKO TRAIL+Z	-27	***
CON TRAIL+N vs TRADD-RIPK1-DKO TRAIL+N	-2,8	ns
CON TRAIL+N vs TRADD-RIPK1-DKO TRAIL+Z+N	-33	***
CON TRAIL+N vs TRADD-RIPK1-DKO TRAIL+C	53	***
CON TRAIL+N vs TRADD-RIPK1-DKO TRAIL+C+Z	-33	***
CON TRAIL+N vs TRADD-RIPK1-DKO TRAIL+C+N	54	***
CON TRAIL+N vs TRADD-RIPK1-DKO TRAIL+C+Z+N	-32	***
CON TRAIL+N vs Casp.8-KO TRAIL	-21	***
CON TRAIL+N vs Casp.8-KO TRAIL+Z	-12	ns
CON TRAIL+N vs Casp.8-KO TRAIL+N	-33	***
CON TRAIL+N vs Casp.8-KO TRAIL+Z+N	-35	***
CON TRAIL+N vs Casp.8-KO TRAIL+C	0,24	ns
CON TRAIL+N vs Casp.8-KO TRAIL+C+Z	3,0	ns
CON TRAIL+N vs Casp.8-KO TRAIL+C+N	-26	***
CON TRAIL+N vs Casp.8-KO TRAIL+C+Z+N	-31	***
CON TRAIL+N vs EV TRAIL	-32	***
CON TRAIL+N vs EV TRAIL+Z	-33	***
CON TRAIL+N vs EV TRAIL+N	-16	ns
CON TRAIL+N vs EV TRAIL+Z+N	-28	**
CON TRAIL+N vs EV TRAIL+C	45	***
CON TRAIL+N vs EV TRAIL+C+Z	-31	***
CON TRAIL+N vs EV TRAIL+C+N	48	***
CON TRAIL+N vs EV TRAIL+C+Z+N	-28	***
CON TRAIL+Z+N vs CON TRAIL+C	88	***
CON TRAIL+Z+N vs CON TRAIL+C+Z	67	***
CON TRAIL+Z+N vs CON TRAIL+C+N	88	***
CON TRAIL+Z+N vs CON TRAIL+C+Z+N	-3,5	ns
CON TRAIL+Z+N vs FADD-KO TRAIL	-2,2	ns
CON TRAIL+Z+N vs FADD-KO TRAIL+Z	3,1	ns
CON TRAIL+Z+N vs FADD-KO TRAIL+N	-0,32	ns
CON TRAIL+Z+N vs FADD-KO TRAIL+Z+N	-2,6	ns
CON TRAIL+Z+N vs FADD-KO TRAIL+C	7,0	ns
CON TRAIL+Z+N vs FADD-KO TRAIL+C+Z	5,1	ns
CON TRAIL+Z+N vs FADD-KO TRAIL+C+N	5,6	ns
CON TRAIL+Z+N vs FADD-KO TRAIL+C+Z+N	2,4	ns
CON TRAIL+Z+N vs TRADD-KO TRAIL	1,7	ns
CON TRAIL+Z+N vs TRADD-KO TRAIL+Z	11	ns
CON TRAIL+Z+N vs TRADD-KO TRAIL+N	15	ns
CON TRAIL+Z+N vs TRADD-KO TRAIL+Z+N	-5,7	ns
CON TRAIL+Z+N vs TRADD-KO TRAIL+C	86	***
CON TRAIL+Z+N vs TRADD-KO TRAIL+C+Z	83	***
CON TRAIL+Z+N vs TRADD-KO TRAIL+C+N	80	***
CON TRAIL+Z+N vs TRADD-KO TRAIL+C+Z+N	-1,8	ns
CON TRAIL+Z+N vs RIPK1-KO TRAIL	8,0	ns
CON TRAIL+Z+N vs RIPK1-KO TRAIL+Z	-1,9	ns
CON TRAIL+Z+N vs RIPK1-KO TRAIL+N	18	***
CON TRAIL+Z+N vs RIPK1-KO TRAIL+Z+N	0,79	ns
CON TRAIL+Z+N vs RIPK1-KO TRAIL+C	85	***
CON TRAIL+Z+N vs RIPK1-KO TRAIL+C+Z	-0,40	ns
CON TRAIL+Z+N vs RIPK1-KO TRAIL+C+N	83	***
CON TRAIL+Z+N vs RIPK1-KO TRAIL+C+Z+N	-0,31	ns
CON TRAIL+Z+N vs FADD-TRADD-DKO TRAIL	14	ns

CON TRAIL+Z+N vs FADD-TRADD-DKO TRAIL+Z	18	***
CON TRAIL+Z+N vs FADD-TRADD-DKO TRAIL+N	18	**
CON TRAIL+Z+N vs FADD-TRADD-DKO TRAIL+Z+N	-0,27	ns
CON TRAIL+Z+N vs FADD-TRADD-DKO TRAIL+C	23	***
CON TRAIL+Z+N vs FADD-TRADD-DKO TRAIL+C+Z	-0,20	ns
CON TRAIL+Z+N vs FADD-TRADD-DKO TRAIL+C+N	19	*
CON TRAIL+Z+N vs FADD-TRADD-DKO TRAIL+C+Z+N	1,3	ns
CON TRAIL+Z+N vs FADD-RIPK1-DKO TRAIL	-4,6	ns
CON TRAIL+Z+N vs FADD-RIPK1-DKO TRAIL+Z	-3,0	ns
CON TRAIL+Z+N vs FADD-RIPK1-DKO TRAIL+N	2,2	ns
CON TRAIL+Z+N vs FADD-RIPK1-DKO TRAIL+Z+N	-1,0	ns
CON TRAIL+Z+N vs FADD-RIPK1-DKO TRAIL+C	7,5	ns
CON TRAIL+Z+N vs FADD-RIPK1-DKO TRAIL+C+Z	6,9	ns
CON TRAIL+Z+N vs FADD-RIPK1-DKO TRAIL+C+N	7,2	ns
CON TRAIL+Z+N vs FADD-RIPK1-DKO TRAIL+C+Z+N	-0,10	ns
CON TRAIL+Z+N vs TRADD-RIPK1-DKO TRAIL	18	***
CON TRAIL+Z+N vs TRADD-RIPK1-DKO TRAIL+Z	2,7	ns
CON TRAIL+Z+N vs TRADD-RIPK1-DKO TRAIL+N	27	***
CON TRAIL+Z+N vs TRADD-RIPK1-DKO TRAIL+Z+N	-3,6	ns
CON TRAIL+Z+N vs TRADD-RIPK1-DKO TRAIL+C	82	***
CON TRAIL+Z+N vs TRADD-RIPK1-DKO TRAIL+C+Z	-3,5	ns
CON TRAIL+Z+N vs TRADD-RIPK1-DKO TRAIL+C+N	83	***
CON TRAIL+Z+N vs TRADD-RIPK1-DKO TRAIL+C+Z+N	-2,5	ns
CON TRAIL+Z+N vs Casp.8-KO TRAIL	8,7	ns
CON TRAIL+Z+N vs Casp.8-KO TRAIL+Z	18	***
CON TRAIL+Z+N vs Casp.8-KO TRAIL+N	-4,0	ns
CON TRAIL+Z+N vs Casp.8-KO TRAIL+Z+N	-5,5	ns
CON TRAIL+Z+N vs Casp.8-KO TRAIL+C	30	***
CON TRAIL+Z+N vs Casp.8-KO TRAIL+C+Z	32	***
CON TRAIL+Z+N vs Casp.8-KO TRAIL+C+N	3,1	ns
CON TRAIL+Z+N vs Casp.8-KO TRAIL+C+Z+N	-2,0	ns
CON TRAIL+Z+N vs EV TRAIL	-2,3	ns
CON TRAIL+Z+N vs EV TRAIL+Z	-3,4	ns
CON TRAIL+Z+N vs EV TRAIL+N	14	ns
CON TRAIL+Z+N vs EV TRAIL+Z+N	1,6	ns
CON TRAIL+Z+N vs EV TRAIL+C	74	***
CON TRAIL+Z+N vs EV TRAIL+C+Z	-1,8	ns
CON TRAIL+Z+N vs EV TRAIL+C+N	77	***
CON TRAIL+Z+N vs EV TRAIL+C+Z+N	1,3	ns
CON TRAIL+C vs CON TRAIL+C+Z	-21	**
CON TRAIL+C vs CON TRAIL+C+N	0,75	ns
CON TRAIL+C vs CON TRAIL+C+Z+N	-91	***
CON TRAIL+C vs FADD-KO TRAIL	-90	***
CON TRAIL+C vs FADD-KO TRAIL+Z	-85	***
CON TRAIL+C vs FADD-KO TRAIL+N	-88	***
CON TRAIL+C vs FADD-KO TRAIL+Z+N	-90	***
CON TRAIL+C vs FADD-KO TRAIL+C	-81	***
CON TRAIL+C vs FADD-KO TRAIL+C+Z	-83	***
CON TRAIL+C vs FADD-KO TRAIL+C+N	-82	***
CON TRAIL+C vs FADD-KO TRAIL+C+Z+N	-85	***
CON TRAIL+C vs TRADD-KO TRAIL	-86	***
CON TRAIL+C vs TRADD-KO TRAIL+Z	-76	***
CON TRAIL+C vs TRADD-KO TRAIL+N	-72	***
CON TRAIL+C vs TRADD-KO TRAIL+Z+N	-93	***

CON TRAIL+C vs TRADD-KO TRAIL+C	-1,4	ns
CON TRAIL+C vs TRADD-KO TRAIL+C+Z	-4,2	ns
CON TRAIL+C vs TRADD-KO TRAIL+C+N	-8,1	ns
CON TRAIL+C vs TRADD-KO TRAIL+C+Z+N	-89	***
CON TRAIL+C vs RIPK1-KO TRAIL	-80	***
CON TRAIL+C vs RIPK1-KO TRAIL+Z	-90	***
CON TRAIL+C vs RIPK1-KO TRAIL+N	-70	***
CON TRAIL+C vs RIPK1-KO TRAIL+Z+N	-87	***
CON TRAIL+C vs RIPK1-KO TRAIL+C	-2,5	ns
CON TRAIL+C vs RIPK1-KO TRAIL+C+Z	-88	***
CON TRAIL+C vs RIPK1-KO TRAIL+C+N	-4,3	ns
CON TRAIL+C vs RIPK1-KO TRAIL+C+Z+N	-88	***
CON TRAIL+C vs FADD-TRADD-DKO TRAIL	-74	***
CON TRAIL+C vs FADD-TRADD-DKO TRAIL+Z	-70	***
CON TRAIL+C vs FADD-TRADD-DKO TRAIL+N	-70	***
CON TRAIL+C vs FADD-TRADD-DKO TRAIL+Z+N	-88	***
CON TRAIL+C vs FADD-TRADD-DKO TRAIL+C	-65	***
CON TRAIL+C vs FADD-TRADD-DKO TRAIL+C+Z	-88	***
CON TRAIL+C vs FADD-TRADD-DKO TRAIL+C+N	-68	***
CON TRAIL+C vs FADD-TRADD-DKO TRAIL+C+Z+N	-86	***
CON TRAIL+C vs FADD-RIPK1-DKO TRAIL	-92	***
CON TRAIL+C vs FADD-RIPK1-DKO TRAIL+Z	-91	***
CON TRAIL+C vs FADD-RIPK1-DKO TRAIL+N	-85	***
CON TRAIL+C vs FADD-RIPK1-DKO TRAIL+Z+N	-89	***
CON TRAIL+C vs FADD-RIPK1-DKO TRAIL+C	-80	***
CON TRAIL+C vs FADD-RIPK1-DKO TRAIL+C+Z	-81	***
CON TRAIL+C vs FADD-RIPK1-DKO TRAIL+C+N	-80	***
CON TRAIL+C vs FADD-RIPK1-DKO TRAIL+C+Z+N	-88	***
CON TRAIL+C vs TRADD-RIPK1-DKO TRAIL	-70	***
CON TRAIL+C vs TRADD-RIPK1-DKO TRAIL+Z	-85	***
CON TRAIL+C vs TRADD-RIPK1-DKO TRAIL+N	-61	***
CON TRAIL+C vs TRADD-RIPK1-DKO TRAIL+Z+N	-91	***
CON TRAIL+C vs TRADD-RIPK1-DKO TRAIL+C	-5,3	ns
CON TRAIL+C vs TRADD-RIPK1-DKO TRAIL+C+Z	-91	***
CON TRAIL+C vs TRADD-RIPK1-DKO TRAIL+C+N	-4,6	ns
CON TRAIL+C vs TRADD-RIPK1-DKO TRAIL+C+Z+N	-90	***
CON TRAIL+C vs Casp.8-KO TRAIL	-79	***
CON TRAIL+C vs Casp.8-KO TRAIL+Z	-70	***
CON TRAIL+C vs Casp.8-KO TRAIL+N	-92	***
CON TRAIL+C vs Casp.8-KO TRAIL+Z+N	-93	***
CON TRAIL+C vs Casp.8-KO TRAIL+C	-58	***
CON TRAIL+C vs Casp.8-KO TRAIL+C+Z	-55	***
CON TRAIL+C vs Casp.8-KO TRAIL+C+N	-85	***
CON TRAIL+C vs Casp.8-KO TRAIL+C+Z+N	-90	***
CON TRAIL+C vs EV TRAIL	-90	***
CON TRAIL+C vs EV TRAIL+Z	-91	***
CON TRAIL+C vs EV TRAIL+N	-74	***
CON TRAIL+C vs EV TRAIL+Z+N	-86	***
CON TRAIL+C vs EV TRAIL+C	-14	ns
CON TRAIL+C vs EV TRAIL+C+Z	-89	***
CON TRAIL+C vs EV TRAIL+C+N	-11	ns
CON TRAIL+C vs EV TRAIL+C+Z+N	-86	***
CON TRAIL+C+Z vs CON TRAIL+C+N	22	ns
CON TRAIL+C+Z vs CON TRAIL+C+Z+N	-70	***

CON TRAIL+C+Z vs FADD-KO TRAIL	-69	***
CON TRAIL+C+Z vs FADD-KO TRAIL+Z	-64	***
CON TRAIL+C+Z vs FADD-KO TRAIL+N	-67	***
CON TRAIL+C+Z vs FADD-KO TRAIL+Z+N	-69	***
CON TRAIL+C+Z vs FADD-KO TRAIL+C	-60	***
CON TRAIL+C+Z vs FADD-KO TRAIL+C+Z	-61	***
CON TRAIL+C+Z vs FADD-KO TRAIL+C+N	-61	***
CON TRAIL+C+Z vs FADD-KO TRAIL+C+Z+N	-64	***
CON TRAIL+C+Z vs TRADD-KO TRAIL	-65	***
CON TRAIL+C+Z vs TRADD-KO TRAIL+Z	-55	***
CON TRAIL+C+Z vs TRADD-KO TRAIL+N	-51	***
CON TRAIL+C+Z vs TRADD-KO TRAIL+Z+N	-72	***
CON TRAIL+C+Z vs TRADD-KO TRAIL+C	20	ns
CON TRAIL+C+Z vs TRADD-KO TRAIL+C+Z	17	ns
CON TRAIL+C+Z vs TRADD-KO TRAIL+C+N	13	ns
CON TRAIL+C+Z vs TRADD-KO TRAIL+C+Z+N	-68	***
CON TRAIL+C+Z vs RIPK1-KO TRAIL	-59	***
CON TRAIL+C+Z vs RIPK1-KO TRAIL+Z	-68	***
CON TRAIL+C+Z vs RIPK1-KO TRAIL+N	-49	***
CON TRAIL+C+Z vs RIPK1-KO TRAIL+Z+N	-66	***
CON TRAIL+C+Z vs RIPK1-KO TRAIL+C	19	ns
CON TRAIL+C+Z vs RIPK1-KO TRAIL+C+Z	-67	***
CON TRAIL+C+Z vs RIPK1-KO TRAIL+C+N	17	ns
CON TRAIL+C+Z vs RIPK1-KO TRAIL+C+Z+N	-67	***
CON TRAIL+C+Z vs FADD-TRADD-DKO TRAIL	-53	***
CON TRAIL+C+Z vs FADD-TRADD-DKO TRAIL+Z	-48	***
CON TRAIL+C+Z vs FADD-TRADD-DKO TRAIL+N	-49	***
CON TRAIL+C+Z vs FADD-TRADD-DKO TRAIL+Z+N	-67	***
CON TRAIL+C+Z vs FADD-TRADD-DKO TRAIL+C	-44	***
CON TRAIL+C+Z vs FADD-TRADD-DKO TRAIL+C+Z	-67	***
CON TRAIL+C+Z vs FADD-TRADD-DKO TRAIL+C+N	-47	***
CON TRAIL+C+Z vs FADD-TRADD-DKO TRAIL+C+Z+N	-65	***
CON TRAIL+C+Z vs FADD-RIPK1-DKO TRAIL	-71	***
CON TRAIL+C+Z vs FADD-RIPK1-DKO TRAIL+Z	-70	***
CON TRAIL+C+Z vs FADD-RIPK1-DKO TRAIL+N	-64	***
CON TRAIL+C+Z vs FADD-RIPK1-DKO TRAIL+Z+N	-68	***
CON TRAIL+C+Z vs FADD-RIPK1-DKO TRAIL+C	-59	***
CON TRAIL+C+Z vs FADD-RIPK1-DKO TRAIL+C+Z	-60	***
CON TRAIL+C+Z vs FADD-RIPK1-DKO TRAIL+C+N	-59	***
CON TRAIL+C+Z vs FADD-RIPK1-DKO TRAIL+C+Z+N	-67	***
CON TRAIL+C+Z vs TRADD-RIPK1-DKO TRAIL	-49	***
CON TRAIL+C+Z vs TRADD-RIPK1-DKO TRAIL+Z	-64	***
CON TRAIL+C+Z vs TRADD-RIPK1-DKO TRAIL+N	-40	***
CON TRAIL+C+Z vs TRADD-RIPK1-DKO TRAIL+Z+N	-70	***
CON TRAIL+C+Z vs TRADD-RIPK1-DKO TRAIL+C	16	ns
CON TRAIL+C+Z vs TRADD-RIPK1-DKO TRAIL+C+Z	-70	***
CON TRAIL+C+Z vs TRADD-RIPK1-DKO TRAIL+C+N	17	ns
CON TRAIL+C+Z vs TRADD-RIPK1-DKO TRAIL+C+Z+N	-69	***
CON TRAIL+C+Z vs Casp.8-KO TRAIL	-58	***
CON TRAIL+C+Z vs Casp.8-KO TRAIL+Z	-49	***
CON TRAIL+C+Z vs Casp.8-KO TRAIL+N	-71	***
CON TRAIL+C+Z vs Casp.8-KO TRAIL+Z+N	-72	***
CON TRAIL+C+Z vs Casp.8-KO TRAIL+C	-37	***
CON TRAIL+C+Z vs Casp.8-KO TRAIL+C+Z	-34	***

CON TRAIL+C+Z vs Casp.8-KO TRAIL+C+N	-64	***
CON TRAIL+C+Z vs Casp.8-KO TRAIL+C+Z+N	-69	***
CON TRAIL+C+Z vs EV TRAIL	-69	***
CON TRAIL+C+Z vs EV TRAIL+Z	-70	***
CON TRAIL+C+Z vs EV TRAIL+N	-53	***
CON TRAIL+C+Z vs EV TRAIL+Z+N	-65	***
CON TRAIL+C+Z vs EV TRAIL+C	7,3	ns
CON TRAIL+C+Z vs EV TRAIL+C+Z	-68	***
CON TRAIL+C+Z vs EV TRAIL+C+N	11	ns
CON TRAIL+C+Z vs EV TRAIL+C+Z+N	-65	***
CON TRAIL+C+N vs CON TRAIL+C+Z+N	-92	***
CON TRAIL+C+N vs FADD-KO TRAIL	-91	***
CON TRAIL+C+N vs FADD-KO TRAIL+Z	-85	***
CON TRAIL+C+N vs FADD-KO TRAIL+N	-89	***
CON TRAIL+C+N vs FADD-KO TRAIL+Z+N	-91	***
CON TRAIL+C+N vs FADD-KO TRAIL+C	-81	***
CON TRAIL+C+N vs FADD-KO TRAIL+C+Z	-83	***
CON TRAIL+C+N vs FADD-KO TRAIL+C+N	-83	***
CON TRAIL+C+N vs FADD-KO TRAIL+C+Z+N	-86	***
CON TRAIL+C+N vs TRADD-KO TRAIL	-87	***
CON TRAIL+C+N vs TRADD-KO TRAIL+Z	-77	***
CON TRAIL+C+N vs TRADD-KO TRAIL+N	-73	***
CON TRAIL+C+N vs TRADD-KO TRAIL+Z+N	-94	***
CON TRAIL+C+N vs TRADD-KO TRAIL+C	-2,1	ns
CON TRAIL+C+N vs TRADD-KO TRAIL+C+Z	-5,0	ns
CON TRAIL+C+N vs TRADD-KO TRAIL+C+N	-8,8	ns
CON TRAIL+C+N vs TRADD-KO TRAIL+C+Z+N	-90	***
CON TRAIL+C+N vs RIPK1-KO TRAIL	-80	***
CON TRAIL+C+N vs RIPK1-KO TRAIL+Z	-90	***
CON TRAIL+C+N vs RIPK1-KO TRAIL+N	-71	***
CON TRAIL+C+N vs RIPK1-KO TRAIL+Z+N	-88	***
CON TRAIL+C+N vs RIPK1-KO TRAIL+C	-3,2	ns
CON TRAIL+C+N vs RIPK1-KO TRAIL+C+Z	-89	***
CON TRAIL+C+N vs RIPK1-KO TRAIL+C+N	-5,0	ns
CON TRAIL+C+N vs RIPK1-KO TRAIL+C+Z+N	-89	***
CON TRAIL+C+N vs FADD-TRADD-DKO TRAIL	-75	***
CON TRAIL+C+N vs FADD-TRADD-DKO TRAIL+Z	-70	***
CON TRAIL+C+N vs FADD-TRADD-DKO TRAIL+N	-71	***
CON TRAIL+C+N vs FADD-TRADD-DKO TRAIL+Z+N	-89	***
CON TRAIL+C+N vs FADD-TRADD-DKO TRAIL+C	-65	***
CON TRAIL+C+N vs FADD-TRADD-DKO TRAIL+C+Z	-89	***
CON TRAIL+C+N vs FADD-TRADD-DKO TRAIL+C+N	-69	***
CON TRAIL+C+N vs FADD-TRADD-DKO TRAIL+C+Z+N	-87	***
CON TRAIL+C+N vs FADD-RIPK1-DKO TRAIL	-93	***
CON TRAIL+C+N vs FADD-RIPK1-DKO TRAIL+Z	-91	***
CON TRAIL+C+N vs FADD-RIPK1-DKO TRAIL+N	-86	***
CON TRAIL+C+N vs FADD-RIPK1-DKO TRAIL+Z+N	-89	***
CON TRAIL+C+N vs FADD-RIPK1-DKO TRAIL+C	-81	***
CON TRAIL+C+N vs FADD-RIPK1-DKO TRAIL+C+Z	-82	***
CON TRAIL+C+N vs FADD-RIPK1-DKO TRAIL+C+N	-81	***
CON TRAIL+C+N vs FADD-RIPK1-DKO TRAIL+C+Z+N	-89	***
CON TRAIL+C+N vs TRADD-RIPK1-DKO TRAIL	-71	***
CON TRAIL+C+N vs TRADD-RIPK1-DKO TRAIL+Z	-86	***
CON TRAIL+C+N vs TRADD-RIPK1-DKO TRAIL+N	-62	***

CON TRAIL+C+N vs TRADD-RIPK1-DKO TRAIL+Z+N	-92	***
CON TRAIL+C+N vs TRADD-RIPK1-DKO TRAIL+C	-6,1	ns
CON TRAIL+C+N vs TRADD-RIPK1-DKO TRAIL+C+Z	-92	***
CON TRAIL+C+N vs TRADD-RIPK1-DKO TRAIL+C+N	-5,3	ns
CON TRAIL+C+N vs TRADD-RIPK1-DKO TRAIL+C+Z+N	-91	***
CON TRAIL+C+N vs Casp.8-KO TRAIL	-80	***
CON TRAIL+C+N vs Casp.8-KO TRAIL+Z	-71	***
CON TRAIL+C+N vs Casp.8-KO TRAIL+N	-92	***
CON TRAIL+C+N vs Casp.8-KO TRAIL+Z+N	-94	***
CON TRAIL+C+N vs Casp.8-KO TRAIL+C	-59	***
CON TRAIL+C+N vs Casp.8-KO TRAIL+C+Z	-56	***
CON TRAIL+C+N vs Casp.8-KO TRAIL+C+N	-85	***
CON TRAIL+C+N vs Casp.8-KO TRAIL+C+Z+N	-90	***
CON TRAIL+C+N vs EV TRAIL	-91	***
CON TRAIL+C+N vs EV TRAIL+Z	-92	***
CON TRAIL+C+N vs EV TRAIL+N	-75	***
CON TRAIL+C+N vs EV TRAIL+Z+N	-87	***
CON TRAIL+C+N vs EV TRAIL+C	-15	ns
CON TRAIL+C+N vs EV TRAIL+C+Z	-90	***
CON TRAIL+C+N vs EV TRAIL+C+N	-11	ns
CON TRAIL+C+N vs EV TRAIL+C+Z+N	-87	***
CON TRAIL+C+Z+N vs FADD-KO TRAIL	1,3	ns
CON TRAIL+C+Z+N vs FADD-KO TRAIL+Z	6,6	ns
CON TRAIL+C+Z+N vs FADD-KO TRAIL+N	3,2	ns
CON TRAIL+C+Z+N vs FADD-KO TRAIL+Z+N	0,89	ns
CON TRAIL+C+Z+N vs FADD-KO TRAIL+C	11	ns
CON TRAIL+C+Z+N vs FADD-KO TRAIL+C+Z	8,7	ns
CON TRAIL+C+Z+N vs FADD-KO TRAIL+C+N	9,1	ns
CON TRAIL+C+Z+N vs FADD-KO TRAIL+C+Z+N	5,9	ns
CON TRAIL+C+Z+N vs TRADD-KO TRAIL	5,2	ns
CON TRAIL+C+Z+N vs TRADD-KO TRAIL+Z	15	ns
CON TRAIL+C+Z+N vs TRADD-KO TRAIL+N	19	**
CON TRAIL+C+Z+N vs TRADD-KO TRAIL+Z+N	-2,1	ns
CON TRAIL+C+Z+N vs TRADD-KO TRAIL+C	90	***
CON TRAIL+C+Z+N vs TRADD-KO TRAIL+C+Z	87	***
CON TRAIL+C+Z+N vs TRADD-KO TRAIL+C+N	83	***
CON TRAIL+C+Z+N vs TRADD-KO TRAIL+C+Z+N	1,7	ns
CON TRAIL+C+Z+N vs RIPK1-KO TRAIL	12	ns
CON TRAIL+C+Z+N vs RIPK1-KO TRAIL+Z	1,7	ns
CON TRAIL+C+Z+N vs RIPK1-KO TRAIL+N	21	***
CON TRAIL+C+Z+N vs RIPK1-KO TRAIL+Z+N	4,3	ns
CON TRAIL+C+Z+N vs RIPK1-KO TRAIL+C	89	***
CON TRAIL+C+Z+N vs RIPK1-KO TRAIL+C+Z	3,1	ns
CON TRAIL+C+Z+N vs RIPK1-KO TRAIL+C+N	87	***
CON TRAIL+C+Z+N vs RIPK1-KO TRAIL+C+Z+N	3,2	ns
CON TRAIL+C+Z+N vs FADD-TRADD-DKO TRAIL	17	*
CON TRAIL+C+Z+N vs FADD-TRADD-DKO TRAIL+Z	22	***
CON TRAIL+C+Z+N vs FADD-TRADD-DKO TRAIL+N	21	***
CON TRAIL+C+Z+N vs FADD-TRADD-DKO TRAIL+Z+N	3,3	ns
CON TRAIL+C+Z+N vs FADD-TRADD-DKO TRAIL+C	27	***
CON TRAIL+C+Z+N vs FADD-TRADD-DKO TRAIL+C+Z	3,3	ns
CON TRAIL+C+Z+N vs FADD-TRADD-DKO TRAIL+C+N	23	***
CON TRAIL+C+Z+N vs FADD-TRADD-DKO TRAIL+C+Z+N	4,8	ns
CON TRAIL+C+Z+N vs FADD-RIPK1-DKO TRAIL	-1,0	ns

CON TRAIL+C+Z+N vs FADD-RIPK1-DKO TRAIL+Z	0,56	ns
CON TRAIL+C+Z+N vs FADD-RIPK1-DKO TRAIL+N	5,7	ns
CON TRAIL+C+Z+N vs FADD-RIPK1-DKO TRAIL+Z+N	2,5	ns
CON TRAIL+C+Z+N vs FADD-RIPK1-DKO TRAIL+C	11	ns
CON TRAIL+C+Z+N vs FADD-RIPK1-DKO TRAIL+C+Z	10	ns
CON TRAIL+C+Z+N vs FADD-RIPK1-DKO TRAIL+C+N	11	ns
CON TRAIL+C+Z+N vs FADD-RIPK1-DKO TRAIL+C+Z+N	3,4	ns
CON TRAIL+C+Z+N vs TRADD-RIPK1-DKO TRAIL	21	***
CON TRAIL+C+Z+N vs TRADD-RIPK1-DKO TRAIL+Z	6,2	ns
CON TRAIL+C+Z+N vs TRADD-RIPK1-DKO TRAIL+N	30	***
CON TRAIL+C+Z+N vs TRADD-RIPK1-DKO TRAIL+Z+N	-0,082	ns
CON TRAIL+C+Z+N vs TRADD-RIPK1-DKO TRAIL+C	86	***
CON TRAIL+C+Z+N vs TRADD-RIPK1-DKO TRAIL+C+Z	-0,0074	ns
CON TRAIL+C+Z+N vs TRADD-RIPK1-DKO TRAIL+C+N	87	***
CON TRAIL+C+Z+N vs TRADD-RIPK1-DKO TRAIL+C+Z+N	1,0	ns
CON TRAIL+C+Z+N vs Casp.8-KO TRAIL	12	ns
CON TRAIL+C+Z+N vs Casp.8-KO TRAIL+Z	21	***
CON TRAIL+C+Z+N vs Casp.8-KO TRAIL+N	-0,48	ns
CON TRAIL+C+Z+N vs Casp.8-KO TRAIL+Z+N	-2,0	ns
CON TRAIL+C+Z+N vs Casp.8-KO TRAIL+C	33	***
CON TRAIL+C+Z+N vs Casp.8-KO TRAIL+C+Z	36	***
CON TRAIL+C+Z+N vs Casp.8-KO TRAIL+C+N	6,6	ns
CON TRAIL+C+Z+N vs Casp.8-KO TRAIL+C+Z+N	1,5	ns
CON TRAIL+C+Z+N vs EV TRAIL	1,2	ns
CON TRAIL+C+Z+N vs EV TRAIL+Z	0,12	ns
CON TRAIL+C+Z+N vs EV TRAIL+N	17	ns
CON TRAIL+C+Z+N vs EV TRAIL+Z+N	5,1	ns
CON TRAIL+C+Z+N vs EV TRAIL+C	77	***
CON TRAIL+C+Z+N vs EV TRAIL+C+Z	1,7	ns
CON TRAIL+C+Z+N vs EV TRAIL+C+N	81	***
CON TRAIL+C+Z+N vs EV TRAIL+C+Z+N	4,9	ns
FADD-KO TRAIL vs FADD-KO TRAIL+Z	5,3	ns
FADD-KO TRAIL vs FADD-KO TRAIL+N	1,9	ns
FADD-KO TRAIL vs FADD-KO TRAIL+Z+N	-0,42	ns
FADD-KO TRAIL vs FADD-KO TRAIL+C	9,2	ns
FADD-KO TRAIL vs FADD-KO TRAIL+C+Z	7,4	ns
FADD-KO TRAIL vs FADD-KO TRAIL+C+N	7,8	ns
FADD-KO TRAIL vs FADD-KO TRAIL+C+Z+N	4,6	ns
FADD-KO TRAIL vs TRADD-KO TRAIL	3,9	ns
FADD-KO TRAIL vs TRADD-KO TRAIL+Z	13	ns
FADD-KO TRAIL vs TRADD-KO TRAIL+N	18	**
FADD-KO TRAIL vs TRADD-KO TRAIL+Z+N	-3,4	ns
FADD-KO TRAIL vs TRADD-KO TRAIL+C	88	***
FADD-KO TRAIL vs TRADD-KO TRAIL+C+Z	86	***
FADD-KO TRAIL vs TRADD-KO TRAIL+C+N	82	***
FADD-KO TRAIL vs TRADD-KO TRAIL+C+Z+N	0,36	ns
FADD-KO TRAIL vs RIPK1-KO TRAIL	10	ns
FADD-KO TRAIL vs RIPK1-KO TRAIL+Z	0,34	ns
FADD-KO TRAIL vs RIPK1-KO TRAIL+N	20	***
FADD-KO TRAIL vs RIPK1-KO TRAIL+Z+N	3,0	ns
FADD-KO TRAIL vs RIPK1-KO TRAIL+C	87	***
FADD-KO TRAIL vs RIPK1-KO TRAIL+C+Z	1,8	ns
FADD-KO TRAIL vs RIPK1-KO TRAIL+C+N	86	***
FADD-KO TRAIL vs RIPK1-KO TRAIL+C+Z+N	1,9	ns

FADD-KO TRAIL vs FADD-TRADD-DKO TRAIL	16	*
FADD-KO TRAIL vs FADD-TRADD-DKO TRAIL+Z	20	***
FADD-KO TRAIL vs FADD-TRADD-DKO TRAIL+N	20	***
FADD-KO TRAIL vs FADD-TRADD-DKO TRAIL+Z+N	1,9	ns
FADD-KO TRAIL vs FADD-TRADD-DKO TRAIL+C	25	***
FADD-KO TRAIL vs FADD-TRADD-DKO TRAIL+C+Z	2,0	ns
FADD-KO TRAIL vs FADD-TRADD-DKO TRAIL+C+N	22	***
FADD-KO TRAIL vs FADD-TRADD-DKO TRAIL+C+Z+N	3,5	ns
FADD-KO TRAIL vs FADD-RIPK1-DKO TRAIL	-2,4	ns
FADD-KO TRAIL vs FADD-RIPK1-DKO TRAIL+Z	-0,76	ns
FADD-KO TRAIL vs FADD-RIPK1-DKO TRAIL+N	4,4	ns
FADD-KO TRAIL vs FADD-RIPK1-DKO TRAIL+Z+N	1,2	ns
FADD-KO TRAIL vs FADD-RIPK1-DKO TRAIL+C	9,7	ns
FADD-KO TRAIL vs FADD-RIPK1-DKO TRAIL+C+Z	9,1	ns
FADD-KO TRAIL vs FADD-RIPK1-DKO TRAIL+C+N	9,4	ns
FADD-KO TRAIL vs FADD-RIPK1-DKO TRAIL+C+Z+N	2,1	ns
FADD-KO TRAIL vs TRADD-RIPK1-DKO TRAIL	20	***
FADD-KO TRAIL vs TRADD-RIPK1-DKO TRAIL+Z	4,9	ns
FADD-KO TRAIL vs TRADD-RIPK1-DKO TRAIL+N	29	***
FADD-KO TRAIL vs TRADD-RIPK1-DKO TRAIL+Z+N	-1,4	ns
FADD-KO TRAIL vs TRADD-RIPK1-DKO TRAIL+C	85	***
FADD-KO TRAIL vs TRADD-RIPK1-DKO TRAIL+C+Z	-1,3	ns
FADD-KO TRAIL vs TRADD-RIPK1-DKO TRAIL+C+N	85	***
FADD-KO TRAIL vs TRADD-RIPK1-DKO TRAIL+C+Z+N	-0,28	ns
FADD-KO TRAIL vs Casp.8-KO TRAIL	11	ns
FADD-KO TRAIL vs Casp.8-KO TRAIL+Z	20	***
FADD-KO TRAIL vs Casp.8-KO TRAIL+N	-1,8	ns
FADD-KO TRAIL vs Casp.8-KO TRAIL+Z+N	-3,3	ns
FADD-KO TRAIL vs Casp.8-KO TRAIL+C	32	***
FADD-KO TRAIL vs Casp.8-KO TRAIL+C+Z	35	***
FADD-KO TRAIL vs Casp.8-KO TRAIL+C+N	5,3	ns
FADD-KO TRAIL vs Casp.8-KO TRAIL+C+Z+N	0,21	ns
FADD-KO TRAIL vs EV TRAIL	-0,069	ns
FADD-KO TRAIL vs EV TRAIL+Z	-1,2	ns
FADD-KO TRAIL vs EV TRAIL+N	16	ns
FADD-KO TRAIL vs EV TRAIL+Z+N	3,8	ns
FADD-KO TRAIL vs EV TRAIL+C	76	***
FADD-KO TRAIL vs EV TRAIL+C+Z	0,38	ns
FADD-KO TRAIL vs EV TRAIL+C+N	79	***
FADD-KO TRAIL vs EV TRAIL+C+Z+N	3,6	ns
FADD-KO TRAIL+Z vs FADD-KO TRAIL+N	-3,4	ns
FADD-KO TRAIL+Z vs FADD-KO TRAIL+Z+N	-5,7	ns
FADD-KO TRAIL+Z vs FADD-KO TRAIL+C	3,9	ns
FADD-KO TRAIL+Z vs FADD-KO TRAIL+C+Z	2,0	ns
FADD-KO TRAIL+Z vs FADD-KO TRAIL+C+N	2,5	ns
FADD-KO TRAIL+Z vs FADD-KO TRAIL+C+Z+N	-0,74	ns
FADD-KO TRAIL+Z vs TRADD-KO TRAIL	-1,4	ns
FADD-KO TRAIL+Z vs TRADD-KO TRAIL+Z	8,1	ns
FADD-KO TRAIL+Z vs TRADD-KO TRAIL+N	12	ns
FADD-KO TRAIL+Z vs TRADD-KO TRAIL+Z+N	-8,8	ns
FADD-KO TRAIL+Z vs TRADD-KO TRAIL+C	83	***
FADD-KO TRAIL+Z vs TRADD-KO TRAIL+C+Z	80	***
FADD-KO TRAIL+Z vs TRADD-KO TRAIL+C+N	77	***
FADD-KO TRAIL+Z vs TRADD-KO TRAIL+C+Z+N	-4,9	ns

FADD-KO TRAIL+Z vs RIPK1-KO TRAIL	4,9	ns
FADD-KO TRAIL+Z vs RIPK1-KO TRAIL+Z	-5,0	ns
FADD-KO TRAIL+Z vs RIPK1-KO TRAIL+N	14	*
FADD-KO TRAIL+Z vs RIPK1-KO TRAIL+Z+N	-2,3	ns
FADD-KO TRAIL+Z vs RIPK1-KO TRAIL+C	82	***
FADD-KO TRAIL+Z vs RIPK1-KO TRAIL+C+Z	-3,5	ns
FADD-KO TRAIL+Z vs RIPK1-KO TRAIL+C+N	80	***
FADD-KO TRAIL+Z vs RIPK1-KO TRAIL+C+Z+N	-3,4	ns
FADD-KO TRAIL+Z vs FADD-TRADD-DKO TRAIL	11	ns
FADD-KO TRAIL+Z vs FADD-TRADD-DKO TRAIL+Z	15	*
FADD-KO TRAIL+Z vs FADD-TRADD-DKO TRAIL+N	15	ns
FADD-KO TRAIL+Z vs FADD-TRADD-DKO TRAIL+Z+N	-3,4	ns
FADD-KO TRAIL+Z vs FADD-TRADD-DKO TRAIL+C	20	***
FADD-KO TRAIL+Z vs FADD-TRADD-DKO TRAIL+C+Z	-3,3	ns
FADD-KO TRAIL+Z vs FADD-TRADD-DKO TRAIL+C+N	16	ns
FADD-KO TRAIL+Z vs FADD-TRADD-DKO TRAIL+C+Z+N	-1,8	ns
FADD-KO TRAIL+Z vs FADD-RIPK1-DKO TRAIL	-7,7	ns
FADD-KO TRAIL+Z vs FADD-RIPK1-DKO TRAIL+Z	-6,1	ns
FADD-KO TRAIL+Z vs FADD-RIPK1-DKO TRAIL+N	-0,88	ns
FADD-KO TRAIL+Z vs FADD-RIPK1-DKO TRAIL+Z+N	-4,1	ns
FADD-KO TRAIL+Z vs FADD-RIPK1-DKO TRAIL+C	4,4	ns
FADD-KO TRAIL+Z vs FADD-RIPK1-DKO TRAIL+C+Z	3,8	ns
FADD-KO TRAIL+Z vs FADD-RIPK1-DKO TRAIL+C+N	4,1	ns
FADD-KO TRAIL+Z vs FADD-RIPK1-DKO TRAIL+C+Z+N	-3,2	ns
FADD-KO TRAIL+Z vs TRADD-RIPK1-DKO TRAIL	15	*
FADD-KO TRAIL+Z vs TRADD-RIPK1-DKO TRAIL+Z	-0,42	ns
FADD-KO TRAIL+Z vs TRADD-RIPK1-DKO TRAIL+N	24	***
FADD-KO TRAIL+Z vs TRADD-RIPK1-DKO TRAIL+Z+N	-6,7	ns
FADD-KO TRAIL+Z vs TRADD-RIPK1-DKO TRAIL+C	79	***
FADD-KO TRAIL+Z vs TRADD-RIPK1-DKO TRAIL+C+Z	-6,6	ns
FADD-KO TRAIL+Z vs TRADD-RIPK1-DKO TRAIL+C+N	80	***
FADD-KO TRAIL+Z vs TRADD-RIPK1-DKO TRAIL+C+Z+N	-5,6	ns
FADD-KO TRAIL+Z vs Casp.8-KO TRAIL	5,6	ns
FADD-KO TRAIL+Z vs Casp.8-KO TRAIL+Z	15	*
FADD-KO TRAIL+Z vs Casp.8-KO TRAIL+N	-7,1	ns
FADD-KO TRAIL+Z vs Casp.8-KO TRAIL+Z+N	-8,6	ns
FADD-KO TRAIL+Z vs Casp.8-KO TRAIL+C	27	***
FADD-KO TRAIL+Z vs Casp.8-KO TRAIL+C+Z	29	***
FADD-KO TRAIL+Z vs Casp.8-KO TRAIL+C+N	-0,038	ns
FADD-KO TRAIL+Z vs Casp.8-KO TRAIL+C+Z+N	-5,1	ns
FADD-KO TRAIL+Z vs EV TRAIL	-5,4	ns
FADD-KO TRAIL+Z vs EV TRAIL+Z	-6,5	ns
FADD-KO TRAIL+Z vs EV TRAIL+N	11	ns
FADD-KO TRAIL+Z vs EV TRAIL+Z+N	-1,5	ns
FADD-KO TRAIL+Z vs EV TRAIL+C	71	***
FADD-KO TRAIL+Z vs EV TRAIL+C+Z	-4,9	ns
FADD-KO TRAIL+Z vs EV TRAIL+C+N	74	***
FADD-KO TRAIL+Z vs EV TRAIL+C+Z+N	-1,8	ns
FADD-KO TRAIL+N vs FADD-KO TRAIL+Z+N	-2,3	ns
FADD-KO TRAIL+N vs FADD-KO TRAIL+C	7,3	ns
FADD-KO TRAIL+N vs FADD-KO TRAIL+C+Z	5,5	ns
FADD-KO TRAIL+N vs FADD-KO TRAIL+C+N	5,9	ns
FADD-KO TRAIL+N vs FADD-KO TRAIL+C+Z+N	2,7	ns
FADD-KO TRAIL+N vs TRADD-KO TRAIL	2,0	ns

FADD-KO TRAIL+N vs TRADD-KO TRAIL+Z	12	ns
FADD-KO TRAIL+N vs TRADD-KO TRAIL+N	16	ns
FADD-KO TRAIL+N vs TRADD-KO TRAIL+Z+N	-5,3	ns
FADD-KO TRAIL+N vs TRADD-KO TRAIL+C	87	***
FADD-KO TRAIL+N vs TRADD-KO TRAIL+C+Z	84	***
FADD-KO TRAIL+N vs TRADD-KO TRAIL+C+N	80	***
FADD-KO TRAIL+N vs TRADD-KO TRAIL+C+Z+N	-1,5	ns
FADD-KO TRAIL+N vs RIPK1-KO TRAIL	8,4	ns
FADD-KO TRAIL+N vs RIPK1-KO TRAIL+Z	-1,5	ns
FADD-KO TRAIL+N vs RIPK1-KO TRAIL+N	18	***
FADD-KO TRAIL+N vs RIPK1-KO TRAIL+Z+N	1,1	ns
FADD-KO TRAIL+N vs RIPK1-KO TRAIL+C	85	***
FADD-KO TRAIL+N vs RIPK1-KO TRAIL+C+Z	-0,077	ns
FADD-KO TRAIL+N vs RIPK1-KO TRAIL+C+N	84	***
FADD-KO TRAIL+N vs RIPK1-KO TRAIL+C+Z+N	0,014	ns
FADD-KO TRAIL+N vs FADD-TRADD-DKO TRAIL	14	ns
FADD-KO TRAIL+N vs FADD-TRADD-DKO TRAIL+Z	18	***
FADD-KO TRAIL+N vs FADD-TRADD-DKO TRAIL+N	18	**
FADD-KO TRAIL+N vs FADD-TRADD-DKO TRAIL+Z+N	0,056	ns
FADD-KO TRAIL+N vs FADD-TRADD-DKO TRAIL+C	23	***
FADD-KO TRAIL+N vs FADD-TRADD-DKO TRAIL+C+Z	0,12	ns
FADD-KO TRAIL+N vs FADD-TRADD-DKO TRAIL+C+N	20	**
FADD-KO TRAIL+N vs FADD-TRADD-DKO TRAIL+C+Z+N	1,6	ns
FADD-KO TRAIL+N vs FADD-RIPK1-DKO TRAIL	-4,2	ns
FADD-KO TRAIL+N vs FADD-RIPK1-DKO TRAIL+Z	-2,6	ns
FADD-KO TRAIL+N vs FADD-RIPK1-DKO TRAIL+N	2,5	ns
FADD-KO TRAIL+N vs FADD-RIPK1-DKO TRAIL+Z+N	-0,71	ns
FADD-KO TRAIL+N vs FADD-RIPK1-DKO TRAIL+C	7,8	ns
FADD-KO TRAIL+N vs FADD-RIPK1-DKO TRAIL+C+Z	7,2	ns
FADD-KO TRAIL+N vs FADD-RIPK1-DKO TRAIL+C+N	7,5	ns
FADD-KO TRAIL+N vs FADD-RIPK1-DKO TRAIL+C+Z+N	0,22	ns
FADD-KO TRAIL+N vs TRADD-RIPK1-DKO TRAIL	18	***
FADD-KO TRAIL+N vs TRADD-RIPK1-DKO TRAIL+Z	3,0	ns
FADD-KO TRAIL+N vs TRADD-RIPK1-DKO TRAIL+N	27	***
FADD-KO TRAIL+N vs TRADD-RIPK1-DKO TRAIL+Z+N	-3,3	ns
FADD-KO TRAIL+N vs TRADD-RIPK1-DKO TRAIL+C	83	***
FADD-KO TRAIL+N vs TRADD-RIPK1-DKO TRAIL+C+Z	-3,2	ns
FADD-KO TRAIL+N vs TRADD-RIPK1-DKO TRAIL+C+N	83	***
FADD-KO TRAIL+N vs TRADD-RIPK1-DKO TRAIL+C+Z+N	-2,2	ns
FADD-KO TRAIL+N vs Casp.8-KO TRAIL	9,0	ns
FADD-KO TRAIL+N vs Casp.8-KO TRAIL+Z	18	***
FADD-KO TRAIL+N vs Casp.8-KO TRAIL+N	-3,7	ns
FADD-KO TRAIL+N vs Casp.8-KO TRAIL+Z+N	-5,2	ns
FADD-KO TRAIL+N vs Casp.8-KO TRAIL+C	30	***
FADD-KO TRAIL+N vs Casp.8-KO TRAIL+C+Z	33	***
FADD-KO TRAIL+N vs Casp.8-KO TRAIL+C+N	3,4	ns
FADD-KO TRAIL+N vs Casp.8-KO TRAIL+C+Z+N	-1,7	ns
FADD-KO TRAIL+N vs EV TRAIL	-2,0	ns
FADD-KO TRAIL+N vs EV TRAIL+Z	-3,1	ns
FADD-KO TRAIL+N vs EV TRAIL+N	14	ns
FADD-KO TRAIL+N vs EV TRAIL+Z+N	1,9	ns
FADD-KO TRAIL+N vs EV TRAIL+C	74	***
FADD-KO TRAIL+N vs EV TRAIL+C+Z	-1,5	ns
FADD-KO TRAIL+N vs EV TRAIL+C+N	77	***

FADD-KO TRAIL+N vs EV TRAIL+C+Z+N	1,7	ns
FADD-KO TRAIL+Z+N vs FADD-KO TRAIL+C	9,6	ns
FADD-KO TRAIL+Z+N vs FADD-KO TRAIL+C+Z	7,8	ns
FADD-KO TRAIL+Z+N vs FADD-KO TRAIL+C+N	8,2	ns
FADD-KO TRAIL+Z+N vs FADD-KO TRAIL+C+Z+N	5,0	ns
FADD-KO TRAIL+Z+N vs TRADD-KO TRAIL	4,3	ns
FADD-KO TRAIL+Z+N vs TRADD-KO TRAIL+Z	14	ns
FADD-KO TRAIL+Z+N vs TRADD-KO TRAIL+N	18	*
FADD-KO TRAIL+Z+N vs TRADD-KO TRAIL+Z+N	-3,0	ns
FADD-KO TRAIL+Z+N vs TRADD-KO TRAIL+C	89	***
FADD-KO TRAIL+Z+N vs TRADD-KO TRAIL+C+Z	86	***
FADD-KO TRAIL+Z+N vs TRADD-KO TRAIL+C+N	82	***
FADD-KO TRAIL+Z+N vs TRADD-KO TRAIL+C+Z+N	0,78	ns
FADD-KO TRAIL+Z+N vs RIPK1-KO TRAIL	11	ns
FADD-KO TRAIL+Z+N vs RIPK1-KO TRAIL+Z	0,76	ns
FADD-KO TRAIL+Z+N vs RIPK1-KO TRAIL+N	20	***
FADD-KO TRAIL+Z+N vs RIPK1-KO TRAIL+Z+N	3,4	ns
FADD-KO TRAIL+Z+N vs RIPK1-KO TRAIL+C	88	***
FADD-KO TRAIL+Z+N vs RIPK1-KO TRAIL+C+Z	2,2	ns
FADD-KO TRAIL+Z+N vs RIPK1-KO TRAIL+C+N	86	***
FADD-KO TRAIL+Z+N vs RIPK1-KO TRAIL+C+Z+N	2,3	ns
FADD-KO TRAIL+Z+N vs FADD-TRADD-DKO TRAIL	17	*
FADD-KO TRAIL+Z+N vs FADD-TRADD-DKO TRAIL+Z	21	***
FADD-KO TRAIL+Z+N vs FADD-TRADD-DKO TRAIL+N	20	***
FADD-KO TRAIL+Z+N vs FADD-TRADD-DKO TRAIL+Z+N	2,4	ns
FADD-KO TRAIL+Z+N vs FADD-TRADD-DKO TRAIL+C	26	***
FADD-KO TRAIL+Z+N vs FADD-TRADD-DKO TRAIL+C+Z	2,4	ns
FADD-KO TRAIL+Z+N vs FADD-TRADD-DKO TRAIL+C+N	22	***
FADD-KO TRAIL+Z+N vs FADD-TRADD-DKO TRAIL+C+Z+N	3,9	ns
FADD-KO TRAIL+Z+N vs FADD-RIPK1-DKO TRAIL	-1,9	ns
FADD-KO TRAIL+Z+N vs FADD-RIPK1-DKO TRAIL+Z	-0,34	ns
FADD-KO TRAIL+Z+N vs FADD-RIPK1-DKO TRAIL+N	4,9	ns
FADD-KO TRAIL+Z+N vs FADD-RIPK1-DKO TRAIL+Z+N	1,6	ns
FADD-KO TRAIL+Z+N vs FADD-RIPK1-DKO TRAIL+C	10	ns
FADD-KO TRAIL+Z+N vs FADD-RIPK1-DKO TRAIL+C+Z	9,5	ns
FADD-KO TRAIL+Z+N vs FADD-RIPK1-DKO TRAIL+C+N	9,8	ns
FADD-KO TRAIL+Z+N vs FADD-RIPK1-DKO TRAIL+C+Z+N	2,5	ns
FADD-KO TRAIL+Z+N vs TRADD-RIPK1-DKO TRAIL	20	***
FADD-KO TRAIL+Z+N vs TRADD-RIPK1-DKO TRAIL+Z	5,3	ns
FADD-KO TRAIL+Z+N vs TRADD-RIPK1-DKO TRAIL+N	29	***
FADD-KO TRAIL+Z+N vs TRADD-RIPK1-DKO TRAIL+Z+N	-0,97	ns
FADD-KO TRAIL+Z+N vs TRADD-RIPK1-DKO TRAIL+C	85	***
FADD-KO TRAIL+Z+N vs TRADD-RIPK1-DKO TRAIL+C+Z	-0,90	ns
FADD-KO TRAIL+Z+N vs TRADD-RIPK1-DKO TRAIL+C+N	86	***
FADD-KO TRAIL+Z+N vs TRADD-RIPK1-DKO TRAIL+C+Z+N	0,14	ns
FADD-KO TRAIL+Z+N vs Casp.8-KO TRAIL	11	ns
FADD-KO TRAIL+Z+N vs Casp.8-KO TRAIL+Z	20	***
FADD-KO TRAIL+Z+N vs Casp.8-KO TRAIL+N	-1,4	ns
FADD-KO TRAIL+Z+N vs Casp.8-KO TRAIL+Z+N	-2,9	ns
FADD-KO TRAIL+Z+N vs Casp.8-KO TRAIL+C	32	***
FADD-KO TRAIL+Z+N vs Casp.8-KO TRAIL+C+Z	35	***
FADD-KO TRAIL+Z+N vs Casp.8-KO TRAIL+C+N	5,7	ns
FADD-KO TRAIL+Z+N vs Casp.8-KO TRAIL+C+Z+N	0,63	ns
FADD-KO TRAIL+Z+N vs EV TRAIL	0,35	ns

FADD-KO TRAIL+Z+N vs EV TRAIL+Z	-0,77	ns
FADD-KO TRAIL+Z+N vs EV TRAIL+N	16	ns
FADD-KO TRAIL+Z+N vs EV TRAIL+Z+N	4,2	ns
FADD-KO TRAIL+Z+N vs EV TRAIL+C	77	***
FADD-KO TRAIL+Z+N vs EV TRAIL+C+Z	0,80	ns
FADD-KO TRAIL+Z+N vs EV TRAIL+C+N	80	***
FADD-KO TRAIL+Z+N vs EV TRAIL+C+Z+N	4,0	ns
FADD-KO TRAIL+C vs FADD-KO TRAIL+C+Z	-1,8	ns
FADD-KO TRAIL+C vs FADD-KO TRAIL+C+N	-1,4	ns
FADD-KO TRAIL+C vs FADD-KO TRAIL+C+Z+N	-4,6	ns
FADD-KO TRAIL+C vs TRADD-KO TRAIL	-5,3	ns
FADD-KO TRAIL+C vs TRADD-KO TRAIL+Z	4,2	ns
FADD-KO TRAIL+C vs TRADD-KO TRAIL+N	8,4	ns
FADD-KO TRAIL+C vs TRADD-KO TRAIL+Z+N	-13	ns
FADD-KO TRAIL+C vs TRADD-KO TRAIL+C	79	***
FADD-KO TRAIL+C vs TRADD-KO TRAIL+C+Z	76	***
FADD-KO TRAIL+C vs TRADD-KO TRAIL+C+N	73	***
FADD-KO TRAIL+C vs TRADD-KO TRAIL+C+Z+N	-8,8	ns
FADD-KO TRAIL+C vs RIPK1-KO TRAIL	1,0	ns
FADD-KO TRAIL+C vs RIPK1-KO TRAIL+Z	-8,9	ns
FADD-KO TRAIL+C vs RIPK1-KO TRAIL+N	11	ns
FADD-KO TRAIL+C vs RIPK1-KO TRAIL+Z+N	-6,2	ns
FADD-KO TRAIL+C vs RIPK1-KO TRAIL+C	78	***
FADD-KO TRAIL+C vs RIPK1-KO TRAIL+C+Z	-7,4	ns
FADD-KO TRAIL+C vs RIPK1-KO TRAIL+C+N	76	***
FADD-KO TRAIL+C vs RIPK1-KO TRAIL+C+Z+N	-7,3	ns
FADD-KO TRAIL+C vs FADD-TRADD-DKO TRAIL	6,9	ns
FADD-KO TRAIL+C vs FADD-TRADD-DKO TRAIL+Z	11	ns
FADD-KO TRAIL+C vs FADD-TRADD-DKO TRAIL+N	11	ns
FADD-KO TRAIL+C vs FADD-TRADD-DKO TRAIL+Z+N	-7,3	ns
FADD-KO TRAIL+C vs FADD-TRADD-DKO TRAIL+C	16	*
FADD-KO TRAIL+C vs FADD-TRADD-DKO TRAIL+C+Z	-7,2	ns
FADD-KO TRAIL+C vs FADD-TRADD-DKO TRAIL+C+N	12	ns
FADD-KO TRAIL+C vs FADD-TRADD-DKO TRAIL+C+Z+N	-5,7	ns
FADD-KO TRAIL+C vs FADD-RIPK1-DKO TRAIL	-12	ns
FADD-KO TRAIL+C vs FADD-RIPK1-DKO TRAIL+Z	-9,9	ns
FADD-KO TRAIL+C vs FADD-RIPK1-DKO TRAIL+N	-4,8	ns
FADD-KO TRAIL+C vs FADD-RIPK1-DKO TRAIL+Z+N	-8,0	ns
FADD-KO TRAIL+C vs FADD-RIPK1-DKO TRAIL+C	0,47	ns
FADD-KO TRAIL+C vs FADD-RIPK1-DKO TRAIL+C+Z	-0,11	ns
FADD-KO TRAIL+C vs FADD-RIPK1-DKO TRAIL+C+N	0,20	ns
FADD-KO TRAIL+C vs FADD-RIPK1-DKO TRAIL+C+Z+N	-7,1	ns
FADD-KO TRAIL+C vs TRADD-RIPK1-DKO TRAIL	11	ns
FADD-KO TRAIL+C vs TRADD-RIPK1-DKO TRAIL+Z	-4,3	ns
FADD-KO TRAIL+C vs TRADD-RIPK1-DKO TRAIL+N	20	***
FADD-KO TRAIL+C vs TRADD-RIPK1-DKO TRAIL+Z+N	-11	ns
FADD-KO TRAIL+C vs TRADD-RIPK1-DKO TRAIL+C	75	***
FADD-KO TRAIL+C vs TRADD-RIPK1-DKO TRAIL+C+Z	-11	ns
FADD-KO TRAIL+C vs TRADD-RIPK1-DKO TRAIL+C+N	76	***
FADD-KO TRAIL+C vs TRADD-RIPK1-DKO TRAIL+C+Z+N	-9,5	ns
FADD-KO TRAIL+C vs Casp.8-KO TRAIL	1,7	ns
FADD-KO TRAIL+C vs Casp.8-KO TRAIL+Z	11	ns
FADD-KO TRAIL+C vs Casp.8-KO TRAIL+N	-11	ns
FADD-KO TRAIL+C vs Casp.8-KO TRAIL+Z+N	-12	ns

FADD-KO TRAIL+C vs Casp.8-KO TRAIL+C	23	***
FADD-KO TRAIL+C vs Casp.8-KO TRAIL+C+Z	25	***
FADD-KO TRAIL+C vs Casp.8-KO TRAIL+C+N	-3,9	ns
FADD-KO TRAIL+C vs Casp.8-KO TRAIL+C+Z+N	-9,0	ns
FADD-KO TRAIL+C vs EV TRAIL	-9,3	ns
FADD-KO TRAIL+C vs EV TRAIL+Z	-10	ns
FADD-KO TRAIL+C vs EV TRAIL+N	6,6	ns
FADD-KO TRAIL+C vs EV TRAIL+Z+N	-5,4	ns
FADD-KO TRAIL+C vs EV TRAIL+C	67	***
FADD-KO TRAIL+C vs EV TRAIL+C+Z	-8,8	ns
FADD-KO TRAIL+C vs EV TRAIL+C+N	70	***
FADD-KO TRAIL+C vs EV TRAIL+C+Z+N	-5,6	ns
FADD-KO TRAIL+C+Z vs FADD-KO TRAIL+C+N	0,41	ns
FADD-KO TRAIL+C+Z vs FADD-KO TRAIL+C+Z+N	-2,8	ns
FADD-KO TRAIL+C+Z vs TRADD-KO TRAIL	-3,5	ns
FADD-KO TRAIL+C+Z vs TRADD-KO TRAIL+Z	6,1	ns
FADD-KO TRAIL+C+Z vs TRADD-KO TRAIL+N	10	ns
FADD-KO TRAIL+C+Z vs TRADD-KO TRAIL+Z+N	-11	ns
FADD-KO TRAIL+C+Z vs TRADD-KO TRAIL+C	81	***
FADD-KO TRAIL+C+Z vs TRADD-KO TRAIL+C+Z	78	***
FADD-KO TRAIL+C+Z vs TRADD-KO TRAIL+C+N	74	***
FADD-KO TRAIL+C+Z vs TRADD-KO TRAIL+C+Z+N	-7,0	ns
FADD-KO TRAIL+C+Z vs RIPK1-KO TRAIL	2,9	ns
FADD-KO TRAIL+C+Z vs RIPK1-KO TRAIL+Z	-7,0	ns
FADD-KO TRAIL+C+Z vs RIPK1-KO TRAIL+N	12	ns
FADD-KO TRAIL+C+Z vs RIPK1-KO TRAIL+Z+N	-4,4	ns
FADD-KO TRAIL+C+Z vs RIPK1-KO TRAIL+C	80	***
FADD-KO TRAIL+C+Z vs RIPK1-KO TRAIL+C+Z	-5,5	ns
FADD-KO TRAIL+C+Z vs RIPK1-KO TRAIL+C+N	78	***
FADD-KO TRAIL+C+Z vs RIPK1-KO TRAIL+C+Z+N	-5,5	ns
FADD-KO TRAIL+C+Z vs FADD-TRADD-DKO TRAIL	8,8	ns
FADD-KO TRAIL+C+Z vs FADD-TRADD-DKO TRAIL+Z	13	ns
FADD-KO TRAIL+C+Z vs FADD-TRADD-DKO TRAIL+N	13	ns
FADD-KO TRAIL+C+Z vs FADD-TRADD-DKO TRAIL+Z+N	-5,4	ns
FADD-KO TRAIL+C+Z vs FADD-TRADD-DKO TRAIL+C	18	*
FADD-KO TRAIL+C+Z vs FADD-TRADD-DKO TRAIL+C+Z	-5,3	ns
FADD-KO TRAIL+C+Z vs FADD-TRADD-DKO TRAIL+C+N	14	ns
FADD-KO TRAIL+C+Z vs FADD-TRADD-DKO TRAIL+C+Z+N	-3,8	ns
FADD-KO TRAIL+C+Z vs FADD-RIPK1-DKO TRAIL	-9,7	ns
FADD-KO TRAIL+C+Z vs FADD-RIPK1-DKO TRAIL+Z	-8,1	ns
FADD-KO TRAIL+C+Z vs FADD-RIPK1-DKO TRAIL+N	-2,9	ns
FADD-KO TRAIL+C+Z vs FADD-RIPK1-DKO TRAIL+Z+N	-6,2	ns
FADD-KO TRAIL+C+Z vs FADD-RIPK1-DKO TRAIL+C	2,3	ns
FADD-KO TRAIL+C+Z vs FADD-RIPK1-DKO TRAIL+C+Z	1,7	ns
FADD-KO TRAIL+C+Z vs FADD-RIPK1-DKO TRAIL+C+N	2,0	ns
FADD-KO TRAIL+C+Z vs FADD-RIPK1-DKO TRAIL+C+Z+N	-5,2	ns
FADD-KO TRAIL+C+Z vs TRADD-RIPK1-DKO TRAIL	13	ns
FADD-KO TRAIL+C+Z vs TRADD-RIPK1-DKO TRAIL+Z	-2,5	ns
FADD-KO TRAIL+C+Z vs TRADD-RIPK1-DKO TRAIL+N	21	***
FADD-KO TRAIL+C+Z vs TRADD-RIPK1-DKO TRAIL+Z+N	-8,7	ns
FADD-KO TRAIL+C+Z vs TRADD-RIPK1-DKO TRAIL+C	77	***
FADD-KO TRAIL+C+Z vs TRADD-RIPK1-DKO TRAIL+C+Z	-8,7	ns
FADD-KO TRAIL+C+Z vs TRADD-RIPK1-DKO TRAIL+C+N	78	***
FADD-KO TRAIL+C+Z vs TRADD-RIPK1-DKO TRAIL+C+Z+N	-7,6	ns

FADD-KO TRAIL+C+Z vs Casp.8-KO TRAIL	3,5	ns
FADD-KO TRAIL+C+Z vs Casp.8-KO TRAIL+Z	12	ns
FADD-KO TRAIL+C+Z vs Casp.8-KO TRAIL+N	-9,1	ns
FADD-KO TRAIL+C+Z vs Casp.8-KO TRAIL+Z+N	-11	ns
FADD-KO TRAIL+C+Z vs Casp.8-KO TRAIL+C	24	***
FADD-KO TRAIL+C+Z vs Casp.8-KO TRAIL+C+Z	27	***
FADD-KO TRAIL+C+Z vs Casp.8-KO TRAIL+C+N	-2,1	ns
FADD-KO TRAIL+C+Z vs Casp.8-KO TRAIL+C+Z+N	-7,1	ns
FADD-KO TRAIL+C+Z vs EV TRAIL	-7,4	ns
FADD-KO TRAIL+C+Z vs EV TRAIL+Z	-8,5	ns
FADD-KO TRAIL+C+Z vs EV TRAIL+N	8,5	ns
FADD-KO TRAIL+C+Z vs EV TRAIL+Z+N	-3,5	ns
FADD-KO TRAIL+C+Z vs EV TRAIL+C	69	***
FADD-KO TRAIL+C+Z vs EV TRAIL+C+Z	-7,0	ns
FADD-KO TRAIL+C+Z vs EV TRAIL+C+N	72	***
FADD-KO TRAIL+C+Z vs EV TRAIL+C+Z+N	-3,8	ns
FADD-KO TRAIL+C+N vs FADD-KO TRAIL+C+Z+N	-3,2	ns
FADD-KO TRAIL+C+N vs TRADD-KO TRAIL	-3,9	ns
FADD-KO TRAIL+C+N vs TRADD-KO TRAIL+Z	5,6	ns
FADD-KO TRAIL+C+N vs TRADD-KO TRAIL+N	9,8	ns
FADD-KO TRAIL+C+N vs TRADD-KO TRAIL+Z+N	-11	ns
FADD-KO TRAIL+C+N vs TRADD-KO TRAIL+C	81	***
FADD-KO TRAIL+C+N vs TRADD-KO TRAIL+C+Z	78	***
FADD-KO TRAIL+C+N vs TRADD-KO TRAIL+C+N	74	***
FADD-KO TRAIL+C+N vs TRADD-KO TRAIL+C+Z+N	-7,4	ns
FADD-KO TRAIL+C+N vs RIPK1-KO TRAIL	2,5	ns
FADD-KO TRAIL+C+N vs RIPK1-KO TRAIL+Z	-7,4	ns
FADD-KO TRAIL+C+N vs RIPK1-KO TRAIL+N	12	ns
FADD-KO TRAIL+C+N vs RIPK1-KO TRAIL+Z+N	-4,8	ns
FADD-KO TRAIL+C+N vs RIPK1-KO TRAIL+C	80	***
FADD-KO TRAIL+C+N vs RIPK1-KO TRAIL+C+Z	-6,0	ns
FADD-KO TRAIL+C+N vs RIPK1-KO TRAIL+C+N	78	***
FADD-KO TRAIL+C+N vs RIPK1-KO TRAIL+C+Z+N	-5,9	ns
FADD-KO TRAIL+C+N vs FADD-TRADD-DKO TRAIL	8,3	ns
FADD-KO TRAIL+C+N vs FADD-TRADD-DKO TRAIL+Z	13	ns
FADD-KO TRAIL+C+N vs FADD-TRADD-DKO TRAIL+N	12	ns
FADD-KO TRAIL+C+N vs FADD-TRADD-DKO TRAIL+Z+N	-5,8	ns
FADD-KO TRAIL+C+N vs FADD-TRADD-DKO TRAIL+C	17	*
FADD-KO TRAIL+C+N vs FADD-TRADD-DKO TRAIL+C+Z	-5,8	ns
FADD-KO TRAIL+C+N vs FADD-TRADD-DKO TRAIL+C+N	14	ns
FADD-KO TRAIL+C+N vs FADD-TRADD-DKO TRAIL+C+Z+N	-4,3	ns
FADD-KO TRAIL+C+N vs FADD-RIPK1-DKO TRAIL	-10	ns
FADD-KO TRAIL+C+N vs FADD-RIPK1-DKO TRAIL+Z	-8,5	ns
FADD-KO TRAIL+C+N vs FADD-RIPK1-DKO TRAIL+N	-3,3	ns
FADD-KO TRAIL+C+N vs FADD-RIPK1-DKO TRAIL+Z+N	-6,6	ns
FADD-KO TRAIL+C+N vs FADD-RIPK1-DKO TRAIL+C	1,9	ns
FADD-KO TRAIL+C+N vs FADD-RIPK1-DKO TRAIL+C+Z	1,3	ns
FADD-KO TRAIL+C+N vs FADD-RIPK1-DKO TRAIL+C+N	1,6	ns
FADD-KO TRAIL+C+N vs FADD-RIPK1-DKO TRAIL+C+Z+N	-5,7	ns
FADD-KO TRAIL+C+N vs TRADD-RIPK1-DKO TRAIL	12	ns
FADD-KO TRAIL+C+N vs TRADD-RIPK1-DKO TRAIL+Z	-2,9	ns
FADD-KO TRAIL+C+N vs TRADD-RIPK1-DKO TRAIL+N	21	***
FADD-KO TRAIL+C+N vs TRADD-RIPK1-DKO TRAIL+Z+N	-9,2	ns
FADD-KO TRAIL+C+N vs TRADD-RIPK1-DKO TRAIL+C	77	***

FADD-KO TRAIL+C+N vs TRADD-RIPK1-DKO TRAIL+C+Z	-9,1	ns
FADD-KO TRAIL+C+N vs TRADD-RIPK1-DKO TRAIL+C+N	78	***
FADD-KO TRAIL+C+N vs TRADD-RIPK1-DKO TRAIL+C+Z+N	-8,0	ns
FADD-KO TRAIL+C+N vs Casp.8-KO TRAIL	3,1	ns
FADD-KO TRAIL+C+N vs Casp.8-KO TRAIL+Z	12	ns
FADD-KO TRAIL+C+N vs Casp.8-KO TRAIL+N	-9,6	ns
FADD-KO TRAIL+C+N vs Casp.8-KO TRAIL+Z+N	-11	ns
FADD-KO TRAIL+C+N vs Casp.8-KO TRAIL+C	24	***
FADD-KO TRAIL+C+N vs Casp.8-KO TRAIL+C+Z	27	***
FADD-KO TRAIL+C+N vs Casp.8-KO TRAIL+C+N	-2,5	ns
FADD-KO TRAIL+C+N vs Casp.8-KO TRAIL+C+Z+N	-7,6	ns
FADD-KO TRAIL+C+N vs EV TRAIL	-7,8	ns
FADD-KO TRAIL+C+N vs EV TRAIL+Z	-9,0	ns
FADD-KO TRAIL+C+N vs EV TRAIL+N	8,0	ns
FADD-KO TRAIL+C+N vs EV TRAIL+Z+N	-4,0	ns
FADD-KO TRAIL+C+N vs EV TRAIL+C	68	***
FADD-KO TRAIL+C+N vs EV TRAIL+C+Z	-7,4	ns
FADD-KO TRAIL+C+N vs EV TRAIL+C+N	72	***
FADD-KO TRAIL+C+N vs EV TRAIL+C+Z+N	-4,2	ns
FADD-KO TRAIL+C+Z+N vs TRADD-KO TRAIL	-0,70	ns
FADD-KO TRAIL+C+Z+N vs TRADD-KO TRAIL+Z	8,8	ns
FADD-KO TRAIL+C+Z+N vs TRADD-KO TRAIL+N	13	ns
FADD-KO TRAIL+C+Z+N vs TRADD-KO TRAIL+Z+N	-8,0	ns
FADD-KO TRAIL+C+Z+N vs TRADD-KO TRAIL+C	84	***
FADD-KO TRAIL+C+Z+N vs TRADD-KO TRAIL+C+Z	81	***
FADD-KO TRAIL+C+Z+N vs TRADD-KO TRAIL+C+N	77	***
FADD-KO TRAIL+C+Z+N vs TRADD-KO TRAIL+C+Z+N	-4,2	ns
FADD-KO TRAIL+C+Z+N vs RIPK1-KO TRAIL	5,7	ns
FADD-KO TRAIL+C+Z+N vs RIPK1-KO TRAIL+Z	-4,2	ns
FADD-KO TRAIL+C+Z+N vs RIPK1-KO TRAIL+N	15	ns
FADD-KO TRAIL+C+Z+N vs RIPK1-KO TRAIL+Z+N	-1,6	ns
FADD-KO TRAIL+C+Z+N vs RIPK1-KO TRAIL+C	83	***
FADD-KO TRAIL+C+Z+N vs RIPK1-KO TRAIL+C+Z	-2,8	ns
FADD-KO TRAIL+C+Z+N vs RIPK1-KO TRAIL+C+N	81	***
FADD-KO TRAIL+C+Z+N vs RIPK1-KO TRAIL+C+Z+N	-2,7	ns
FADD-KO TRAIL+C+Z+N vs FADD-TRADD-DKO TRAIL	12	ns
FADD-KO TRAIL+C+Z+N vs FADD-TRADD-DKO TRAIL+Z	16	ns
FADD-KO TRAIL+C+Z+N vs FADD-TRADD-DKO TRAIL+N	15	ns
FADD-KO TRAIL+C+Z+N vs FADD-TRADD-DKO TRAIL+Z+N	-2,6	ns
FADD-KO TRAIL+C+Z+N vs FADD-TRADD-DKO TRAIL+C	21	***
FADD-KO TRAIL+C+Z+N vs FADD-TRADD-DKO TRAIL+C+Z	-2,6	ns
FADD-KO TRAIL+C+Z+N vs FADD-TRADD-DKO TRAIL+C+N	17	ns
FADD-KO TRAIL+C+Z+N vs FADD-TRADD-DKO TRAIL+C+Z+N	-1,1	ns
FADD-KO TRAIL+C+Z+N vs FADD-RIPK1-DKO TRAIL	-6,9	ns
FADD-KO TRAIL+C+Z+N vs FADD-RIPK1-DKO TRAIL+Z	-5,3	ns
FADD-KO TRAIL+C+Z+N vs FADD-RIPK1-DKO TRAIL+N	-0,14	ns
FADD-KO TRAIL+C+Z+N vs FADD-RIPK1-DKO TRAIL+Z+N	-3,4	ns
FADD-KO TRAIL+C+Z+N vs FADD-RIPK1-DKO TRAIL+C	5,1	ns
FADD-KO TRAIL+C+Z+N vs FADD-RIPK1-DKO TRAIL+C+Z	4,5	ns
FADD-KO TRAIL+C+Z+N vs FADD-RIPK1-DKO TRAIL+C+N	4,8	ns
FADD-KO TRAIL+C+Z+N vs FADD-RIPK1-DKO TRAIL+C+Z+N	-2,5	ns
FADD-KO TRAIL+C+Z+N vs TRADD-RIPK1-DKO TRAIL	15	ns
FADD-KO TRAIL+C+Z+N vs TRADD-RIPK1-DKO TRAIL+Z	0,32	ns
FADD-KO TRAIL+C+Z+N vs TRADD-RIPK1-DKO TRAIL+N	24	***

FADD-KO TRAIL+C+Z+N vs TRADD-RIPK1-DKO TRAIL+Z+N	-6,0	ns
FADD-KO TRAIL+C+Z+N vs TRADD-RIPK1-DKO TRAIL+C	80	***
FADD-KO TRAIL+C+Z+N vs TRADD-RIPK1-DKO TRAIL+C+Z	-5,9	ns
FADD-KO TRAIL+C+Z+N vs TRADD-RIPK1-DKO TRAIL+C+N	81	***
FADD-KO TRAIL+C+Z+N vs TRADD-RIPK1-DKO TRAIL+C+Z+N	-4,9	ns
FADD-KO TRAIL+C+Z+N vs Casp.8-KO TRAIL	6,3	ns
FADD-KO TRAIL+C+Z+N vs Casp.8-KO TRAIL+Z	15	ns
FADD-KO TRAIL+C+Z+N vs Casp.8-KO TRAIL+N	-6,4	ns
FADD-KO TRAIL+C+Z+N vs Casp.8-KO TRAIL+Z+N	-7,9	ns
FADD-KO TRAIL+C+Z+N vs Casp.8-KO TRAIL+C	27	***
FADD-KO TRAIL+C+Z+N vs Casp.8-KO TRAIL+C+Z	30	***
FADD-KO TRAIL+C+Z+N vs Casp.8-KO TRAIL+C+N	0,70	ns
FADD-KO TRAIL+C+Z+N vs Casp.8-KO TRAIL+C+Z+N	-4,4	ns
FADD-KO TRAIL+C+Z+N vs EV TRAIL	-4,6	ns
FADD-KO TRAIL+C+Z+N vs EV TRAIL+Z	-5,8	ns
FADD-KO TRAIL+C+Z+N vs EV TRAIL+N	11	ns
FADD-KO TRAIL+C+Z+N vs EV TRAIL+Z+N	-0,76	ns
FADD-KO TRAIL+C+Z+N vs EV TRAIL+C	72	***
FADD-KO TRAIL+C+Z+N vs EV TRAIL+C+Z	-4,2	ns
FADD-KO TRAIL+C+Z+N vs EV TRAIL+C+N	75	***
FADD-KO TRAIL+C+Z+N vs EV TRAIL+C+Z+N	-1,0	ns
TRADD-KO TRAIL vs TRADD-KO TRAIL+Z	9,5	ns
TRADD-KO TRAIL vs TRADD-KO TRAIL+N	14	ns
TRADD-KO TRAIL vs TRADD-KO TRAIL+Z+N	-7,3	ns
TRADD-KO TRAIL vs TRADD-KO TRAIL+C	85	***
TRADD-KO TRAIL vs TRADD-KO TRAIL+C+Z	82	***
TRADD-KO TRAIL vs TRADD-KO TRAIL+C+N	78	***
TRADD-KO TRAIL vs TRADD-KO TRAIL+C+Z+N	-3,5	ns
TRADD-KO TRAIL vs RIPK1-KO TRAIL	6,4	ns
TRADD-KO TRAIL vs RIPK1-KO TRAIL+Z	-3,5	ns
TRADD-KO TRAIL vs RIPK1-KO TRAIL+N	16	*
TRADD-KO TRAIL vs RIPK1-KO TRAIL+Z+N	-0,88	ns
TRADD-KO TRAIL vs RIPK1-KO TRAIL+C	84	***
TRADD-KO TRAIL vs RIPK1-KO TRAIL+C+Z	-2,1	ns
TRADD-KO TRAIL vs RIPK1-KO TRAIL+C+N	82	***
TRADD-KO TRAIL vs RIPK1-KO TRAIL+C+Z+N	-2,0	ns
TRADD-KO TRAIL vs FADD-TRADD-DKO TRAIL	12	ns
TRADD-KO TRAIL vs FADD-TRADD-DKO TRAIL+Z	16	*
TRADD-KO TRAIL vs FADD-TRADD-DKO TRAIL+N	16	ns
TRADD-KO TRAIL vs FADD-TRADD-DKO TRAIL+Z+N	-1,9	ns
TRADD-KO TRAIL vs FADD-TRADD-DKO TRAIL+C	21	***
TRADD-KO TRAIL vs FADD-TRADD-DKO TRAIL+C+Z	-1,9	ns
TRADD-KO TRAIL vs FADD-TRADD-DKO TRAIL+C+N	18	ns
TRADD-KO TRAIL vs FADD-TRADD-DKO TRAIL+C+Z+N	-0,36	ns
TRADD-KO TRAIL vs FADD-RIPK1-DKO TRAIL	-6,2	ns
TRADD-KO TRAIL vs FADD-RIPK1-DKO TRAIL+Z	-4,6	ns
TRADD-KO TRAIL vs FADD-RIPK1-DKO TRAIL+N	0,56	ns
TRADD-KO TRAIL vs FADD-RIPK1-DKO TRAIL+Z+N	-2,7	ns
TRADD-KO TRAIL vs FADD-RIPK1-DKO TRAIL+C	5,8	ns
TRADD-KO TRAIL vs FADD-RIPK1-DKO TRAIL+C+Z	5,2	ns
TRADD-KO TRAIL vs FADD-RIPK1-DKO TRAIL+C+N	5,5	ns
TRADD-KO TRAIL vs FADD-RIPK1-DKO TRAIL+C+Z+N	-1,8	ns
TRADD-KO TRAIL vs TRADD-RIPK1-DKO TRAIL	16	ns
TRADD-KO TRAIL vs TRADD-RIPK1-DKO TRAIL+Z	1,0	ns

TRADD-KO TRAIL vs TRADD-RIPK1-DKO TRAIL+N	25	***
TRADD-KO TRAIL vs TRADD-RIPK1-DKO TRAIL+Z+N	-5,3	ns
TRADD-KO TRAIL vs TRADD-RIPK1-DKO TRAIL+C	81	***
TRADD-KO TRAIL vs TRADD-RIPK1-DKO TRAIL+C+Z	-5,2	ns
TRADD-KO TRAIL vs TRADD-RIPK1-DKO TRAIL+C+N	81	***
TRADD-KO TRAIL vs TRADD-RIPK1-DKO TRAIL+C+Z+N	-4,2	ns
TRADD-KO TRAIL vs Casp.8-KO TRAIL	7,0	ns
TRADD-KO TRAIL vs Casp.8-KO TRAIL+Z	16	*
TRADD-KO TRAIL vs Casp.8-KO TRAIL+N	-5,7	ns
TRADD-KO TRAIL vs Casp.8-KO TRAIL+Z+N	-7,2	ns
TRADD-KO TRAIL vs Casp.8-KO TRAIL+C	28	***
TRADD-KO TRAIL vs Casp.8-KO TRAIL+C+Z	31	***
TRADD-KO TRAIL vs Casp.8-KO TRAIL+C+N	1,4	ns
TRADD-KO TRAIL vs Casp.8-KO TRAIL+C+Z+N	-3,7	ns
TRADD-KO TRAIL vs EV TRAIL	-3,9	ns
TRADD-KO TRAIL vs EV TRAIL+Z	-5,1	ns
TRADD-KO TRAIL vs EV TRAIL+N	12	ns
TRADD-KO TRAIL vs EV TRAIL+Z+N	-0,063	ns
TRADD-KO TRAIL vs EV TRAIL+C	72	***
TRADD-KO TRAIL vs EV TRAIL+C+Z	-3,5	ns
TRADD-KO TRAIL vs EV TRAIL+C+N	75	***
TRADD-KO TRAIL vs EV TRAIL+C+Z+N	-0,31	ns
TRADD-KO TRAIL+Z vs TRADD-KO TRAIL+N	4,2	ns
TRADD-KO TRAIL+Z vs TRADD-KO TRAIL+Z+N	-17	ns
TRADD-KO TRAIL+Z vs TRADD-KO TRAIL+C	75	***
TRADD-KO TRAIL+Z vs TRADD-KO TRAIL+C+Z	72	***
TRADD-KO TRAIL+Z vs TRADD-KO TRAIL+C+N	68	***
TRADD-KO TRAIL+Z vs TRADD-KO TRAIL+C+Z+N	-13	ns
TRADD-KO TRAIL+Z vs RIPK1-KO TRAIL	-3,2	ns
TRADD-KO TRAIL+Z vs RIPK1-KO TRAIL+Z	-13	ns
TRADD-KO TRAIL+Z vs RIPK1-KO TRAIL+N	6,3	ns
TRADD-KO TRAIL+Z vs RIPK1-KO TRAIL+Z+N	-10	ns
TRADD-KO TRAIL+Z vs RIPK1-KO TRAIL+C	74	***
TRADD-KO TRAIL+Z vs RIPK1-KO TRAIL+C+Z	-12	ns
TRADD-KO TRAIL+Z vs RIPK1-KO TRAIL+C+N	72	***
TRADD-KO TRAIL+Z vs RIPK1-KO TRAIL+C+Z+N	-12	ns
TRADD-KO TRAIL+Z vs FADD-TRADD-DKO TRAIL	2,7	ns
TRADD-KO TRAIL+Z vs FADD-TRADD-DKO TRAIL+Z	6,9	ns
TRADD-KO TRAIL+Z vs FADD-TRADD-DKO TRAIL+N	6,5	ns
TRADD-KO TRAIL+Z vs FADD-TRADD-DKO TRAIL+Z+N	-11	ns
TRADD-KO TRAIL+Z vs FADD-TRADD-DKO TRAIL+C	12	ns
TRADD-KO TRAIL+Z vs FADD-TRADD-DKO TRAIL+C+Z	-11	ns
TRADD-KO TRAIL+Z vs FADD-TRADD-DKO TRAIL+C+N	8,3	ns
TRADD-KO TRAIL+Z vs FADD-TRADD-DKO TRAIL+C+Z+N	-9,9	ns
TRADD-KO TRAIL+Z vs FADD-RIPK1-DKO TRAIL	-16	ns
TRADD-KO TRAIL+Z vs FADD-RIPK1-DKO TRAIL+Z	-14	ns
TRADD-KO TRAIL+Z vs FADD-RIPK1-DKO TRAIL+N	-9,0	ns
TRADD-KO TRAIL+Z vs FADD-RIPK1-DKO TRAIL+Z+N	-12	ns
TRADD-KO TRAIL+Z vs FADD-RIPK1-DKO TRAIL+C	-3,7	ns
TRADD-KO TRAIL+Z vs FADD-RIPK1-DKO TRAIL+C+Z	-4,3	ns
TRADD-KO TRAIL+Z vs FADD-RIPK1-DKO TRAIL+C+N	-4,0	ns
TRADD-KO TRAIL+Z vs FADD-RIPK1-DKO TRAIL+C+Z+N	-11	ns
TRADD-KO TRAIL+Z vs TRADD-RIPK1-DKO TRAIL	6,5	ns
TRADD-KO TRAIL+Z vs TRADD-RIPK1-DKO TRAIL+Z	-8,5	ns

TRADD-KO TRAIL+Z vs TRADD-RIPK1-DKO TRAIL+N	15	ns
TRADD-KO TRAIL+Z vs TRADD-RIPK1-DKO TRAIL+Z+N	-15	ns
TRADD-KO TRAIL+Z vs TRADD-RIPK1-DKO TRAIL+C	71	***
TRADD-KO TRAIL+Z vs TRADD-RIPK1-DKO TRAIL+C+Z	-15	ns
TRADD-KO TRAIL+Z vs TRADD-RIPK1-DKO TRAIL+C+N	72	***
TRADD-KO TRAIL+Z vs TRADD-RIPK1-DKO TRAIL+C+Z+N	-14	ns
TRADD-KO TRAIL+Z vs Casp.8-KO TRAIL	-2,5	ns
TRADD-KO TRAIL+Z vs Casp.8-KO TRAIL+Z	6,4	ns
TRADD-KO TRAIL+Z vs Casp.8-KO TRAIL+N	-15	ns
TRADD-KO TRAIL+Z vs Casp.8-KO TRAIL+Z+N	-17	ns
TRADD-KO TRAIL+Z vs Casp.8-KO TRAIL+C	18	*
TRADD-KO TRAIL+Z vs Casp.8-KO TRAIL+C+Z	21	*
TRADD-KO TRAIL+Z vs Casp.8-KO TRAIL+C+N	-8,1	ns
TRADD-KO TRAIL+Z vs Casp.8-KO TRAIL+C+Z+N	-13	ns
TRADD-KO TRAIL+Z vs EV TRAIL	-13	ns
TRADD-KO TRAIL+Z vs EV TRAIL+Z	-15	ns
TRADD-KO TRAIL+Z vs EV TRAIL+N	2,4	ns
TRADD-KO TRAIL+Z vs EV TRAIL+Z+N	-9,6	ns
TRADD-KO TRAIL+Z vs EV TRAIL+C	63	***
TRADD-KO TRAIL+Z vs EV TRAIL+C+Z	-13	ns
TRADD-KO TRAIL+Z vs EV TRAIL+C+N	66	***
TRADD-KO TRAIL+Z vs EV TRAIL+C+Z+N	-9,8	ns
TRADD-KO TRAIL+N vs TRADD-KO TRAIL+Z+N	-21	**
TRADD-KO TRAIL+N vs TRADD-KO TRAIL+C	71	***
TRADD-KO TRAIL+N vs TRADD-KO TRAIL+C+Z	68	***
TRADD-KO TRAIL+N vs TRADD-KO TRAIL+C+N	64	***
TRADD-KO TRAIL+N vs TRADD-KO TRAIL+C+Z+N	-17	ns
TRADD-KO TRAIL+N vs RIPK1-KO TRAIL	-7,3	ns
TRADD-KO TRAIL+N vs RIPK1-KO TRAIL+Z	-17	ns
TRADD-KO TRAIL+N vs RIPK1-KO TRAIL+N	2,1	ns
TRADD-KO TRAIL+N vs RIPK1-KO TRAIL+Z+N	-15	ns
TRADD-KO TRAIL+N vs RIPK1-KO TRAIL+C	70	***
TRADD-KO TRAIL+N vs RIPK1-KO TRAIL+C+Z	-16	ns
TRADD-KO TRAIL+N vs RIPK1-KO TRAIL+C+N	68	***
TRADD-KO TRAIL+N vs RIPK1-KO TRAIL+C+Z+N	-16	ns
TRADD-KO TRAIL+N vs FADD-TRADD-DKO TRAIL	-1,5	ns
TRADD-KO TRAIL+N vs FADD-TRADD-DKO TRAIL+Z	2,8	ns
TRADD-KO TRAIL+N vs FADD-TRADD-DKO TRAIL+N	2,3	ns
TRADD-KO TRAIL+N vs FADD-TRADD-DKO TRAIL+Z+N	-16	ns
TRADD-KO TRAIL+N vs FADD-TRADD-DKO TRAIL+C	7,7	ns
TRADD-KO TRAIL+N vs FADD-TRADD-DKO TRAIL+C+Z	-16	ns
TRADD-KO TRAIL+N vs FADD-TRADD-DKO TRAIL+C+N	4,1	ns
TRADD-KO TRAIL+N vs FADD-TRADD-DKO TRAIL+C+Z+N	-14	ns
TRADD-KO TRAIL+N vs FADD-RIPK1-DKO TRAIL	-20	*
TRADD-KO TRAIL+N vs FADD-RIPK1-DKO TRAIL+Z	-18	*
TRADD-KO TRAIL+N vs FADD-RIPK1-DKO TRAIL+N	-13	ns
TRADD-KO TRAIL+N vs FADD-RIPK1-DKO TRAIL+Z+N	-16	ns
TRADD-KO TRAIL+N vs FADD-RIPK1-DKO TRAIL+C	-7,9	ns
TRADD-KO TRAIL+N vs FADD-RIPK1-DKO TRAIL+C+Z	-8,5	ns
TRADD-KO TRAIL+N vs FADD-RIPK1-DKO TRAIL+C+N	-8,2	ns
TRADD-KO TRAIL+N vs FADD-RIPK1-DKO TRAIL+C+Z+N	-15	ns
TRADD-KO TRAIL+N vs TRADD-RIPK1-DKO TRAIL	2,3	ns
TRADD-KO TRAIL+N vs TRADD-RIPK1-DKO TRAIL+Z	-13	ns
TRADD-KO TRAIL+N vs TRADD-RIPK1-DKO TRAIL+N	11	ns

TRADD-KO TRAIL+N vs TRADD-RIPK1-DKO TRAIL+Z+N	-19	ns
TRADD-KO TRAIL+N vs TRADD-RIPK1-DKO TRAIL+C	67	***
TRADD-KO TRAIL+N vs TRADD-RIPK1-DKO TRAIL+C+Z	-19	ns
TRADD-KO TRAIL+N vs TRADD-RIPK1-DKO TRAIL+C+N	68	***
TRADD-KO TRAIL+N vs TRADD-RIPK1-DKO TRAIL+C+Z+N	-18	ns
TRADD-KO TRAIL+N vs Casp.8-KO TRAIL	-6,7	ns
TRADD-KO TRAIL+N vs Casp.8-KO TRAIL+Z	2,2	ns
TRADD-KO TRAIL+N vs Casp.8-KO TRAIL+N	-19	*
TRADD-KO TRAIL+N vs Casp.8-KO TRAIL+Z+N	-21	*
TRADD-KO TRAIL+N vs Casp.8-KO TRAIL+C	14	ns
TRADD-KO TRAIL+N vs Casp.8-KO TRAIL+C+Z	17	ns
TRADD-KO TRAIL+N vs Casp.8-KO TRAIL+C+N	-12	ns
TRADD-KO TRAIL+N vs Casp.8-KO TRAIL+C+Z+N	-17	ns
TRADD-KO TRAIL+N vs EV TRAIL	-18	ns
TRADD-KO TRAIL+N vs EV TRAIL+Z	-19	ns
TRADD-KO TRAIL+N vs EV TRAIL+N	-1,8	ns
TRADD-KO TRAIL+N vs EV TRAIL+Z+N	-14	ns
TRADD-KO TRAIL+N vs EV TRAIL+C	59	***
TRADD-KO TRAIL+N vs EV TRAIL+C+Z	-17	ns
TRADD-KO TRAIL+N vs EV TRAIL+C+N	62	***
TRADD-KO TRAIL+N vs EV TRAIL+C+Z+N	-14	ns
TRADD-KO TRAIL+Z+N vs TRADD-KO TRAIL+C	92	***
TRADD-KO TRAIL+Z+N vs TRADD-KO TRAIL+C+Z	89	***
TRADD-KO TRAIL+Z+N vs TRADD-KO TRAIL+C+N	85	***
TRADD-KO TRAIL+Z+N vs TRADD-KO TRAIL+C+Z+N	3,8	ns
TRADD-KO TRAIL+Z+N vs RIPK1-KO TRAIL	14	ns
TRADD-KO TRAIL+Z+N vs RIPK1-KO TRAIL+Z	3,8	ns
TRADD-KO TRAIL+Z+N vs RIPK1-KO TRAIL+N	23	***
TRADD-KO TRAIL+Z+N vs RIPK1-KO TRAIL+Z+N	6,4	ns
TRADD-KO TRAIL+Z+N vs RIPK1-KO TRAIL+C	91	***
TRADD-KO TRAIL+Z+N vs RIPK1-KO TRAIL+C+Z	5,3	ns
TRADD-KO TRAIL+Z+N vs RIPK1-KO TRAIL+C+N	89	***
TRADD-KO TRAIL+Z+N vs RIPK1-KO TRAIL+C+Z+N	5,3	ns
TRADD-KO TRAIL+Z+N vs FADD-TRADD-DKO TRAIL	20	**
TRADD-KO TRAIL+Z+N vs FADD-TRADD-DKO TRAIL+Z	24	***
TRADD-KO TRAIL+Z+N vs FADD-TRADD-DKO TRAIL+N	23	***
TRADD-KO TRAIL+Z+N vs FADD-TRADD-DKO TRAIL+Z+N	5,4	ns
TRADD-KO TRAIL+Z+N vs FADD-TRADD-DKO TRAIL+C	29	***
TRADD-KO TRAIL+Z+N vs FADD-TRADD-DKO TRAIL+C+Z	5,4	ns
TRADD-KO TRAIL+Z+N vs FADD-TRADD-DKO TRAIL+C+N	25	***
TRADD-KO TRAIL+Z+N vs FADD-TRADD-DKO TRAIL+C+Z+N	6,9	ns
TRADD-KO TRAIL+Z+N vs FADD-RIPK1-DKO TRAIL	1,1	ns
TRADD-KO TRAIL+Z+N vs FADD-RIPK1-DKO TRAIL+Z	2,7	ns
TRADD-KO TRAIL+Z+N vs FADD-RIPK1-DKO TRAIL+N	7,9	ns
TRADD-KO TRAIL+Z+N vs FADD-RIPK1-DKO TRAIL+Z+N	4,6	ns
TRADD-KO TRAIL+Z+N vs FADD-RIPK1-DKO TRAIL+C	13	ns
TRADD-KO TRAIL+Z+N vs FADD-RIPK1-DKO TRAIL+C+Z	13	ns
TRADD-KO TRAIL+Z+N vs FADD-RIPK1-DKO TRAIL+C+N	13	ns
TRADD-KO TRAIL+Z+N vs FADD-RIPK1-DKO TRAIL+C+Z+N	5,6	ns
TRADD-KO TRAIL+Z+N vs TRADD-RIPK1-DKO TRAIL	23	***
TRADD-KO TRAIL+Z+N vs TRADD-RIPK1-DKO TRAIL+Z	8,3	ns
TRADD-KO TRAIL+Z+N vs TRADD-RIPK1-DKO TRAIL+N	32	***
TRADD-KO TRAIL+Z+N vs TRADD-RIPK1-DKO TRAIL+Z+N	2,1	ns
TRADD-KO TRAIL+Z+N vs TRADD-RIPK1-DKO TRAIL+C	88	***

TRADD-KO TRAIL+Z+N vs TRADD-RIPK1-DKO TRAIL+C+Z	2,1	ns
TRADD-KO TRAIL+Z+N vs TRADD-RIPK1-DKO TRAIL+C+N	89	***
TRADD-KO TRAIL+Z+N vs TRADD-RIPK1-DKO TRAIL+C+Z+N	3,2	ns
TRADD-KO TRAIL+Z+N vs Casp.8-KO TRAIL	14	ns
TRADD-KO TRAIL+Z+N vs Casp.8-KO TRAIL+Z	23	***
TRADD-KO TRAIL+Z+N vs Casp.8-KO TRAIL+N	1,7	ns
TRADD-KO TRAIL+Z+N vs Casp.8-KO TRAIL+Z+N	0,14	ns
TRADD-KO TRAIL+Z+N vs Casp.8-KO TRAIL+C	35	***
TRADD-KO TRAIL+Z+N vs Casp.8-KO TRAIL+C+Z	38	***
TRADD-KO TRAIL+Z+N vs Casp.8-KO TRAIL+C+N	8,7	ns
TRADD-KO TRAIL+Z+N vs Casp.8-KO TRAIL+C+Z+N	3,7	ns
TRADD-KO TRAIL+Z+N vs EV TRAIL	3,4	ns
TRADD-KO TRAIL+Z+N vs EV TRAIL+Z	2,3	ns
TRADD-KO TRAIL+Z+N vs EV TRAIL+N	19	ns
TRADD-KO TRAIL+Z+N vs EV TRAIL+Z+N	7,3	ns
TRADD-KO TRAIL+Z+N vs EV TRAIL+C	80	***
TRADD-KO TRAIL+Z+N vs EV TRAIL+C+Z	3,8	ns
TRADD-KO TRAIL+Z+N vs EV TRAIL+C+N	83	***
TRADD-KO TRAIL+Z+N vs EV TRAIL+C+Z+N	7,0	ns
TRADD-KO TRAIL+C vs TRADD-KO TRAIL+C+Z	-2,8	ns
TRADD-KO TRAIL+C vs TRADD-KO TRAIL+C+N	-6,7	ns
TRADD-KO TRAIL+C vs TRADD-KO TRAIL+C+Z+N	-88	***
TRADD-KO TRAIL+C vs RIPK1-KO TRAIL	-78	***
TRADD-KO TRAIL+C vs RIPK1-KO TRAIL+Z	-88	***
TRADD-KO TRAIL+C vs RIPK1-KO TRAIL+N	-69	***
TRADD-KO TRAIL+C vs RIPK1-KO TRAIL+Z+N	-85	***
TRADD-KO TRAIL+C vs RIPK1-KO TRAIL+C	-1,1	ns
TRADD-KO TRAIL+C vs RIPK1-KO TRAIL+C+Z	-87	***
TRADD-KO TRAIL+C vs RIPK1-KO TRAIL+C+N	-2,9	ns
TRADD-KO TRAIL+C vs RIPK1-KO TRAIL+C+Z+N	-87	***
TRADD-KO TRAIL+C vs FADD-TRADD-DKO TRAIL	-72	***
TRADD-KO TRAIL+C vs FADD-TRADD-DKO TRAIL+Z	-68	***
TRADD-KO TRAIL+C vs FADD-TRADD-DKO TRAIL+N	-69	***
TRADD-KO TRAIL+C vs FADD-TRADD-DKO TRAIL+Z+N	-87	***
TRADD-KO TRAIL+C vs FADD-TRADD-DKO TRAIL+C	-63	***
TRADD-KO TRAIL+C vs FADD-TRADD-DKO TRAIL+C+Z	-86	***
TRADD-KO TRAIL+C vs FADD-TRADD-DKO TRAIL+C+N	-67	***
TRADD-KO TRAIL+C vs FADD-TRADD-DKO TRAIL+C+Z+N	-85	***
TRADD-KO TRAIL+C vs FADD-RIPK1-DKO TRAIL	-91	***
TRADD-KO TRAIL+C vs FADD-RIPK1-DKO TRAIL+Z	-89	***
TRADD-KO TRAIL+C vs FADD-RIPK1-DKO TRAIL+N	-84	***
TRADD-KO TRAIL+C vs FADD-RIPK1-DKO TRAIL+Z+N	-87	***
TRADD-KO TRAIL+C vs FADD-RIPK1-DKO TRAIL+C	-79	***
TRADD-KO TRAIL+C vs FADD-RIPK1-DKO TRAIL+C+Z	-79	***
TRADD-KO TRAIL+C vs FADD-RIPK1-DKO TRAIL+C+N	-79	***
TRADD-KO TRAIL+C vs FADD-RIPK1-DKO TRAIL+C+Z+N	-86	***
TRADD-KO TRAIL+C vs TRADD-RIPK1-DKO TRAIL	-69	***
TRADD-KO TRAIL+C vs TRADD-RIPK1-DKO TRAIL+Z	-84	***
TRADD-KO TRAIL+C vs TRADD-RIPK1-DKO TRAIL+N	-60	***
TRADD-KO TRAIL+C vs TRADD-RIPK1-DKO TRAIL+Z+N	-90	***
TRADD-KO TRAIL+C vs TRADD-RIPK1-DKO TRAIL+C	-3,9	ns
TRADD-KO TRAIL+C vs TRADD-RIPK1-DKO TRAIL+C+Z	-90	***
TRADD-KO TRAIL+C vs TRADD-RIPK1-DKO TRAIL+C+N	-3,2	ns
TRADD-KO TRAIL+C vs TRADD-RIPK1-DKO TRAIL+C+Z+N	-89	***

TRADD-KO TRAIL+C vs Casp.8-KO TRAIL	-78	***
TRADD-KO TRAIL+C vs Casp.8-KO TRAIL+Z	-69	***
TRADD-KO TRAIL+C vs Casp.8-KO TRAIL+N	-90	***
TRADD-KO TRAIL+C vs Casp.8-KO TRAIL+Z+N	-92	***
TRADD-KO TRAIL+C vs Casp.8-KO TRAIL+C	-57	***
TRADD-KO TRAIL+C vs Casp.8-KO TRAIL+C+Z	-54	***
TRADD-KO TRAIL+C vs Casp.8-KO TRAIL+C+N	-83	***
TRADD-KO TRAIL+C vs Casp.8-KO TRAIL+C+Z+N	-88	***
TRADD-KO TRAIL+C vs EV TRAIL	-89	***
TRADD-KO TRAIL+C vs EV TRAIL+Z	-90	***
TRADD-KO TRAIL+C vs EV TRAIL+N	-73	***
TRADD-KO TRAIL+C vs EV TRAIL+Z+N	-85	***
TRADD-KO TRAIL+C vs EV TRAIL+C	-12	ns
TRADD-KO TRAIL+C vs EV TRAIL+C+Z	-88	***
TRADD-KO TRAIL+C vs EV TRAIL+C+N	-9,2	ns
TRADD-KO TRAIL+C vs EV TRAIL+C+Z+N	-85	***
TRADD-KO TRAIL+C+Z vs TRADD-KO TRAIL+C+N	-3,8	ns
TRADD-KO TRAIL+C+Z vs TRADD-KO TRAIL+C+Z+N	-85	***
TRADD-KO TRAIL+C+Z vs RIPK1-KO TRAIL	-75	***
TRADD-KO TRAIL+C+Z vs RIPK1-KO TRAIL+Z	-85	***
TRADD-KO TRAIL+C+Z vs RIPK1-KO TRAIL+N	-66	***
TRADD-KO TRAIL+C+Z vs RIPK1-KO TRAIL+Z+N	-83	***
TRADD-KO TRAIL+C+Z vs RIPK1-KO TRAIL+C	1,7	ns
TRADD-KO TRAIL+C+Z vs RIPK1-KO TRAIL+C+Z	-84	***
TRADD-KO TRAIL+C+Z vs RIPK1-KO TRAIL+C+N	-0,033	ns
TRADD-KO TRAIL+C+Z vs RIPK1-KO TRAIL+C+Z+N	-84	***
TRADD-KO TRAIL+C+Z vs FADD-TRADD-DKO TRAIL	-70	***
TRADD-KO TRAIL+C+Z vs FADD-TRADD-DKO TRAIL+Z	-65	***
TRADD-KO TRAIL+C+Z vs FADD-TRADD-DKO TRAIL+N	-66	***
TRADD-KO TRAIL+C+Z vs FADD-TRADD-DKO TRAIL+Z+N	-84	***
TRADD-KO TRAIL+C+Z vs FADD-TRADD-DKO TRAIL+C	-60	***
TRADD-KO TRAIL+C+Z vs FADD-TRADD-DKO TRAIL+C+Z	-84	***
TRADD-KO TRAIL+C+Z vs FADD-TRADD-DKO TRAIL+C+N	-64	***
TRADD-KO TRAIL+C+Z vs FADD-TRADD-DKO TRAIL+C+Z+N	-82	***
TRADD-KO TRAIL+C+Z vs FADD-RIPK1-DKO TRAIL	-88	***
TRADD-KO TRAIL+C+Z vs FADD-RIPK1-DKO TRAIL+Z	-86	***
TRADD-KO TRAIL+C+Z vs FADD-RIPK1-DKO TRAIL+N	-81	***
TRADD-KO TRAIL+C+Z vs FADD-RIPK1-DKO TRAIL+Z+N	-84	***
TRADD-KO TRAIL+C+Z vs FADD-RIPK1-DKO TRAIL+C	-76	***
TRADD-KO TRAIL+C+Z vs FADD-RIPK1-DKO TRAIL+C+Z	-77	***
TRADD-KO TRAIL+C+Z vs FADD-RIPK1-DKO TRAIL+C+N	-76	***
TRADD-KO TRAIL+C+Z vs FADD-RIPK1-DKO TRAIL+C+Z+N	-84	***
TRADD-KO TRAIL+C+Z vs TRADD-RIPK1-DKO TRAIL	-66	***
TRADD-KO TRAIL+C+Z vs TRADD-RIPK1-DKO TRAIL+Z	-81	***
TRADD-KO TRAIL+C+Z vs TRADD-RIPK1-DKO TRAIL+N	-57	***
TRADD-KO TRAIL+C+Z vs TRADD-RIPK1-DKO TRAIL+Z+N	-87	***
TRADD-KO TRAIL+C+Z vs TRADD-RIPK1-DKO TRAIL+C	-1,1	ns
TRADD-KO TRAIL+C+Z vs TRADD-RIPK1-DKO TRAIL+C+Z	-87	***
TRADD-KO TRAIL+C+Z vs TRADD-RIPK1-DKO TRAIL+C+N	-0,33	ns
TRADD-KO TRAIL+C+Z vs TRADD-RIPK1-DKO TRAIL+C+Z+N	-86	***
TRADD-KO TRAIL+C+Z vs Casp.8-KO TRAIL	-75	***
TRADD-KO TRAIL+C+Z vs Casp.8-KO TRAIL+Z	-66	***
TRADD-KO TRAIL+C+Z vs Casp.8-KO TRAIL+N	-87	***
TRADD-KO TRAIL+C+Z vs Casp.8-KO TRAIL+Z+N	-89	***

TRADD-KO TRAIL+C+Z vs Casp.8-KO TRAIL+C	-54	***
TRADD-KO TRAIL+C+Z vs Casp.8-KO TRAIL+C+Z	-51	***
TRADD-KO TRAIL+C+Z vs Casp.8-KO TRAIL+C+N	-80	***
TRADD-KO TRAIL+C+Z vs Casp.8-KO TRAIL+C+Z+N	-85	***
TRADD-KO TRAIL+C+Z vs EV TRAIL	-86	***
TRADD-KO TRAIL+C+Z vs EV TRAIL+Z	-87	***
TRADD-KO TRAIL+C+Z vs EV TRAIL+N	-70	***
TRADD-KO TRAIL+C+Z vs EV TRAIL+Z+N	-82	***
TRADD-KO TRAIL+C+Z vs EV TRAIL+C	-9,5	ns
TRADD-KO TRAIL+C+Z vs EV TRAIL+C+Z	-85	***
TRADD-KO TRAIL+C+Z vs EV TRAIL+C+N	-6,3	ns
TRADD-KO TRAIL+C+Z vs EV TRAIL+C+Z+N	-82	***
TRADD-KO TRAIL+C+N vs TRADD-KO TRAIL+C+Z+N	-81	***
TRADD-KO TRAIL+C+N vs RIPK1-KO TRAIL	-72	***
TRADD-KO TRAIL+C+N vs RIPK1-KO TRAIL+Z	-81	***
TRADD-KO TRAIL+C+N vs RIPK1-KO TRAIL+N	-62	***
TRADD-KO TRAIL+C+N vs RIPK1-KO TRAIL+Z+N	-79	***
TRADD-KO TRAIL+C+N vs RIPK1-KO TRAIL+C	5,6	ns
TRADD-KO TRAIL+C+N vs RIPK1-KO TRAIL+C+Z	-80	***
TRADD-KO TRAIL+C+N vs RIPK1-KO TRAIL+C+N	3,8	ns
TRADD-KO TRAIL+C+N vs RIPK1-KO TRAIL+C+Z+N	-80	***
TRADD-KO TRAIL+C+N vs FADD-TRADD-DKO TRAIL	-66	***
TRADD-KO TRAIL+C+N vs FADD-TRADD-DKO TRAIL+Z	-61	***
TRADD-KO TRAIL+C+N vs FADD-TRADD-DKO TRAIL+N	-62	***
TRADD-KO TRAIL+C+N vs FADD-TRADD-DKO TRAIL+Z+N	-80	***
TRADD-KO TRAIL+C+N vs FADD-TRADD-DKO TRAIL+C	-57	***
TRADD-KO TRAIL+C+N vs FADD-TRADD-DKO TRAIL+C+Z	-80	***
TRADD-KO TRAIL+C+N vs FADD-TRADD-DKO TRAIL+C+N	-60	***
TRADD-KO TRAIL+C+N vs FADD-TRADD-DKO TRAIL+C+Z+N	-78	***
TRADD-KO TRAIL+C+N vs FADD-RIPK1-DKO TRAIL	-84	***
TRADD-KO TRAIL+C+N vs FADD-RIPK1-DKO TRAIL+Z	-83	***
TRADD-KO TRAIL+C+N vs FADD-RIPK1-DKO TRAIL+N	-77	***
TRADD-KO TRAIL+C+N vs FADD-RIPK1-DKO TRAIL+Z+N	-81	***
TRADD-KO TRAIL+C+N vs FADD-RIPK1-DKO TRAIL+C	-72	***
TRADD-KO TRAIL+C+N vs FADD-RIPK1-DKO TRAIL+C+Z	-73	***
TRADD-KO TRAIL+C+N vs FADD-RIPK1-DKO TRAIL+C+N	-72	***
TRADD-KO TRAIL+C+N vs FADD-RIPK1-DKO TRAIL+C+Z+N	-80	***
TRADD-KO TRAIL+C+N vs TRADD-RIPK1-DKO TRAIL	-62	***
TRADD-KO TRAIL+C+N vs TRADD-RIPK1-DKO TRAIL+Z	-77	***
TRADD-KO TRAIL+C+N vs TRADD-RIPK1-DKO TRAIL+N	-53	***
TRADD-KO TRAIL+C+N vs TRADD-RIPK1-DKO TRAIL+Z+N	-83	***
TRADD-KO TRAIL+C+N vs TRADD-RIPK1-DKO TRAIL+C	2,8	ns
TRADD-KO TRAIL+C+N vs TRADD-RIPK1-DKO TRAIL+C+Z	-83	***
TRADD-KO TRAIL+C+N vs TRADD-RIPK1-DKO TRAIL+C+N	3,5	ns
TRADD-KO TRAIL+C+N vs TRADD-RIPK1-DKO TRAIL+C+Z+N	-82	***
TRADD-KO TRAIL+C+N vs Casp.8-KO TRAIL	-71	***
TRADD-KO TRAIL+C+N vs Casp.8-KO TRAIL+Z	-62	***
TRADD-KO TRAIL+C+N vs Casp.8-KO TRAIL+N	-84	***
TRADD-KO TRAIL+C+N vs Casp.8-KO TRAIL+Z+N	-85	***
TRADD-KO TRAIL+C+N vs Casp.8-KO TRAIL+C	-50	***
TRADD-KO TRAIL+C+N vs Casp.8-KO TRAIL+C+Z	-47	***
TRADD-KO TRAIL+C+N vs Casp.8-KO TRAIL+C+N	-77	***
TRADD-KO TRAIL+C+N vs Casp.8-KO TRAIL+C+Z+N	-82	***
TRADD-KO TRAIL+C+N vs EV TRAIL	-82	***

TRADD-KO TRAIL+C+N vs EV TRAIL+Z	-83	***
TRADD-KO TRAIL+C+N vs EV TRAIL+N	-66	***
TRADD-KO TRAIL+C+N vs EV TRAIL+Z+N	-78	***
TRADD-KO TRAIL+C+N vs EV TRAIL+C	-5,7	ns
TRADD-KO TRAIL+C+N vs EV TRAIL+C+Z	-81	***
TRADD-KO TRAIL+C+N vs EV TRAIL+C+N	-2,5	ns
TRADD-KO TRAIL+C+N vs EV TRAIL+C+Z+N	-78	***
TRADD-KO TRAIL+C+Z+N vs RIPK1-KO TRAIL	9,9	ns
TRADD-KO TRAIL+C+Z+N vs RIPK1-KO TRAIL+Z	-0,022	ns
TRADD-KO TRAIL+C+Z+N vs RIPK1-KO TRAIL+N	19	*
TRADD-KO TRAIL+C+Z+N vs RIPK1-KO TRAIL+Z+N	2,6	ns
TRADD-KO TRAIL+C+Z+N vs RIPK1-KO TRAIL+C	87	***
TRADD-KO TRAIL+C+Z+N vs RIPK1-KO TRAIL+C+Z	1,4	ns
TRADD-KO TRAIL+C+Z+N vs RIPK1-KO TRAIL+C+N	85	***
TRADD-KO TRAIL+C+Z+N vs RIPK1-KO TRAIL+C+Z+N	1,5	ns
TRADD-KO TRAIL+C+Z+N vs FADD-TRADD-DKO TRAIL	16	ns
TRADD-KO TRAIL+C+Z+N vs FADD-TRADD-DKO TRAIL+Z	20	*
TRADD-KO TRAIL+C+Z+N vs FADD-TRADD-DKO TRAIL+N	20	*
TRADD-KO TRAIL+C+Z+N vs FADD-TRADD-DKO TRAIL+Z+N	1,6	ns
TRADD-KO TRAIL+C+Z+N vs FADD-TRADD-DKO TRAIL+C	25	***
TRADD-KO TRAIL+C+Z+N vs FADD-TRADD-DKO TRAIL+C+Z	1,6	ns
TRADD-KO TRAIL+C+Z+N vs FADD-TRADD-DKO TRAIL+C+N	21	*
TRADD-KO TRAIL+C+Z+N vs FADD-TRADD-DKO TRAIL+C+Z+N	3,1	ns
TRADD-KO TRAIL+C+Z+N vs FADD-RIPK1-DKO TRAIL	-2,7	ns
TRADD-KO TRAIL+C+Z+N vs FADD-RIPK1-DKO TRAIL+Z	-1,1	ns
TRADD-KO TRAIL+C+Z+N vs FADD-RIPK1-DKO TRAIL+N	4,1	ns
TRADD-KO TRAIL+C+Z+N vs FADD-RIPK1-DKO TRAIL+Z+N	0,81	ns
TRADD-KO TRAIL+C+Z+N vs FADD-RIPK1-DKO TRAIL+C	9,3	ns
TRADD-KO TRAIL+C+Z+N vs FADD-RIPK1-DKO TRAIL+C+Z	8,7	ns
TRADD-KO TRAIL+C+Z+N vs FADD-RIPK1-DKO TRAIL+C+N	9,0	ns
TRADD-KO TRAIL+C+Z+N vs FADD-RIPK1-DKO TRAIL+C+Z+N	1,7	ns
TRADD-KO TRAIL+C+Z+N vs TRADD-RIPK1-DKO TRAIL	20	*
TRADD-KO TRAIL+C+Z+N vs TRADD-RIPK1-DKO TRAIL+Z	4,5	ns
TRADD-KO TRAIL+C+Z+N vs TRADD-RIPK1-DKO TRAIL+N	28	***
TRADD-KO TRAIL+C+Z+N vs TRADD-RIPK1-DKO TRAIL+Z+N	-1,8	ns
TRADD-KO TRAIL+C+Z+N vs TRADD-RIPK1-DKO TRAIL+C	84	***
TRADD-KO TRAIL+C+Z+N vs TRADD-RIPK1-DKO TRAIL+C+Z	-1,7	ns
TRADD-KO TRAIL+C+Z+N vs TRADD-RIPK1-DKO TRAIL+C+N	85	***
TRADD-KO TRAIL+C+Z+N vs TRADD-RIPK1-DKO TRAIL+C+Z+N	-0,65	ns
TRADD-KO TRAIL+C+Z+N vs Casp.8-KO TRAIL	11	ns
TRADD-KO TRAIL+C+Z+N vs Casp.8-KO TRAIL+Z	19	*
TRADD-KO TRAIL+C+Z+N vs Casp.8-KO TRAIL+N	-2,2	ns
TRADD-KO TRAIL+C+Z+N vs Casp.8-KO TRAIL+Z+N	-3,7	ns
TRADD-KO TRAIL+C+Z+N vs Casp.8-KO TRAIL+C	31	***
TRADD-KO TRAIL+C+Z+N vs Casp.8-KO TRAIL+C+Z	34	***
TRADD-KO TRAIL+C+Z+N vs Casp.8-KO TRAIL+C+N	4,9	ns
TRADD-KO TRAIL+C+Z+N vs Casp.8-KO TRAIL+C+Z+N	-0,16	ns
TRADD-KO TRAIL+C+Z+N vs EV TRAIL	-0,43	ns
TRADD-KO TRAIL+C+Z+N vs EV TRAIL+Z	-1,6	ns
TRADD-KO TRAIL+C+Z+N vs EV TRAIL+N	15	ns
TRADD-KO TRAIL+C+Z+N vs EV TRAIL+Z+N	3,4	ns
TRADD-KO TRAIL+C+Z+N vs EV TRAIL+C	76	***
TRADD-KO TRAIL+C+Z+N vs EV TRAIL+C+Z	0,016	ns
TRADD-KO TRAIL+C+Z+N vs EV TRAIL+C+N	79	***

TRADD-KO TRAIL+C+Z+N vs EV TRAIL+C+Z+N	3,2	ns
RIPK1-KO TRAIL vs RIPK1-KO TRAIL+Z	-9,9	ns
RIPK1-KO TRAIL vs RIPK1-KO TRAIL+N	9,5	ns
RIPK1-KO TRAIL vs RIPK1-KO TRAIL+Z+N	-7,2	ns
RIPK1-KO TRAIL vs RIPK1-KO TRAIL+C	77	***
RIPK1-KO TRAIL vs RIPK1-KO TRAIL+C+Z	-8,4	ns
RIPK1-KO TRAIL vs RIPK1-KO TRAIL+C+N	75	***
RIPK1-KO TRAIL vs RIPK1-KO TRAIL+C+Z+N	-8,3	ns
RIPK1-KO TRAIL vs FADD-TRADD-DKO TRAIL	5,9	ns
RIPK1-KO TRAIL vs FADD-TRADD-DKO TRAIL+Z	10	ns
RIPK1-KO TRAIL vs FADD-TRADD-DKO TRAIL+N	9,7	ns
RIPK1-KO TRAIL vs FADD-TRADD-DKO TRAIL+Z+N	-8,3	ns
RIPK1-KO TRAIL vs FADD-TRADD-DKO TRAIL+C	15	ns
RIPK1-KO TRAIL vs FADD-TRADD-DKO TRAIL+C+Z	-8,2	ns
RIPK1-KO TRAIL vs FADD-TRADD-DKO TRAIL+C+N	11	ns
RIPK1-KO TRAIL vs FADD-TRADD-DKO TRAIL+C+Z+N	-6,7	ns
RIPK1-KO TRAIL vs FADD-RIPK1-DKO TRAIL	-13	ns
RIPK1-KO TRAIL vs FADD-RIPK1-DKO TRAIL+Z	-11	ns
RIPK1-KO TRAIL vs FADD-RIPK1-DKO TRAIL+N	-5,8	ns
RIPK1-KO TRAIL vs FADD-RIPK1-DKO TRAIL+Z+N	-9,1	ns
RIPK1-KO TRAIL vs FADD-RIPK1-DKO TRAIL+C	-0,57	ns
RIPK1-KO TRAIL vs FADD-RIPK1-DKO TRAIL+C+Z	-1,2	ns
RIPK1-KO TRAIL vs FADD-RIPK1-DKO TRAIL+C+N	-0,85	ns
RIPK1-KO TRAIL vs FADD-RIPK1-DKO TRAIL+C+Z+N	-8,1	ns
RIPK1-KO TRAIL vs TRADD-RIPK1-DKO TRAIL	9,7	ns
RIPK1-KO TRAIL vs TRADD-RIPK1-DKO TRAIL+Z	-5,3	ns
RIPK1-KO TRAIL vs TRADD-RIPK1-DKO TRAIL+N	19	**
RIPK1-KO TRAIL vs TRADD-RIPK1-DKO TRAIL+Z+N	-12	ns
RIPK1-KO TRAIL vs TRADD-RIPK1-DKO TRAIL+C	74	***
RIPK1-KO TRAIL vs TRADD-RIPK1-DKO TRAIL+C+Z	-12	ns
RIPK1-KO TRAIL vs TRADD-RIPK1-DKO TRAIL+C+N	75	***
RIPK1-KO TRAIL vs TRADD-RIPK1-DKO TRAIL+C+Z+N	-11	ns
RIPK1-KO TRAIL vs Casp.8-KO TRAIL	0,64	ns
RIPK1-KO TRAIL vs Casp.8-KO TRAIL+Z	9,6	ns
RIPK1-KO TRAIL vs Casp.8-KO TRAIL+N	-12	ns
RIPK1-KO TRAIL vs Casp.8-KO TRAIL+Z+N	-14	ns
RIPK1-KO TRAIL vs Casp.8-KO TRAIL+C	22	***
RIPK1-KO TRAIL vs Casp.8-KO TRAIL+C+Z	24	***
RIPK1-KO TRAIL vs Casp.8-KO TRAIL+C+N	-5,0	ns
RIPK1-KO TRAIL vs Casp.8-KO TRAIL+C+Z+N	-10	ns
RIPK1-KO TRAIL vs EV TRAIL	-10	ns
RIPK1-KO TRAIL vs EV TRAIL+Z	-11	ns
RIPK1-KO TRAIL vs EV TRAIL+N	5,6	ns
RIPK1-KO TRAIL vs EV TRAIL+Z+N	-6,4	ns
RIPK1-KO TRAIL vs EV TRAIL+C	66	***
RIPK1-KO TRAIL vs EV TRAIL+C+Z	-9,9	ns
RIPK1-KO TRAIL vs EV TRAIL+C+N	69	***
RIPK1-KO TRAIL vs EV TRAIL+C+Z+N	-6,7	ns
RIPK1-KO TRAIL+Z vs RIPK1-KO TRAIL+N	19	***
RIPK1-KO TRAIL+Z vs RIPK1-KO TRAIL+Z+N	2,7	ns
RIPK1-KO TRAIL+Z vs RIPK1-KO TRAIL+C	87	***
RIPK1-KO TRAIL+Z vs RIPK1-KO TRAIL+C+Z	1,5	ns
RIPK1-KO TRAIL+Z vs RIPK1-KO TRAIL+C+N	85	***
RIPK1-KO TRAIL+Z vs RIPK1-KO TRAIL+C+Z+N	1,6	ns

RIPK1-KO TRAIL+Z vs FADD-TRADD-DKO TRAIL	16	ns
RIPK1-KO TRAIL+Z vs FADD-TRADD-DKO TRAIL+Z	20	***
RIPK1-KO TRAIL+Z vs FADD-TRADD-DKO TRAIL+N	20	**
RIPK1-KO TRAIL+Z vs FADD-TRADD-DKO TRAIL+Z+N	1,6	ns
RIPK1-KO TRAIL+Z vs FADD-TRADD-DKO TRAIL+C	25	***
RIPK1-KO TRAIL+Z vs FADD-TRADD-DKO TRAIL+C+Z	1,7	ns
RIPK1-KO TRAIL+Z vs FADD-TRADD-DKO TRAIL+C+N	21	**
RIPK1-KO TRAIL+Z vs FADD-TRADD-DKO TRAIL+C+Z+N	3,2	ns
RIPK1-KO TRAIL+Z vs FADD-RIPK1-DKO TRAIL	-2,7	ns
RIPK1-KO TRAIL+Z vs FADD-RIPK1-DKO TRAIL+Z	-1,1	ns
RIPK1-KO TRAIL+Z vs FADD-RIPK1-DKO TRAIL+N	4,1	ns
RIPK1-KO TRAIL+Z vs FADD-RIPK1-DKO TRAIL+Z+N	0,84	ns
RIPK1-KO TRAIL+Z vs FADD-RIPK1-DKO TRAIL+C	9,3	ns
RIPK1-KO TRAIL+Z vs FADD-RIPK1-DKO TRAIL+C+Z	8,7	ns
RIPK1-KO TRAIL+Z vs FADD-RIPK1-DKO TRAIL+C+N	9,1	ns
RIPK1-KO TRAIL+Z vs FADD-RIPK1-DKO TRAIL+C+Z+N	1,8	ns
RIPK1-KO TRAIL+Z vs TRADD-RIPK1-DKO TRAIL	20	***
RIPK1-KO TRAIL+Z vs TRADD-RIPK1-DKO TRAIL+Z	4,6	ns
RIPK1-KO TRAIL+Z vs TRADD-RIPK1-DKO TRAIL+N	28	***
RIPK1-KO TRAIL+Z vs TRADD-RIPK1-DKO TRAIL+Z+N	-1,7	ns
RIPK1-KO TRAIL+Z vs TRADD-RIPK1-DKO TRAIL+C	84	***
RIPK1-KO TRAIL+Z vs TRADD-RIPK1-DKO TRAIL+C+Z	-1,7	ns
RIPK1-KO TRAIL+Z vs TRADD-RIPK1-DKO TRAIL+C+N	85	***
RIPK1-KO TRAIL+Z vs TRADD-RIPK1-DKO TRAIL+C+Z+N	-0,62	ns
RIPK1-KO TRAIL+Z vs Casp.8-KO TRAIL	11	ns
RIPK1-KO TRAIL+Z vs Casp.8-KO TRAIL+Z	19	***
RIPK1-KO TRAIL+Z vs Casp.8-KO TRAIL+N	-2,1	ns
RIPK1-KO TRAIL+Z vs Casp.8-KO TRAIL+Z+N	-3,6	ns
RIPK1-KO TRAIL+Z vs Casp.8-KO TRAIL+C	31	***
RIPK1-KO TRAIL+Z vs Casp.8-KO TRAIL+C+Z	34	***
RIPK1-KO TRAIL+Z vs Casp.8-KO TRAIL+C+N	4,9	ns
RIPK1-KO TRAIL+Z vs Casp.8-KO TRAIL+C+Z+N	-0,13	ns
RIPK1-KO TRAIL+Z vs EV TRAIL	-0,41	ns
RIPK1-KO TRAIL+Z vs EV TRAIL+Z	-1,5	ns
RIPK1-KO TRAIL+Z vs EV TRAIL+N	15	ns
RIPK1-KO TRAIL+Z vs EV TRAIL+Z+N	3,5	ns
RIPK1-KO TRAIL+Z vs EV TRAIL+C	76	***
RIPK1-KO TRAIL+Z vs EV TRAIL+C+Z	0,038	ns
RIPK1-KO TRAIL+Z vs EV TRAIL+C+N	79	***
RIPK1-KO TRAIL+Z vs EV TRAIL+C+Z+N	3,2	ns
RIPK1-KO TRAIL+N vs RIPK1-KO TRAIL+Z+N	-17	*
RIPK1-KO TRAIL+N vs RIPK1-KO TRAIL+C	68	***
RIPK1-KO TRAIL+N vs RIPK1-KO TRAIL+C+Z	-18	*
RIPK1-KO TRAIL+N vs RIPK1-KO TRAIL+C+N	66	***
RIPK1-KO TRAIL+N vs RIPK1-KO TRAIL+C+Z+N	-18	*
RIPK1-KO TRAIL+N vs FADD-TRADD-DKO TRAIL	-3,6	ns
RIPK1-KO TRAIL+N vs FADD-TRADD-DKO TRAIL+Z	0,62	ns
RIPK1-KO TRAIL+N vs FADD-TRADD-DKO TRAIL+N	0,18	ns
RIPK1-KO TRAIL+N vs FADD-TRADD-DKO TRAIL+Z+N	-18	**
RIPK1-KO TRAIL+N vs FADD-TRADD-DKO TRAIL+C	5,5	ns
RIPK1-KO TRAIL+N vs FADD-TRADD-DKO TRAIL+C+Z	-18	ns
RIPK1-KO TRAIL+N vs FADD-TRADD-DKO TRAIL+C+N	1,9	ns
RIPK1-KO TRAIL+N vs FADD-TRADD-DKO TRAIL+C+Z+N	-16	ns
RIPK1-KO TRAIL+N vs FADD-RIPK1-DKO TRAIL	-22	***

RIPK1-KO TRAIL+N vs FADD-RIPK1-DKO TRAIL+Z	-20	***
RIPK1-KO TRAIL+N vs FADD-RIPK1-DKO TRAIL+N	-15	ns
RIPK1-KO TRAIL+N vs FADD-RIPK1-DKO TRAIL+Z+N	-19	***
RIPK1-KO TRAIL+N vs FADD-RIPK1-DKO TRAIL+C	-10	ns
RIPK1-KO TRAIL+N vs FADD-RIPK1-DKO TRAIL+C+Z	-11	ns
RIPK1-KO TRAIL+N vs FADD-RIPK1-DKO TRAIL+C+N	-10	ns
RIPK1-KO TRAIL+N vs FADD-RIPK1-DKO TRAIL+C+Z+N	-18	ns
RIPK1-KO TRAIL+N vs TRADD-RIPK1-DKO TRAIL	0,16	ns
RIPK1-KO TRAIL+N vs TRADD-RIPK1-DKO TRAIL+Z	-15	ns
RIPK1-KO TRAIL+N vs TRADD-RIPK1-DKO TRAIL+N	9,1	ns
RIPK1-KO TRAIL+N vs TRADD-RIPK1-DKO TRAIL+Z+N	-21	***
RIPK1-KO TRAIL+N vs TRADD-RIPK1-DKO TRAIL+C	65	***
RIPK1-KO TRAIL+N vs TRADD-RIPK1-DKO TRAIL+C+Z	-21	**
RIPK1-KO TRAIL+N vs TRADD-RIPK1-DKO TRAIL+C+N	66	***
RIPK1-KO TRAIL+N vs TRADD-RIPK1-DKO TRAIL+C+Z+N	-20	**
RIPK1-KO TRAIL+N vs Casp.8-KO TRAIL	-8,9	ns
RIPK1-KO TRAIL+N vs Casp.8-KO TRAIL+Z	0,077	ns
RIPK1-KO TRAIL+N vs Casp.8-KO TRAIL+N	-22	***
RIPK1-KO TRAIL+N vs Casp.8-KO TRAIL+Z+N	-23	***
RIPK1-KO TRAIL+N vs Casp.8-KO TRAIL+C	12	ns
RIPK1-KO TRAIL+N vs Casp.8-KO TRAIL+C+Z	15	ns
RIPK1-KO TRAIL+N vs Casp.8-KO TRAIL+C+N	-14	ns
RIPK1-KO TRAIL+N vs Casp.8-KO TRAIL+C+Z+N	-20	**
RIPK1-KO TRAIL+N vs EV TRAIL	-20	*
RIPK1-KO TRAIL+N vs EV TRAIL+Z	-21	*
RIPK1-KO TRAIL+N vs EV TRAIL+N	-3,9	ns
RIPK1-KO TRAIL+N vs EV TRAIL+Z+N	-16	ns
RIPK1-KO TRAIL+N vs EV TRAIL+C	56	***
RIPK1-KO TRAIL+N vs EV TRAIL+C+Z	-19	ns
RIPK1-KO TRAIL+N vs EV TRAIL+C+N	60	***
RIPK1-KO TRAIL+N vs EV TRAIL+C+Z+N	-16	ns
RIPK1-KO TRAIL+Z+N vs RIPK1-KO TRAIL+C	84	***
RIPK1-KO TRAIL+Z+N vs RIPK1-KO TRAIL+C+Z	-1,2	ns
RIPK1-KO TRAIL+Z+N vs RIPK1-KO TRAIL+C+N	83	***
RIPK1-KO TRAIL+Z+N vs RIPK1-KO TRAIL+C+Z+N	-1,1	ns
RIPK1-KO TRAIL+Z+N vs FADD-TRADD-DKO TRAIL	13	ns
RIPK1-KO TRAIL+Z+N vs FADD-TRADD-DKO TRAIL+Z	17	*
RIPK1-KO TRAIL+Z+N vs FADD-TRADD-DKO TRAIL+N	17	ns
RIPK1-KO TRAIL+Z+N vs FADD-TRADD-DKO TRAIL+Z+N	-1,1	ns
RIPK1-KO TRAIL+Z+N vs FADD-TRADD-DKO TRAIL+C	22	***
RIPK1-KO TRAIL+Z+N vs FADD-TRADD-DKO TRAIL+C+Z	-0,99	ns
RIPK1-KO TRAIL+Z+N vs FADD-TRADD-DKO TRAIL+C+N	19	ns
RIPK1-KO TRAIL+Z+N vs FADD-TRADD-DKO TRAIL+C+Z+N	0,51	ns
RIPK1-KO TRAIL+Z+N vs FADD-RIPK1-DKO TRAIL	-5,4	ns
RIPK1-KO TRAIL+Z+N vs FADD-RIPK1-DKO TRAIL+Z	-3,8	ns
RIPK1-KO TRAIL+Z+N vs FADD-RIPK1-DKO TRAIL+N	1,4	ns
RIPK1-KO TRAIL+Z+N vs FADD-RIPK1-DKO TRAIL+Z+N	-1,8	ns
RIPK1-KO TRAIL+Z+N vs FADD-RIPK1-DKO TRAIL+C	6,7	ns
RIPK1-KO TRAIL+Z+N vs FADD-RIPK1-DKO TRAIL+C+Z	6,1	ns
RIPK1-KO TRAIL+Z+N vs FADD-RIPK1-DKO TRAIL+C+N	6,4	ns
RIPK1-KO TRAIL+Z+N vs FADD-RIPK1-DKO TRAIL+C+Z+N	-0,89	ns
RIPK1-KO TRAIL+Z+N vs TRADD-RIPK1-DKO TRAIL	17	*
RIPK1-KO TRAIL+Z+N vs TRADD-RIPK1-DKO TRAIL+Z	1,9	ns
RIPK1-KO TRAIL+Z+N vs TRADD-RIPK1-DKO TRAIL+N	26	***

RIPK1-KO TRAIL+Z+N vs TRADD-RIPK1-DKO TRAIL+Z+N	-4,4	ns
RIPK1-KO TRAIL+Z+N vs TRADD-RIPK1-DKO TRAIL+C	82	***
RIPK1-KO TRAIL+Z+N vs TRADD-RIPK1-DKO TRAIL+C+Z	-4,3	ns
RIPK1-KO TRAIL+Z+N vs TRADD-RIPK1-DKO TRAIL+C+N	82	***
RIPK1-KO TRAIL+Z+N vs TRADD-RIPK1-DKO TRAIL+C+Z+N	-3,3	ns
RIPK1-KO TRAIL+Z+N vs Casp.8-KO TRAIL	7,9	ns
RIPK1-KO TRAIL+Z+N vs Casp.8-KO TRAIL+Z	17	*
RIPK1-KO TRAIL+Z+N vs Casp.8-KO TRAIL+N	-4,8	ns
RIPK1-KO TRAIL+Z+N vs Casp.8-KO TRAIL+Z+N	-6,3	ns
RIPK1-KO TRAIL+Z+N vs Casp.8-KO TRAIL+C	29	***
RIPK1-KO TRAIL+Z+N vs Casp.8-KO TRAIL+C+Z	32	***
RIPK1-KO TRAIL+Z+N vs Casp.8-KO TRAIL+C+N	2,3	ns
RIPK1-KO TRAIL+Z+N vs Casp.8-KO TRAIL+C+Z+N	-2,8	ns
RIPK1-KO TRAIL+Z+N vs EV TRAIL	-3,1	ns
RIPK1-KO TRAIL+Z+N vs EV TRAIL+Z	-4,2	ns
RIPK1-KO TRAIL+Z+N vs EV TRAIL+N	13	ns
RIPK1-KO TRAIL+Z+N vs EV TRAIL+Z+N	0,81	ns
RIPK1-KO TRAIL+Z+N vs EV TRAIL+C	73	***
RIPK1-KO TRAIL+Z+N vs EV TRAIL+C+Z	-2,6	ns
RIPK1-KO TRAIL+Z+N vs EV TRAIL+C+N	76	***
RIPK1-KO TRAIL+Z+N vs EV TRAIL+C+Z+N	0,56	ns
RIPK1-KO TRAIL+C vs RIPK1-KO TRAIL+C+Z	-86	***
RIPK1-KO TRAIL+C vs RIPK1-KO TRAIL+C+N	-1,8	ns
RIPK1-KO TRAIL+C vs RIPK1-KO TRAIL+C+Z+N	-85	***
RIPK1-KO TRAIL+C vs FADD-TRADD-DKO TRAIL	-71	***
RIPK1-KO TRAIL+C vs FADD-TRADD-DKO TRAIL+Z	-67	***
RIPK1-KO TRAIL+C vs FADD-TRADD-DKO TRAIL+N	-67	***
RIPK1-KO TRAIL+C vs FADD-TRADD-DKO TRAIL+Z+N	-85	***
RIPK1-KO TRAIL+C vs FADD-TRADD-DKO TRAIL+C	-62	***
RIPK1-KO TRAIL+C vs FADD-TRADD-DKO TRAIL+C+Z	-85	***
RIPK1-KO TRAIL+C vs FADD-TRADD-DKO TRAIL+C+N	-66	***
RIPK1-KO TRAIL+C vs FADD-TRADD-DKO TRAIL+C+Z+N	-84	***
RIPK1-KO TRAIL+C vs FADD-RIPK1-DKO TRAIL	-90	***
RIPK1-KO TRAIL+C vs FADD-RIPK1-DKO TRAIL+Z	-88	***
RIPK1-KO TRAIL+C vs FADD-RIPK1-DKO TRAIL+N	-83	***
RIPK1-KO TRAIL+C vs FADD-RIPK1-DKO TRAIL+Z+N	-86	***
RIPK1-KO TRAIL+C vs FADD-RIPK1-DKO TRAIL+C	-78	***
RIPK1-KO TRAIL+C vs FADD-RIPK1-DKO TRAIL+C+Z	-78	***
RIPK1-KO TRAIL+C vs FADD-RIPK1-DKO TRAIL+C+N	-78	***
RIPK1-KO TRAIL+C vs FADD-RIPK1-DKO TRAIL+C+Z+N	-85	***
RIPK1-KO TRAIL+C vs TRADD-RIPK1-DKO TRAIL	-67	***
RIPK1-KO TRAIL+C vs TRADD-RIPK1-DKO TRAIL+Z	-82	***
RIPK1-KO TRAIL+C vs TRADD-RIPK1-DKO TRAIL+N	-59	***
RIPK1-KO TRAIL+C vs TRADD-RIPK1-DKO TRAIL+Z+N	-89	***
RIPK1-KO TRAIL+C vs TRADD-RIPK1-DKO TRAIL+C	-2,8	ns
RIPK1-KO TRAIL+C vs TRADD-RIPK1-DKO TRAIL+C+Z	-89	***
RIPK1-KO TRAIL+C vs TRADD-RIPK1-DKO TRAIL+C+N	-2,1	ns
RIPK1-KO TRAIL+C vs TRADD-RIPK1-DKO TRAIL+C+Z+N	-88	***
RIPK1-KO TRAIL+C vs Casp.8-KO TRAIL	-77	***
RIPK1-KO TRAIL+C vs Casp.8-KO TRAIL+Z	-68	***
RIPK1-KO TRAIL+C vs Casp.8-KO TRAIL+N	-89	***
RIPK1-KO TRAIL+C vs Casp.8-KO TRAIL+Z+N	-91	***
RIPK1-KO TRAIL+C vs Casp.8-KO TRAIL+C	-56	***
RIPK1-KO TRAIL+C vs Casp.8-KO TRAIL+C+Z	-53	***

RIPK1-KO TRAIL+C vs Casp.8-KO TRAIL+C+N	-82	***
RIPK1-KO TRAIL+C vs Casp.8-KO TRAIL+C+Z+N	-87	***
RIPK1-KO TRAIL+C vs EV TRAIL	-87	***
RIPK1-KO TRAIL+C vs EV TRAIL+Z	-89	***
RIPK1-KO TRAIL+C vs EV TRAIL+N	-72	***
RIPK1-KO TRAIL+C vs EV TRAIL+Z+N	-84	***
RIPK1-KO TRAIL+C vs EV TRAIL+C	-11	ns
RIPK1-KO TRAIL+C vs EV TRAIL+C+Z	-87	***
RIPK1-KO TRAIL+C vs EV TRAIL+C+N	-8,1	ns
RIPK1-KO TRAIL+C vs EV TRAIL+C+Z+N	-84	***
RIPK1-KO TRAIL+C+Z vs RIPK1-KO TRAIL+C+N	84	***
RIPK1-KO TRAIL+C+Z vs RIPK1-KO TRAIL+C+Z+N	0,091	ns
RIPK1-KO TRAIL+C+Z vs FADD-TRADD-DKO TRAIL	14	ns
RIPK1-KO TRAIL+C+Z vs FADD-TRADD-DKO TRAIL+Z	19	*
RIPK1-KO TRAIL+C+Z vs FADD-TRADD-DKO TRAIL+N	18	*
RIPK1-KO TRAIL+C+Z vs FADD-TRADD-DKO TRAIL+Z+N	0,13	ns
RIPK1-KO TRAIL+C+Z vs FADD-TRADD-DKO TRAIL+C	23	***
RIPK1-KO TRAIL+C+Z vs FADD-TRADD-DKO TRAIL+C+Z	0,20	ns
RIPK1-KO TRAIL+C+Z vs FADD-TRADD-DKO TRAIL+C+N	20	ns
RIPK1-KO TRAIL+C+Z vs FADD-TRADD-DKO TRAIL+C+Z+N	1,7	ns
RIPK1-KO TRAIL+C+Z vs FADD-RIPK1-DKO TRAIL	-4,2	ns
RIPK1-KO TRAIL+C+Z vs FADD-RIPK1-DKO TRAIL+Z	-2,6	ns
RIPK1-KO TRAIL+C+Z vs FADD-RIPK1-DKO TRAIL+N	2,6	ns
RIPK1-KO TRAIL+C+Z vs FADD-RIPK1-DKO TRAIL+Z+N	-0,63	ns
RIPK1-KO TRAIL+C+Z vs FADD-RIPK1-DKO TRAIL+C	7,9	ns
RIPK1-KO TRAIL+C+Z vs FADD-RIPK1-DKO TRAIL+C+Z	7,3	ns
RIPK1-KO TRAIL+C+Z vs FADD-RIPK1-DKO TRAIL+C+N	7,6	ns
RIPK1-KO TRAIL+C+Z vs FADD-RIPK1-DKO TRAIL+C+Z+N	0,30	ns
RIPK1-KO TRAIL+C+Z vs TRADD-RIPK1-DKO TRAIL	18	*
RIPK1-KO TRAIL+C+Z vs TRADD-RIPK1-DKO TRAIL+Z	3,1	ns
RIPK1-KO TRAIL+C+Z vs TRADD-RIPK1-DKO TRAIL+N	27	***
RIPK1-KO TRAIL+C+Z vs TRADD-RIPK1-DKO TRAIL+Z+N	-3,2	ns
RIPK1-KO TRAIL+C+Z vs TRADD-RIPK1-DKO TRAIL+C	83	***
RIPK1-KO TRAIL+C+Z vs TRADD-RIPK1-DKO TRAIL+C+Z	-3,1	ns
RIPK1-KO TRAIL+C+Z vs TRADD-RIPK1-DKO TRAIL+C+N	84	***
RIPK1-KO TRAIL+C+Z vs TRADD-RIPK1-DKO TRAIL+C+Z+N	-2,1	ns
RIPK1-KO TRAIL+C+Z vs Casp.8-KO TRAIL	9,1	ns
RIPK1-KO TRAIL+C+Z vs Casp.8-KO TRAIL+Z	18	*
RIPK1-KO TRAIL+C+Z vs Casp.8-KO TRAIL+N	-3,6	ns
RIPK1-KO TRAIL+C+Z vs Casp.8-KO TRAIL+Z+N	-5,1	ns
RIPK1-KO TRAIL+C+Z vs Casp.8-KO TRAIL+C	30	***
RIPK1-KO TRAIL+C+Z vs Casp.8-KO TRAIL+C+Z	33	***
RIPK1-KO TRAIL+C+Z vs Casp.8-KO TRAIL+C+N	3,5	ns
RIPK1-KO TRAIL+C+Z vs Casp.8-KO TRAIL+C+Z+N	-1,6	ns
RIPK1-KO TRAIL+C+Z vs EV TRAIL	-1,9	ns
RIPK1-KO TRAIL+C+Z vs EV TRAIL+Z	-3,0	ns
RIPK1-KO TRAIL+C+Z vs EV TRAIL+N	14	ns
RIPK1-KO TRAIL+C+Z vs EV TRAIL+Z+N	2,0	ns
RIPK1-KO TRAIL+C+Z vs EV TRAIL+C	74	***
RIPK1-KO TRAIL+C+Z vs EV TRAIL+C+Z	-1,4	ns
RIPK1-KO TRAIL+C+Z vs EV TRAIL+C+N	78	***
RIPK1-KO TRAIL+C+Z vs EV TRAIL+C+Z+N	1,8	ns
RIPK1-KO TRAIL+C+N vs RIPK1-KO TRAIL+C+Z+N	-84	***
RIPK1-KO TRAIL+C+N vs FADD-TRADD-DKO TRAIL	-70	***

RIPK1-KO TRAIL+C+N vs FADD-TRADD-DKO TRAIL+Z	-65	***
RIPK1-KO TRAIL+C+N vs FADD-TRADD-DKO TRAIL+N	-66	***
RIPK1-KO TRAIL+C+N vs FADD-TRADD-DKO TRAIL+Z+N	-84	***
RIPK1-KO TRAIL+C+N vs FADD-TRADD-DKO TRAIL+C	-60	***
RIPK1-KO TRAIL+C+N vs FADD-TRADD-DKO TRAIL+C+Z	-84	***
RIPK1-KO TRAIL+C+N vs FADD-TRADD-DKO TRAIL+C+N	-64	***
RIPK1-KO TRAIL+C+N vs FADD-TRADD-DKO TRAIL+C+Z+N	-82	***
RIPK1-KO TRAIL+C+N vs FADD-RIPK1-DKO TRAIL	-88	***
RIPK1-KO TRAIL+C+N vs FADD-RIPK1-DKO TRAIL+Z	-86	***
RIPK1-KO TRAIL+C+N vs FADD-RIPK1-DKO TRAIL+N	-81	***
RIPK1-KO TRAIL+C+N vs FADD-RIPK1-DKO TRAIL+Z+N	-84	***
RIPK1-KO TRAIL+C+N vs FADD-RIPK1-DKO TRAIL+C	-76	***
RIPK1-KO TRAIL+C+N vs FADD-RIPK1-DKO TRAIL+C+Z	-77	***
RIPK1-KO TRAIL+C+N vs FADD-RIPK1-DKO TRAIL+C+N	-76	***
RIPK1-KO TRAIL+C+N vs FADD-RIPK1-DKO TRAIL+C+Z+N	-84	***
RIPK1-KO TRAIL+C+N vs TRADD-RIPK1-DKO TRAIL	-66	***
RIPK1-KO TRAIL+C+N vs TRADD-RIPK1-DKO TRAIL+Z	-81	***
RIPK1-KO TRAIL+C+N vs TRADD-RIPK1-DKO TRAIL+N	-57	***
RIPK1-KO TRAIL+C+N vs TRADD-RIPK1-DKO TRAIL+Z+N	-87	***
RIPK1-KO TRAIL+C+N vs TRADD-RIPK1-DKO TRAIL+C	-1,1	ns
RIPK1-KO TRAIL+C+N vs TRADD-RIPK1-DKO TRAIL+C+Z	-87	***
RIPK1-KO TRAIL+C+N vs TRADD-RIPK1-DKO TRAIL+C+N	-0,30	ns
RIPK1-KO TRAIL+C+N vs TRADD-RIPK1-DKO TRAIL+C+Z+N	-86	***
RIPK1-KO TRAIL+C+N vs Casp.8-KO TRAIL	-75	***
RIPK1-KO TRAIL+C+N vs Casp.8-KO TRAIL+Z	-66	***
RIPK1-KO TRAIL+C+N vs Casp.8-KO TRAIL+N	-87	***
RIPK1-KO TRAIL+C+N vs Casp.8-KO TRAIL+Z+N	-89	***
RIPK1-KO TRAIL+C+N vs Casp.8-KO TRAIL+C	-54	***
RIPK1-KO TRAIL+C+N vs Casp.8-KO TRAIL+C+Z	-51	***
RIPK1-KO TRAIL+C+N vs Casp.8-KO TRAIL+C+N	-80	***
RIPK1-KO TRAIL+C+N vs Casp.8-KO TRAIL+C+Z+N	-85	***
RIPK1-KO TRAIL+C+N vs EV TRAIL	-86	***
RIPK1-KO TRAIL+C+N vs EV TRAIL+Z	-87	***
RIPK1-KO TRAIL+C+N vs EV TRAIL+N	-70	***
RIPK1-KO TRAIL+C+N vs EV TRAIL+Z+N	-82	***
RIPK1-KO TRAIL+C+N vs EV TRAIL+C	-9,5	ns
RIPK1-KO TRAIL+C+N vs EV TRAIL+C+Z	-85	***
RIPK1-KO TRAIL+C+N vs EV TRAIL+C+N	-6,3	ns
RIPK1-KO TRAIL+C+N vs EV TRAIL+C+Z+N	-82	***
RIPK1-KO TRAIL+C+Z+N vs FADD-TRADD-DKO TRAIL	14	ns
RIPK1-KO TRAIL+C+Z+N vs FADD-TRADD-DKO TRAIL+Z	18	*
RIPK1-KO TRAIL+C+Z+N vs FADD-TRADD-DKO TRAIL+N	18	ns
RIPK1-KO TRAIL+C+Z+N vs FADD-TRADD-DKO TRAIL+Z+N	0,042	ns
RIPK1-KO TRAIL+C+Z+N vs FADD-TRADD-DKO TRAIL+C	23	***
RIPK1-KO TRAIL+C+Z+N vs FADD-TRADD-DKO TRAIL+C+Z	0,11	ns
RIPK1-KO TRAIL+C+Z+N vs FADD-TRADD-DKO TRAIL+C+N	20	ns
RIPK1-KO TRAIL+C+Z+N vs FADD-TRADD-DKO TRAIL+C+Z+N	1,6	ns
RIPK1-KO TRAIL+C+Z+N vs FADD-RIPK1-DKO TRAIL	-4,3	ns
RIPK1-KO TRAIL+C+Z+N vs FADD-RIPK1-DKO TRAIL+Z	-2,7	ns
RIPK1-KO TRAIL+C+Z+N vs FADD-RIPK1-DKO TRAIL+N	2,5	ns
RIPK1-KO TRAIL+C+Z+N vs FADD-RIPK1-DKO TRAIL+Z+N	-0,72	ns
RIPK1-KO TRAIL+C+Z+N vs FADD-RIPK1-DKO TRAIL+C	7,8	ns
RIPK1-KO TRAIL+C+Z+N vs FADD-RIPK1-DKO TRAIL+C+Z	7,2	ns
RIPK1-KO TRAIL+C+Z+N vs FADD-RIPK1-DKO TRAIL+C+N	7,5	ns

RIPK1-KO TRAIL+C+Z+N vs FADD-RIPK1-DKO TRAIL+C+Z+N	0,21	ns
RIPK1-KO TRAIL+C+Z+N vs TRADD-RIPK1-DKO TRAIL	18	*
RIPK1-KO TRAIL+C+Z+N vs TRADD-RIPK1-DKO TRAIL+Z	3,0	ns
RIPK1-KO TRAIL+C+Z+N vs TRADD-RIPK1-DKO TRAIL+N	27	***
RIPK1-KO TRAIL+C+Z+N vs TRADD-RIPK1-DKO TRAIL+Z+N	-3,3	ns
RIPK1-KO TRAIL+C+Z+N vs TRADD-RIPK1-DKO TRAIL+C	83	***
RIPK1-KO TRAIL+C+Z+N vs TRADD-RIPK1-DKO TRAIL+C+Z	-3,2	ns
RIPK1-KO TRAIL+C+Z+N vs TRADD-RIPK1-DKO TRAIL+C+N	83	***
RIPK1-KO TRAIL+C+Z+N vs TRADD-RIPK1-DKO TRAIL+C+Z+N	-2,2	ns
RIPK1-KO TRAIL+C+Z+N vs Casp.8-KO TRAIL	9,0	ns
RIPK1-KO TRAIL+C+Z+N vs Casp.8-KO TRAIL+Z	18	*
RIPK1-KO TRAIL+C+Z+N vs Casp.8-KO TRAIL+N	-3,7	ns
RIPK1-KO TRAIL+C+Z+N vs Casp.8-KO TRAIL+Z+N	-5,2	ns
RIPK1-KO TRAIL+C+Z+N vs Casp.8-KO TRAIL+C	30	***
RIPK1-KO TRAIL+C+Z+N vs Casp.8-KO TRAIL+C+Z	33	***
RIPK1-KO TRAIL+C+Z+N vs Casp.8-KO TRAIL+C+N	3,4	ns
RIPK1-KO TRAIL+C+Z+N vs Casp.8-KO TRAIL+C+Z+N	-1,7	ns
RIPK1-KO TRAIL+C+Z+N vs EV TRAIL	-2,0	ns
RIPK1-KO TRAIL+C+Z+N vs EV TRAIL+Z	-3,1	ns
RIPK1-KO TRAIL+C+Z+N vs EV TRAIL+N	14	ns
RIPK1-KO TRAIL+C+Z+N vs EV TRAIL+Z+N	1,9	ns
RIPK1-KO TRAIL+C+Z+N vs EV TRAIL+C	74	***
RIPK1-KO TRAIL+C+Z+N vs EV TRAIL+C+Z	-1,5	ns
RIPK1-KO TRAIL+C+Z+N vs EV TRAIL+C+N	77	***
RIPK1-KO TRAIL+C+Z+N vs EV TRAIL+C+Z+N	1,7	ns
FADD-TRADD-DKO TRAIL vs FADD-TRADD-DKO TRAIL+Z	4,2	ns
FADD-TRADD-DKO TRAIL vs FADD-TRADD-DKO TRAIL+N	3,8	ns
FADD-TRADD-DKO TRAIL vs FADD-TRADD-DKO TRAIL+Z+N	-14	ns
FADD-TRADD-DKO TRAIL vs FADD-TRADD-DKO TRAIL+C	9,1	ns
FADD-TRADD-DKO TRAIL vs FADD-TRADD-DKO TRAIL+C+Z	-14	ns
FADD-TRADD-DKO TRAIL vs FADD-TRADD-DKO TRAIL+C+N	5,6	ns
FADD-TRADD-DKO TRAIL vs FADD-TRADD-DKO TRAIL+C+Z+N	-13	ns
FADD-TRADD-DKO TRAIL vs FADD-RIPK1-DKO TRAIL	-18	*
FADD-TRADD-DKO TRAIL vs FADD-RIPK1-DKO TRAIL+Z	-17	ns
FADD-TRADD-DKO TRAIL vs FADD-RIPK1-DKO TRAIL+N	-12	ns
FADD-TRADD-DKO TRAIL vs FADD-RIPK1-DKO TRAIL+Z+N	-15	ns
FADD-TRADD-DKO TRAIL vs FADD-RIPK1-DKO TRAIL+C	-6,4	ns
FADD-TRADD-DKO TRAIL vs FADD-RIPK1-DKO TRAIL+C+Z	-7,0	ns
FADD-TRADD-DKO TRAIL vs FADD-RIPK1-DKO TRAIL+C+N	-6,7	ns
FADD-TRADD-DKO TRAIL vs FADD-RIPK1-DKO TRAIL+C+Z+N	-14	ns
FADD-TRADD-DKO TRAIL vs TRADD-RIPK1-DKO TRAIL	3,8	ns
FADD-TRADD-DKO TRAIL vs TRADD-RIPK1-DKO TRAIL+Z	-11	ns
FADD-TRADD-DKO TRAIL vs TRADD-RIPK1-DKO TRAIL+N	13	ns
FADD-TRADD-DKO TRAIL vs TRADD-RIPK1-DKO TRAIL+Z+N	-18	ns
FADD-TRADD-DKO TRAIL vs TRADD-RIPK1-DKO TRAIL+C	68	***
FADD-TRADD-DKO TRAIL vs TRADD-RIPK1-DKO TRAIL+C+Z	-17	ns
FADD-TRADD-DKO TRAIL vs TRADD-RIPK1-DKO TRAIL+C+N	69	***
FADD-TRADD-DKO TRAIL vs TRADD-RIPK1-DKO TRAIL+C+Z+N	-16	ns
FADD-TRADD-DKO TRAIL vs Casp.8-KO TRAIL	-5,2	ns
FADD-TRADD-DKO TRAIL vs Casp.8-KO TRAIL+Z	3,7	ns
FADD-TRADD-DKO TRAIL vs Casp.8-KO TRAIL+N	-18	ns
FADD-TRADD-DKO TRAIL vs Casp.8-KO TRAIL+Z+N	-19	*
FADD-TRADD-DKO TRAIL vs Casp.8-KO TRAIL+C	16	ns
FADD-TRADD-DKO TRAIL vs Casp.8-KO TRAIL+C+Z	18	ns

FADD-TRADD-DKO TRAIL vs Casp.8-KO TRAIL+C+N	-11	ns
FADD-TRADD-DKO TRAIL vs Casp.8-KO TRAIL+C+Z+N	-16	ns
FADD-TRADD-DKO TRAIL vs EV TRAIL	-16	ns
FADD-TRADD-DKO TRAIL vs EV TRAIL+Z	-17	ns
FADD-TRADD-DKO TRAIL vs EV TRAIL+N	-0,30	ns
FADD-TRADD-DKO TRAIL vs EV TRAIL+Z+N	-12	ns
FADD-TRADD-DKO TRAIL vs EV TRAIL+C	60	***
FADD-TRADD-DKO TRAIL vs EV TRAIL+C+Z	-16	ns
FADD-TRADD-DKO TRAIL vs EV TRAIL+C+N	63	***
FADD-TRADD-DKO TRAIL vs EV TRAIL+C+Z+N	-13	ns
FADD-TRADD-DKO TRAIL+Z vs FADD-TRADD-DKO TRAIL+N	-0,45	ns
FADD-TRADD-DKO TRAIL+Z vs FADD-TRADD-DKO TRAIL+Z+N	-18	**
FADD-TRADD-DKO TRAIL+Z vs FADD-TRADD-DKO TRAIL+C	4,9	ns
FADD-TRADD-DKO TRAIL+Z vs FADD-TRADD-DKO TRAIL+C+Z	-18	ns
FADD-TRADD-DKO TRAIL+Z vs FADD-TRADD-DKO TRAIL+C+N	1,3	ns
FADD-TRADD-DKO TRAIL+Z vs FADD-TRADD-DKO TRAIL+C+Z+N	-17	ns
FADD-TRADD-DKO TRAIL+Z vs FADD-RIPK1-DKO TRAIL	-23	***
FADD-TRADD-DKO TRAIL+Z vs FADD-RIPK1-DKO TRAIL+Z	-21	***
FADD-TRADD-DKO TRAIL+Z vs FADD-RIPK1-DKO TRAIL+N	-16	ns
FADD-TRADD-DKO TRAIL+Z vs FADD-RIPK1-DKO TRAIL+Z+N	-19	***
FADD-TRADD-DKO TRAIL+Z vs FADD-RIPK1-DKO TRAIL+C	-11	ns
FADD-TRADD-DKO TRAIL+Z vs FADD-RIPK1-DKO TRAIL+C+Z	-11	ns
FADD-TRADD-DKO TRAIL+Z vs FADD-RIPK1-DKO TRAIL+C+N	-11	ns
FADD-TRADD-DKO TRAIL+Z vs FADD-RIPK1-DKO TRAIL+C+Z+N	-18	ns
FADD-TRADD-DKO TRAIL+Z vs TRADD-RIPK1-DKO TRAIL	-0,46	ns
FADD-TRADD-DKO TRAIL+Z vs TRADD-RIPK1-DKO TRAIL+Z	-15	ns
FADD-TRADD-DKO TRAIL+Z vs TRADD-RIPK1-DKO TRAIL+N	8,5	ns
FADD-TRADD-DKO TRAIL+Z vs TRADD-RIPK1-DKO TRAIL+Z+N	-22	**
FADD-TRADD-DKO TRAIL+Z vs TRADD-RIPK1-DKO TRAIL+C	64	***
FADD-TRADD-DKO TRAIL+Z vs TRADD-RIPK1-DKO TRAIL+C+Z	-22	**
FADD-TRADD-DKO TRAIL+Z vs TRADD-RIPK1-DKO TRAIL+C+N	65	***
FADD-TRADD-DKO TRAIL+Z vs TRADD-RIPK1-DKO TRAIL+C+Z+N	-21	**
FADD-TRADD-DKO TRAIL+Z vs Casp.8-KO TRAIL	-9,5	ns
FADD-TRADD-DKO TRAIL+Z vs Casp.8-KO TRAIL+Z	-0,55	ns
FADD-TRADD-DKO TRAIL+Z vs Casp.8-KO TRAIL+N	-22	***
FADD-TRADD-DKO TRAIL+Z vs Casp.8-KO TRAIL+Z+N	-24	***
FADD-TRADD-DKO TRAIL+Z vs Casp.8-KO TRAIL+C	11	ns
FADD-TRADD-DKO TRAIL+Z vs Casp.8-KO TRAIL+C+Z	14	ns
FADD-TRADD-DKO TRAIL+Z vs Casp.8-KO TRAIL+C+N	-15	ns
FADD-TRADD-DKO TRAIL+Z vs Casp.8-KO TRAIL+C+Z+N	-20	**
FADD-TRADD-DKO TRAIL+Z vs EV TRAIL	-20	*
FADD-TRADD-DKO TRAIL+Z vs EV TRAIL+Z	-22	*
FADD-TRADD-DKO TRAIL+Z vs EV TRAIL+N	-4,5	ns
FADD-TRADD-DKO TRAIL+Z vs EV TRAIL+Z+N	-17	ns
FADD-TRADD-DKO TRAIL+Z vs EV TRAIL+C	56	***
FADD-TRADD-DKO TRAIL+Z vs EV TRAIL+C+Z	-20	ns
FADD-TRADD-DKO TRAIL+Z vs EV TRAIL+C+N	59	***
FADD-TRADD-DKO TRAIL+Z vs EV TRAIL+C+Z+N	-17	ns
FADD-TRADD-DKO TRAIL+N vs FADD-TRADD-DKO TRAIL+Z+N	-18	*
FADD-TRADD-DKO TRAIL+N vs FADD-TRADD-DKO TRAIL+C	5,3	ns
FADD-TRADD-DKO TRAIL+N vs FADD-TRADD-DKO TRAIL+C+Z	-18	ns
FADD-TRADD-DKO TRAIL+N vs FADD-TRADD-DKO TRAIL+C+N	1,8	ns
FADD-TRADD-DKO TRAIL+N vs FADD-TRADD-DKO TRAIL+C+Z+N	-16	ns
FADD-TRADD-DKO TRAIL+N vs FADD-RIPK1-DKO TRAIL	-22	***

FADD-TRADD-DKO TRAIL+N vs FADD-RIPK1-DKO TRAIL+Z	-21	***
FADD-TRADD-DKO TRAIL+N vs FADD-RIPK1-DKO TRAIL+N	-15	ns
FADD-TRADD-DKO TRAIL+N vs FADD-RIPK1-DKO TRAIL+Z+N	-19	**
FADD-TRADD-DKO TRAIL+N vs FADD-RIPK1-DKO TRAIL+C	-10	ns
FADD-TRADD-DKO TRAIL+N vs FADD-RIPK1-DKO TRAIL+C+Z	-11	ns
FADD-TRADD-DKO TRAIL+N vs FADD-RIPK1-DKO TRAIL+C+N	-11	ns
FADD-TRADD-DKO TRAIL+N vs FADD-RIPK1-DKO TRAIL+C+Z+N	-18	ns
FADD-TRADD-DKO TRAIL+N vs TRADD-RIPK1-DKO TRAIL	-0,017	ns
FADD-TRADD-DKO TRAIL+N vs TRADD-RIPK1-DKO TRAIL+Z	-15	ns
FADD-TRADD-DKO TRAIL+N vs TRADD-RIPK1-DKO TRAIL+N	8,9	ns
FADD-TRADD-DKO TRAIL+N vs TRADD-RIPK1-DKO TRAIL+Z+N	-21	**
FADD-TRADD-DKO TRAIL+N vs TRADD-RIPK1-DKO TRAIL+C	65	***
FADD-TRADD-DKO TRAIL+N vs TRADD-RIPK1-DKO TRAIL+C+Z	-21	*
FADD-TRADD-DKO TRAIL+N vs TRADD-RIPK1-DKO TRAIL+C+N	65	***
FADD-TRADD-DKO TRAIL+N vs TRADD-RIPK1-DKO TRAIL+C+Z+N	-20	**
FADD-TRADD-DKO TRAIL+N vs Casp.8-KO TRAIL	-9,0	ns
FADD-TRADD-DKO TRAIL+N vs Casp.8-KO TRAIL+Z	-0,10	ns
FADD-TRADD-DKO TRAIL+N vs Casp.8-KO TRAIL+N	-22	**
FADD-TRADD-DKO TRAIL+N vs Casp.8-KO TRAIL+Z+N	-23	***
FADD-TRADD-DKO TRAIL+N vs Casp.8-KO TRAIL+C	12	ns
FADD-TRADD-DKO TRAIL+N vs Casp.8-KO TRAIL+C+Z	15	ns
FADD-TRADD-DKO TRAIL+N vs Casp.8-KO TRAIL+C+N	-15	ns
FADD-TRADD-DKO TRAIL+N vs Casp.8-KO TRAIL+C+Z+N	-20	*
FADD-TRADD-DKO TRAIL+N vs EV TRAIL	-20	*
FADD-TRADD-DKO TRAIL+N vs EV TRAIL+Z	-21	ns
FADD-TRADD-DKO TRAIL+N vs EV TRAIL+N	-4,1	ns
FADD-TRADD-DKO TRAIL+N vs EV TRAIL+Z+N	-16	ns
FADD-TRADD-DKO TRAIL+N vs EV TRAIL+C	56	***
FADD-TRADD-DKO TRAIL+N vs EV TRAIL+C+Z	-20	ns
FADD-TRADD-DKO TRAIL+N vs EV TRAIL+C+N	59	***
FADD-TRADD-DKO TRAIL+N vs EV TRAIL+C+Z+N	-16	ns
FADD-TRADD-DKO TRAIL+Z+N vs FADD-TRADD-DKO TRAIL+C	23	***
FADD-TRADD-DKO TRAIL+Z+N vs FADD-TRADD-DKO TRAIL+C+Z	0,067	ns
FADD-TRADD-DKO TRAIL+Z+N vs FADD-TRADD-DKO TRAIL+C+N	20	*
FADD-TRADD-DKO TRAIL+Z+N vs FADD-TRADD-DKO TRAIL+C+Z+N	1,6	ns
FADD-TRADD-DKO TRAIL+Z+N vs FADD-RIPK1-DKO TRAIL	-4,3	ns
FADD-TRADD-DKO TRAIL+Z+N vs FADD-RIPK1-DKO TRAIL+Z	-2,7	ns
FADD-TRADD-DKO TRAIL+Z+N vs FADD-RIPK1-DKO TRAIL+N	2,5	ns
FADD-TRADD-DKO TRAIL+Z+N vs FADD-RIPK1-DKO TRAIL+Z+N	-0,76	ns
FADD-TRADD-DKO TRAIL+Z+N vs FADD-RIPK1-DKO TRAIL+C	7,7	ns
FADD-TRADD-DKO TRAIL+Z+N vs FADD-RIPK1-DKO TRAIL+C+Z	7,1	ns
FADD-TRADD-DKO TRAIL+Z+N vs FADD-RIPK1-DKO TRAIL+C+N	7,5	ns
FADD-TRADD-DKO TRAIL+Z+N vs FADD-RIPK1-DKO TRAIL+C+Z+N	0,17	ns
FADD-TRADD-DKO TRAIL+Z+N vs TRADD-RIPK1-DKO TRAIL	18	**
FADD-TRADD-DKO TRAIL+Z+N vs TRADD-RIPK1-DKO TRAIL+Z	3,0	ns
FADD-TRADD-DKO TRAIL+Z+N vs TRADD-RIPK1-DKO TRAIL+N	27	***
FADD-TRADD-DKO TRAIL+Z+N vs TRADD-RIPK1-DKO TRAIL+Z+N	-3,3	ns
FADD-TRADD-DKO TRAIL+Z+N vs TRADD-RIPK1-DKO TRAIL+C	83	***
FADD-TRADD-DKO TRAIL+Z+N vs TRADD-RIPK1-DKO TRAIL+C+Z	-3,3	ns
FADD-TRADD-DKO TRAIL+Z+N vs TRADD-RIPK1-DKO TRAIL+C+N	83	***
FADD-TRADD-DKO TRAIL+Z+N vs TRADD-RIPK1-DKO TRAIL+C+Z+N	-2,2	ns
FADD-TRADD-DKO TRAIL+Z+N vs Casp.8-KO TRAIL	8,9	ns
FADD-TRADD-DKO TRAIL+Z+N vs Casp.8-KO TRAIL+Z	18	**
FADD-TRADD-DKO TRAIL+Z+N vs Casp.8-KO TRAIL+N	-3,7	ns

FADD-TRADD-DKO TRAIL+Z+N vs Casp.8-KO TRAIL+Z+N	-5,2	ns
FADD-TRADD-DKO TRAIL+Z+N vs Casp.8-KO TRAIL+C	30	***
FADD-TRADD-DKO TRAIL+Z+N vs Casp.8-KO TRAIL+C+Z	33	***
FADD-TRADD-DKO TRAIL+Z+N vs Casp.8-KO TRAIL+C+N	3,3	ns
FADD-TRADD-DKO TRAIL+Z+N vs Casp.8-KO TRAIL+C+Z+N	-1,7	ns
FADD-TRADD-DKO TRAIL+Z+N vs EV TRAIL	-2,0	ns
FADD-TRADD-DKO TRAIL+Z+N vs EV TRAIL+Z	-3,1	ns
FADD-TRADD-DKO TRAIL+Z+N vs EV TRAIL+N	14	ns
FADD-TRADD-DKO TRAIL+Z+N vs EV TRAIL+Z+N	1,9	ns
FADD-TRADD-DKO TRAIL+Z+N vs EV TRAIL+C	74	***
FADD-TRADD-DKO TRAIL+Z+N vs EV TRAIL+C+Z	-1,6	ns
FADD-TRADD-DKO TRAIL+Z+N vs EV TRAIL+C+N	77	***
FADD-TRADD-DKO TRAIL+Z+N vs EV TRAIL+C+Z+N	1,6	ns
FADD-TRADD-DKO TRAIL+C vs FADD-TRADD-DKO TRAIL+C+Z	-23	ns
FADD-TRADD-DKO TRAIL+C vs FADD-TRADD-DKO TRAIL+C+N	-3,6	ns
FADD-TRADD-DKO TRAIL+C vs FADD-TRADD-DKO TRAIL+C+Z+N	-22	**
FADD-TRADD-DKO TRAIL+C vs FADD-RIPK1-DKO TRAIL	-28	***
FADD-TRADD-DKO TRAIL+C vs FADD-RIPK1-DKO TRAIL+Z	-26	***
FADD-TRADD-DKO TRAIL+C vs FADD-RIPK1-DKO TRAIL+N	-21	**
FADD-TRADD-DKO TRAIL+C vs FADD-RIPK1-DKO TRAIL+Z+N	-24	***
FADD-TRADD-DKO TRAIL+C vs FADD-RIPK1-DKO TRAIL+C	-16	ns
FADD-TRADD-DKO TRAIL+C vs FADD-RIPK1-DKO TRAIL+C+Z	-16	ns
FADD-TRADD-DKO TRAIL+C vs FADD-RIPK1-DKO TRAIL+C+N	-16	ns
FADD-TRADD-DKO TRAIL+C vs FADD-RIPK1-DKO TRAIL+C+Z+N	-23	***
FADD-TRADD-DKO TRAIL+C vs TRADD-RIPK1-DKO TRAIL	-5,4	ns
FADD-TRADD-DKO TRAIL+C vs TRADD-RIPK1-DKO TRAIL+Z	-20	**
FADD-TRADD-DKO TRAIL+C vs TRADD-RIPK1-DKO TRAIL+N	3,6	ns
FADD-TRADD-DKO TRAIL+C vs TRADD-RIPK1-DKO TRAIL+Z+N	-27	***
FADD-TRADD-DKO TRAIL+C vs TRADD-RIPK1-DKO TRAIL+C	59	***
FADD-TRADD-DKO TRAIL+C vs TRADD-RIPK1-DKO TRAIL+C+Z	-27	***
FADD-TRADD-DKO TRAIL+C vs TRADD-RIPK1-DKO TRAIL+C+N	60	***
FADD-TRADD-DKO TRAIL+C vs TRADD-RIPK1-DKO TRAIL+C+Z+N	-26	***
FADD-TRADD-DKO TRAIL+C vs Casp.8-KO TRAIL	-14	ns
FADD-TRADD-DKO TRAIL+C vs Casp.8-KO TRAIL+Z	-5,4	ns
FADD-TRADD-DKO TRAIL+C vs Casp.8-KO TRAIL+N	-27	***
FADD-TRADD-DKO TRAIL+C vs Casp.8-KO TRAIL+Z+N	-29	***
FADD-TRADD-DKO TRAIL+C vs Casp.8-KO TRAIL+C	6,6	ns
FADD-TRADD-DKO TRAIL+C vs Casp.8-KO TRAIL+C+Z	9,3	ns
FADD-TRADD-DKO TRAIL+C vs Casp.8-KO TRAIL+C+N	-20	**
FADD-TRADD-DKO TRAIL+C vs Casp.8-KO TRAIL+C+Z+N	-25	***
FADD-TRADD-DKO TRAIL+C vs EV TRAIL	-25	***
FADD-TRADD-DKO TRAIL+C vs EV TRAIL+Z	-26	***
FADD-TRADD-DKO TRAIL+C vs EV TRAIL+N	-9,4	ns
FADD-TRADD-DKO TRAIL+C vs EV TRAIL+Z+N	-21	ns
FADD-TRADD-DKO TRAIL+C vs EV TRAIL+C	51	***
FADD-TRADD-DKO TRAIL+C vs EV TRAIL+C+Z	-25	**
FADD-TRADD-DKO TRAIL+C vs EV TRAIL+C+N	54	***
FADD-TRADD-DKO TRAIL+C vs EV TRAIL+C+Z+N	-22	ns
FADD-TRADD-DKO TRAIL+C+Z vs FADD-TRADD-DKO TRAIL+C+N	20	ns
FADD-TRADD-DKO TRAIL+C+Z vs FADD-TRADD-DKO TRAIL+C+Z+N	1,5	ns
FADD-TRADD-DKO TRAIL+C+Z vs FADD-RIPK1-DKO TRAIL	-4,4	ns
FADD-TRADD-DKO TRAIL+C+Z vs FADD-RIPK1-DKO TRAIL+Z	-2,8	ns
FADD-TRADD-DKO TRAIL+C+Z vs FADD-RIPK1-DKO TRAIL+N	2,4	ns
FADD-TRADD-DKO TRAIL+C+Z vs FADD-RIPK1-DKO TRAIL+Z+N	-0,83	ns

FADD-TRADD-DKO TRAIL+C+Z vs FADD-RIPK1-DKO TRAIL+C	7,7	ns
FADD-TRADD-DKO TRAIL+C+Z vs FADD-RIPK1-DKO TRAIL+C+Z	7,1	ns
FADD-TRADD-DKO TRAIL+C+Z vs FADD-RIPK1-DKO TRAIL+C+N	7,4	ns
FADD-TRADD-DKO TRAIL+C+Z vs FADD-RIPK1-DKO TRAIL+C+Z+N	0,10	ns
FADD-TRADD-DKO TRAIL+C+Z vs TRADD-RIPK1-DKO TRAIL	18	ns
FADD-TRADD-DKO TRAIL+C+Z vs TRADD-RIPK1-DKO TRAIL+Z	2,9	ns
FADD-TRADD-DKO TRAIL+C+Z vs TRADD-RIPK1-DKO TRAIL+N	27	**
FADD-TRADD-DKO TRAIL+C+Z vs TRADD-RIPK1-DKO TRAIL+Z+N	-3,4	ns
FADD-TRADD-DKO TRAIL+C+Z vs TRADD-RIPK1-DKO TRAIL+C	83	***
FADD-TRADD-DKO TRAIL+C+Z vs TRADD-RIPK1-DKO TRAIL+C+Z	-3,3	ns
FADD-TRADD-DKO TRAIL+C+Z vs TRADD-RIPK1-DKO TRAIL+C+N	83	***
FADD-TRADD-DKO TRAIL+C+Z vs TRADD-RIPK1-DKO TRAIL+C+Z+N	-2,3	ns
FADD-TRADD-DKO TRAIL+C+Z vs Casp.8-KO TRAIL	8,9	ns
FADD-TRADD-DKO TRAIL+C+Z vs Casp.8-KO TRAIL+Z	18	ns
FADD-TRADD-DKO TRAIL+C+Z vs Casp.8-KO TRAIL+N	-3,8	ns
FADD-TRADD-DKO TRAIL+C+Z vs Casp.8-KO TRAIL+Z+N	-5,3	ns
FADD-TRADD-DKO TRAIL+C+Z vs Casp.8-KO TRAIL+C	30	***
FADD-TRADD-DKO TRAIL+C+Z vs Casp.8-KO TRAIL+C+Z	33	***
FADD-TRADD-DKO TRAIL+C+Z vs Casp.8-KO TRAIL+C+N	3,3	ns
FADD-TRADD-DKO TRAIL+C+Z vs Casp.8-KO TRAIL+C+Z+N	-1,8	ns
FADD-TRADD-DKO TRAIL+C+Z vs EV TRAIL	-2,1	ns
FADD-TRADD-DKO TRAIL+C+Z vs EV TRAIL+Z	-3,2	ns
FADD-TRADD-DKO TRAIL+C+Z vs EV TRAIL+N	14	ns
FADD-TRADD-DKO TRAIL+C+Z vs EV TRAIL+Z+N	1,8	ns
FADD-TRADD-DKO TRAIL+C+Z vs EV TRAIL+C	74	***
FADD-TRADD-DKO TRAIL+C+Z vs EV TRAIL+C+Z	-1,6	ns
FADD-TRADD-DKO TRAIL+C+Z vs EV TRAIL+C+N	77	***
FADD-TRADD-DKO TRAIL+C+Z vs EV TRAIL+C+Z+N	1,6	ns
FADD-TRADD-DKO TRAIL+C+N vs FADD-TRADD-DKO TRAIL+C+Z+N	-18	ns
FADD-TRADD-DKO TRAIL+C+N vs FADD-RIPK1-DKO TRAIL	-24	***
FADD-TRADD-DKO TRAIL+C+N vs FADD-RIPK1-DKO TRAIL+Z	-22	**
FADD-TRADD-DKO TRAIL+C+N vs FADD-RIPK1-DKO TRAIL+N	-17	ns
FADD-TRADD-DKO TRAIL+C+N vs FADD-RIPK1-DKO TRAIL+Z+N	-20	**
FADD-TRADD-DKO TRAIL+C+N vs FADD-RIPK1-DKO TRAIL+C	-12	ns
FADD-TRADD-DKO TRAIL+C+N vs FADD-RIPK1-DKO TRAIL+C+Z	-13	ns
FADD-TRADD-DKO TRAIL+C+N vs FADD-RIPK1-DKO TRAIL+C+N	-12	ns
FADD-TRADD-DKO TRAIL+C+N vs FADD-RIPK1-DKO TRAIL+C+Z+N	-20	ns
FADD-TRADD-DKO TRAIL+C+N vs TRADD-RIPK1-DKO TRAIL	-1,8	ns
FADD-TRADD-DKO TRAIL+C+N vs TRADD-RIPK1-DKO TRAIL+Z	-17	ns
FADD-TRADD-DKO TRAIL+C+N vs TRADD-RIPK1-DKO TRAIL+N	7,1	ns
FADD-TRADD-DKO TRAIL+C+N vs TRADD-RIPK1-DKO TRAIL+Z+N	-23	**
FADD-TRADD-DKO TRAIL+C+N vs TRADD-RIPK1-DKO TRAIL+C	63	***
FADD-TRADD-DKO TRAIL+C+N vs TRADD-RIPK1-DKO TRAIL+C+Z	-23	*
FADD-TRADD-DKO TRAIL+C+N vs TRADD-RIPK1-DKO TRAIL+C+N	64	***
FADD-TRADD-DKO TRAIL+C+N vs TRADD-RIPK1-DKO TRAIL+C+Z+N	-22	*
FADD-TRADD-DKO TRAIL+C+N vs Casp.8-KO TRAIL	-11	ns
FADD-TRADD-DKO TRAIL+C+N vs Casp.8-KO TRAIL+Z	-1,9	ns
FADD-TRADD-DKO TRAIL+C+N vs Casp.8-KO TRAIL+N	-23	**
FADD-TRADD-DKO TRAIL+C+N vs Casp.8-KO TRAIL+Z+N	-25	**
FADD-TRADD-DKO TRAIL+C+N vs Casp.8-KO TRAIL+C	10	ns
FADD-TRADD-DKO TRAIL+C+N vs Casp.8-KO TRAIL+C+Z	13	ns
FADD-TRADD-DKO TRAIL+C+N vs Casp.8-KO TRAIL+C+N	-16	ns
FADD-TRADD-DKO TRAIL+C+N vs Casp.8-KO TRAIL+C+Z+N	-21	*
FADD-TRADD-DKO TRAIL+C+N vs EV TRAIL	-22	ns

FADD-TRADD-DKO TRAIL+C+N vs EV TRAIL+Z	-23	ns
FADD-TRADD-DKO TRAIL+C+N vs EV TRAIL+N	-5,9	ns
FADD-TRADD-DKO TRAIL+C+N vs EV TRAIL+Z+N	-18	ns
FADD-TRADD-DKO TRAIL+C+N vs EV TRAIL+C	54	***
FADD-TRADD-DKO TRAIL+C+N vs EV TRAIL+C+Z	-21	ns
FADD-TRADD-DKO TRAIL+C+N vs EV TRAIL+C+N	58	***
FADD-TRADD-DKO TRAIL+C+N vs EV TRAIL+C+Z+N	-18	ns
FADD-TRADD-DKO TRAIL+C+Z+N vs FADD-RIPK1-DKO TRAIL	-5,9	ns
FADD-TRADD-DKO TRAIL+C+Z+N vs FADD-RIPK1-DKO TRAIL+Z	-4,3	ns
FADD-TRADD-DKO TRAIL+C+Z+N vs FADD-RIPK1-DKO TRAIL+N	0,93	ns
FADD-TRADD-DKO TRAIL+C+Z+N vs FADD-RIPK1-DKO TRAIL+Z+N	-2,3	ns
FADD-TRADD-DKO TRAIL+C+Z+N vs FADD-RIPK1-DKO TRAIL+C	6,2	ns
FADD-TRADD-DKO TRAIL+C+Z+N vs FADD-RIPK1-DKO TRAIL+C+Z	5,6	ns
FADD-TRADD-DKO TRAIL+C+Z+N vs FADD-RIPK1-DKO TRAIL+C+N	5,9	ns
FADD-TRADD-DKO TRAIL+C+Z+N vs FADD-RIPK1-DKO TRAIL+C+Z+N	-1,4	ns
FADD-TRADD-DKO TRAIL+C+Z+N vs TRADD-RIPK1-DKO TRAIL	16	ns
FADD-TRADD-DKO TRAIL+C+Z+N vs TRADD-RIPK1-DKO TRAIL+Z	1,4	ns
FADD-TRADD-DKO TRAIL+C+Z+N vs TRADD-RIPK1-DKO TRAIL+N	25	***
FADD-TRADD-DKO TRAIL+C+Z+N vs TRADD-RIPK1-DKO TRAIL+Z+N	-4,9	ns
FADD-TRADD-DKO TRAIL+C+Z+N vs TRADD-RIPK1-DKO TRAIL+C	81	***
FADD-TRADD-DKO TRAIL+C+Z+N vs TRADD-RIPK1-DKO TRAIL+C+Z	-4,8	ns
FADD-TRADD-DKO TRAIL+C+Z+N vs TRADD-RIPK1-DKO TRAIL+C+N	82	***
FADD-TRADD-DKO TRAIL+C+Z+N vs TRADD-RIPK1-DKO TRAIL+C+Z+N	-3,8	ns
FADD-TRADD-DKO TRAIL+C+Z+N vs Casp.8-KO TRAIL	7,4	ns
FADD-TRADD-DKO TRAIL+C+Z+N vs Casp.8-KO TRAIL+Z	16	ns
FADD-TRADD-DKO TRAIL+C+Z+N vs Casp.8-KO TRAIL+N	-5,3	ns
FADD-TRADD-DKO TRAIL+C+Z+N vs Casp.8-KO TRAIL+Z+N	-6,8	ns
FADD-TRADD-DKO TRAIL+C+Z+N vs Casp.8-KO TRAIL+C	28	***
FADD-TRADD-DKO TRAIL+C+Z+N vs Casp.8-KO TRAIL+C+Z	31	***
FADD-TRADD-DKO TRAIL+C+Z+N vs Casp.8-KO TRAIL+C+N	1,8	ns
FADD-TRADD-DKO TRAIL+C+Z+N vs Casp.8-KO TRAIL+C+Z+N	-3,3	ns
FADD-TRADD-DKO TRAIL+C+Z+N vs EV TRAIL	-3,6	ns
FADD-TRADD-DKO TRAIL+C+Z+N vs EV TRAIL+Z	-4,7	ns
FADD-TRADD-DKO TRAIL+C+Z+N vs EV TRAIL+N	12	ns
FADD-TRADD-DKO TRAIL+C+Z+N vs EV TRAIL+Z+N	0,30	ns
FADD-TRADD-DKO TRAIL+C+Z+N vs EV TRAIL+C	73	***
FADD-TRADD-DKO TRAIL+C+Z+N vs EV TRAIL+C+Z	-3,1	ns
FADD-TRADD-DKO TRAIL+C+Z+N vs EV TRAIL+C+N	76	***
FADD-TRADD-DKO TRAIL+C+Z+N vs EV TRAIL+C+Z+N	0,050	ns
FADD-RIPK1-DKO TRAIL vs FADD-RIPK1-DKO TRAIL+Z	1,6	ns
FADD-RIPK1-DKO TRAIL vs FADD-RIPK1-DKO TRAIL+N	6,8	ns
FADD-RIPK1-DKO TRAIL vs FADD-RIPK1-DKO TRAIL+Z+N	3,5	ns
FADD-RIPK1-DKO TRAIL vs FADD-RIPK1-DKO TRAIL+C	12	ns
FADD-RIPK1-DKO TRAIL vs FADD-RIPK1-DKO TRAIL+C+Z	11	ns
FADD-RIPK1-DKO TRAIL vs FADD-RIPK1-DKO TRAIL+C+N	12	ns
FADD-RIPK1-DKO TRAIL vs FADD-RIPK1-DKO TRAIL+C+Z+N	4,5	ns
FADD-RIPK1-DKO TRAIL vs TRADD-RIPK1-DKO TRAIL	22	***
FADD-RIPK1-DKO TRAIL vs TRADD-RIPK1-DKO TRAIL+Z	7,3	ns
FADD-RIPK1-DKO TRAIL vs TRADD-RIPK1-DKO TRAIL+N	31	***
FADD-RIPK1-DKO TRAIL vs TRADD-RIPK1-DKO TRAIL+Z+N	0,97	ns
FADD-RIPK1-DKO TRAIL vs TRADD-RIPK1-DKO TRAIL+C	87	***
FADD-RIPK1-DKO TRAIL vs TRADD-RIPK1-DKO TRAIL+C+Z	1,0	ns
FADD-RIPK1-DKO TRAIL vs TRADD-RIPK1-DKO TRAIL+C+N	88	***
FADD-RIPK1-DKO TRAIL vs TRADD-RIPK1-DKO TRAIL+C+Z+N	2,1	ns

FADD-RIPK1-DKO TRAIL vs Casp.8-KO TRAIL	13	ns
FADD-RIPK1-DKO TRAIL vs Casp.8-KO TRAIL+Z	22	***
FADD-RIPK1-DKO TRAIL vs Casp.8-KO TRAIL+N	0,57	ns
FADD-RIPK1-DKO TRAIL vs Casp.8-KO TRAIL+Z+N	-0,94	ns
FADD-RIPK1-DKO TRAIL vs Casp.8-KO TRAIL+C	34	***
FADD-RIPK1-DKO TRAIL vs Casp.8-KO TRAIL+C+Z	37	***
FADD-RIPK1-DKO TRAIL vs Casp.8-KO TRAIL+C+N	7,6	ns
FADD-RIPK1-DKO TRAIL vs Casp.8-KO TRAIL+C+Z+N	2,6	ns
FADD-RIPK1-DKO TRAIL vs EV TRAIL	2,3	ns
FADD-RIPK1-DKO TRAIL vs EV TRAIL+Z	1,2	ns
FADD-RIPK1-DKO TRAIL vs EV TRAIL+N	18	ns
FADD-RIPK1-DKO TRAIL vs EV TRAIL+Z+N	6,2	ns
FADD-RIPK1-DKO TRAIL vs EV TRAIL+C	78	***
FADD-RIPK1-DKO TRAIL vs EV TRAIL+C+Z	2,7	ns
FADD-RIPK1-DKO TRAIL vs EV TRAIL+C+N	82	***
FADD-RIPK1-DKO TRAIL vs EV TRAIL+C+Z+N	5,9	ns
FADD-RIPK1-DKO TRAIL+Z vs FADD-RIPK1-DKO TRAIL+N	5,2	ns
FADD-RIPK1-DKO TRAIL+Z vs FADD-RIPK1-DKO TRAIL+Z+N	1,9	ns
FADD-RIPK1-DKO TRAIL+Z vs FADD-RIPK1-DKO TRAIL+C	10	ns
FADD-RIPK1-DKO TRAIL+Z vs FADD-RIPK1-DKO TRAIL+C+Z	9,8	ns
FADD-RIPK1-DKO TRAIL+Z vs FADD-RIPK1-DKO TRAIL+C+N	10	ns
FADD-RIPK1-DKO TRAIL+Z vs FADD-RIPK1-DKO TRAIL+C+Z+N	2,9	ns
FADD-RIPK1-DKO TRAIL+Z vs TRADD-RIPK1-DKO TRAIL	21	***
FADD-RIPK1-DKO TRAIL+Z vs TRADD-RIPK1-DKO TRAIL+Z	5,6	ns
FADD-RIPK1-DKO TRAIL+Z vs TRADD-RIPK1-DKO TRAIL+N	30	***
FADD-RIPK1-DKO TRAIL+Z vs TRADD-RIPK1-DKO TRAIL+Z+N	-0,64	ns
FADD-RIPK1-DKO TRAIL+Z vs TRADD-RIPK1-DKO TRAIL+C	85	***
FADD-RIPK1-DKO TRAIL+Z vs TRADD-RIPK1-DKO TRAIL+C+Z	-0,56	ns
FADD-RIPK1-DKO TRAIL+Z vs TRADD-RIPK1-DKO TRAIL+C+N	86	***
FADD-RIPK1-DKO TRAIL+Z vs TRADD-RIPK1-DKO TRAIL+C+Z+N	0,47	ns
FADD-RIPK1-DKO TRAIL+Z vs Casp.8-KO TRAIL	12	ns
FADD-RIPK1-DKO TRAIL+Z vs Casp.8-KO TRAIL+Z	21	***
FADD-RIPK1-DKO TRAIL+Z vs Casp.8-KO TRAIL+N	-1,0	ns
FADD-RIPK1-DKO TRAIL+Z vs Casp.8-KO TRAIL+Z+N	-2,5	ns
FADD-RIPK1-DKO TRAIL+Z vs Casp.8-KO TRAIL+C	33	***
FADD-RIPK1-DKO TRAIL+Z vs Casp.8-KO TRAIL+C+Z	35	***
FADD-RIPK1-DKO TRAIL+Z vs Casp.8-KO TRAIL+C+N	6,0	ns
FADD-RIPK1-DKO TRAIL+Z vs Casp.8-KO TRAIL+C+Z+N	0,96	ns
FADD-RIPK1-DKO TRAIL+Z vs EV TRAIL	0,69	ns
FADD-RIPK1-DKO TRAIL+Z vs EV TRAIL+Z	-0,44	ns
FADD-RIPK1-DKO TRAIL+Z vs EV TRAIL+N	17	ns
FADD-RIPK1-DKO TRAIL+Z vs EV TRAIL+Z+N	4,6	ns
FADD-RIPK1-DKO TRAIL+Z vs EV TRAIL+C	77	***
FADD-RIPK1-DKO TRAIL+Z vs EV TRAIL+C+Z	1,1	ns
FADD-RIPK1-DKO TRAIL+Z vs EV TRAIL+C+N	80	***
FADD-RIPK1-DKO TRAIL+Z vs EV TRAIL+C+Z+N	4,3	ns
FADD-RIPK1-DKO TRAIL+N vs FADD-RIPK1-DKO TRAIL+Z+N	-3,3	ns
FADD-RIPK1-DKO TRAIL+N vs FADD-RIPK1-DKO TRAIL+C	5,2	ns
FADD-RIPK1-DKO TRAIL+N vs FADD-RIPK1-DKO TRAIL+C+Z	4,6	ns
FADD-RIPK1-DKO TRAIL+N vs FADD-RIPK1-DKO TRAIL+C+N	5,0	ns
FADD-RIPK1-DKO TRAIL+N vs FADD-RIPK1-DKO TRAIL+C+Z+N	-2,3	ns
FADD-RIPK1-DKO TRAIL+N vs TRADD-RIPK1-DKO TRAIL	15	ns
FADD-RIPK1-DKO TRAIL+N vs TRADD-RIPK1-DKO TRAIL+Z	0,46	ns
FADD-RIPK1-DKO TRAIL+N vs TRADD-RIPK1-DKO TRAIL+N	24	***

FADD-RIPK1-DKO TRAIL+N vs TRADD-RIPK1-DKO TRAIL+Z+N	-5,8	ns
FADD-RIPK1-DKO TRAIL+N vs TRADD-RIPK1-DKO TRAIL+C	80	***
FADD-RIPK1-DKO TRAIL+N vs TRADD-RIPK1-DKO TRAIL+C+Z	-5,8	ns
FADD-RIPK1-DKO TRAIL+N vs TRADD-RIPK1-DKO TRAIL+C+N	81	***
FADD-RIPK1-DKO TRAIL+N vs TRADD-RIPK1-DKO TRAIL+C+Z+N	-4,7	ns
FADD-RIPK1-DKO TRAIL+N vs Casp.8-KO TRAIL	6,4	ns
FADD-RIPK1-DKO TRAIL+N vs Casp.8-KO TRAIL+Z	15	ns
FADD-RIPK1-DKO TRAIL+N vs Casp.8-KO TRAIL+N	-6,2	ns
FADD-RIPK1-DKO TRAIL+N vs Casp.8-KO TRAIL+Z+N	-7,7	ns
FADD-RIPK1-DKO TRAIL+N vs Casp.8-KO TRAIL+C	27	***
FADD-RIPK1-DKO TRAIL+N vs Casp.8-KO TRAIL+C+Z	30	***
FADD-RIPK1-DKO TRAIL+N vs Casp.8-KO TRAIL+C+N	0,84	ns
FADD-RIPK1-DKO TRAIL+N vs Casp.8-KO TRAIL+C+Z+N	-4,2	ns
FADD-RIPK1-DKO TRAIL+N vs EV TRAIL	-4,5	ns
FADD-RIPK1-DKO TRAIL+N vs EV TRAIL+Z	-5,6	ns
FADD-RIPK1-DKO TRAIL+N vs EV TRAIL+N	11	ns
FADD-RIPK1-DKO TRAIL+N vs EV TRAIL+Z+N	-0,63	ns
FADD-RIPK1-DKO TRAIL+N vs EV TRAIL+C	72	***
FADD-RIPK1-DKO TRAIL+N vs EV TRAIL+C+Z	-4,1	ns
FADD-RIPK1-DKO TRAIL+N vs EV TRAIL+C+N	75	***
FADD-RIPK1-DKO TRAIL+N vs EV TRAIL+C+Z+N	-0,88	ns
FADD-RIPK1-DKO TRAIL+Z+N vs FADD-RIPK1-DKO TRAIL+C	8,5	ns
FADD-RIPK1-DKO TRAIL+Z+N vs FADD-RIPK1-DKO TRAIL+C+Z	7,9	ns
FADD-RIPK1-DKO TRAIL+Z+N vs FADD-RIPK1-DKO TRAIL+C+N	8,2	ns
FADD-RIPK1-DKO TRAIL+Z+N vs FADD-RIPK1-DKO TRAIL+C+Z+N	0,93	ns
FADD-RIPK1-DKO TRAIL+Z+N vs TRADD-RIPK1-DKO TRAIL	19	***
FADD-RIPK1-DKO TRAIL+Z+N vs TRADD-RIPK1-DKO TRAIL+Z	3,7	ns
FADD-RIPK1-DKO TRAIL+Z+N vs TRADD-RIPK1-DKO TRAIL+N	28	***
FADD-RIPK1-DKO TRAIL+Z+N vs TRADD-RIPK1-DKO TRAIL+Z+N	-2,6	ns
FADD-RIPK1-DKO TRAIL+Z+N vs TRADD-RIPK1-DKO TRAIL+C	83	***
FADD-RIPK1-DKO TRAIL+Z+N vs TRADD-RIPK1-DKO TRAIL+C+Z	-2,5	ns
FADD-RIPK1-DKO TRAIL+Z+N vs TRADD-RIPK1-DKO TRAIL+C+N	84	***
FADD-RIPK1-DKO TRAIL+Z+N vs TRADD-RIPK1-DKO TRAIL+C+Z+N	-1,5	ns
FADD-RIPK1-DKO TRAIL+Z+N vs Casp.8-KO TRAIL	9,7	ns
FADD-RIPK1-DKO TRAIL+Z+N vs Casp.8-KO TRAIL+Z	19	***
FADD-RIPK1-DKO TRAIL+Z+N vs Casp.8-KO TRAIL+N	-3,0	ns
FADD-RIPK1-DKO TRAIL+Z+N vs Casp.8-KO TRAIL+Z+N	-4,5	ns
FADD-RIPK1-DKO TRAIL+Z+N vs Casp.8-KO TRAIL+C	31	***
FADD-RIPK1-DKO TRAIL+Z+N vs Casp.8-KO TRAIL+C+Z	33	***
FADD-RIPK1-DKO TRAIL+Z+N vs Casp.8-KO TRAIL+C+N	4,1	ns
FADD-RIPK1-DKO TRAIL+Z+N vs Casp.8-KO TRAIL+C+Z+N	-0,97	ns
FADD-RIPK1-DKO TRAIL+Z+N vs EV TRAIL	-1,2	ns
FADD-RIPK1-DKO TRAIL+Z+N vs EV TRAIL+Z	-2,4	ns
FADD-RIPK1-DKO TRAIL+Z+N vs EV TRAIL+N	15	ns
FADD-RIPK1-DKO TRAIL+Z+N vs EV TRAIL+Z+N	2,6	ns
FADD-RIPK1-DKO TRAIL+Z+N vs EV TRAIL+C	75	***
FADD-RIPK1-DKO TRAIL+Z+N vs EV TRAIL+C+Z	-0,80	ns
FADD-RIPK1-DKO TRAIL+Z+N vs EV TRAIL+C+N	78	***
FADD-RIPK1-DKO TRAIL+Z+N vs EV TRAIL+C+Z+N	2,4	ns
FADD-RIPK1-DKO TRAIL+C vs FADD-RIPK1-DKO TRAIL+C+Z	-0,58	ns
FADD-RIPK1-DKO TRAIL+C vs FADD-RIPK1-DKO TRAIL+C+N	-0,27	ns
FADD-RIPK1-DKO TRAIL+C vs FADD-RIPK1-DKO TRAIL+C+Z+N	-7,6	ns
FADD-RIPK1-DKO TRAIL+C vs TRADD-RIPK1-DKO TRAIL	10	ns
FADD-RIPK1-DKO TRAIL+C vs TRADD-RIPK1-DKO TRAIL+Z	-4,8	ns

FADD-RIPK1-DKO TRAIL+C vs TRADD-RIPK1-DKO TRAIL+N	19	*
FADD-RIPK1-DKO TRAIL+C vs TRADD-RIPK1-DKO TRAIL+Z+N	-11	ns
FADD-RIPK1-DKO TRAIL+C vs TRADD-RIPK1-DKO TRAIL+C	75	***
FADD-RIPK1-DKO TRAIL+C vs TRADD-RIPK1-DKO TRAIL+C+Z	-11	ns
FADD-RIPK1-DKO TRAIL+C vs TRADD-RIPK1-DKO TRAIL+C+N	76	***
FADD-RIPK1-DKO TRAIL+C vs TRADD-RIPK1-DKO TRAIL+C+Z+N	-9,9	ns
FADD-RIPK1-DKO TRAIL+C vs Casp.8-KO TRAIL	1,2	ns
FADD-RIPK1-DKO TRAIL+C vs Casp.8-KO TRAIL+Z	10	ns
FADD-RIPK1-DKO TRAIL+C vs Casp.8-KO TRAIL+N	-11	ns
FADD-RIPK1-DKO TRAIL+C vs Casp.8-KO TRAIL+Z+N	-13	ns
FADD-RIPK1-DKO TRAIL+C vs Casp.8-KO TRAIL+C	22	***
FADD-RIPK1-DKO TRAIL+C vs Casp.8-KO TRAIL+C+Z	25	***
FADD-RIPK1-DKO TRAIL+C vs Casp.8-KO TRAIL+C+N	-4,4	ns
FADD-RIPK1-DKO TRAIL+C vs Casp.8-KO TRAIL+C+Z+N	-9,5	ns
FADD-RIPK1-DKO TRAIL+C vs EV TRAIL	-9,7	ns
FADD-RIPK1-DKO TRAIL+C vs EV TRAIL+Z	-11	ns
FADD-RIPK1-DKO TRAIL+C vs EV TRAIL+N	6,1	ns
FADD-RIPK1-DKO TRAIL+C vs EV TRAIL+Z+N	-5,9	ns
FADD-RIPK1-DKO TRAIL+C vs EV TRAIL+C	66	***
FADD-RIPK1-DKO TRAIL+C vs EV TRAIL+C+Z	-9,3	ns
FADD-RIPK1-DKO TRAIL+C vs EV TRAIL+C+N	70	***
FADD-RIPK1-DKO TRAIL+C vs EV TRAIL+C+Z+N	-6,1	ns
FADD-RIPK1-DKO TRAIL+C+Z vs FADD-RIPK1-DKO TRAIL+C+N	0,31	ns
FADD-RIPK1-DKO TRAIL+C+Z vs FADD-RIPK1-DKO TRAIL+C+Z+N	-7,0	ns
FADD-RIPK1-DKO TRAIL+C+Z vs TRADD-RIPK1-DKO TRAIL	11	ns
FADD-RIPK1-DKO TRAIL+C+Z vs TRADD-RIPK1-DKO TRAIL+Z	-4,2	ns
FADD-RIPK1-DKO TRAIL+C+Z vs TRADD-RIPK1-DKO TRAIL+N	20	*
FADD-RIPK1-DKO TRAIL+C+Z vs TRADD-RIPK1-DKO TRAIL+Z+N	-10	ns
FADD-RIPK1-DKO TRAIL+C+Z vs TRADD-RIPK1-DKO TRAIL+C	75	***
FADD-RIPK1-DKO TRAIL+C+Z vs TRADD-RIPK1-DKO TRAIL+C+Z	-10	ns
FADD-RIPK1-DKO TRAIL+C+Z vs TRADD-RIPK1-DKO TRAIL+C+N	76	***
FADD-RIPK1-DKO TRAIL+C+Z vs TRADD-RIPK1-DKO TRAIL+C+Z+N	-9,4	ns
FADD-RIPK1-DKO TRAIL+C+Z vs Casp.8-KO TRAIL	1,8	ns
FADD-RIPK1-DKO TRAIL+C+Z vs Casp.8-KO TRAIL+Z	11	ns
FADD-RIPK1-DKO TRAIL+C+Z vs Casp.8-KO TRAIL+N	-11	ns
FADD-RIPK1-DKO TRAIL+C+Z vs Casp.8-KO TRAIL+Z+N	-12	ns
FADD-RIPK1-DKO TRAIL+C+Z vs Casp.8-KO TRAIL+C	23	***
FADD-RIPK1-DKO TRAIL+C+Z vs Casp.8-KO TRAIL+C+Z	25	***
FADD-RIPK1-DKO TRAIL+C+Z vs Casp.8-KO TRAIL+C+N	-3,8	ns
FADD-RIPK1-DKO TRAIL+C+Z vs Casp.8-KO TRAIL+C+Z+N	-8,9	ns
FADD-RIPK1-DKO TRAIL+C+Z vs EV TRAIL	-9,1	ns
FADD-RIPK1-DKO TRAIL+C+Z vs EV TRAIL+Z	-10	ns
FADD-RIPK1-DKO TRAIL+C+Z vs EV TRAIL+N	6,7	ns
FADD-RIPK1-DKO TRAIL+C+Z vs EV TRAIL+Z+N	-5,3	ns
FADD-RIPK1-DKO TRAIL+C+Z vs EV TRAIL+C	67	***
FADD-RIPK1-DKO TRAIL+C+Z vs EV TRAIL+C+Z	-8,7	ns
FADD-RIPK1-DKO TRAIL+C+Z vs EV TRAIL+C+N	70	***
FADD-RIPK1-DKO TRAIL+C+Z vs EV TRAIL+C+Z+N	-5,5	ns
FADD-RIPK1-DKO TRAIL+C+N vs FADD-RIPK1-DKO TRAIL+C+Z+N	-7,3	ns
FADD-RIPK1-DKO TRAIL+C+N vs TRADD-RIPK1-DKO TRAIL	11	ns
FADD-RIPK1-DKO TRAIL+C+N vs TRADD-RIPK1-DKO TRAIL+Z	-4,5	ns
FADD-RIPK1-DKO TRAIL+C+N vs TRADD-RIPK1-DKO TRAIL+N	19	*
FADD-RIPK1-DKO TRAIL+C+N vs TRADD-RIPK1-DKO TRAIL+Z+N	-11	ns
FADD-RIPK1-DKO TRAIL+C+N vs TRADD-RIPK1-DKO TRAIL+C	75	***

FADD-RIPK1-DKO TRAIL+C+N vs TRADD-RIPK1-DKO TRAIL+C+Z	-11	ns
FADD-RIPK1-DKO TRAIL+C+N vs TRADD-RIPK1-DKO TRAIL+C+N	76	***
FADD-RIPK1-DKO TRAIL+C+N vs TRADD-RIPK1-DKO TRAIL+C+Z+N	-9,7	ns
FADD-RIPK1-DKO TRAIL+C+N vs Casp.8-KO TRAIL	1,5	ns
FADD-RIPK1-DKO TRAIL+C+N vs Casp.8-KO TRAIL+Z	10	ns
FADD-RIPK1-DKO TRAIL+C+N vs Casp.8-KO TRAIL+N	-11	ns
FADD-RIPK1-DKO TRAIL+C+N vs Casp.8-KO TRAIL+Z+N	-13	ns
FADD-RIPK1-DKO TRAIL+C+N vs Casp.8-KO TRAIL+C	22	***
FADD-RIPK1-DKO TRAIL+C+N vs Casp.8-KO TRAIL+C+Z	25	***
FADD-RIPK1-DKO TRAIL+C+N vs Casp.8-KO TRAIL+C+N	-4,1	ns
FADD-RIPK1-DKO TRAIL+C+N vs Casp.8-KO TRAIL+C+Z+N	-9,2	ns
FADD-RIPK1-DKO TRAIL+C+N vs EV TRAIL	-9,5	ns
FADD-RIPK1-DKO TRAIL+C+N vs EV TRAIL+Z	-11	ns
FADD-RIPK1-DKO TRAIL+C+N vs EV TRAIL+N	6,4	ns
FADD-RIPK1-DKO TRAIL+C+N vs EV TRAIL+Z+N	-5,6	ns
FADD-RIPK1-DKO TRAIL+C+N vs EV TRAIL+C	67	***
FADD-RIPK1-DKO TRAIL+C+N vs EV TRAIL+C+Z	-9,0	ns
FADD-RIPK1-DKO TRAIL+C+N vs EV TRAIL+C+N	70	***
FADD-RIPK1-DKO TRAIL+C+N vs EV TRAIL+C+Z+N	-5,8	ns
FADD-RIPK1-DKO TRAIL+C+Z+N vs TRADD-RIPK1-DKO TRAIL	18	ns
FADD-RIPK1-DKO TRAIL+C+Z+N vs TRADD-RIPK1-DKO TRAIL+Z	2,8	ns
FADD-RIPK1-DKO TRAIL+C+Z+N vs TRADD-RIPK1-DKO TRAIL+N	27	***
FADD-RIPK1-DKO TRAIL+C+Z+N vs TRADD-RIPK1-DKO TRAIL+Z+N	-3,5	ns
FADD-RIPK1-DKO TRAIL+C+Z+N vs TRADD-RIPK1-DKO TRAIL+C	82	***
FADD-RIPK1-DKO TRAIL+C+Z+N vs TRADD-RIPK1-DKO TRAIL+C+Z	-3,4	ns
FADD-RIPK1-DKO TRAIL+C+Z+N vs TRADD-RIPK1-DKO TRAIL+C+N	83	***
FADD-RIPK1-DKO TRAIL+C+Z+N vs TRADD-RIPK1-DKO TRAIL+C+Z+N	-2,4	ns
FADD-RIPK1-DKO TRAIL+C+Z+N vs Casp.8-KO TRAIL	8,8	ns
FADD-RIPK1-DKO TRAIL+C+Z+N vs Casp.8-KO TRAIL+Z	18	*
FADD-RIPK1-DKO TRAIL+C+Z+N vs Casp.8-KO TRAIL+N	-3,9	ns
FADD-RIPK1-DKO TRAIL+C+Z+N vs Casp.8-KO TRAIL+Z+N	-5,4	ns
FADD-RIPK1-DKO TRAIL+C+Z+N vs Casp.8-KO TRAIL+C	30	***
FADD-RIPK1-DKO TRAIL+C+Z+N vs Casp.8-KO TRAIL+C+Z	32	***
FADD-RIPK1-DKO TRAIL+C+Z+N vs Casp.8-KO TRAIL+C+N	3,2	ns
FADD-RIPK1-DKO TRAIL+C+Z+N vs Casp.8-KO TRAIL+C+Z+N	-1,9	ns
FADD-RIPK1-DKO TRAIL+C+Z+N vs EV TRAIL	-2,2	ns
FADD-RIPK1-DKO TRAIL+C+Z+N vs EV TRAIL+Z	-3,3	ns
FADD-RIPK1-DKO TRAIL+C+Z+N vs EV TRAIL+N	14	ns
FADD-RIPK1-DKO TRAIL+C+Z+N vs EV TRAIL+Z+N	1,7	ns
FADD-RIPK1-DKO TRAIL+C+Z+N vs EV TRAIL+C	74	***
FADD-RIPK1-DKO TRAIL+C+Z+N vs EV TRAIL+C+Z	-1,7	ns
FADD-RIPK1-DKO TRAIL+C+Z+N vs EV TRAIL+C+N	77	***
FADD-RIPK1-DKO TRAIL+C+Z+N vs EV TRAIL+C+Z+N	1,4	ns
TRADD-RIPK1-DKO TRAIL vs TRADD-RIPK1-DKO TRAIL+Z	-15	ns
TRADD-RIPK1-DKO TRAIL vs TRADD-RIPK1-DKO TRAIL+N	8,9	ns
TRADD-RIPK1-DKO TRAIL vs TRADD-RIPK1-DKO TRAIL+Z+N	-21	**
TRADD-RIPK1-DKO TRAIL vs TRADD-RIPK1-DKO TRAIL+C	65	***
TRADD-RIPK1-DKO TRAIL vs TRADD-RIPK1-DKO TRAIL+C+Z	-21	**
TRADD-RIPK1-DKO TRAIL vs TRADD-RIPK1-DKO TRAIL+C+N	65	***
TRADD-RIPK1-DKO TRAIL vs TRADD-RIPK1-DKO TRAIL+C+Z+N	-20	**
TRADD-RIPK1-DKO TRAIL vs Casp.8-KO TRAIL	-9,0	ns
TRADD-RIPK1-DKO TRAIL vs Casp.8-KO TRAIL+Z	-0,083	ns
TRADD-RIPK1-DKO TRAIL vs Casp.8-KO TRAIL+N	-22	***
TRADD-RIPK1-DKO TRAIL vs Casp.8-KO TRAIL+Z+N	-23	***

TRADD-RIPK1-DKO TRAIL vs Casp.8-KO TRAIL+C	12	ns
TRADD-RIPK1-DKO TRAIL vs Casp.8-KO TRAIL+C+Z	15	ns
TRADD-RIPK1-DKO TRAIL vs Casp.8-KO TRAIL+C+N	-15	ns
TRADD-RIPK1-DKO TRAIL vs Casp.8-KO TRAIL+C+Z+N	-20	**
TRADD-RIPK1-DKO TRAIL vs EV TRAIL	-20	*
TRADD-RIPK1-DKO TRAIL vs EV TRAIL+Z	-21	*
TRADD-RIPK1-DKO TRAIL vs EV TRAIL+N	-4,1	ns
TRADD-RIPK1-DKO TRAIL vs EV TRAIL+Z+N	-16	ns
TRADD-RIPK1-DKO TRAIL vs EV TRAIL+C	56	***
TRADD-RIPK1-DKO TRAIL vs EV TRAIL+C+Z	-20	ns
TRADD-RIPK1-DKO TRAIL vs EV TRAIL+C+N	59	***
TRADD-RIPK1-DKO TRAIL vs EV TRAIL+C+Z+N	-16	ns
TRADD-RIPK1-DKO TRAIL+Z vs TRADD-RIPK1-DKO TRAIL+N	24	***
TRADD-RIPK1-DKO TRAIL+Z vs TRADD-RIPK1-DKO TRAIL+Z+N	-6,3	ns
TRADD-RIPK1-DKO TRAIL+Z vs TRADD-RIPK1-DKO TRAIL+C	80	***
TRADD-RIPK1-DKO TRAIL+Z vs TRADD-RIPK1-DKO TRAIL+C+Z	-6,2	ns
TRADD-RIPK1-DKO TRAIL+Z vs TRADD-RIPK1-DKO TRAIL+C+N	80	***
TRADD-RIPK1-DKO TRAIL+Z vs TRADD-RIPK1-DKO TRAIL+C+Z+N	-5,2	ns
TRADD-RIPK1-DKO TRAIL+Z vs Casp.8-KO TRAIL	6,0	ns
TRADD-RIPK1-DKO TRAIL+Z vs Casp.8-KO TRAIL+Z	15	ns
TRADD-RIPK1-DKO TRAIL+Z vs Casp.8-KO TRAIL+N	-6,7	ns
TRADD-RIPK1-DKO TRAIL+Z vs Casp.8-KO TRAIL+Z+N	-8,2	ns
TRADD-RIPK1-DKO TRAIL+Z vs Casp.8-KO TRAIL+C	27	***
TRADD-RIPK1-DKO TRAIL+Z vs Casp.8-KO TRAIL+C+Z	30	***
TRADD-RIPK1-DKO TRAIL+Z vs Casp.8-KO TRAIL+C+N	0,38	ns
TRADD-RIPK1-DKO TRAIL+Z vs Casp.8-KO TRAIL+C+Z+N	-4,7	ns
TRADD-RIPK1-DKO TRAIL+Z vs EV TRAIL	-5,0	ns
TRADD-RIPK1-DKO TRAIL+Z vs EV TRAIL+Z	-6,1	ns
TRADD-RIPK1-DKO TRAIL+Z vs EV TRAIL+N	11	ns
TRADD-RIPK1-DKO TRAIL+Z vs EV TRAIL+Z+N	-1,1	ns
TRADD-RIPK1-DKO TRAIL+Z vs EV TRAIL+C	71	***
TRADD-RIPK1-DKO TRAIL+Z vs EV TRAIL+C+Z	-4,5	ns
TRADD-RIPK1-DKO TRAIL+Z vs EV TRAIL+C+N	74	***
TRADD-RIPK1-DKO TRAIL+Z vs EV TRAIL+C+Z+N	-1,3	ns
TRADD-RIPK1-DKO TRAIL+N vs TRADD-RIPK1-DKO TRAIL+Z+N	-30	***
TRADD-RIPK1-DKO TRAIL+N vs TRADD-RIPK1-DKO TRAIL+C	56	***
TRADD-RIPK1-DKO TRAIL+N vs TRADD-RIPK1-DKO TRAIL+C+Z	-30	***
TRADD-RIPK1-DKO TRAIL+N vs TRADD-RIPK1-DKO TRAIL+C+N	57	***
TRADD-RIPK1-DKO TRAIL+N vs TRADD-RIPK1-DKO TRAIL+C+Z+N	-29	***
TRADD-RIPK1-DKO TRAIL+N vs Casp.8-KO TRAIL	-18	*
TRADD-RIPK1-DKO TRAIL+N vs Casp.8-KO TRAIL+Z	-9,0	ns
TRADD-RIPK1-DKO TRAIL+N vs Casp.8-KO TRAIL+N	-31	***
TRADD-RIPK1-DKO TRAIL+N vs Casp.8-KO TRAIL+Z+N	-32	***
TRADD-RIPK1-DKO TRAIL+N vs Casp.8-KO TRAIL+C	3,0	ns
TRADD-RIPK1-DKO TRAIL+N vs Casp.8-KO TRAIL+C+Z	5,8	ns
TRADD-RIPK1-DKO TRAIL+N vs Casp.8-KO TRAIL+C+N	-24	***
TRADD-RIPK1-DKO TRAIL+N vs Casp.8-KO TRAIL+C+Z+N	-29	***
TRADD-RIPK1-DKO TRAIL+N vs EV TRAIL	-29	***
TRADD-RIPK1-DKO TRAIL+N vs EV TRAIL+Z	-30	***
TRADD-RIPK1-DKO TRAIL+N vs EV TRAIL+N	-13	ns
TRADD-RIPK1-DKO TRAIL+N vs EV TRAIL+Z+N	-25	ns
TRADD-RIPK1-DKO TRAIL+N vs EV TRAIL+C	47	***
TRADD-RIPK1-DKO TRAIL+N vs EV TRAIL+C+Z	-28	***
TRADD-RIPK1-DKO TRAIL+N vs EV TRAIL+C+N	51	***

TRADD-RIPK1-DKO TRAIL+N vs EV TRAIL+C+Z+N	-25	ns
TRADD-RIPK1-DKO TRAIL+Z+N vs TRADD-RIPK1-DKO TRAIL+C	86	***
TRADD-RIPK1-DKO TRAIL+Z+N vs TRADD-RIPK1-DKO TRAIL+C+Z	0,075	ns
TRADD-RIPK1-DKO TRAIL+Z+N vs TRADD-RIPK1-DKO TRAIL+C+N	87	***
TRADD-RIPK1-DKO TRAIL+Z+N vs TRADD-RIPK1-DKO TRAIL+C+Z+N	1,1	ns
TRADD-RIPK1-DKO TRAIL+Z+N vs Casp.8-KO TRAIL	12	ns
TRADD-RIPK1-DKO TRAIL+Z+N vs Casp.8-KO TRAIL+Z	21	***
TRADD-RIPK1-DKO TRAIL+Z+N vs Casp.8-KO TRAIL+N	-0,40	ns
TRADD-RIPK1-DKO TRAIL+Z+N vs Casp.8-KO TRAIL+Z+N	-1,9	ns
TRADD-RIPK1-DKO TRAIL+Z+N vs Casp.8-KO TRAIL+C	33	***
TRADD-RIPK1-DKO TRAIL+Z+N vs Casp.8-KO TRAIL+C+Z	36	***
TRADD-RIPK1-DKO TRAIL+Z+N vs Casp.8-KO TRAIL+C+N	6,7	ns
TRADD-RIPK1-DKO TRAIL+Z+N vs Casp.8-KO TRAIL+C+Z+N	1,6	ns
TRADD-RIPK1-DKO TRAIL+Z+N vs EV TRAIL	1,3	ns
TRADD-RIPK1-DKO TRAIL+Z+N vs EV TRAIL+Z	0,20	ns
TRADD-RIPK1-DKO TRAIL+Z+N vs EV TRAIL+N	17	ns
TRADD-RIPK1-DKO TRAIL+Z+N vs EV TRAIL+Z+N	5,2	ns
TRADD-RIPK1-DKO TRAIL+Z+N vs EV TRAIL+C	77	***
TRADD-RIPK1-DKO TRAIL+Z+N vs EV TRAIL+C+Z	1,8	ns
TRADD-RIPK1-DKO TRAIL+Z+N vs EV TRAIL+C+N	81	***
TRADD-RIPK1-DKO TRAIL+Z+N vs EV TRAIL+C+Z+N	4,9	ns
TRADD-RIPK1-DKO TRAIL+C vs TRADD-RIPK1-DKO TRAIL+C+Z	-86	***
TRADD-RIPK1-DKO TRAIL+C vs TRADD-RIPK1-DKO TRAIL+C+N	0,75	ns
TRADD-RIPK1-DKO TRAIL+C vs TRADD-RIPK1-DKO TRAIL+C+Z+N	-85	***
TRADD-RIPK1-DKO TRAIL+C vs Casp.8-KO TRAIL	-74	***
TRADD-RIPK1-DKO TRAIL+C vs Casp.8-KO TRAIL+Z	-65	***
TRADD-RIPK1-DKO TRAIL+C vs Casp.8-KO TRAIL+N	-86	***
TRADD-RIPK1-DKO TRAIL+C vs Casp.8-KO TRAIL+Z+N	-88	***
TRADD-RIPK1-DKO TRAIL+C vs Casp.8-KO TRAIL+C	-53	***
TRADD-RIPK1-DKO TRAIL+C vs Casp.8-KO TRAIL+C+Z	-50	***
TRADD-RIPK1-DKO TRAIL+C vs Casp.8-KO TRAIL+C+N	-79	***
TRADD-RIPK1-DKO TRAIL+C vs Casp.8-KO TRAIL+C+Z+N	-84	***
TRADD-RIPK1-DKO TRAIL+C vs EV TRAIL	-85	***
TRADD-RIPK1-DKO TRAIL+C vs EV TRAIL+Z	-86	***
TRADD-RIPK1-DKO TRAIL+C vs EV TRAIL+N	-69	***
TRADD-RIPK1-DKO TRAIL+C vs EV TRAIL+Z+N	-81	***
TRADD-RIPK1-DKO TRAIL+C vs EV TRAIL+C	-8,5	ns
TRADD-RIPK1-DKO TRAIL+C vs EV TRAIL+C+Z	-84	***
TRADD-RIPK1-DKO TRAIL+C vs EV TRAIL+C+N	-5,3	ns
TRADD-RIPK1-DKO TRAIL+C vs EV TRAIL+C+Z+N	-81	***
TRADD-RIPK1-DKO TRAIL+C+Z vs TRADD-RIPK1-DKO TRAIL+C+N	87	***
TRADD-RIPK1-DKO TRAIL+C+Z vs TRADD-RIPK1-DKO TRAIL+C+Z+N	1,0	ns
TRADD-RIPK1-DKO TRAIL+C+Z vs Casp.8-KO TRAIL	12	ns
TRADD-RIPK1-DKO TRAIL+C+Z vs Casp.8-KO TRAIL+Z	21	**
TRADD-RIPK1-DKO TRAIL+C+Z vs Casp.8-KO TRAIL+N	-0,47	ns
TRADD-RIPK1-DKO TRAIL+C+Z vs Casp.8-KO TRAIL+Z+N	-2,0	ns
TRADD-RIPK1-DKO TRAIL+C+Z vs Casp.8-KO TRAIL+C	33	***
TRADD-RIPK1-DKO TRAIL+C+Z vs Casp.8-KO TRAIL+C+Z	36	***
TRADD-RIPK1-DKO TRAIL+C+Z vs Casp.8-KO TRAIL+C+N	6,6	ns
TRADD-RIPK1-DKO TRAIL+C+Z vs Casp.8-KO TRAIL+C+Z+N	1,5	ns
TRADD-RIPK1-DKO TRAIL+C+Z vs EV TRAIL	1,3	ns
TRADD-RIPK1-DKO TRAIL+C+Z vs EV TRAIL+Z	0,13	ns
TRADD-RIPK1-DKO TRAIL+C+Z vs EV TRAIL+N	17	ns
TRADD-RIPK1-DKO TRAIL+C+Z vs EV TRAIL+Z+N	5,1	ns

TRADD-RIPK1-DKO TRAIL+C+Z vs EV TRAIL+C	77	***
TRADD-RIPK1-DKO TRAIL+C+Z vs EV TRAIL+C+Z	1,7	ns
TRADD-RIPK1-DKO TRAIL+C+Z vs EV TRAIL+C+N	81	***
TRADD-RIPK1-DKO TRAIL+C+Z vs EV TRAIL+C+Z+N	4,9	ns
TRADD-RIPK1-DKO TRAIL+C+N vs TRADD-RIPK1-DKO TRAIL+C+Z+N	-86	***
TRADD-RIPK1-DKO TRAIL+C+N vs Casp.8-KO TRAIL	-74	***
TRADD-RIPK1-DKO TRAIL+C+N vs Casp.8-KO TRAIL+Z	-66	***
TRADD-RIPK1-DKO TRAIL+C+N vs Casp.8-KO TRAIL+N	-87	***
TRADD-RIPK1-DKO TRAIL+C+N vs Casp.8-KO TRAIL+Z+N	-89	***
TRADD-RIPK1-DKO TRAIL+C+N vs Casp.8-KO TRAIL+C	-54	***
TRADD-RIPK1-DKO TRAIL+C+N vs Casp.8-KO TRAIL+C+Z	-51	***
TRADD-RIPK1-DKO TRAIL+C+N vs Casp.8-KO TRAIL+C+N	-80	***
TRADD-RIPK1-DKO TRAIL+C+N vs Casp.8-KO TRAIL+C+Z+N	-85	***
TRADD-RIPK1-DKO TRAIL+C+N vs EV TRAIL	-85	***
TRADD-RIPK1-DKO TRAIL+C+N vs EV TRAIL+Z	-87	***
TRADD-RIPK1-DKO TRAIL+C+N vs EV TRAIL+N	-70	***
TRADD-RIPK1-DKO TRAIL+C+N vs EV TRAIL+Z+N	-82	***
TRADD-RIPK1-DKO TRAIL+C+N vs EV TRAIL+C	-9,2	ns
TRADD-RIPK1-DKO TRAIL+C+N vs EV TRAIL+C+Z	-85	***
TRADD-RIPK1-DKO TRAIL+C+N vs EV TRAIL+C+N	-6,0	ns
TRADD-RIPK1-DKO TRAIL+C+N vs EV TRAIL+C+Z+N	-82	***
TRADD-RIPK1-DKO TRAIL+C+Z+N vs Casp.8-KO TRAIL	11	ns
TRADD-RIPK1-DKO TRAIL+C+Z+N vs Casp.8-KO TRAIL+Z	20	**
TRADD-RIPK1-DKO TRAIL+C+Z+N vs Casp.8-KO TRAIL+N	-1,5	ns
TRADD-RIPK1-DKO TRAIL+C+Z+N vs Casp.8-KO TRAIL+Z+N	-3,0	ns
TRADD-RIPK1-DKO TRAIL+C+Z+N vs Casp.8-KO TRAIL+C	32	***
TRADD-RIPK1-DKO TRAIL+C+Z+N vs Casp.8-KO TRAIL+C+Z	35	***
TRADD-RIPK1-DKO TRAIL+C+Z+N vs Casp.8-KO TRAIL+C+N	5,6	ns
TRADD-RIPK1-DKO TRAIL+C+Z+N vs Casp.8-KO TRAIL+C+Z+N	0,49	ns
TRADD-RIPK1-DKO TRAIL+C+Z+N vs EV TRAIL	0,22	ns
TRADD-RIPK1-DKO TRAIL+C+Z+N vs EV TRAIL+Z	-0,91	ns
TRADD-RIPK1-DKO TRAIL+C+Z+N vs EV TRAIL+N	16	ns
TRADD-RIPK1-DKO TRAIL+C+Z+N vs EV TRAIL+Z+N	4,1	ns
TRADD-RIPK1-DKO TRAIL+C+Z+N vs EV TRAIL+C	76	***
TRADD-RIPK1-DKO TRAIL+C+Z+N vs EV TRAIL+C+Z	0,66	ns
TRADD-RIPK1-DKO TRAIL+C+Z+N vs EV TRAIL+C+N	80	***
TRADD-RIPK1-DKO TRAIL+C+Z+N vs EV TRAIL+C+Z+N	3,8	ns
Casp.8-KO TRAIL vs Casp.8-KO TRAIL+Z	8,9	ns
Casp.8-KO TRAIL vs Casp.8-KO TRAIL+N	-13	ns
Casp.8-KO TRAIL vs Casp.8-KO TRAIL+Z+N	-14	ns
Casp.8-KO TRAIL vs Casp.8-KO TRAIL+C	21	***
Casp.8-KO TRAIL vs Casp.8-KO TRAIL+C+Z	24	***
Casp.8-KO TRAIL vs Casp.8-KO TRAIL+C+N	-5,6	ns
Casp.8-KO TRAIL vs Casp.8-KO TRAIL+C+Z+N	-11	ns
Casp.8-KO TRAIL vs EV TRAIL	-11	ns
Casp.8-KO TRAIL vs EV TRAIL+Z	-12	ns
Casp.8-KO TRAIL vs EV TRAIL+N	4,9	ns
Casp.8-KO TRAIL vs EV TRAIL+Z+N	-7,1	ns
Casp.8-KO TRAIL vs EV TRAIL+C	65	***
Casp.8-KO TRAIL vs EV TRAIL+C+Z	-10	ns
Casp.8-KO TRAIL vs EV TRAIL+C+N	68	***
Casp.8-KO TRAIL vs EV TRAIL+C+Z+N	-7,3	ns
Casp.8-KO TRAIL+Z vs Casp.8-KO TRAIL+N	-22	***
Casp.8-KO TRAIL+Z vs Casp.8-KO TRAIL+Z+N	-23	***

Casp.8-KO TRAIL+Z vs Casp.8-KO TRAIL+C	12	ns
Casp.8-KO TRAIL+Z vs Casp.8-KO TRAIL+C+Z	15	ns
Casp.8-KO TRAIL+Z vs Casp.8-KO TRAIL+C+N	-15	ns
Casp.8-KO TRAIL+Z vs Casp.8-KO TRAIL+C+Z+N	-20	**
Casp.8-KO TRAIL+Z vs EV TRAIL	-20	*
Casp.8-KO TRAIL+Z vs EV TRAIL+Z	-21	*
Casp.8-KO TRAIL+Z vs EV TRAIL+N	-4,0	ns
Casp.8-KO TRAIL+Z vs EV TRAIL+Z+N	-16	ns
Casp.8-KO TRAIL+Z vs EV TRAIL+C	56	***
Casp.8-KO TRAIL+Z vs EV TRAIL+C+Z	-19	ns
Casp.8-KO TRAIL+Z vs EV TRAIL+C+N	60	***
Casp.8-KO TRAIL+Z vs EV TRAIL+C+Z+N	-16	ns
Casp.8-KO TRAIL+N vs Casp.8-KO TRAIL+Z+N	-1,5	ns
Casp.8-KO TRAIL+N vs Casp.8-KO TRAIL+C	34	***
Casp.8-KO TRAIL+N vs Casp.8-KO TRAIL+C+Z	36	***
Casp.8-KO TRAIL+N vs Casp.8-KO TRAIL+C+N	7,1	ns
Casp.8-KO TRAIL+N vs Casp.8-KO TRAIL+C+Z+N	2,0	ns
Casp.8-KO TRAIL+N vs EV TRAIL	1,7	ns
Casp.8-KO TRAIL+N vs EV TRAIL+Z	0,60	ns
Casp.8-KO TRAIL+N vs EV TRAIL+N	18	ns
Casp.8-KO TRAIL+N vs EV TRAIL+Z+N	5,6	ns
Casp.8-KO TRAIL+N vs EV TRAIL+C	78	***
Casp.8-KO TRAIL+N vs EV TRAIL+C+Z	2,2	ns
Casp.8-KO TRAIL+N vs EV TRAIL+C+N	81	***
Casp.8-KO TRAIL+N vs EV TRAIL+C+Z+N	5,3	ns
Casp.8-KO TRAIL+Z+N vs Casp.8-KO TRAIL+C	35	***
Casp.8-KO TRAIL+Z+N vs Casp.8-KO TRAIL+C+Z	38	***
Casp.8-KO TRAIL+Z+N vs Casp.8-KO TRAIL+C+N	8,6	ns
Casp.8-KO TRAIL+Z+N vs Casp.8-KO TRAIL+C+Z+N	3,5	ns
Casp.8-KO TRAIL+Z+N vs EV TRAIL	3,2	ns
Casp.8-KO TRAIL+Z+N vs EV TRAIL+Z	2,1	ns
Casp.8-KO TRAIL+Z+N vs EV TRAIL+N	19	ns
Casp.8-KO TRAIL+Z+N vs EV TRAIL+Z+N	7,1	ns
Casp.8-KO TRAIL+Z+N vs EV TRAIL+C	79	***
Casp.8-KO TRAIL+Z+N vs EV TRAIL+C+Z	3,7	ns
Casp.8-KO TRAIL+Z+N vs EV TRAIL+C+N	83	***
Casp.8-KO TRAIL+Z+N vs EV TRAIL+C+Z+N	6,9	ns
Casp.8-KO TRAIL+C vs Casp.8-KO TRAIL+C+Z	2,8	ns
Casp.8-KO TRAIL+C vs Casp.8-KO TRAIL+C+N	-27	***
Casp.8-KO TRAIL+C vs Casp.8-KO TRAIL+C+Z+N	-32	***
Casp.8-KO TRAIL+C vs EV TRAIL	-32	***
Casp.8-KO TRAIL+C vs EV TRAIL+Z	-33	***
Casp.8-KO TRAIL+C vs EV TRAIL+N	-16	ns
Casp.8-KO TRAIL+C vs EV TRAIL+Z+N	-28	**
Casp.8-KO TRAIL+C vs EV TRAIL+C	44	***
Casp.8-KO TRAIL+C vs EV TRAIL+C+Z	-31	***
Casp.8-KO TRAIL+C vs EV TRAIL+C+N	48	***
Casp.8-KO TRAIL+C vs EV TRAIL+C+Z+N	-28	**
Casp.8-KO TRAIL+C+Z vs Casp.8-KO TRAIL+C+N	-29	***
Casp.8-KO TRAIL+C+Z vs Casp.8-KO TRAIL+C+Z+N	-34	***
Casp.8-KO TRAIL+C+Z vs EV TRAIL	-35	***
Casp.8-KO TRAIL+C+Z vs EV TRAIL+Z	-36	***
Casp.8-KO TRAIL+C+Z vs EV TRAIL+N	-19	ns
Casp.8-KO TRAIL+C+Z vs EV TRAIL+Z+N	-31	***

Casp.8-KO TRAIL+C+Z vs EV TRAIL+C	42	***
Casp.8-KO TRAIL+C+Z vs EV TRAIL+C+Z	-34	***
Casp.8-KO TRAIL+C+Z vs EV TRAIL+C+N	45	***
Casp.8-KO TRAIL+C+Z vs EV TRAIL+C+Z+N	-31	***
Casp.8-KO TRAIL+C+N vs Casp.8-KO TRAIL+C+Z+N	-5,1	ns
Casp.8-KO TRAIL+C+N vs EV TRAIL	-5,3	ns
Casp.8-KO TRAIL+C+N vs EV TRAIL+Z	-6,5	ns
Casp.8-KO TRAIL+C+N vs EV TRAIL+N	11	ns
Casp.8-KO TRAIL+C+N vs EV TRAIL+Z+N	-1,5	ns
Casp.8-KO TRAIL+C+N vs EV TRAIL+C	71	***
Casp.8-KO TRAIL+C+N vs EV TRAIL+C+Z	-4,9	ns
Casp.8-KO TRAIL+C+N vs EV TRAIL+C+N	74	***
Casp.8-KO TRAIL+C+N vs EV TRAIL+C+Z+N	-1,7	ns
Casp.8-KO TRAIL+C+Z+N vs EV TRAIL	-0,28	ns
Casp.8-KO TRAIL+C+Z+N vs EV TRAIL+Z	-1,4	ns
Casp.8-KO TRAIL+C+Z+N vs EV TRAIL+N	16	ns
Casp.8-KO TRAIL+C+Z+N vs EV TRAIL+Z+N	3,6	ns
Casp.8-KO TRAIL+C+Z+N vs EV TRAIL+C	76	***
Casp.8-KO TRAIL+C+Z+N vs EV TRAIL+C+Z	0,17	ns
Casp.8-KO TRAIL+C+Z+N vs EV TRAIL+C+N	79	***
Casp.8-KO TRAIL+C+Z+N vs EV TRAIL+C+Z+N	3,3	ns
EV TRAIL vs EV TRAIL+Z	-1,1	ns
EV TRAIL vs EV TRAIL+N	16	ns
EV TRAIL vs EV TRAIL+Z+N	3,9	ns
EV TRAIL vs EV TRAIL+C	76	***
EV TRAIL vs EV TRAIL+C+Z	0,45	ns
EV TRAIL vs EV TRAIL+C+N	79	***
EV TRAIL vs EV TRAIL+C+Z+N	3,6	ns
EV TRAIL+Z vs EV TRAIL+N	17	ns
EV TRAIL+Z vs EV TRAIL+Z+N	5,0	ns
EV TRAIL+Z vs EV TRAIL+C	77	***
EV TRAIL+Z vs EV TRAIL+C+Z	1,6	ns
EV TRAIL+Z vs EV TRAIL+C+N	81	***
EV TRAIL+Z vs EV TRAIL+C+Z+N	4,8	ns
EV TRAIL+N vs EV TRAIL+Z+N	-12	ns
EV TRAIL+N vs EV TRAIL+C	60	***
EV TRAIL+N vs EV TRAIL+C+Z	-15	ns
EV TRAIL+N vs EV TRAIL+C+N	64	***
EV TRAIL+N vs EV TRAIL+C+Z+N	-12	ns
EV TRAIL+Z+N vs EV TRAIL+C	72	***
EV TRAIL+Z+N vs EV TRAIL+C+Z	-3,4	ns
EV TRAIL+Z+N vs EV TRAIL+C+N	76	***
EV TRAIL+Z+N vs EV TRAIL+C+Z+N	-0,25	ns
EV TRAIL+C vs EV TRAIL+C+Z	-76	***
EV TRAIL+C vs EV TRAIL+C+N	3,2	ns
EV TRAIL+C vs EV TRAIL+C+Z+N	-73	***
EV TRAIL+C+Z vs EV TRAIL+C+N	79	***
EV TRAIL+C+Z vs EV TRAIL+C+Z+N	3,2	ns
EV TRAIL+C+N vs EV TRAIL+C+Z+N	-76	***
